# Diretriz Brasileira de Dispositivos Cardíacos Eletrônicos Implantáveis – 2023

**DOI:** 10.36660/abc.20220892

**Published:** 2023-01-16

**Authors:** Ricardo Alkmim Teixeira, Alexsandro Alves Fagundes, José Mário Baggio, Júlio César de Oliveira, Paulo de Tarso Jorge Medeiros, Bruno Pereira Valdigem, Luiz Antônio Castilho Teno, Rodrigo Tavares Silva, Celso Salgado de Melo, Jorge Elias, Antonio Vitor Moraes, Anisio Alexandre Andrade Pedrosa, Fernando Mello Porto, Hélio Lima de Brito, Thiago Gonçalves Schroder e Souza, José Carlos Pachón Mateos, Luis Gustavo Belo de Moraes, Alexander Romeno Janner Dal Forno, Andre Luiz Buchele D’Avila, Diogo Alberto de Magalhães Cavaco, Ricardo Ryoshim Kuniyoshi, Mauricio Pimentel, Luiz Eduardo Montenegro Camanho, Eduardo Benchimol Saad, Leandro Ioschpe Zimerman, Eduardo Bartholomay Oliveira, Mauricio Ibrahim Scanavacca, Martino Martinelli, Carlos Eduardo Batista de Lima, Giselle de Lima Peixoto, Francisco Carlos da Costa Darrieux, Jussara de Oliveira Pinheiro Duarte, Silas dos Santos Galvão, Eduardo Rodrigues Bento Costa, Enrique Indalécio Pachón Mateo, Sissy Lara De Melo, Thiago da Rocha Rodrigues, Eduardo Arrais Rocha, Denise Tessariol Hachul, Adalberto Menezes Lorga, Silvana Angelina D’Orio Nishioka, Eduardo Barreto Gadelha, Roberto Costa, Veridiana Silva de Andrade, Gustavo Gomes Torres, Nestor Rodrigues de Oliveira, Fernando Antonio Lucchese, Henrique Murad, José Wanderley, Paulo Roberto Slud Brofman, Rui M. S. Almeida, João Carlos Ferreira Leal

**Affiliations:** 1 Hospital Renascentista Pouso Alegre MG Brasil Hospital Renascentista, Pouso Alegre, MG – Brasil; 2 Hospital Ana Nery Salvador BA Brasil Hospital Ana Nery, Salvador, BA – Brasil; 3 Instituto de Cardiologia do Distrito Federal Brasília DF Brasil Instituto de Cardiologia do Distrito Federal, Brasília, DF – Brasil; 4 Universidade Federal de Mato Grosso Cuiabá MT Brasil Universidade Federal de Mato Grosso (UFMT),Cuiabá, MT – Brasil; 5 Instituto Dante Pazzanese de Cardiologia São Paulo SP Brasil Instituto Dante Pazzanese de Cardiologia, São Paulo, SP – Brasil; 6 Clínica Cardiovascular Ribeirão Preto Ribeirão Preto SP Brasil Clínica Cardiovascular Ribeirão Preto, Ribeirão Preto, SP – Brasil; 7 Universidade de Franca Franca SP Brasil Universidade de Franca (UNIFRAN), Franca, SP – Brasil; 8 Centro Universitário Municipal de Franca Franca SP Brasil Centro Universitário Municipal de Franca (Uni-FACEF), Franca, SP – Brasil; 9 Clínica de Marca-passos Cardíacos Uberaba MG Brasil Clínica de Marca-passos Cardíacos, Uberaba, MG – Brasil; 10 Universidade Federal do Espírito Santo Vitória ES Brasil Universidade Federal do Espírito Santo (UFES), Vitória, ES – Brasil; 11 Santa Casa de Ribeirão Preto Ribeirão Preto SP Brasil Santa Casa de Ribeirão Preto, Ribeirão Preto, SP – Brasil; 12 Unimed de Ribeirão Preto Ribeirão Preto SP Brasil Unimed de Ribeirão Preto, Ribeirão Preto, SP – Brasil; 13 Hospital das Clínicas Faculdade de Medicina Universidade de São Paulo São Paulo SP Brasil Instituto do Coração (Incor) do Hospital das Clínicas da Faculdade de Medicina da Universidade de São Paulo (FMUSP), São Paulo, SP – Brasil; 14 Pontifícia Universidade Católica de Campinas Campinas SP Brasil Pontifícia Universidade Católica de Campinas, Campinas, SP – Brasil; 15 Universidade Federal de Juiz de Fora Juiz de Fora MG Brasil Universidade Federal de Juiz de Fora, Juiz de Fora, MG – Brasil; 16 Hospital Universitário Universidade Federal de Juiz de Fora Juiz de Fora MG Brasil Hospital Universitário da Universidade Federal de Juiz de Fora (UFJF), Juiz de Fora, MG – Brasil; 17 Hospital Universitário Clementino Fraga Filho Universidade Federal do Rio de Janeiro Rio de Janeiro RJ Brasil Hospital Universitário Clementino Fraga Filho, Universidade Federal do Rio de Janeiro (UFRJ), Rio de Janeiro, RJ – Brasil; 18 Hospital SOS Cárdio Florianópolis SC Brasil Hospital SOS Cárdio, Florianópolis, SC – Brasil; 19 Hospital de Santa Cruz Lisboa Portugal Hospital de Santa Cruz, Lisboa – Portugal; 20 Centrocor Vitória Vitória ES Brasil Centrocor Vitória, Vitória, ES – Brasil; 21 Vitória Apart Hospital Vitória ES Brasil Vitória Apart Hospital, Vitória, ES – Brasil; 22 Hospital de Clínicas de Porto Alegre Universidade Federal do Rio Grande do Sul Porto Alegre RS Brasil Hospital de Clínicas de Porto Alegre, Universidade Federal do Rio Grande do Sul (UFRGS), Porto Alegre, RS – Brasil; 23 Hospital Pró-Cardíaco Rio de Janeiro RJ Brasil Hospital Pró-Cardíaco, Rio de Janeiro, RJ – Brasil; 24 Hospital Samaritano Rio de Janeiro RJ Brasil Hospital Samaritano, Rio de Janeiro, RJ – Brasil; 25 Universidade Federal do Rio Grande do Sul Porto Alegre RS Brasil Universidade Federal do Rio Grande do Sul (UFRGS), Porto Alegre, RS – Brasil; 26 Hospital Mãe de Deus Porto Alegre RS Brasil Hospital Mãe de Deus, Porto Alegre, RS – Brasil; 27 Hospital Universitário Universidade Federal do Piauí Teresina PI Brasil Hospital Universitário da Universidade Federal do Piauí (UFPI), Teresina, PI – Brasil; 28 Empresa Brasileira de Serviços Hospitalares Brasília DF Brasil Empresa Brasileira de Serviços Hospitalares (EBSERH), Brasília, DF – Brasil; 29 DentCor Clínica Médica e Odontológica Santo André SP Brasil DentCor Clínica Médica e Odontológica, Santo André, SP – Brasil; 30 Hospital Universitário Professor Edgard Santos Universidade Federal da Bahia Salvador BA Brasil Hospital Universitário Professor Edgard Santos, Universidade Federal da Bahia (UFBA), Salvador, BA – Brasil; 31 Centro Avançado de Ritmologia e Eletrofisiologia Vista SP Brasil Centro Avançado de Ritmologia e Eletrofisiologia (CARE), Vista, SP – Brasil; 32 CardioRitmo Clínica de Arritmias Cardíacas São José do Rio Preto RP Brasil CardioRitmo, Clínica de Arritmias Cardíacas, São José do Rio Preto, RP – Brasil; 33 Serviço de Eletrofisiologia, Marca-passos e Arritmias São Paulo SP Brasil Serviço de Eletrofisiologia, Marca-passos e Arritmias (SEMAP),São Paulo, SP – Brasil; 34 Hospital Felício Rocho Belo Horizonte MG Brasil Hospital Felício Rocho, Belo Horizonte, MG – Brasil; 35 Hospital Universitário Walter Cantídio Universidade Federal do Ceará Fortaleza CE Brasil Hospital Universitário Walter Cantídio, Universidade Federal do Ceará (UFC), Fortaleza, CE – Brasil; 36 Instituto de Moléstias Cardiovasculares São José do Rio Preto SP Brasil Instituto de Moléstias Cardiovasculares (IMC), São José do Rio Preto, SP – Brasil; 37 Hospital Dom Helder Camara Recife PE Brasil Hospital Dom Helder Camara, Recife, PE – Brasil; 38 Faculdade de Medicina Universidade de São Paulo São Paulo SP Brasil Faculdade de Medicina da Universidade de São Paulo (FMUSP), São Paulo, SP – Brasil; 39 Universidade Federal de São Paulo São Paulo SP Brasil Universidade Federal de São Paulo (UNIFESP), São Paulo, SP – Brasil; 40 Hospital Universitário Onofre Lopes Universidade Federal do Rio Grande do Norte Natal RN Brasil Hospital Universitário Onofre Lopes, Universidade Federal do Rio Grande do Norte (UFRN), Natal, RN – Brasil; 41 Irmandade Santa Casa de Misericórdia de Porto Alegre Porto Alegre RS Brasil Irmandade Santa Casa de Misericórdia de Porto Alegre, Porto Alegre, RS – Brasil; 42 Universidade Federal do Rio de Janeiro Rio de Janeiro RJ Brasil Universidade Federal do Rio de Janeiro (UFRJ), Rio de Janeiro, RJ – Brasil; 43 Universidade Federal de Alagoas Maceió AL Brasil Universidade Federal de Alagoas (UFAL), Maceió, AL – Brasil; 44 Santa Casa Curitiba Curitiba PR Brasil Santa Casa Curitiba, Curitiba, PR – Brasil; 45 Centro Universitário Fundação Assis Gurgacz Cascavel PR Brasil Centro Universitário Fundação Assis Gurgacz, Cascavel, PR – Brasil; 46 Faculdade de Medicina de São José do Rio Preto São José do Rio Preto SP Brasil Faculdade de Medicina de São José do Rio Preto (FAMERP), São José do Rio Preto, SP – Brasil

**Table:** 

Diretriz Brasileira de Dispositivos Cardíacos Eletrônicos Implantáveis – 2023	
O relatório abaixo lista as declarações de interesse conforme relatadas à SBC pelos especialistas durante o período de desenvolvimento deste posicionamento, 2019/2022.	
Especialista	Tipo de relacionamento com a indústria
Adalberto Menezes Lorga Filho	Declaração financeira A - Pagamento de qualquer espécie e desde que economicamente apreciáveis, feitos a (i) você, (ii) ao seu cônjuge/ companheiro ou a qualquer outro membro que resida com você, (iii) a qualquer pessoa jurídica em que qualquer destes seja controlador, sócio, acionista ou participante, de forma direta ou indireta, recebimento por palestras, aulas, atuação como proctor de treinamentos, remunerações, honorários pagos por participações em conselhos consultivos, de investigadores, ou outros comitês, etc. Provenientes da indústria farmacêutica, de órteses, próteses, equipamentos e implantes, brasileiras ou estrangeiras: - Vatis / Libbs; Lixiana / Daiichi Sankyo; Eliquis / Pfizer: Fibrilação atrial. Outros relacionamentos Financiamento de atividades de educação médica continuada, incluindo viagens, hospedagens e inscrições para congressos e cursos, provenientes da indústria farmacêutica, de órteses, próteses, equipamentos e implantes, brasileiras ou estrangeiras: - Daiichi Sankyo: Lixiana
Alexander Romeno Janner Dal Forno	Declaração financeira A - Pagamento de qualquer espécie e desde que economicamente apreciáveis, feitos a (i) você, (ii) ao seu cônjuge/ companheiro ou a qualquer outro membro que resida com você, (iii) a qualquer pessoa jurídica em que qualquer destes seja controlador, sócio, acionista ou participante, de forma direta ou indireta, recebimento por palestras, aulas, atuação como proctor de treinamentos, remunerações, honorários pagos por participações em conselhos consultivos, de investigadores, ou outros comitês, etc. Provenientes da indústria farmacêutica, de órteses, próteses, equipamentos e implantes, brasileiras ou estrangeiras: - Biotronik: Marca-passo, Abbott: Fibrilação atrial. B - Financiamento de pesquisas sob sua responsabilidade direta/pessoal (direcionado ao departamento ou instituição) provenientes da indústria farmacêutica, de órteses, próteses, equipamentos e implantes, brasileiras ou estrangeiras: - Proadi.
Alexsandro Alves Fagundes	Declaração financeira A - Pagamento de qualquer espécie e desde que economicamente apreciáveis, feitos a (i) você, (ii) ao seu cônjuge/ companheiro ou a qualquer outro membro que resida com você, (iii) a qualquer pessoa jurídica em que qualquer destes seja controlador, sócio, acionista ou participante, de forma direta ou indireta, recebimento por palestras, aulas, atuação como proctor de treinamentos, remunerações, honorários pagos por participações em conselhos consultivos, de investigadores, ou outros comitês, etc. Provenientes da indústria farmacêutica, de órteses, próteses, equipamentos e implantes, brasileiras ou estrangeiras: - Bayer: Xarelto, Medtronic, Sankyo, Libbs. Outros relacionamentos Financiamento de atividades de educação médica continuada, incluindo viagens, hospedagens e inscrições para congressos e cursos, provenientes da indústria farmacêutica, de órteses, próteses, equipamentos e implantes, brasileiras ou estrangeiras: - Boston.
Andre Luiz Buchele D’Avila	Nada a ser declarado
Anisio Alexandre Andrade Pedrosa	Declaração financeira A - Pagamento de qualquer espécie e desde que economicamente apreciáveis, feitos a (i) você, (ii) ao seu cônjuge/ companheiro ou a qualquer outro membro que resida com você, (iii) a qualquer pessoa jurídica em que qualquer destes seja controlador, sócio, acionista ou participante, de forma direta ou indireta, recebimento por palestras, aulas, atuação como proctor de treinamentos, remunerações, honorários pagos por participações em conselhos consultivos, de investigadores, ou outros comitês, etc. Provenientes da indústria farmacêutica, de órteses, próteses, equipamentos e implantes, brasileiras ou estrangeiras: - Medtronic / Biomedical / Biocath / Imagem: Proctor Outros relacionamentos Financiamento de atividades de educação médica continuada, incluindo viagens, hospedagens e inscrições para congressos e cursos, provenientes da indústria farmacêutica, de órteses, próteses, equipamentos e implantes, brasileiras ou estrangeiras: - Medtronic, Biocath, Biomedical.
Antonio Vitor Moraes Júnior	Declaração financeira A - Pagamento de qualquer espécie e desde que economicamente apreciáveis, feitos a (i) você, (ii) ao seu cônjuge/ companheiro ou a qualquer outro membro que resida com você, (iii) a qualquer pessoa jurídica em que qualquer destes seja controlador, sócio, acionista ou participante, de forma direta ou indireta, recebimento por palestras, aulas, atuação como proctor de treinamentos, remunerações, honorários pagos por participações em conselhos consultivos, de investigadores, ou outros comitês, etc. Provenientes da indústria farmacêutica, de órteses, próteses, equipamentos e implantes, brasileiras ou estrangeiras: - Biotronik. Outros relacionamentos Financiamento de atividades de educação médica continuada, incluindo viagens, hospedagens e inscrições para congressos e cursos, provenientes da indústria farmacêutica, de órteses, próteses, equipamentos e implantes, brasileiras ou estrangeiras: - Xarelto / Bayer: Fibrilação atrial.
Bruno Pereira Valdigem	Nada a ser declarado
Carlos Eduardo Batista de Lima	Declaração financeira A - Pagamento de qualquer espécie e desde que economicamente apreciáveis, feitos a (i) você, (ii) ao seu cônjuge/ companheiro ou a qualquer outro membro que resida com você, (iii) a qualquer pessoa jurídica em que qualquer destes seja controlador, sócio, acionista ou participante, de forma direta ou indireta, recebimento por palestras, aulas, atuação como proctor de treinamentos, remunerações, honorários pagos por participações em conselhos consultivos, de investigadores, ou outros comitês, etc. Provenientes da indústria farmacêutica, de órteses, próteses, equipamentos e implantes, brasileiras ou estrangeiras: - Daiichi Sankyo / Lixiana: Fibrilação atrial C - Financiamento de pesquisa (pessoal), cujas receitas tenham sido provenientes da indústria farmacêutica, de órteses, próteses, equipamentos e implantes, brasileiras ou estrangeiras: - RIVER AF trial, Thrombosis Research Institute / Brazilian Clinical Research.
Celso Salgado de Melo	Nada a ser declarado
Denise Tessariol Hachul	Nada a ser declarado
Diogo Alberto de Magalhães Cavaco	Declaração financeira A - Pagamento de qualquer espécie e desde que economicamente apreciáveis, feitos a (i) você, (ii) ao seu cônjuge/ companheiro ou a qualquer outro membro que resida com você, (iii) a qualquer pessoa jurídica em que qualquer destes seja controlador, sócio, acionista ou participante, de forma direta ou indireta, recebimento por palestras, aulas, atuação como proctor de treinamentos, remunerações, honorários pagos por participações em conselhos consultivos, de investigadores, ou outros comitês, etc. Provenientes da indústria farmacêutica, de órteses, próteses, equipamentos e implantes, brasileiras ou estrangeiras: - Boston Scientific/S-ICD.
Eduardo Arrais Rocha	Declaração financeira A - Pagamento de qualquer espécie e desde que economicamente apreciáveis, feitos a (i) você, (ii) ao seu cônjuge/ companheiro ou a qualquer outro membro que resida com você, (iii) a qualquer pessoa jurídica em que qualquer destes seja controlador, sócio, acionista ou participante, de forma direta ou indireta, recebimento por palestras, aulas, atuação como proctor de treinamentos, remunerações, honorários pagos por participações em conselhos consultivos, de investigadores, ou outros comitês, etc. Provenientes da indústria farmacêutica, de órteses, próteses, equipamentos e implantes, brasileiras ou estrangeiras: - ABBOTT: aula sobre monitoramento remoto. Outros relacionamentos Financiamento de atividades de educação médica continuada, incluindo viagens, hospedagens e inscrições para congressos e cursos, provenientes da indústria farmacêutica, de órteses, próteses, equipamentos e implantes, brasileiras ou estrangeiras: - Biotronik / Bayer: Congresso. Participação societária de qualquer natureza e qualquer valor economicamente apreciável de empresas na área de saúde, de ensino ou em empresas concorrentes ou fornecedoras da SBC: - Área de Saúde.
Eduardo Barreto Gadelha	Nada a ser declarado
Eduardo Bartholomay Oliveira	Declaração financeira A - Pagamento de qualquer espécie e desde que economicamente apreciáveis, feitos a (i) você, (ii) ao seu cônjuge/ companheiro ou a qualquer outro membro que resida com você, (iii) a qualquer pessoa jurídica em que qualquer destes seja controlador, sócio, acionista ou participante, de forma direta ou indireta, recebimento por palestras, aulas, atuação como proctor de treinamentos, remunerações, honorários pagos por participações em conselhos consultivos, de investigadores, ou outros comitês, etc. Provenientes da indústria farmacêutica, de órteses, próteses, equipamentos e implantes, brasileiras ou estrangeiras: - Biotronik.
Eduardo Benchimol Saad	Declaração financeira A - Pagamento de qualquer espécie e desde que economicamente apreciáveis, feitos a (i) você, (ii) ao seu cônjuge/ companheiro ou a qualquer outro membro que resida com você, (iii) a qualquer pessoa jurídica em que qualquer destes seja controlador, sócio, acionista ou participante, de forma direta ou indireta, recebimento por palestras, aulas, atuação como proctor de treinamentos, remunerações, honorários pagos por participações em conselhos consultivos, de investigadores, ou outros comitês, etc. Provenientes da indústria farmacêutica, de órteses, próteses, equipamentos e implantes, brasileiras ou estrangeiras: - Biosense Webster: Ablação por cateter.
Eduardo Rodrigues Bento Costa	Nada a ser declarado
Enrique Indalécio Pachón Mateo	Nada a ser declarado
Fernando Antônio Lucchese	Nada a ser declarado
Fernando Mello Porto	Nada a ser declarado
Francisco Carlos da Costa Darrieux	Declaração financeira A - Pagamento de qualquer espécie e desde que economicamente apreciáveis, feitos a (i) você, (ii) ao seu cônjuge/ companheiro ou a qualquer outro membro que resida com você, (iii) a qualquer pessoa jurídica em que qualquer destes seja controlador, sócio, acionista ou participante, de forma direta ou indireta, recebimento por palestras, aulas, atuação como proctor de treinamentos, remunerações, honorários pagos por participações em conselhos consultivos, de investigadores, ou outros comitês, etc. Provenientes da indústria farmacêutica, de órteses, próteses, equipamentos e implantes, brasileiras ou estrangeiras: - Bayer / Boehringer Ingelheim / Pfizer / Libbs / Biolab: Anticoagulação e fibrilação atrial. Outros relacionamentos Financiamento de atividades de educação médica continuada, incluindo viagens, hospedagens e inscrições para congressos e cursos, provenientes da indústria farmacêutica, de órteses, próteses, equipamentos e implantes, brasileiras ou estrangeiras: - Bayer / Pfizer: Congressos e Cursos Virtuais.
Giselle de Lima Peixoto	Nada a ser declarado
Gustavo Gomes Torres	Declaração financeira A - Pagamento de qualquer espécie e desde que economicamente apreciáveis, feitos a (i) você, (ii) ao seu cônjuge/ companheiro ou a qualquer outro membro que resida com você, (iii) a qualquer pessoa jurídica em que qualquer destes seja controlador, sócio, acionista ou participante, de forma direta ou indireta, recebimento por palestras, aulas, atuação como proctor de treinamentos, remunerações, honorários pagos por participações em conselhos consultivos, de investigadores, ou outros comitês, etc. Provenientes da indústria farmacêutica, de órteses, próteses, equipamentos e implantes, brasileiras ou estrangeiras: - Biotronik.
Hélio Lima de Brito Júnior	Outros relacionamentos Participação societária de qualquer natureza e qualquer valor economicamente apreciável de empresas na área de saúde, de ensino ou em empresas concorrentes ou fornecedoras da SBC: - Área de Saúde.
Henrique Murad	Nada a ser declarado
Jorge Elias Neto	Nada a ser declarado
João Carlos Ferreira Leal	Declaração financeira B - Financiamento de pesquisas sob sua responsabilidade direta/pessoal (direcionado ao departamento ou instituição) provenientes da indústria farmacêutica, de órteses, próteses, equipamentos e implantes, brasileiras ou estrangeiras: - Braile Biomédica: Inovare® Alpha - Prótese Valvular Biológica / Artivion-Neomex: Evita open plus, prótese híbrida.
José Carlos Pachón Mateos	Nada a ser declarado
José Mário Baggio Junior	Declaração financeira A - Pagamento de qualquer espécie e desde que economicamente apreciáveis, feitos a (i) você, (ii) ao seu cônjuge/ companheiro ou a qualquer outro membro que resida com você, (iii) a qualquer pessoa jurídica em que qualquer destes seja controlador, sócio, acionista ou participante, de forma direta ou indireta, recebimento por palestras, aulas, atuação como proctor de treinamentos, remunerações, honorários pagos por participações em conselhos consultivos, de investigadores, ou outros comitês, etc. Provenientes da indústria farmacêutica, de órteses, próteses, equipamentos e implantes, brasileiras ou estrangeiras: - Abbott: Palestra em Webinar; Biotronik: Simpósio satélite em congresso; Medtronic: Simpósio satélite em congresso. Outros relacionamentos Financiamento de atividades de educação médica continuada, incluindo viagens, hospedagens e inscrições para congressos e cursos, provenientes da indústria farmacêutica, de órteses, próteses, equipamentos e implantes, brasileiras ou estrangeiras: - Medtronic: Treinamento sobre "leadless pacing" e estimulação fisiológica; Biotronik: Congresso; Abbott: Treinamento de produtos.
José Wanderley Neto	Nada a ser declarado
Júlio César de Oliveira	Declaração financeira A - Pagamento de qualquer espécie e desde que economicamente apreciáveis, feitos a (i) você, (ii) ao seu cônjuge/ companheiro ou a qualquer outro membro que resida com você, (iii) a qualquer pessoa jurídica em que qualquer destes seja controlador, sócio, acionista ou participante, de forma direta ou indireta, recebimento por palestras, aulas, atuação como proctor de treinamentos, remunerações, honorários pagos por participações em conselhos consultivos, de investigadores, ou outros comitês, etc. Provenientes da indústria farmacêutica, de órteses, próteses, equipamentos e implantes, brasileiras ou estrangeiras: - Infinity.
Jussara de Oliveira Pinheiro Duarte	Nada a ser declarado
Leandro Ioschpe Zimerman	Declaração financeira A - Pagamento de qualquer espécie e desde que economicamente apreciáveis, feitos a (i) você, (ii) ao seu cônjuge/ companheiro ou a qualquer outro membro que resida com você, (iii) a qualquer pessoa jurídica em que qualquer destes seja controlador, sócio, acionista ou participante, de forma direta ou indireta, recebimento por palestras, aulas, atuação como proctor de treinamentos, remunerações, honorários pagos por participações em conselhos consultivos, de investigadores, ou outros comitês, etc. Provenientes da indústria farmacêutica, de órteses, próteses, equipamentos e implantes, brasileiras ou estrangeiras: - Bayer/Xarelto; Daiichi Sankyo/Lixiana; Libbs/Propafenona e Amiodarona; Pfizer/Eliquis.
Luis Gustavo Belo de Moraes	Outros relacionamentos Participação societária de qualquer natureza e qualquer valor economicamente apreciável de empresas na área de saúde, de ensino ou em empresas concorrentes ou fornecedoras da SBC: - Sócio cotista da Cardioritmo Serviços Médicos Ltda e Luis Belo Serviços Médicos.
Luiz Antônio Castilho Teno	Nada a ser declarado
Luiz Eduardo Montenegro Camanho	Nada a ser declarado
Martino Martinelli Filho	Nada a ser declarado
Mauricio Ibrahim Scanavacca	Declaração financeira A - Pagamento de qualquer espécie e desde que economicamente apreciáveis, feitos a (i) você, (ii) ao seu cônjuge/ companheiro ou a qualquer outro membro que resida com você, (iii) a qualquer pessoa jurídica em que qualquer destes seja controlador, sócio, acionista ou participante, de forma direta ou indireta, recebimento por palestras, aulas, atuação como proctor de treinamentos, remunerações, honorários pagos por participações em conselhos consultivos, de investigadores, ou outros comitês, etc. Provenientes da indústria farmacêutica, de órteses, próteses, equipamentos e implantes, brasileiras ou estrangeiras: - Johnson & Johnson. B - Financiamento de pesquisas sob sua responsabilidade direta/pessoal (direcionado ao departamento ou instituição) provenientes da indústria farmacêutica, de órteses, próteses, equipamentos e implantes, brasileiras ou estrangeiras: - Milestone Pharmaceuticals: etripamil NS for the treatment of Paroxysmal. C - Financiamento de pesquisa (pessoal), cujas receitas tenham sido provenientes da indústria farmacêutica, de órteses, próteses, equipamentos e implantes, brasileiras ou estrangeiras: - Medtronic, J&J and Abbott.
Mauricio Pimentel	Declaração financeira A - Pagamento de qualquer espécie e desde que economicamente apreciáveis, feitos a (i) você, (ii) ao seu cônjuge/ companheiro ou a qualquer outro membro que resida com você, (iii) a qualquer pessoa jurídica em que qualquer destes seja controlador, sócio, acionista ou participante, de forma direta ou indireta, recebimento por palestras, aulas, atuação como proctor de treinamentos, remunerações, honorários pagos por participações em conselhos consultivos, de investigadores, ou outros comitês, etc. Provenientes da indústria farmacêutica, de órteses, próteses, equipamentos e implantes, brasileiras ou estrangeiras: - Bayer: Anticoagulação.
Nestor Rodrigues de Oliveira Neto	Nada a ser declarado
Paulo Roberto Slud Brofman	Nada a ser declarado
Paulo de Tarso Jorge Medeiros	Nada a ser declarado
Ricardo Alkmim Teixeira	Declaração financeira A - Pagamento de qualquer espécie e desde que economicamente apreciáveis, feitos a (i) você, (ii) ao seu cônjuge/ companheiro ou a qualquer outro membro que resida com você, (iii) a qualquer pessoa jurídica em que qualquer destes seja controlador, sócio, acionista ou participante, de forma direta ou indireta, recebimento por palestras, aulas, atuação como proctor de treinamentos, remunerações, honorários pagos por participações em conselhos consultivos, de investigadores, ou outros comitês, etc. Provenientes da indústria farmacêutica, de órteses, próteses, equipamentos e implantes, brasileiras ou estrangeiras: - Boehringer-Ingelheim: Pradaxa, Jardiance; Daiichi-Sankyo: Lixiana; Abbott: Dispositivos Cardíacos Eletrônicos Implantáveis; Biotronik: Dispositivos Cardíacos Eletrônicos Implantáveis; Medtronic: Dispositivos Cardíacos Eletrônicos Implantáveis; Biomedical: Extração de Cabos-Eletrodos de Dispositivos Cardíacos Eletrônicos Implantáveis. Outros relacionamentos Financiamento de atividades de educação médica continuada, incluindo viagens, hospedagens e inscrições para congressos e cursos, provenientes da indústria farmacêutica, de órteses, próteses, equipamentos e implantes, brasileiras ou estrangeiras: - Biomedical: Extração de Cabos-Eletrodos de Dispositivos Cardíacos Eletrônicos Implantáveis.
Ricardo Ryoshim Kuniyoshi	Nada a ser declarado
Roberto Costa	Declaração financeira A - Pagamento de qualquer espécie e desde que economicamente apreciáveis, feitos a (i) você, (ii) ao seu cônjuge/ companheiro ou a qualquer outro membro que resida com você, (iii) a qualquer pessoa jurídica em que qualquer destes seja controlador, sócio, acionista ou participante, de forma direta ou indireta, recebimento por palestras, aulas, atuação como proctor de treinamentos, remunerações, honorários pagos por participações em conselhos consultivos, de investigadores, ou outros comitês, etc. Provenientes da indústria farmacêutica, de órteses, próteses, equipamentos e implantes, brasileiras ou estrangeiras: - Boston Scientific: Palestrante e consultor. Outros relacionamentos Participação em comitês de compras de materiais ou fármacos em instituições de saúde ou funções assemelhadas: - Parecerista dos pregões do Hospital das Clínicas da FMUSP.
Rodrigo Tavares Silva	Nada a ser declarado
Rui M. S. Almeida	Outros relacionamentos Participação societária de qualquer natureza e qualquer valor economicamente apreciável de empresas na área de saúde, de ensino ou em empresas concorrentes ou fornecedoras da SBC: - Área da Saúde e Educação.
Silas dos Santos Galvão Filho	Nada a ser declarado
Silvana Angelina D’Orio Nishioka	Outros relacionamentos Financiamento de atividades de educação médica continuada, incluindo viagens, hospedagens e inscrições para congressos e cursos, provenientes da indústria farmacêutica, de órteses, próteses, equipamentos e implantes, brasileiras ou estrangeiras: - Abbott. Atuação no último ano como auditor médico para empresa operadora de planos de saúde ou assemelhada: - Somente para aulas e treinamento médico.
Sissy Lara de Melo	Nada a ser declarado
Thiago da Rocha Rodrigues	Declaração financeira A - Pagamento de qualquer espécie e desde que economicamente apreciáveis, feitos a (i) você, (ii) ao seu cônjuge/ companheiro ou a qualquer outro membro que resida com você, (iii) a qualquer pessoa jurídica em que qualquer destes seja controlador, sócio, acionista ou participante, de forma direta ou indireta, recebimento por palestras, aulas, atuação como proctor de treinamentos, remunerações, honorários pagos por participações em conselhos consultivos, de investigadores, ou outros comitês, etc. Provenientes da indústria farmacêutica, de órteses, próteses, equipamentos e implantes, brasileiras ou estrangeiras: - Bayer / Pfizer: eliquis; Daichi-Sanchio: lixiana; Abbott: rivacrist; Bhoeringer-Ingelheim: jardiance; Libbs: Vatis.
Thiago Gonçalves Schroder e Souza	Nada a ser declarado
Veridiana Silva de Andrade	Nada a ser declarado

## Sumário

1. Recomendações Gerais 8

1.1. Sala Cirúrgica de DCEI 8

**1.1.1. Recursos Humanos** 8

**1.1.2. Recursos Materiais** 9

**1.1.2.1. Radioscopia** 9

**1.1.2.2. Monitorização** 9

**1.1.2.3. Materiais Cirúrgicos** 9

1.2. Clínica de Avaliação e Programação Eletrônica de DCEI 9

1.3. Avaliação Clínica antes do Implante de DCEI 9

1.4. Procedimento Cirúrgico e Tipos de DCEI 10

2. Recomendações para Implante de Marca-passo Convencional 10

2.1. Doença do Nó Sinusal 10

2.2. Bloqueios Atrioventriculares e Bloqueios Intraventriculares 12

**2.2.1. Bloqueios Atrioventriculares** 12

**2.2.2. Bloqueios Intraventriculares (BIV) com Condução Atrioventricular 1:1** 14

2.3. Síndrome da Hipersensibilidade do Seio Carotídeo 14

2.4. Síncope Vasovagal 16

2.5. Cardiomiopatia Hipertrófica 17

2.6. Doenças Neuromusculares 18

2.7. Síndrome da Apneia Obstrutiva do Sono 18

2.8. Síndrome do QT Longo Congênito (SQTLc) 19

2.9. Coração Transplantado 20

2.10. Escolha do Tipo de Marca-passo e do Modo de Estimulação 20

2.11. Estimulação Direta do Sistema Excito-condutor Cardíaco (Feixe de His, Ramo Esquerdo) 21

2.12. Estimulação sem Cabo-eletrodo (Leadless Pacemaker) 22

3. Recomendações para Implante de Marca-passo Multissítio/Terapia de Ressincronização Cardíaca (TRC) 24

3.1. Paciente em Ritmo Sinusal 24

3.2. Paciente com Fibrilação Atrial 25

3.3. *Upgrade* de Marca-passo Convencional 27

3.4. Na Indicação de Maca-passo Antibradicardia (1º Implante) 27

3.5. Na Indicação de Cardio-desfibrilador Implantável (TRC-D) 28

3.6. Estimulação Direta do Sistema Excito-condutor Cardíaco 28

**3.6.1. Modulação de Contratilidade Cardíaca** 29

4. Recomendações para Indicação de Cardioversor-desfibrilador Implantável 30

4.1. Prevenção Primária de Morte Súbita 30

**4.1.1. Miocardiopatia Isquêmica** 30

**4.1.2. Miocardiopatia Não Isquêmica** 31

**4.1.3. Cardiomiopatia Hipertrófica** 32

**4.1.4. Cardiomiopatia Chagásica** 34

**4.1.5. Cardiomiopatia Arritmogênica do Ventrículo Direito** 36

**4.1.6. Miocardiopatia Não Compactada** 38

**4.1.7. Síndrome do QT Longo e Síndrome do QT Curto Congênito** 39

**4.1.8. Síndrome de Brugada** 39

**4.1.9. Taquicardia Ventricular Polimórfica Catecolaminérgica (TVPC)** 40

**4.1.10. Taquicardia Ventricular Idiopática** 40

4.2. Prevenção Secundária de Morte Súbita 41

**4.2.1. Recuperados de Parada Cardíaca ou Taquicardia Ventricular Sustentada** 41

**4.2.1.1. Recuperados de Parada Cardíaca ou Taquicardia Ventricular Sustentada na Presença de Cardiopatia Estrutural** 41

**4.2.1.2. Recuperados de Parada Cardíaca ou Taquicardia Ventricular Sustentada na Ausência de Cardiopatia Estrutural** 42

**4.2.2. Síncope e Taquicardia/Fibrilação Ventricular no Estudo Eletrofisiológico** 42

4.3. Crianças, Adolescentes e Cardiopatia Congênita 43

4.4. Escolha do Tipo de CDI e Modo de Estimulação 46

**4.4.1. Técnica de Implante** 46

**4.4.2. Modo de Estimulação** 46

4.5. Custo-efetividade do CDI na Prevenção Primária e Secundária de Morte Súbita 46

**4.5.1. Prevenção Primária** 47

**4.5.2. Prevenção Secundária** 47

5. Recomendações para Monitor de Eventos (Loop Recorder) Implantável 48

6. Recomendações para Avaliação e Programação Eletrônica dos DCEI 49

6.1. Marca-passo Convencional 49

**6.1.1. Doença do Nó Sinusal** 49

**6.1.2. Bloqueio Atrioventricular** 50

**6.1.3. Fibrilação Atrial** 50

**6.1.4. Síncope Neuromediada e Síndrome do Seio Carotídeo** 50

6.2. Terapia de Ressincronização Cardíaca 50

6.3. Cardioversor-desfibrilador Implantável 52

6.4. Monitor de Eventos Implantável ( *Loop Recorder* ) 53

6.5. Monitoramento Remoto ( *Via Web* ) 53

7. Recomendações para Prevenção e Tratamento de Infecções e Explante de DCEI 54

7.1. Prevenção e Tratamento de Infecções 54

7.2. Remoção de Cabos-eletrodos de Dispositivos Cardíacos Eletrônicos Implantáveis 59

8. Recomendações para prevenção de interferências eletromagnéticas 64

8.1. Cirurgia com Uso de Eletrocautério 64

8.2. Ressonância Magnética 64

8.3. Radioterapia 66

9. Conclusão 66

Referências 68

## 1. Recomendações Gerais

Apesar da normatização e simplificação das técnicas de implante de dispositivos cardíacos eletrônicos implantáveis (DCEI), além do conhecimento médico e da experiência cirúrgica, são necessários local e materiais adequados. O conhecimento de eletrocardiografia, principalmente das arritmias cardíacas e dos princípios de eletrofisiologia cardíaca, é fundamental.

### 1.1. Sala Cirúrgica de DCEI

Os procedimentos cirúrgicos de estimulação cardíaca artificial são realizados por médicos cirurgiões cardiovasculares ou cardiologistas com área de atuação em estimulação cardíaca eletrônica implantável (SOBRAC/SBC, ABEC/SBCCV, AMB). Os procedimentos devem ser realizados em centro cirúrgico, laboratório de hemodinâmica ou de eletrofisiologia. A sala operatória deve ter dimensões, iluminação e ventilação adequadas, lavatório para a antissepsia e sistema de eletricidade bivolt (com aterramento que elimine interferências eletromagnéticas e proteção dos equipamentos).

#### 1.1.1. Recursos Humanos

Os profissionais envolvidos na realização de procedimentos cirúrgicos de DCEI são:

- Médicos com formação em estimulação cardíaca eletrônica implantável (ECEI), responsável e auxiliar
- Médico anestesiologista
- Instrumentador cirúrgico, preferencialmente com treinamento na área de ECEI
- Profissional de enfermagem, preferencialmente com treinamento na área de ECEI
- Técnico em ECEI
- Técnico de radiologia

#### 1.1.2. Recursos Materiais

##### 1.1.2.1. Radioscopia

Requisito ainda fundamental, a radioscopia (intensificador de imagem) pode ser fixa, como nos laboratórios de hemodinâmica, ou portátil (arco cirúrgico). A qualidade da imagem e os recursos de gravação e espelhamento de imagens facilitam bastante o procedimento, especialmente para terapia de ressincronização cardíaca (TRC). O intensificador de imagem deve possibilitar a visibilização de fios-guia de pequeno calibre e movimentos em diferentes projeções (oblíquas).

##### 1.1.2.2. Monitorização

A monitorização eletrocardiográfica deve ser contínua, com a possibilidade de armazenamento dos traçados. As derivações disponíveis devem permitir adequada avaliação intraoperatória de TRC e de estimulação fisiológica do sistema de condução cardíaco (feixe de His, ramo esquerdo, septal profunda); ademais, nesses casos, é fundamental a análise de eletrogramas intracavitários (polígrafo).

A monitorização não invasiva da pressão arterial e o oxímetro de pulso devem estar disponíveis.

##### 1.1.2.3. Materiais Cirúrgicos

- Caixa de instrumental cirúrgico apropriado
- Eletrocautério
- Cardioversor-desfibrilador externo
- Marca-passo (MP) externo temporário
- Estrutura de suporte avançado de vida
- Materiais e medicamentos para anestesia e estabilidade cardiovascular (analgésicos, anestésicos, antiarrítmicos, fármacos vasoativos, antibióticos etc.).
- Gerador de pulsos, cabos-eletrodos, introdutores, bainhas para cateterização de seio coronário e sistema de condução
- Programador e analisador específico do DCEI em uso ou a ser implantado.
- Ultrassonografia para acesso venoso pode ser útil para redução de complicações relacionadas à punção venosa profunda (p. ex., punção arterial acidental, pneumotórax)
- Ecocardiograma transesofágico: útil durante extração percutânea de cabos-eletrodos para diagnóstico precoce de tamponamento cardíaco

## 1.2. Clínica de Avaliação e Programação Eletrônica de DCEI

O médico responsável pela clínica de seguimento eletrônico de DCEI deve ter área de atuação em estimulação cardíaca eletrônica implantável (SOBRAC/SBC, ABEC/SBCCV, AMB). A clínica deve ter, em sua estrutura os seguintes recursos:

- Equipamento de eletrocardiograma (ECG)
- Programadores dos diversos fabricantes de DCEI
- Equipamento *nobreak*
- Cardioversor-desfibrilador externo com MP transcutâneo
- Imã
- Ecocardiograma transtorácico
- Acesso a exames complementares como teste ergométrico, *Holter* -24h, e exames de imagem (raio X, ressonância magnética, cintilografia miocárdica, tomografia computadorizada). O exame de teste de inclinação ( *Tilt Table Test* ) deve estar disponível na própria clínica de avaliação de DCEI ou em outra estrutura referenciada.
- Acesso a engenheiro especializado em ECEI

## 1.3. Avaliação Clínica antes do Implante de DCEI

A avaliação clínica inicial do paciente, antes da cirurgia de DCEI, deve incluir:

a) Anamnese e exame físico

A anamnese deve incluir investigação de sinais e sintomas relacionados às arritmias cardíacas, como síncope, pré-síncope, tonturas, palpitações, sinais e sintomas de insuficiência cardíaca (IC). História de morte súbita familiar, principalmente precoce e em parentes de primeiro grau, tem grande relevância.

O exame físico deve incluir inspeção, palpação dos pulsos periféricos, aferição da pressão arterial, ausculta cardíaca e de carótidas, frequência cardíaca e perfusão periférica.

Anticoagulantes orais e antiagregantes plaquetários devem ser suspensos temporariamente antes do procedimento cirúrgico, se possível.^1^ Outros fármacos, geralmente, não precisam ser suspensos preventivamente. Pacientes com sinais de infecção ativa não devem receber implantes de dispositivos até a resolução do quadro.

b) Exames complementares pré-operatórios

- ECG em repouso
- Radiografia de tórax (PA + perfil esquerdo)
- Exames laboratoriais: todos os pacientes devem realizar hemograma completo e de coagulação. Para procedimentos em que se usa contraste endovenoso, como na TRC ou obstrução venosa, é fundamental avaliação da função renal com dosagem de eletrólitos. Nos pacientes diabéticos, a glicemia de jejum deve ser avaliada. Os exames de urina tipo I e urocultura devem ser indicados aos pacientes com queixas urinárias.
- Outros exames como ecocardiograma, *Holter* -24h, estudo eletrofisiológico e ultrassom ou flebografia de membros superiores estão indicados somente quando a condição clínica justificar

Os pacientes devem permanecer em jejum absoluto por pelo menos 6 a 8 horas antes da cirurgia, dependendo da complexidade da cirurgia e do tipo de anestesia. Tricotomia, antissepsia local e antibioticoterapia profilática^2^ devem seguir protocolo institucional.

## 1.4. Procedimento Cirúrgico e Tipos de DCEI

**a) Procedimentos cirúrgicos**

Antes da cirurgia, as equipes médica e de enfermagem devem seguir os protocolos de cirurgia segura: confirmar nome do paciente, data de nascimento, número de registro hospitalar e lateralidade. Também devem confirmar a indicação do procedimento e checar os exames pré-operatórios.

O procedimento cirúrgico de DCEI deve ser realizado em centro cirúrgico ou laboratório de hemodinâmica/eletrofisiologia sob visão fluoroscópica, com monitorização eletrocardiográfica contínua, oximetria de pulso e registros intermitentes ou contínuos da pressão arterial. Dispositivos como os cardioversores-desfibriladores implantáveis (CDI) subcutâneos e os monitores de eventos implantáveis dispensam o uso do intensificador de imagem durante o implante.

A anestesia pode ser local, preferencialmente associada à sedação, ou geral. A escolha do tipo de anestesia depende da complexidade do procedimento, da via de acesso e das condições clínicas do paciente.

Para escolha do acesso cirúrgico, deve-se levar em consideração o local de implante do gerador de pulsos, o acesso ao coração para implante dos cabos-eletrodos (via transvenosa ou epicárdica) e a possibilidade de implante de sistemas sem cabo-eletrodo transvenoso ( *leadless pacemaker,* CDI subcutâneo). Outras variáveis que podem influenciar na escolha da estratégia cirúrgica são: uso de MP temporário e cateteres venosos, cirurgias torácicas prévias, necessidade de radioterapia (RT), características anatômicas, infecções de pele, membro superior dominante.

A região da bolsa do gerador de pulso geralmente é peitoral, podendo ser abdominal em situações específicas, em posição subcutânea ou submuscular. O acesso venoso é realizado por dissecção da veia cefálica ou punção de veia axilar, subclávia, jugular ou femoral. O número de cabos-eletrodos varia de acordo com o sistema implantado (geralmente de 1 a 3), sendo preferidos atualmente os cabos-eletrodos de fixação ativa ( *screw-in* ) em relação aos de fixação passiva, de acordo com a experiência profissional.

Durante o procedimento cirúrgico, é fundamental aferir os limiares de estimulação, de sensibilidade e as impedâncias dos cabos-eletrodos, além do eletrograma endocavitário ou epicárdico. Em caso de CDI, deve ser incluída medida de impedância de choque; a avaliação de limiar de desfibrilação é opcional para sistemas de CDI transvenosos (geralmente desnecessário), mas recomendável em implantes à direita, abdominal e CDI subcutâneo.^3 - 5^ Nos sistemas multissítios, com implante do cabo-eletrodo para estimulação do ventrículo esquerdo (VE) pelo seio coronário, são necessários acessórios específicos para essa abordagem como bainhas, cateter de eletrofisiologia e cateter para venografia para escolha da melhor veia tributária para implante do cabo-eletrodo.

O relatório de cirurgia de DCEI deve incluir identificação do paciente, descrição do ato operatório, dados técnicos do sistema e a ocorrência ou não de complicações (p. ex., pneumotórax, hemotórax, falha de captura e/ou sensibilidade, má conexão eletrodo-gerador, deslocamento do cabo-eletrodo, perfuração do ventrículo direito (VD), estimulação diafragmática, hematoma na bolsa do gerador, contaminação e arritmias). O registro brasileiro de marca-passos e cardioversores-desfibriladores (RBM) deve ser preenchido.

*Avaliação no pós-operatório e período de internação*

Após a cirurgia de DCEI, é necessário realizar avaliação clínica do paciente, avaliação eletrônica do sistema implantado e exames de ECG e radiografia de tórax para confirmar o funcionamento adequado do DCEI, a posição dos cabos-eletrodos e diagnosticar possíveis disfunções e complicações.

O paciente geralmente permanece internado em ambiente hospitalar por 12 a 24h. Pacientes submetidos a procedimentos sem necessidade de acesso intravascular (troca de gerador de pulsos ou implante de dispositivo subcutâneo) habitualmente permanecem em observação pós-operatória pelo período de 6 a 12 horas (hospital-dia).

**b) Tipos de DCEI**

O Quadro 1 resume os principais DCEI e suas características.

**Quadro 1:** – Principais tipos e características dos DCEI

Dispositivos	Características
Marca-passo (MP)	✓ Dispositivo cardíaco eletrônico multiprogramável, capaz de monitorar e promover a estimulação elétrica do coração, restaurar o sincronismo atrioventricular e a variabilidade da frequência cardíaca, detectar e registrar arritmias e tratar arritmias atriais usando mecanismos de *overpace* atrial ✓ Geralmente são compostos por unidade de gerador de pulsos; um circuito eletrônico e por cabos-eletrodos que interligam o gerador à interface do endocárdio; podem ser uni, bicamerais ✓ Recentemente introduzidos, os MP sem cabos-eletrodos ( *leadless pacemaker* ) são sistemas de estimulação elétrica, no qual todas as unidades do dispositivo são contidas em um único componente de implante intracardíaco realizado por sistema de bainhas transvenosas. ✓ As bradiarritmias são o foco da terapia com MP
Terapia de ressincroização cardíaca (TRC)	✓ Dispositivo cardíaco com função antibradicardia como os MP, mas que permitem a estimulação cardíaca multissítio, ou seja, estimulação do ventrículo direito e esquerdo (biventricular) para promover a correção da dissincronia intraventricular ✓ O tratamento adjuvante da insuficiência cardíaca avançada é o foco primordial da TRC
Cardioversor-desfibrilador implantável (CDI)	✓ Dispositivo cardíaco eletrônico que possui as funções do MP convencional, a capacidade de detectar, registrar e tratar automaticamente taquiarritmias ventriculares potencialmente fatais, por meio de terapia de choque (cardioversão ou desfibrilação) ou com estimulação ventricular programada ( *overdrive* – terapia antitaquicardia [do inglês, ATP]) ✓ As taquiarritmias ventriculares potencialmente letais associadas ou não às bradiarritmias podem ser tratadas com CDI
TRC + CDI	✓ São dispositivos que agregam em um único aparelho as funções da TRC e do CDI
Monitor de eventos implantável	✓ Dispositivo cardíaco de implante subcutâneo utilizado para monitoramento cardíaco prolongado (3 a 4 anos), com objetivo de detectar arritmias cardíacas intermitentes e esporádicas em casos de síncopes inexplicadas ou acidente vascular cerebral (AVC) criptogênico, por meio do registro contínuo da atividade elétrica do coração.

## 2. Recomendações para Implante de Marca-passo Convencional

### 2.1. Doença do Nó Sinusal

As disfunções do nó sinusal, quando resultam em sintomas, são denominadas doença do nó sinusal (DNS) e costumam ser a mais comum indicação para estimulação cardíaca artificial em âmbito global, correspondendo a aproximadamente metade dos implantes de MP definitivo.^6^

A DNS é caracterizada, do ponto de vista eletrocardiográfico, por uma ou mais das seguintes manifestações: bradicardia sinusal, pausa ou parada sinusal, bloqueio sinoatrial, taquiarritmias atriais (principalmente flutter ou fibrilação atrial [FA]) associadas a bradiarritmias (pausas sinusais): síndrome bradi-taqui e incompetência cronotrópica (resposta inadequada da frequência cardíaca ao exercício ou estresse).^7 - 9^

Os sintomas atribuíveis à DNS ocorrem devido à baixa frequência cardíaca ou de acordo com a duração da pausa dos batimentos cardíacos; os sintomas mais comuns são: palpitação, cansaço e dispneia, tontura, pré-síncope ou síncope. A síncope é um sintoma clínico comum, podendo estar presente em aproximadamente 50% dos pacientes encaminhados para implante de MP em decorrência de DNS.^10^

Embora possa ocorrer em qualquer idade, a DNS aumenta com a idade, afetando 1 em cada 600 pacientes com idade superior a 65 anos, não havendo preferência por sexo.^11 - 13^

A fisiopatologia da DNS é variada e geralmente envolve remodelamento eletrofisiológico e estrutural complexo.^14 , 15^Sua etiologia pode ser dividida em causas intrínsecas e extrínsecas.

As causas intrínsecas de DNS incluem processos inflamatórios, infecciosos e imunológicos, fibrose degenerativa, disfunção de canais iônicos e remodelamento do nó sinusal. A fibrose degenerativa idiopática, relacionada com a idade, é a causa intrínseca mais comum; no entanto, pesquisas recentes têm demonstrado que uma disfunção dos canais iônicos herdadas geneticamente também pode participar na gênese da disfunção sinusal resultante do envelhecimento.^16 - 19^Além disso, a resposta barorreflexa e a variabilidade da FC encontram-se diminuídas na população idosa.^20 , 21^

Outros mecanismos intrínsecos de DNS incluem: doenças infiltrativas (p. ex., hemocromatose, amiloidose), inflamatórias (p. ex., sarcoidose), IC e FA (mudanças estruturais e anatômicas ao longo da *crista terminalis* ) e coronariopatia crônica (acometimento da artéria do nó sinusal). Análises genômicas já identificaram *locus* nas proteínas que interagem com canais iônicos e canais relacionados às frequências cardíacas normais e anormais em repouso, fornecendo informações sobre os mecanismos que controlam a frequência cardíaca.^22 - 24^

Dentre as causas extrínsecas, agentes farmacológicos, distúrbios metabólicos e disfunção autonômica são os principais envolvidos na ocorrência de DNS. Os fármacos mais associados a DNS são os betabloqueadores, bloqueadores dos canais de cálcio, digitálicos, antiarrítmicos e medicações simpaticolíticas.^24^O lítio também pode estar associado à disfunção sinusal, muitas vezes definitiva.^25^

A disfunção autonômica, com manifestação cadioinibitória, pode mimetizar ou intensificar a DNS na síncope vasovagal e na hipersensibilidade do seio carotídeo.^26^

Outra causa rara de bradicardia recorrente e síncope é a epilepsia assistólica do lobo temporal. Nessa entidade, embora o implante de MP provisório possa ser fundamental na fase aguda, muitas vezes, o manejo específico (cirúrgico ou farmacológico) da epilepsia pode levar a regularização da disfunção sinusal e/ou bloqueio atrioventricular (BAV) sem a necessidade do implante de MP definitivo. As mesmas considerações são válidas para uma outra condição rara, a neuralgia do glossofaríngeo associada à assistolia e síncope.^27 , 28^

Anormalidades metabólicas, tais como acidose sistêmica grave, hipercalemia, hipocalemia ou hipocalcemia, podem causar bradicardia sinusal, incomum em situações agudas. Outros fatores extrínsecos possíveis são hipotireoidismo, hipóxia, hipotermia e toxinas. Também tem sido descrita a associação entre a síndrome de Brugada, um tipo de canalopatia, e a ocorrência de DNS.^29^

Os pacientes com DNS são geralmente assintomáticos nas fases iniciais no curso da doença, com os sintomas ocorrendo após vários anos de evolução. É também importante identificar quadros de bradicardia funcional assintomática, como ocorre durante o sono, em jovens saudáveis e em atletas, que não representam risco e não devem ser tratadas de forma geral.

A documentação da correlação das alterações eletrocardiográficas com as manifestações clínicas é essencial para o diagnóstico da DNS, seja por meio de ECG convencional de 12 derivações ou outros métodos, como *Holter* (24/48 horas, 7 dias), monitor de eventos externo ou monitor de eventos implantável.^30^ O estudo eletrofisiológico invasivo (EEF) não deve ser utilizado regularmente na prática clínica devido à ausência de dados conclusivos sobre a real indicação de MP definitivo nos pacientes que apresentem um tempo de recuperação do nó sinusal (TRNS) ou tempo de condução sinoatrial (TCSA) alterado.

Para definição de conduta e eventual indicação de implante de MP em pacientes com DNS, é fundamental a correlação entre os sintomas clínicos e a bradicardia, além da identificação de causas reversíveis.

Na ausência de causas reversíveis e na presença de sintomas, o implante de MP definitivo representa o tratamento de escolha para a DNS, embora não haja evidências de que a estimulação cardíaca artificial tenha impacto quanto à sobrevida ou ao risco de morte súbita cardíaca dos pacientes com DNS em relação à população em geral.^30^Contudo, o implante de MP melhora significativamente a qualidade de vida, pode reduzir o risco de FA e tromboembolismo sistêmico, permite o uso de fármacos antiarrítmicos que podem causar bradicardia e possibilita monitoramento contínuo do ritmo cardíaco.^31^ As recomendações para implante de MP definitivo na DNS encontram-se na Tabela 1 .

**Tabela 1:** – Recomendações para implante de marca-passo (MP) definitivo na doença do nó sinusal (DNS)

	Classe de recomendação	Nível de evidência
Doença do nó sinusal (bradicardia sinusal, pausa/parada sinusal, bloqueio sinoatrial ou síndrome bradi-taquicardia) espontânea, sem causa passível de tratamento ou induzida por fármacos necessários e insubstituíveis, com documentação de sintomas (síncope, pré-síncope, tontura ou cansaço/fadiga) relacionados à bradicardia	I	C
Doença do nó sinusal (bradicardia sinusal, pausa/parada sinusal, bloqueio sinoatrial ou síndrome bradi-taquicardia) espontânea, sem causa passível de tratamento ou induzida por fármacos necessários e insubstituíveis, com sintomas (síncope/pré-síncope/tontura ou cansaço/fadiga) não documentados após investigação preconizada (diagnóstico presuntivo) Síncope inexplicada com evidência de DNS no EEF	IIa	C
Síncope inexplicada, quando houver documentação de pausa sinusal >6s assintomática	IIb	C
Paciente assintomático, com sintoma documentado e claramente não relacionado à bradicardia; paciente sintomático com bradicardia decorrente de causas reversíveis, incluindo-se fármacos não essenciais	III	C

*DNS: doença do nó sinusal; EEF: estudo eletrofisiológico.*

Quanto ao modo de estimulação, os grandes estudos randomizados não evidenciaram melhora da sobrevida com a estimulação atrial (AAI) ou atrioventricular (DDD) em relação à ventricular (VVI); entretanto, houve benefícios, como redução de taxas de FA, incidência de síncopes e síndrome do MP.^32 - 35^ Em uma revisão sistemática dos grandes estudos randomizados, houve significativa redução de acidente vascular cerebral (AVC) ( *hazard ratio* [HR]:0,81) e FA (HR:0,80) com a estimulação AAI e/ou DDD comparada com a estimulação VVI.^36^O impacto do modo de estimulação na prevenção de IC ou AVC e a melhora na qualidade de vida são menos evidentes.

Os resultados do estudo DANPACE,^10^ que analisou a estimulação AAI *versus* DDD em pacientes com DNS, mostraram que a estimulação AAIR associou-se a maior *incidência* de FA paroxística (HR:1,24) e o dobro de reoperações quando comparada com a estimulação DDDR. As reoperações ocorreram principalmente pela necessidade de *upgrade* de AAIR para DDDR, em decorrência do desenvolvimento de BAV durante o acompanhamento. Outro aspecto relevante observado nesse estudo foi que o benefício da estimulação AAI pode ser atenuado em pacientes com longos intervalos PR que podem resultar em insuficiência mitral diastólica.

A estimulação preferencial atrial (AAIR ou DDDR) é o modo predileto em pacientes com DNS ( Tabela 2 ).

**Tabela 2:** – Recomendações para escolha do modo de estimulação na doença do nó sinusal (DNS)

	Classe de recomendação	Nível de evidência
1. AAI(R); na presença de condução AV normal 2. DDD(R), na presença de BAV avançado	I	A
1. AAI(R) com reversão automática para DDD(R), na presença de BAV avançado intermitente	I	B
1. DDD com algoritmo para minimizar a estimulação ventricular em pacientes com condução AV preservada	IIa	B
1. VVI(R) em idosos, na ausência de condução retrógrada VA	IIb	B
1. VVI(R) em pacientes com possibilidade de demandar apenas curtos períodos de estimulação ventricular ou portadores de comorbidades significativas com impacto na sobrevida ou eventos clínicos	IIb	C
1. VVI(R) na presença de condução retrógrada VA; VDD(R) 2. AAI(R); na presença de BAV avançado	III	C

*AV: atrioventricular; BAV: bloqueio atrioventricular.*

A maioria dos pacientes com DNS apresenta condução atrioventricular preservada. Por outro lado, sabe-se que a estimulação do VD tem sido associada a consequências fisiológicas negativas como resultado da dissincronia ventricular: remodelamento e redução da fração de ejeção do VE (FEVE) e insuficiência mitral funcional.^37^ Em decorrência disso, em pacientes sem BAV associado, utiliza-se a programação de algoritmos que buscam diminuir a estimulação ventricular desnecessária, como a histerese atrioventricular e a mudança automática do modo DDD para AAI.^38^

Algoritmos para suprimir a ocorrência de FA, como estimulação atrial contínua ( *overpace* atrial) ou estimulação atrial desencadeada pela sensibilidade de atividade atrial intrinseca, isoladamente ou em combinação, não têm seus beneficios comprovados. Da mesma forma, sítios alternativos de estimulação – tais como estimulação do fascículo de Bachmann, estimulação biatrial ou multissítio atrial – falharam em mostrar efeitos consistentes.^39^

Os dispositivos atuais são dotados de um ou mais mecanismos de sensores de resposta de frequência, geralmente com base em movimento corporal (cristais piezoelétricos ou acelerômetros) ou ventilação/volume minuto. O objetivo principal dos sensores é aumentar a frequência cardíaca de maneira fisiológica e não necessariamente alterar os resultados clínicos. Embora o dispositivo não consiga avaliar com precisão a resposta cronotrópica atrial, ele pode fornecer indicadores da progressão da doença atrial através dos histogramas de frequência e arritmia, porcentagem de estimulação atrial e atividade diária do paciente. Tais dados podem ser úteis para a programação do sensor. Não existem evidências de que a utilização de uma combinação de sensores (p. ex., acelerômetro e ventilação minuto) possa proporcionar melhora da qualidade de vida.

### 2.2. Bloqueios Atrioventriculares e Bloqueios Intraventriculares

#### 2.2.1. Bloqueios Atrioventriculares

O estímulo elétrico originado no nó sinusal é propagado pelo miocárdio pelo sistema de condução especializado. O retardo ou a falha na propagação do estímulo entre os átrios e os ventrículos caracterizam os BAV. Essa alteração da propagação do estímulo pode corresponder a uma alteração patológica ou ser um fenômeno funcional decorrente da refratariedade fisiológica (propriedade intrínseca das células do sistema de condução).^40^

Do ponto de vista eletrocardiográfico, os BAV são classificados em 1º grau, 2º grau (Mobitz I, Mobitz II, 2:1, avançado) e 3º grau.

O BAV de 1º grau corresponde ao retardo na condução do estímulo do átrio ao ventrículo com um intervalo PR > 200ms.

No BAV de 2º grau tipo I ( *Mobitz* I), o bloqueio ocorre após prolongamento gradual dos intervalos PR (fenômeno de *Wenckebach* ), com uma onda P bloqueada ao final. No BAV de 2º grau tipo II ( *Mobitz* II), a onda P é subitamente bloqueada, ou seja, sem o alargamento progressivo do intervalo PR. Quando a condução AV ocorre com um padrão 2:1, o bloqueio de segundo grau habitualmente não pode ser classificado inequivocamente como tipo I ou tipo II sem o auxílio de manobras autonômicas, fármacos ou mesmo o estudo eletrofisiológico invasivo. O BAV avançado refere-se ao bloqueio de duas ou mais ondas P consecutivas com alguns batimentos conduzidos, o que indica alguma preservação da condução AV. Na presença de FA com pausas significativas (> 5s), deve ser considerada a possibilidade de BAV de alto grau.

Por fim, o BAV de 3º grau (bloqueio atrioventricular total [BAVT]) é definido como ausência de condução AV (completa dissociação entre ondas P e QRS, com frequência atrial superior à frequência ventricular).^41^

Existem inúmeras patologias, congênitas ou mais frequentemente adquiridas, que podem afetar a condução AV. As causas degenerativas são as mais comuns na prática clínica e estão associadas ao aumento da idade, hipertensão arterial sistêmica e diabetes melito. Entre as causas infecciosas, em nosso meio, destacam-se a miocardite crônica da doença de Chagas e, em menor proporção, as miocardites agudas virais, que podem ocasionar bloqueios intermitentes agudos e definitivos.

O BAV atribuível à isquemia da parede inferior ou ao infarto agudo do miocárdio pode ser reversível, assim como os bloqueios mediados pelo sistema nervoso autônomo.

Causas iatrogênicas, principalmente por ação farmacológica, também devem ser lembradas na dependência da situação clínica.

O BAV pode ser classificado anatomicamente, de acordo com o sítio do bloqueio, em nodal AV, intra-His e infra-His. O BAV nodal está associado a progressão mais lenta, escape juncional mais rápido e confiável e melhor resposta à manipulação autonômica como administração de atropina, isoproterenol e epinefrina. Em contraste, os BAV intra-His ou infra-His progridem mais rapidamente e estão associados a escape ventricular mais lento e imprevisível, QRS mais largos e que respondem mal à atividade adrenérgica ou bloqueio vagal. Os bloqueios de alto grau (avançados ou 3º grau) apresentam maior risco de baixo débito e assistolias graves, o que implica necessidade de terapêutica urgente. Na presença de FA, identifica-se BAVT quando a resposta ventricular está baixa (< 50bpm) com intervalo RR regular.

O BAV de 1º grau geralmente é assintomático, mas pode resultar em fadiga ou intolerância ao esforço se o intervalo PR for longo o suficiente para permitir a perda do sincronismo AV. Essa alteração, “pseudossíndrome do MP”, pode ocorrer com intervalo PR > 300ms.^42^ Da mesma forma, o BAV de 2º grau tipo I é frequentemente assintomático e observado em pacientes ativos e saudáveis com ou sem histórico de doença cardíaca, principalmente durante atividade parassimpática. No entanto, se ocorrer com frequência ou durante o exercício, pode causar sintomas de intolerância ao esforço ou tontura.^43^

Em 61% dos pacientes com síncopes e bloqueio de ramo subjacente ou bloqueio bifascicular, podem estar presentes anormalidades da condução no sistema His-Purkinje significativas e clinicamente relevantes, identificados no EEF.^44^

Em pacientes com BAV, a avaliação clínica pode ajudar a definir causas transitórias ou reversíveis, e o tratamento ou resolução pode tornar desnecessária a estimulação artificial permanente.

No BAVT congênito, a indicação de MP definitivo é mandatória na presença de sintomas ou quando a criança apresenta frequência cardíaca de repouso < 55 bpm ou < 70 bpm, se associada à doença cardíaca estrutural.^45^ Nos casos assintomáticos, o acompanhamento regular com exames complementares é necessário para avaliar a FC média, o intervalo QT, pausas, arritmia ventricular, distúrbio de condução intraventricular (DCIV), presença ou surgimento de doença cardíaca estrutural, baixo desenvolvimento cognitivo-pondero-estatura e intolerância ao exercício físico, a fim de verificar a necessidade de MP definitivo.^46^ Também recomenda-se implante de MP profilático, ou CDI, em alguns pacientes assintomáticos com disfunções neuromusculares e outras doenças genéticas.

Em pacientes com bradicardia indicativa de implante de MP e disfunção ventricular esquerda, o implante de CDI deve ser considerado (ver item 4).

O implante de MP não é indicado em pacientes assintomáticos com FA permanente com frequência cardíaca baixa em repouso, que apresentam resposta cronotrópica apropriada na vigília, independentemente da ocorrência e duração das pausas. Já nos casos com pausas significativas (> 3s) sintomáticas ou atribuíveis a bloqueio infranodal, indica-se MP.^30^

O implante de MP para suporte terapêutico, especialmente em pacientes com IC ou doença coronária, pode ser necessário, especialmente frente à necessidade de uso crônico de betabloqueadores.^47^

Para se determinar o melhor tipo de dispositivo e modo de estimulação artificial para pacientes com BAV, duas variáveis clínicas devem ser consideradas: porcentagem de estimulação ventricular esperada e função sistólica do VE (FEVE).

Estudos que compararam a estimulação com preservação da ativação atrioventricular sequencial com a estimulação unicameral ventricular em pacientes com BAV (PASE, MOST, CTOPP) não demonstraram redução significativa da mortalidade ou da taxa de AVC.^34 , 35 , 48^

Por outro lado, em uma revisão sistemática, os autores identificaram que a estimulação bicameral seria a mais recomendada por diminuir a incidência de FA e reduzir a prevalência de síndrome do MP quando comparado à estimulação unicameral ventricular (VVI).^49^ Já no estudo UKPace (pacientes ≥ 75 anos), não ficou demonstrado benefício da estimulação AV em termos de mortalidade, incidência de FA ou IC quando comparada à estimulação ventricular. Também observou taxas similares de AVC e maior taxa de complicações relacionadas ao procedimento cirúrgico nos pacientes submetidos ao implante de MP bicameral (7,8% *x* 3,5%; p < 0,001).^50^ Assim, é razoável indicar implante de MP unicameral (VVI) em pacientes com fragilidade ou comorbidades significativas, idade avançada, estilo de vida muito sedentário ou pouca necessidade diária de estimulação nessa população de pacientes.

Em pacientes que apresentam condução retrógrada VA, a estimulação ventricular pode ocasionar sintomas da “síndrome do MP”. Nesses casos, deve-se preferir estimulação bicameral para evitar a dissincronia atrioventricular.^51^

Os efeitos deletérios da estimulação crônica do VD foram demonstrados em vários estudos, embora apenas uma minoria (5% a 9%) de indivíduos com estimulação crônica do VD desenvolva disfunção ventricular grave com sintomas de IC.^52 - 54^Nesse sentido, pacientes com disfunção de VE e indicação de MP devido a BAV foram avaliados nos estudos COMBAT^55^ (FEVE < 35%) e BLOCK-HF^56^ (FEVE ≤ 50%). Nesses estudos, que compararam a TRC *versus* a estimulação convencional do VD, demostrou-se melhora clínica (NYHA) e remodelamento reverso do VE (com aumento da FEVE) com a TRC, com redução significativa de desfechos primários.

Em pacientes com FA e disfunção de VE, submetidos à ablação do nó AV para controle de FC, a estimulação com TRC ou do sistema excito-condutor cardíaco (feixe de His ou ramo esquerdo) parece estar associada a melhores resultados em comparação com a estimulação convencional do VD.^30^

As recomendações para indicação de MP nos BAV estão sumarizadas na Tabela 3 .

**Tabela 3:** – Recomendações para implante de marca-passo (MP) definitivo no bloqueio atrioventricular (BAV)

	Classe de recomendação	Nível de evidência
BAV adquirido, de 2° grau Mobitz II, grau avançado ou de 3° grau não atribuíveis à causa reversível ou fisiológica, independentemente da ocorrência de sintomas BAV de 2º grau Mobitz II, avançado, de 3º grau ou bloqueio de ramo alternante, mesmo assintomático, e persistente após pelo menos 72 horas de IAM BAV avançado ou bloqueio de ramo alternante após TAVI, persistente por pelo menos 24 a 48 horas BAV avançado após IAM, persistente por pelo menos 5 dias BAV avançado sintomático, persistente após pelo menos 5 dias de cirurgia cardíaca valvar, revascularização ou cirurgia de fibrilação atrial BAV de 3º grau congênito sintomático BAV de 3º grau congênito assintomático associado a fatores de risco (pausa superior a 3 vezes o ciclo RR basal, QRS largo, QTc prolongado, arritmia ventricular complexa, FC média <50bpm, disfunção ventricular)	I	B
BAV adquirido de 2º grau Mobitz I sintomático não atribuível à causa reversível ou fármaco não essencial BAV de 2º grau Mobitz II, avançado ou de 3º grau, assintomático e persistente após pelo menos 5 dias de cirurgia cardíaca valvar, revascularização ou cirurgia de fibrilação atrial FA permanente com baixa resposta ventricular com sintomas atribuídos à bradicardia	I	C
BAV de 3º grau congênito em adultos (>18 anos) assintomáticos	IIa	B
Em pacientes sintomáticos, claramente em decorrência de BAV de 1º grau significativo (pseudossíndrome do MP)	IIa	C

*BAV: bloqueio atrioventricular; FA: fibrilação atrial; IAM: infarto agudo do miocárdio; MP: marca-passo; QTc: intervalo QT corrigido; TAVI: implante percutâneo transcateter de válvula aórtica.*

#### 2.2.2. Bloqueios Intraventriculares (BIV) com Condução Atrioventricular 1:1

As anormalidades do complexo QRS, representadas pelos bloqueios fasciculares ou bloqueios de ramo, são causadas por atraso da condução ou bloqueio de um ou mais ramos do sistema His-Purkinje.

O atraso de condução ou bloqueio do ramo direito associado a boqueio de um dos fascículos do ramo esquerdo é denominado bloqueio bifascicular (a mesma terminologia cabe no caso do bloqueio de ramo esquerdo [BRE]). Condições clínicas que podem ocasionar BIV incluem: genético-hereditárias, inflamatórias, infecciosas, infiltrativas, metabólicas, isquêmicas e degenerativas.

A presença isolada de BIV são raramente associados a sintomas, mas pode ser marcador de doença cardíaca estrutural; a presença ou surgimento de BRE pode causar dissincronia cardíaca e disfunção progressiva do VE. Alguns estudos demonstram correlação do BRE com doença coronariana e IC.^57 , 58^A progressão do BRE ou bloqueio bifascicular para BAV avançado é baixa, cerca de 1%/ano.^59^ A presença de BRE costuma estar associada a maior mortalidade que os demais distúrbios da condução intraventricular.^60^ O implante de MP é recomendado em pacientes com BIV em algumas doenças neuromusculares devido à alta incidência de BAVT e morte súbita cardíaca.

O EEF pode identificar distúrbios da condução de alto risco; no entanto, é um procedimento com sensibilidade variável e não é isento de riscos. Em pacientes com síncope, a presença de BIV é preditor de anormalidades ao EEF.^61^

O bloqueio de ramo alternante, independentemente de sintomas, quando a morfologia do complexo QRS alterna espontaneamente entre BRE e BRD, também é indicativo de implante de MP, pois denota doença do sistema de condução em nível infranodal, com alto risco de BAVT grave.

As recomendações para indicação de MP nos BIV estão sumarizadas na Tabela 4 .

**Tabela 4:** – Recomendações para implante de marca-passo (MP) definitivo no bloqueio intraventricular (BIV)

	Classe de recomendação	Nível de evidência
Em pacientes com síncope e bloqueio de ramo com registro de intervalo HV ≥ 70ms ou bloqueio infranodal no EEF, sem registro de TV hemodinamicamente instável, está recomendado o implante de MP definitivo Em pacientes com bloqueio de ramo alternante, independentemente de sintomas, está recomendado o implante de MP definitivo BRE novo persistente com QRS > 150ms por mais de 48 horas após TAVI, na presença de PRi > 240ms	I	C
Em pacientes com bloqueio bifascicular e síncope inexplicada, sem a realização de estudo eletrofisiológico (idosos, pacientes frágeis e síncope recorrente)	IIb	B
BRE novo persistente com QRS > 150ms por mais de 48 horas após TAVI, na presença de PRi normal	IIb	C
Pacientes assintomáticos com distúrbio da condução intraventricular isolado e condução AV 1:1 na ausência de outras indicações para implante de MP	III	B

*BIV: bloqueio intraventricular; BRE: bloqueio de ramo esquerdo; EEF: estudo eletrofisiológico; MP: marca-passo; PRi: intervalo PR; TAVI: implante percutâneo transcateter de válvula aórtica.*

## 2.3. Síndrome da Hipersensibilidade do Seio Carotídeo

A síndrome da hipersensibilidade do seio carotídeo (SHSC) é caracterizada pela história de síncope associada à resposta reflexa exacerbada decorrente da estimulação mecânica do seio carotídeo, espontânea ou por massagem (MSC).^62^ A síncope é consequente à bradicardia e/ou hipotensão arterial significativas, deflagradas por movimentos da cabeça ou situações que ocasionam compressão involuntária do pescoço e seio carotídeo, embora esta correlação não seja clinicamente evidente em muitos pacientes.^63^

O diagnóstico de SHSC é feito quando, na ausência de fármacos depressores do sistema excito-condutor, ocorre pausa > 3s (parada sinusal ou BAV) e/ou queda da pressão arterial sistólica (PAS) ≥ 50mmHg, com reprodução da síncope, durante manobra de compressão sequencial dos seios carotídeos direito e esquerdo, por 5 a 10s, realizada na posição supina e inclinada (teste de inclinação), em pacientes com mais de 40 anos de idade.^64^ A reprodução da síncope durante a MSC aumenta a especificidade do teste diagnóstico, e a compressão carotídea na posição inclinada aumenta sua sensibilidade.^63^

As respostas reflexas na SHSC podem ser classificadas, quanto ao perfil hemodinâmico, em três tipos: cardioinibidora (pausa ventricular > 3s), mista (pausa ventricular > 3s associada à queda da PA sistólica ≥ 50mmHg) ou vasodepressora (queda isolada da PAS ≥ 50mmHg). A incidência de SHSC aumenta com a idade (< 50 anos: ~0%, 50 a 59 anos: 2,4%, 60 a 69 anos: 9,1%, 70 a 79 anos: 20% e > 80 anos: 40%).^63^

As evidências que suportam o implante de MP definitivo na SHSC são baseadas em pequenos estudos controlados e estudos observacionais retrospectivos.^65 - 68^

Em uma revisão de 12 estudos, com 601 pacientes tratados com MP e 305 controles, apesar da heterogeneidade quanto à seleção dos pacientes, posição durante a MSC (horizontal ou inclinada), tempo de seguimento e modo de estimulação, observaram-se taxas menores de recorrência de síncope nos pacientes tratados (0% a 20%) quando comparados aos controles (20% a 60%).^68^ A metanálise de três estudos controlados, com seguimento médio de 3,3 anos, demonstrou redução significativa (76%) na taxa de recorrência de síncope nos pacientes tratados com MP *versus* o grupo controle (9% e 38%, respectivamente; RR 0,24; IC 95% 0,12-0,48).^68^

O estudo SAFE PACE,^69^ que avaliou 175 pacientes idosos com quedas recorrentes inexplicadas, aparentemente sem perda de consciência e resposta cardioinibitória durante compressão do seio carotídeo, sugere que o diagnóstico de SHSC deve ser considerado nesses casos. No grupo randomizado para implante de MP definitivo, observou-se redução significativa na taxa de eventos (síncopes 53%, quedas 70% e traumatismos físicos 70%) durante o seguimento.

Em um estudo no qual o diagnóstico de SHSC foi complementado com o registro de pausas espontâneas por meio de monitores de eventos implantados, ocorreu redução de 98% na carga de síncope após o implante de MP (1,68 episódio por paciente/ano, IC 95% 1,66-1,70; *vs.* 0,04 episódio por paciente/ano, IC 95% 0,038-0,042).^70^ Na investigação de síncopes inexplicadas em pacientes com mais de 40 anos, os monitores de eventos implantáveis podem ser úteis no diagnóstico da SHSC, assim como na síncope vasovagal, através do registro de pausas espontâneas, quando a investigação inicial através de MSC e teste de inclinação é negativa.^71^As recomendações para implante de MP na SHSC estão listadas na Tabela 5 .

**Tabela 5:** – Recomendações para implante de marca-passo (MP) definitivo na síndrome da hipersensibilidade do seio carotídeo (SHSC) e síncope vasovagal

	Classe de recomendação	Nível de evidência
Síncope recorrente, > 40 anos de idade e documentação de pausa sintomática espontânea >3s (pausa sinusal e/ou BAV) ou pausa >6s assintomática	I	A
Síncope recorrente, > 40 anos de idade e manobra de massagem do seio carotídeo com resposta cardioinibidora (pausa >3s, pausa sinusal e/ou BAV) ou mista (pausa > 3s + hipotensão) na ausência de fármaco depressor do sistema excito-condutor	IIa	B
Síncope recorrente, > 40 anos de idade e pausa sintomática > 3s (pausa sinusal e/ou BAV) induzida no teste de inclinação Queda recorrente, inexplicada, sem pródromos, > 40 anos de idade e manobra de massagem do seio carotídeo com resposta cardioinibidora (pausa > 3s, pausa sinusal e/ou BAV)	IIb	B
Paciente com síncope e ausência de resposta cardioinibidora documentada	III	B
Paciente assintomático(a) e massagem do seio carotídeo com resposta cardioinibidora	III	C

*BAV: bloqueio atrioventricular; SHSC: síndrome da hipersensibilidade do seio carotídeo.*

É importante salientar que pacientes idosos assintomáticos podem apresentar hipersensibilidade de seio carotídeo, porém sem caracterização da SHSC e sem indicação para implante de MP. Por outro lado, esses pacientes que apresentam quedas recorrentes inexplicadas e resposta cardioinibidora durante a compressão do seio carotídeo (hipersensibilidade de seio carotídeo), podem se beneficiar do MP definitivo.^69^

Com relação ao modo de estimulação na SHSC, em um estudo que avaliou as alterações hemodinâmicas agudas em pacientes com SHSC submetidos à MSC, evidenciou-se que o modo VVI (câmara-única) esteve associado a maior queda da PAS e maior taxa de persistência dos sintomas do que a estimulação em modo DVI (dupla-câmara).^72^ Estudos subsequentes comparando o modo de estimulação unicameral com o bicameral no longo prazo evidenciaram tendência de menores taxas de recorrência de síncope e pré-síncope em pacientes com estimulação dupla-câmara^73 - 76^( Tabela 6 ).

**Tabela 6:** – Recomendações para modo de estimulação na síndrome da hipersensibilidade do seio carotídeo (SHSC) e síncope vasovagal

	Classe de recomendação	Nível de evidência
MP bicameral (DDD,O/R) na SHSC e na síncope vasovagal	IIa	B

*MP: marca-passo; SHSC: síndrome da hipersensibilidade do seio carotídeo.*

## 2.4. Síncope Vasovagal

A síncope vasovagal é caracterizada pela história de perda da consciência associada a reflexo neuromediado exacerbado que cursa com redução súbita do fluxo sanguíneo cerebral secundária à vasodilatação e/ou redução da frequência cardíaca. Na maioria dos casos, a síncope é secundária à queda súbita e significativa da PA, acompanhada de graus variáveis de bradicardia e geralmente precedida de manifestações prodrômicas, tais como: mal-estar, sudorese, sensação de calor, palidez e tontura, e seguida de fadiga. A síncope vasovagal, frequentemente, é desencadeada por um gatilho, como um estresse emocional importante, medo ou dor, e representa a principal causa de síncope, principalmente em indivíduos jovens. São fatores predisponentes: ortostatismo prolongado, ambientes fechados ou quentes, punção venosa, traumatismo físico e outros.

De acordo com alterações observadas na PA e na FC, classifica-se a resposta vasovagal em três tipos: tipo 1 ou resposta mista (queda significativa da PA acompanhada de diminuição discreta da FC); tipo 2 ou resposta cardioinibidora (diminuição importante da FC < 40 bpm ou assistolia > 3s); e tipo 3 ou resposta vasodepressora (queda significativa da PA sem diminuição significativa da FC).^77^

Apesar de, eventualmente, estar relacionada a traumatismos físicos e inaptidão para realizar atividades de risco pessoal ou coletivo, a síncope vasovagal apresenta prognóstico benigno a longo prazo, e o tratamento, na maioria das vezes, é não farmacológico, por meio de orientações e mudanças de hábitos de vida. Entretanto, cerca de 14% dos pacientes apresentam formas severas de síncope vasovagal e necessitam de tratamento adicional (síncopes recorrentes muito frequentes, com comprometimento da qualidade de vida, com pródromos curtos ou ausentes e maior risco de traumatismos, ou associadas a atividades de alto risco (p. ex., motorista profissional, operador de máquinas, piloto de avião, esportista competitivo etc.). A idade do paciente é o fator mais importante na escolha da terapia mais apropriada.

Existem poucas opções terapêuticas baseadas em evidências na síncope vasovagal. A estimulação cardíaca artificial pode ser efetiva em pacientes com síncope vasovagal e reflexo cardioinibidor dominante; portanto, o foco da investigação clínica deve ser a documentação da correlação entre a síncope e a bradicardia.

A efetividade do MP definitivo foi avaliada em alguns estudos randomizados. O estudo VPS-I, randomizado aberto, avaliou pacientes com síncopes recorrentes (seis ou mais) e teste de inclinação positivo (bradicardia < 60bpm ou pausa > 1s) randomizados para tratamento com MP (DDD *rate-drop response* ) ou tratamento clínico.^78^ A recorrência de síncope após 12 meses de seguimento foi 22% (6/27) no grupo MP e 70% (19/27) no grupo de tratamento clínico (RR 85,4%; 95% IC 59,7%-94,7%; 2p = 0,00002). O estudo SYDIT, randomizado aberto, incluiu pacientes com síncopes recorrentes (três ou mais) e teste de inclinação positivo (bradicardia) para tratamento com MP (DDD *rate-drop response* ) ou tratamento clínico (atenolol 100mg/dia).^79^ A recorrência de síncope no grupo MP foi 4,3% (2/46), e no grupo atenolol foi 25,5% (12/47), após seguimento médio de 135 dias (OR 0,133; 95% IC 0,028-0,632; p = 0,004). No estudo VASIS, randomizado aberto, os pacientes foram randomizados para MP (DDI histerese de FC) ou nenhum tratamento.^80^ A recorrência de síncope, após seguimento médio de 3,7 anos, foi 5% (1/19) no grupo MP e 61% (14/23) no grupo controle (p = 0,0006). No estudo VPS II, randomizado cego, foram selecionados pacientes com síncopes recorrentes (seis ou mais), porém bradicardia significativa no teste de inclinação não foi critério de inclusão.^81^ Todos os pacientes foram submetidos a implante de MP, programados em “ativado/ *ON* ” ou “desativado/ *OFF* ”. A taxa de recorrência de síncope foi 33% (16/48) no grupo MP bicameral ( *rate-drop response* ) ativado e 42% (22/52) no grupo MP desativado (modo ODO), sem redução significativa no risco de síncope (RR 30%; 95% IC -33% a 63%; p = 0,14). No estudo randomizado duplo-cego mais importante publicado, o ISSUE-3, pacientes com mais de 40 anos de idade e documentação da síncope espontânea por meio de monitor de eventos implantável associada à assistolia >3s, ou assistolia > 6s na ausência de síncope, foram randomizados para MP bicameral ( *rate drop response* ) “ativado/ *ON* ” ou “desativado/ *OFF* ”.^82^ Durante seguimento médio de 2 anos, ocorreu redução significativa (57%) na taxa de recorrência de síncope (25% no grupo “ *ON* ” e 57% no grupo “ *OFF* ”, p = 0,039). Nos principais estudos, utilizou-se MP bicameral com o modo *rate drop response* .

Em um pequeno estudo retrospectivo, o MP bicameral com o modo *closed-loop stimulation* (CLS) ativado, foi comparado com o modo desativado, com demonstração de menores taxas de recorrência de síncope.^83^

As recomendações para implante de MP definitivo na síncope vasovagal estão resumidas na Tabela 5 , e as recomendações para escolha do modo de estimulação, na Tabela 6 .

## 2.5. Cardiomiopatia Hipertrófica

A cardiomiopatia hipertrófica (CMH) é uma doença cardiovascular genética comum, caracterizada por hipertrofia ventricular esquerda, na ausência de outras alterações cardíacas ou doenças sistêmicas capazes de produzir a magnitude de hipertrofia ventricular encontrada em seus portadores.^84^ Em sua forma obstrutiva, existe um gradiente de pressão no trato de saída do VE, sendo que gradientes maiores estão associados a sintomas mais graves e aumento de mortalidade.^85^

Em paciente com sintomas causados por obstrução da via de saída do VE (VSVE), as opções terapêuticas incluem fármacos com efeitos inotrópicos negativos, miectomia septal cirúrgica, ablação alcoólica septal e transplante cardíaco.^86^

Em pacientes com obstrução da VSVE, a estimulação da ponta do VD promove alterações no padrão de contração ventricular e cria uma dissincronia regional, que tem como resultado a ativação tardia da parte basal do septo e a redução da contratilidade do VE, que, por sua vez, acarreta na redução do movimento sistólico anterior da valva mitral e reduz o gradiente pressórico na VSVE.^87 , 88^ Entretanto, a dissincronia ventricular ocasionada pela ativação com QRS largo, por si só, reduz a contratilidade do VE e pode levar à redução do gradiente no trato de saída. Assim, o benefício, neste caso, estaria relacionado a um efeito colateral do MP.

A redução nos gradientes da VSVE com estimulação ventricular foi demonstrada em três pequenos estudos randomizados e controlados e em diversos estudos observacionais; contudo, a melhora dos sintomas e a qualidade de vida apresentaram resultados variáveis.^89 - 93^ Em um estudo, a análise de subgrupos sugere que pacientes com mais de 65 anos de idade apresentam maior chance de benefício.^94^

Em uma revisão da base de dados da Cochrane, os autores concluíram que os dados de benefício da estimulação ventricular na CMH são baseados em medidas de gradientes, sem evidências em relação a desfechos clínicos relevantes.^95^ Além disso, em geral, a magnitude de redução dos gradientes é menor quando comparada com a miectomia ou a ablação septal. Desse modo, a indicação de MP dupla-câmara unicamente para reduzir o gradiente na VSVE está restrita a condições muito específicas: pacientes com mais de 65 anos de idade, com hipertrofia moderada, com sintomas definidos devido à obstrução da VSVE e que não tenham indicação de CDI.^93^

Em geral, pacientes com CMH obstrutiva, com sintomas refratários ao tratamento farmacológico, devem ser considerados para miectomia ou ablação septal como primeira escolha. Casos muito graves poderão precisar de transplante cardíaco.

Nos pacientes submetidos a implante de MP para redução do gradiente da VSVE, a programação de intervalo AV curto é crucial (intervalo AV em VAT = 100 ± 30ms), para obter pré-excitação máxima do VD sem comprometer o enchimento ventricular diastólico.^96^ Adicionalmente, deve-se programar a frequência máxima de seguimento atrial maior que a frequência máxima apresentada pelo paciente durante teste de esforço. Geralmente, pacientes com CMH, que toleram muito mal frequência cardíaca elevada, utilizam betabloqueadores – o que resulta em frequência máxima menor durante o esforço; por outro lado, são pacientes suscetíveis ao desenvolvimento de FA. Assim, deve-se programar a reversão automática de modo ( *automatic mode switch* [mas]) para DDI(R), evitando-se estimulação ventricular com frequência alta no caso de FA. Caso o eletrodo atrial seja pouco eficiente na detecção de FA, a frequência máxima de seguimento deve ser programada em valor reduzido.

Por fim, um significante número de pacientes com CMH recebe CDI para prevenção de morte súbita. Para esses pacientes, um dispositivo bicameral programado em DDD com intervalo AV curto pode reduzir o gradiente na VSVE e prevenir ou retardar a necessidade de intervenções complementares.

As recomendações para o implante de MP definitivo em pacientes com CMH estão elencadas na Tabela 7 .

**Tabela 7:** – Recomendações para implante de marca-passo (MP) definitivo na cardiomiopatia hipertrófica (CMH)

	Classe de recomendação	Nível de evidência
Pacientes em ritmo sinusal com BAV de 2º grau Mobitz tipo II, grau avançado ou 3º grau espontâneo ou após ablação septal ou miectomia cirúrgica (recomendada estimulação bicameral)	I	B
Pacientes com CMH obstrutiva, com indicação de CDI, um dispositivo bicameral deverá ser considerado	IIa	C
Em pacientes com obstrução do trato de saída do VE (gradiente ≥ 50mmHg), a estimulação bicameral com intervalo AV curto pode ser considerada em casos refratários ao tratamento clínico, que não tenham indicação de CDI e que tenham contraindicação ou não aceitem ablação septal, miectomia cirúrgica ou transplante cardíaco	IIb	C

*CDI: cardioversor-desfibrilador implantável; CHM: cardiomiopatia hipertrófica; MP: marca-passo; VE: ventrículo esquerdo.*

## 2.6. Doenças Neuromusculares

Certas doenças neuromusculares podem provocar progressiva e insidiosa doença do sistema excito-condutor cardíaco. Entre elas, estão a distrofia muscular de Duchene, a distrofia muscular fascioescápuloumeral, a distrofia ligada ao cromossomo X, a miastenia *gravis* , a distrofia miotônica e a ataxia de Friedreich.^97^

As principais manifestações identificadas estão relacionadas a distúrbios da condução infranodal, resultando em bloqueios fasciculares e BAV de 3º grau. Tais achados são particularmente observados na síndrome de Kearns-Sayre (oftalmoplegia externa progressiva, com retinopatia pigmentar), na síndrome de Guillain-Barré, na distrofia muscular miotônica, na distrofia muscular de Becker e na distrofia muscular fascioescápuloumeral.

A distrofia muscular miotônica e a síndrome de Kearns-Sayre são, ambas, associadas com alta incidência de doença do sistema de condução, que frequentemente progride rapidamente e não pode ser prevista por registros eletrocardiográficos ou intracavitários. A doença acomete quase sempre o sistema His-Purkinje e pode culminar ataques de Stokes-Adams ou morte súbita, exceto quando antecipados pelo implante de MP.

Em um estudo com 49 pacientes com distrofia miotônica (média de 46 anos de idade, intervalo HV ≥ 70ms), BAV de alto grau foi registrado em 47% dos pacientes após o implante do MP, mesmo sem evidência de bradicardia no início do estudo.^98^ Os autores concluíram que o implante de MP definitivo deve ser considerado em pacientes com distrofia miotônica com intervalo HV aumentado (≥ 70ms), mesmo quando assintomáticos ou com bradicardia ao ECG.

Nos pacientes com doenças neuromusculares, a espera pela documentação de BAVT pode resultar em significante risco de morte súbita ou síncope. Por isso, o implante de MP definitivo deve ser considerado precocemente no curso da doença neuromuscular, assim que houver qualquer anormalidade da condução, mesmo em assintomáticos ( Tabela 8 ).

**Tabela 8:** – Recomendações para implante de marca-passo (MP) definitivo nas doenças neuromusculares

	Classe de recomendação	Nível de evidência
Pacientes com BAV de 2º grau (tipo II ou avançado) ou 3º grau, com ou sem sintomas.	I	B
Pacientes com BAV de 1º (PR > 240ms) ou QRS alargado (QRS > 120ms) assintomáticos (na distrofia muscular miotônica).	IIb	C

*BAV: bloqueio atrioventricular; MP: marca-passo.*

Anormalidades eletrocardiográficas como ritmo não sinusal, QRS > 120ms, PRi > 240ms, BAV de 2º ou 3º graus e taquiarritmias atriais foram preditores independentes de morte súbita em pacientes com distrofia muscular miotônica tipo 1.^99^

## 2.7. Síndrome da Apneia Obstrutiva do Sono

Bradiarritmias, como bradicardia sinusal, pausas sinusais, BAV de 2º grau tipo I ou de grau avançado e ritmo de escape juncional são frequentes durante o sono, principalmente em jovens saudáveis com bom condicionamento físico. Dados diretos e indiretos têm mostrado relação com hipertonia vagal. Na maioria absoluta dos casos, esses achados são fisiológicos, sem indicação de tratamento específico. Entretanto, têm sido observadas arritmias cardíacas na síndrome da apneia obstrutiva do sono (SAOS).^100^

Os casos mais importantes estão relacionados com síndrome de Pickwick, obesidade, hipertensão arterial sistêmica, síndrome metabólica, obstrução anatômica e/ou funcional das vias aéreas (p. ex., constitucional, macroglossia, hipertrofia do palato mole, hipertrofia tonsilar e de adenoides), doenças pulmonares crônicas, doenças neurológicas e outras.

Quando há obstrução de vias aéreas, durante a apneia, ocorre dessaturação de oxigênio que pode resultar em hipoxemia grave com consequente surgimento de bradi e taquiarritmias atriais e ventriculares.^101^

O tratamento principal é dirigido à correção da apneia e perda de peso. O uso de aparelhos de pressão positiva em vias aéreas superiores (CPAP) para suporte respiratório durante o sono pode ser de grande importância, tendo sido observado, inclusive, desaparecimento das bradiarritmias em boa parte dos casos.^102^ Assim, não há indicação primária de MP para as bradiarritmias relacionadas à SAOS.

Na prática clínica, é frequente que esta condição faça parte de um cenário caracterizado por hipertonia vagal, bradiarritmia noturna e FA comumente deflagrada pela bradicardia (síndrome braditaquicardia).^102^Quando a correção da obstrução das vias aéreas não é suficiente para melhorar o quadro, a ablação por cateter de radiofrequência pode ser indicada (cardioneuroablação e/ou ablação da FA). Em casos excepcionais, o implante de MP pode facilitar o tratamento da FA (betabloqueadores ou outros antiarrítmicos).

A hipertonia vagal excessiva pode ser bem caracterizada pela análise temporal e espectral da variabilidade RR ao *Holter* -24h, comparando-se os períodos de vigília e sono. A variabilidade RR e o número de pausas maiores que 2,5s pré e 1 ano pós-cardioneuroablação foram comparados em um estudo que incluiu 18 pacientes com história de SAOS e braditaquicardia e/ou hipertonia vagal. Nesse estudo, os autores demonstraram importante redução da variabilidade RR (SDNN pré-CNA 131,2 ± 38ms *versus* 91,9 ± 37ms 11 meses pós-CNA, p = 0,0001). Ademais, o número de pausas reduziu significativamente, de 6,5 ± 9,4 pré-CNA para 1,1 ± 3 após 11 meses da CNA, p = 0,03. Nenhum paciente recebeu implante de MP.^103^ Dessa maneira, habitualmente, o implante de MP está reservado aos casos em que se identifica comprometimento do sistema de condução ( Tabela 9 ).

**Tabela 9:** – Recomendações para implante de marca-passo (MP) definitivo na síndrome da apneia obstrutiva do sono (SAOS)

	Classe de recomendação	Nível de evidência
Pacientes com bradiarritmias noturnas, sem cardiopatia significativa, assintomáticos no período de vigília, com SAOS nos quais não foi realizado o tratamento específico	III	C

*SAOS: síndrome da apneia obstrutiva do sono.*

## 2.8. Síndrome do QT Longo Congênito (SQTLc)

A síndrome do QT longo congênito (SQTLc) é uma canalopatia causada por uma anormalidade da repolarização cardíaca e é caracterizada pela presença de intervalo QT prolongado, arritmias ventriculares (extrassistolia ventricular, taquicardia ventricular polimórfica, *torsade de pointes* ) e história de síncope e/ou de morte súbita. Em geral, existe história familiar em parentes próximos e pode ser autossômica recessiva (muito rara), acompanhada de surdez (síndrome de Jervell-Lange Nielsen) ou autossômica dominante, mais frequente (síndrome de Romano Ward). Esses dois tipos perfazem 90% dos casos de SQTLc; porém, atualmente, são conhecidos pelo menos 14 tipos diferentes da forma congênita dessa síndrome.^104^

Existem portadores da anomalia genética que não apresentam nenhuma manifestação clínica espontânea, podendo moastrar, diante de certas condições, como uso de certos fármacos, estresse físico ou alterações eletrolíticas.

A medida do intervalo QT (QTi) pode ser realizada em qualquer derivação, porém, mais frequentemente, são utilizadas as derivações D2 e V5.^105^ A medida é feita do início do complexo QRS até o final da onda T, excluindo-se a onda U. O QTi varia em condições fisiológicas, inversamente proporcional à frequência cardíaca. Desse modo, o valor normal deve ser corrigido pela frequência, sendo a fórmula de *Bazett* a mais utilizada.^106^ Nesta, o QTi medido deve ser dividido pela raiz quadrada do intervalo RR precedente, sendo as unidades medidas em segundos. O resultado é o QT corrigido (QTc), cujo limite normal é 450ms e 460ms, respectivamente, para os gêneros masculino e feminino. Intervalo QTc > 480ms 4 minutos após teste de esforço é altamente sugestivo desta síndrome. Aproximadamente 20% dos casos com genótipo positivo apresentam QT normal.

Nos diversos tipos de síndrome de SQTLc, seja por redução na função dos canais de potássio ou por aumento na função do canal de sódio (retardo na inativação dos canais), ocorre retardo na repolarização celular que se manifesta por aumento do QTi. Aparentemente, as anormalidades eletrofisiológicas são heterogêneas e se tornam muito mais acentuadas diante de algumas condições como estimulação autonômica, estresse físico e mental, alterações eletrolíticas, ação de fármacos, isquemia etc., resultando em instabilidade elétrica, extrassistolia, taquicardia polimórfica, *torsade de pointes* , fibrilação ventricular e morte súbita.

É fundamental, sempre que possível, definir o tipo de SQTLc (especificamente, os tipos 1, 2 e 3) de acordo com a manifestação clínica e eletrocardiográfica, tendo em vista o tratamento recomendado para cada tipo.

Os tipos 1 e 2 são mais frequentes, ocasionados por diminuição da função de canais de potássio; o tipo 3 ocorre por aumento da função dos canais de sódio.^107^ Tipicamente, as manifestações da SQTLc tendem a surgir na infância ou adolescência. Geralmente são mais precoces no sexo masculino (adolescência) do que no feminino (idade adulta). A síncope é a manifestação mais frequente, ocorrendo geralmente entre 5 e 15 anos de idade. História familiar de morte súbita é forte preditor de mortalidade. De modo geral, quanto maior o QTc, maior o risco de morte súbita.

A SQTLc tipo 1 geralmente tem as arritmias deflagradas durante esforço físico, notadamente a natação. Já o tipo 2, mais comumente, apresenta arritmias induzidas por estresse mental ocasionado, por exemplo, por fortes ruídos, principalmente durante descanso ou sono. O tipo 3 tipicamente apresenta as arritmias em repouso ou durante o sono, sem relação clara com uma condição de estresse.

É absolutamente fundamental que esses pacientes evitem distúrbios eletrolíticos como a hipopotassemia e estejam atentos para evitar o uso de fármacos que podem desencadear arritmias fatais. Estão disponíveis na internet diversos *sites* que listam fármacos com potencial risco de prolongamento do QTc (http://www.crediblemeds.org), e devem ser sempre consultados antes do uso de algum medicamento.

Todos os pacientes, sintomáticos, assintomáticos e portadores “silenciosos” devem reduzir acentuadamente a atividade física. Os esportes competitivos estão contraindicados. Certos deflagradores relacionados ao tipo, tais como natação extenuante no SQTL1 e ruídos muito altos na SQTL2, devem ser evitados.^108^Certa liberdade para esportes recreacionais não competitivos pode ser considerada, com cautela, para os pacientes com SQTL3, condicionado ao fácil acesso de um desfibrilador externo automático (DEA) no ambiente. Estão absolutamente contraindicados os medicamentos e as substâncias que prolongam a repolarização, tais como os bloqueadores dos canais de potássio, que podem induzir *torsade de pointes* mesmo em casos assintomáticos. Da mesma forma, também devem ser evitados os simpaticomiméticos. É recomendável que esses pacientes sejam portadores de um documento com a lista dos medicamentos proibidos.

O tratamento farmacológico baseia-se no uso de betabloqueadores, sendo os mais eficazes o propranolol e o nadolol, essencialmente na SQTL tipos 1 e 2. O metoprolol parece ser menos eficaz e não deve ser preferido.^109^

Estudos retrospectivos têm demonstrado benefício inquestionável de betabloqueadores ou denervação cirúrgica (retirada do gânglio estrelado esquerdo), com mortalidade de 9% no grupo tratado *versus* 60% no grupo sem tratamento.

O QTc pode ser reduzido experimentalmente com agentes potencializadores da bomba de potássio, como o nicorandil na SQTL1, ou espironolactona combinada com potássio oral na SQTL2. A SQTL3 pode ser beneficiada com bloqueadores de canal de sódio, como mexiletine ou flecainida, que podem encurtar o QTc, mas este último pode induzir um fenótipo de Brugada. Existem relatos de tratamento com sucesso de tempestade elétrica na SQTL3 com mexiletine, que é recomendada por alguns profissionais quando há um QTc muito longo.

Não há, atualmente, indicação primária de implante de MP para prevenção de morte súbita na SQTLc, visto que o CDI confere segurança mais ampla.^110^ Entretanto, o MP pode ser indicado em casos em que se identifica BAV ou quando existem arritmias ventriculares deflagradas ou agravadas por bradicardia ou pausas, desde que não haja história de morte súbita recuperada e que estejam ausentes os sinais de alto risco: surdez congênita, síncope, arritmias ventriculares complexas documentadas, história familiar de morte súbita, sexo feminino, QTc > 0,60s. Eventualmente, o MP pode ter indicação em conjunto com a terapia betabloqueadora para evitar bradicardia resultante da própria ação farmacológica. A estimulação cardíaca em uma frequência acima da frequência sinusal espontânea pode, reflexamente, inibir a ação simpática e ser bastante útil para controle de tempestades arrítmicas. O MP deve estimular somente átrio (evitar dissincronia ventricular) e pode ser programado com frequência mais elevada (p. ex., 80ppm), que pode reduzir a duração do QTc. Ademais, a ativação do sensor de resposta de frequência pode garantir adaptação cronotrópica, prejudicada devido ao uso de betabloqueadores ( Tabela 10 ).

**Tabela 10:** – Recomendações para implante de marca-passo (MP) definitivo na SQTLc

	Classe de recomendação	Nível de evidência
Pacientes de baixo risco (ausência das condições de alto risco*), principalmente na SQTL3, com bradiarritmia (sinusal ou BAV) agravada ou não pelo uso de betabloqueadores	IIb	C

*Alto risco na SQTLc: surdez congênita, síncope, arritmias ventriculares complexas documentadas, história familiar de morte súbita, sexo feminino, QTc > 0,60s. SQTLc: síndrome do QT Longo Congênito.

A denervação cirúrgica pode ser alternativa nos casos de síncopes recorrentes, apesar do uso regular em dose plena de betabloqueadores ou quando há impedimento para a terapia farmacológica otimizada (TFO) (p. ex., asma).

## 2.9. Coração Transplantado

A taxa de implante de MP definitivo após o transplante cardíaco varia entre 2% e 24% e tem apresentado queda significativa com emprego da técnica de anastomose bicaval, em relação às técnicas de anastomose biatrial.^111 - 113^

A maioria dos estudos reporta a DNS como a anormalidade mais comum encontrada. As causas de DNS são variadas e incluem: traumatismo cirúrgico, danos à artéria do nó sinusal por traumatismo e isquemia, tempos prolongados de isquemia cardíaca, denervação cardíaca e características basais do coração doado.^114^

Cerca de 10% dos pacientes que necessitam de MP apresentam distúrbios da condução AV, principalmente BAV de 2º e 3º graus, sendo postulado que essas alterações provavelmente estejam relacionadas com preservação inadequada do enxerto.^115^

A bradicardia é comum no período precoce após transplante cardíaco, ocorrendo em cerca de dois terços dos pacientes; porém, frequentemente, tende a resolver espontaneamente. Se a bradicardia perdurar por algumas semanas e cursar com sintomas, o implante de MP pode ser necessário.

Estudos demonstram que vários pacientes com DNS e bradicardia que foram submetidos a implante de MP não são dependentes do dispositivo no final de 3 meses. Entretanto, os pacientes com BAV precoce, frequentemente, requerem estimulação cardíaca a longo prazo^116 , 117^( Tabela 11 ).

**Tabela 11:** – Recomendações para implante de marca-passo (MP) definitivo após transplante cardíaco

	Classe de recomendação	Nível de evidência
Pacientes sintomáticos com bradiarritmias ou incompetência cronotrópica em que a resolução espontânea não é esperada ou que, mesmo que transitória, pode persistir por meses, o implante de marca-passo deve ser considerado	IIa	C

## 2.10. Escolha do Tipo de Marca-passo e do Modo de Estimulação

Estudos clínicos randomizados não evidenciaram impacto na sobrevida com a estimulação atrial ou atrioventricular (AAI/DDD) em relação à estimulação ventricular exclusiva (VVI); entretanto, demonstraram benefícios em relação à redução de ocorrência de FA, incidência de síncopes e síndrome do MP.^32 - 35^ Em uma revisão sistemática, os autores demonstraram significativa redução das taxas de AVC ( *hazard ratio* [HR]:0,81) e FA (HR:0,80) com a estimulação atrioventricular sequencial em comparação com a estimulação ventricular.^36^

**Figura 1:**
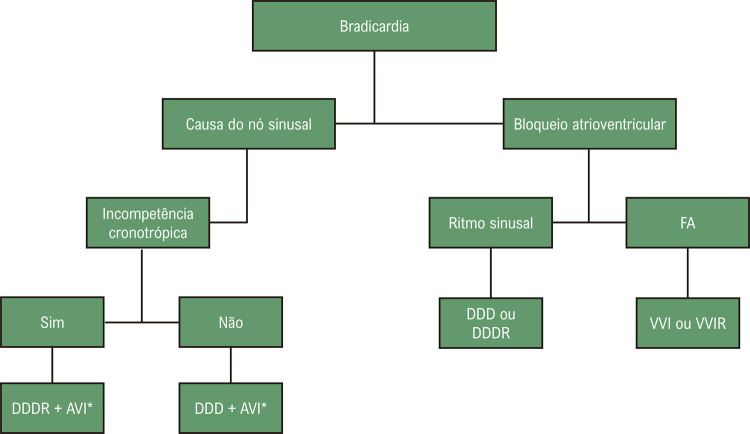
– Algoritmo para preservação da condução atrioventricular intrínseca.

Em pacientes com bradicardia persistente, a estimulação bicameral é o modo de escolha. Os resultados do estudo DANPACE mostraram que a estimulação AAIR associou-se com maior incidência de FA paroxística (HR:1,24) e o dobro de reoperações quando comparada com a estimulação DDDR.^118^ A estimulação ventricular deve ser evitada em pacientes em ritmo sinusal, uma vez que pode causar FA e piora de IC^119^ ( Figura 1 ). Ressalta-se que a programação de intervalo AV excessivamente longo, visando evitar estimulação ventricular, em pacientes com condução AV espontânea, pode ser prejudicial do ponto de vista hemodinâmico em pacientes com BAV de 1º grau amplo, podendo causar regurgitação mitral diastólica, pseudossíndrome do MP e FA.^120^

A ativação do sensor de variação de frequência pode ser benéfica em pacientes com incompetência cronotrópica. O estudo ADEPT comparou os modos DDDR *versus* DDD em termos de melhoria de qualidade de vida em 872 pacientes com incompetência cronotrópica. Aos 6 meses de seguimento, os pacientes randomizados para o modo DDDR tiveram maior pico de frequência cardíaca em comparação com aqueles em modo DDD (113,3ppm x 101,1ppm; p < 0,0001).^121^ Contudo, com 1 ano, não ocorreram diferenças significativas entre os dois grupos com respeito à escala de atividade ou desfechos secundários de qualidade de vida.

## 2.11. Estimulação Direta do Sistema Excito-condutor Cardíaco (Feixe de His, Ramo Esquerdo)

O remodelamento e a consequente disfunção ventricular esquerda promovidos pela dissincronia associada à estimulação artificial do VD são desfechos que justificam a busca por sítios alternativos para estimulação em pacientes com bradiarritmias que necessitam de estimulação ventricular artificial.

O estudo MOST demonstrou que, em pacientes com disfunção sinusal, a estimulação ventricular na ponta do VD determinou aumento significativo de episódios de FA e internação por IC.^35^ Mais importante ainda: esses eventos adversos estiveram diretamente relacionados ao percentual cumulativo de estimulação ventricular.

Dessa maneira, a busca por uma forma de estimulação artificial que mantenha o sincronismo intra e interventricular, além da correção da bradiarritmia, é uma necessidade de relevância clínica que vem sendo remetida à chamada estimulação fisiológica.^122 , 123^

A estimulação direta do sistema de condução é a maneira mais fisiológica de estimulação ventricular artificial porque mantém a ativação elétrica natural do coração, uma vez que o estímulo segue pelas vias normais de condução especializada (His-Purkinje), evitando a dissincronia induzida pela estimulação muscular do VD.^124^

Sharma et al. demonstraram, em estudo não randomizado com 202 pacientes em seguimento de 2 anos, que a estimulação pelo feixe de His foi superior à estimulação convencional pela ponta do VD. Nos pacientes com mais de 40% de percentual de estimulação ventricular, houve redução significativa da necessidade de internação por IC (15% *vs.* 2% p = 0,02).^125^

A estimulação do feixe de His apresenta algumas limitações, que incluem dificuldade técnica de localização do sítio mais apropriado para estimulação (maior tempo cirúrgico), limiares de estimulação mais elevados, menor amplitude de sinal intracavitário e possibilidade de inibição anormal por *cross-sensing* .^126^

A estimulação direta do ramo esquerdo ou de região próxima por via septal profunda (eletrodo entregue via bainha, perfurando até o lado esquerdo do septo interventricular) é alternativa viável para manutenção de QRS estreito e prevenção de dissincronia. Apesar da diferença técnica entre a captura direta do ramo esquerdo e a captura da região do ramo esquerdo, do ponto de vista funcional, a estimulação da região do ramo esquerdo é capaz de promover sincronismo equivalente à estimulação direta do feixe de His.

Mafi-Rad et al. demonstraram, em uma pequena serie de 10 pacientes com DNS, a viabilidade de estimular a região esquerda do septo interventricular resultando em QRS estreito, com padrão de atraso final pelo ramo direito, com limiares estáveis e melhor *performance* hemodinâmica (mensurada pela dP/dT) do que com a estimulação apical do VD.^127^ Mais tarde, Huang demonstrou a possibilidade de captura direta do ramo esquerdo por meio da estimulação septal profunda, corrigindo o BRE em pacientes com essa abordagem, e estabeleceu critérios para definição de captura do ramo esquerdo que incluem pelo menos três critérios: 1) presença de potencial de ramo esquerdo captado no sinal do EGM do eletrodo; 2) tempo de ativação da parede livre do VE (LVAT) menor que 90ms sem modificação com energia de estimulação de 2V ou 5V; 3) padrão de BRD incompleto ao ECG; 4) evidência de captura seletiva e não seletiva do ramo esquerdo; 5) evidência de estimulação direta do ramo esquerdo através de eletrodo concomitante no His ou no septo esquerdo.^128 , 129^

A estimulação fisiológica através do feixe de His ou do ramo esquerdo vem sendo utilizada com eficiência em vários cenários de bradiarritmias com necessidade de estimulação ventricular ( Tabela 12 ).

**Tabela 12:** – Recomendações para estimulação fisiológica (feixe de His, ramo esquerdo) para tratamento de bradiarritmias

	Classe de recomendação	Nível de evidência
DNS com indicação de MP convencional em paciente com retardo de condução intraventricular	IIa	C
FA permanente com indicação de ablação da junção AV para controle de FC	IIa	C
BAV sem disfunção sistólica de VE	IIb	B

*AV: atrioventricular; BAV: bloqueio atrioventricular; DNS: doença do nó sinusal; FA: fibrilação atrial; FC: frequência cardíaca; VE: ventrículo esquerdo.*

Ambas as estratégias de estimulação (His, ramo esquerdo) têm demonstrado segurança, estabilidade e melhores resultados de medidas de sincronia, duração do QRS e tendência de melhora da função ventricular nas séries que compararam com a estimulação pelo VD. Quando comparadas as duas técnicas, a estimulação direta do feixe de His é capaz de promover um QRS normal e, aparentemente, ainda mais fisiológico, mas à custa de tempo maior de implante, limiares mais elevados e menor valor de onda R que a estimulação do ramo esquerdo.^130 , 131^

A evolução da tecnologia das ferramentas de implante, a disponibilidade de geradores dedicados que possibilitem algoritmos específicos para detecção e gasto de energia proporcional e os resultados de estudos controlados a longo prazo determinarão o papel das técnicas de estimulação fisiológica possivelmente como preferencial em futuro próximo.^132^

## 2.12. Estimulação sem Cabo-eletrodo (Leadless Pacemaker)

A estimulação cardíaca artificial não é isenta de problemas. A incidência de complicações com cabos-eletrodos e o gerador de pulsos, especialmente em loja subcutânea, aumenta ao longo dos anos de seguimento e pode afetar mais de 10% dos portadores desses dispositivos.

O implante de sistemas convencionais associa-se a risco de pneumotórax, hemotórax, deslocamento dos cabos-eletrodos, oclusão venosa, insuficiência da valva tricúspide e infecção (que, por vezes, pode cursar com endocardite). Ademais, o implante subcutâneo também se associa à ocorrência de hematoma da loja e infecção, que podem ocorrer não só na primeira cirurgia, mas também no momento da troca do gerador.^133 , 134^ De todas as complicações descritas, a endocardite infecciosa merece especial destaque pela elevada morbidade e mortalidade associadas.^135 , 136^

Sendo o cabo-eletrodo de MP a fonte principal de problemas e complicações, é natural que a tecnologia tenha avançado no sentido de privilegiar soluções em que fosse possível dispensá-lo. Neste sentido, o MP sem cabo-eletrodo ( *leadless pacemaker* ) é uma evolução tecnológica que traz algumas vantagens potenciais em relação aos sistemas convencionais. Inicialmente, foram lançados no mercado dois sistemas – Nanostim ( *Abbott Medical Inc. Abbott Park, IL, USA* ) e MICRA TPS ( *Medtronic, Inc., MN, USA* ), mas, atualmente, apenas o MICRA TPS é comercializado.

Trata-se de um uma pequena “cápsula” (26mm de comprimento por 6,7mm de diâmetro) em que está contido o eletrodo e o gerador de pulsos, que é implantado na cavidade cardíaca por via transvenosa. Todo o processo é relativamente simples e envolve a cateterização da veia femoral, passando uma bainha (de grande calibre – 24F) pela veia cava inferior até ao átrio direito. Um sistema de entrega segue depois, dentro dessa bainha, e permite libertar o dispositivo no VD. A posição inicial recomendada para o implante era o apex do VD, mas atualmente tem sido preconizado que seja implantado no septo interventricular.^137^

O sistema MICRA foi avaliado em um ensaio clínico prospetivo, multicêntrico (MICRA IDE), de braço único, que incluiu 725 pacientes (idade média 75,9 ± 10,9 anos, 58,8% do sexo masculino) com indicação de implante de MP definitivo de câmara única. O objetivo principal foi avaliar a eficácia (limiar de captura no seguimento de 6 meses) e segurança (complicações maiores). O implante foi bem-sucedido em 99,2% dos casos. Ocorreram 28 complicações maiores em 3,4% dos pacientes, tendo sido registados perfuração ou derrame pericárdico (1,6%), complicações no local de acesso vascular (0,7%) e limiar de estimulação elevado (0,3%). Não ocorreram deslocamentos ou embolizações do dispositivo. Os valores médios de onda R, limiar de estimulação e impedância permaneceram estáveis. Nesse estudo, a taxa de complicações foi comparada com uma população de mais de 2.000 pacientes (controles históricos de outros ensaios clínicos de MP convencionais da mesma marca), tendo-se verificado menor taxa de complicações maiores (HR 0,49 IC95% 0,33-0,75, p = 0,001), incluindo menor número de hospitalizações (2,3% *vs.* 3,9%) e de necessidade de revisão de sistema (0,4% *vs.* 3,5%). Destaca-se, ainda, baixa taxa de infecções, não relacionados com o implante ou com a presença do dispositivo.^138^ Esses resultados foram depois confirmados no seguimento anual dessa população.^139^ A análise dessa mesma coorte mostrou melhoria de parâmetros de qualidade de vida aos 3 e 12 meses e elevados níveis de satisfação. No mesmo estudo, o sistema MICRA foi associado a menos restrições na atividade que os sistemas convencionais.^140^

O sistema MICRA foi também já avaliado em registos do “mundo real”. O maior desses registos é multicêntrico (96 centros em 20 países) e pretende incluir 1.830 doentes. O desfecho primário do estudo é a ocorrência de complicações nos primeiros 30 dias pós procedimento. Os resultados dos primeiros 795 doentes foram já publicados e demonstraram elevada taxa de sucesso no implante (99,6%), com baixa taxa de complicações maiores (1,5%). Cerca de 20% dos pacientes desse registo tinham contraindicação para implante de sistema convencional (sobretudo por problemas relacionados com acesso vascular). Na população estudada, ocorreram 5 derrames pericárdicos (dois com necessidade de drenagem).^141^ Pequenos estudos unicêntricos de seguimento de centro único que avaliam o *leadless pacemaker* no mundo real também foram publicados e confirmam a elevada taxa de sucesso no implante, assim como baixa taxa de complicações.^142 - 144^

A segurança do implante do sistema também foi avaliada em populações especiais, como pacientes em hemodiálise (HD) e após extração de dispositivos convencionais por infecção.

O dispositivo Nanostim foi também avaliado em estudo multicêntrico e observacional,^145 , 146^ cujos resultados obtidos até 6 meses de seguimento não diferem dos resultados obtidos com os estudos do MICRA. O motivo para ter sido retirado do mercado tem a ver com falência inesperada da bateria, que impede estimulação ventricular e comunicação com o dispositivo em cerca de 0,5% dos casos.^147^

Além das complicações intraoperatórias que já foram referidas, existem duas situações que constituem, ainda, uma área de incerteza. Em primeiro lugar, ainda não está claro se o dispositivo tem interferência ou não na função da válvula tricúspide. Em um estudo inicial, com 2 meses de seguimento de 23 pacientes, concluiu-se que não existia interferência na função da válvula tricúspide.^148^ Outro estudo mais recente, que incluiu 53 pacientes, concluiu que o *leadless pacemaker* interfere na função da válvula e pode causar ou agravar insuficiência tricúspide.^149^ O mecanismo mais provável é a interferência mecânica do dispositivo com o aparelho subvalvular, sendo a lesão aguda (durante o implante) da válvula ou a dissincronia induzida pelo MP as causas menos prováveis. Os autores desse estudo descrevem, ainda, que os doentes em que o dispositivo fica em posição septal (a posição atualmente recomendada, pelo menor risco de perfuração) são aqueles em que mais vezes se observou insuficiência tricúspide, provavelmente pela maior proximidade à válvula e ao aparelho subvalvular.

Outra área de incerteza é a atitude a tomar no fim da vida do gerador. Está publicada a experiência mundial com o explante precoce do sistema MICRA – entre o primeiro e o 95ª dia – com bons resultados e baixa taxa de complicações.^150^ Está também publicado um relato de um caso clínico em que foi possível extrair o dispositivo, por via percutânea, 4 anos após o implante.^151^ É, no entanto, desconhecido qual vai ser o comportamento em seguimento de longo prazo e se será possível a extração (ou se adiciona-se outro dispositivo em outra localização).^152 , 153^

É necessário, também, não esquecer que, com o aparecimento de novas tecnologias, podem surgir novas complicações não descritas – que poderão aparecer quando existirem seguimentos mais longos. Importante também recordar que não existe, até a data presente, qualquer comparação randomizada entre sistemas de MP convencionais e *leadless pacemaker* .

O dispositivo disponível atualmente é de câmara única e permite resposta em frequência (VVIR). As indicações, em termos gerais, são as mesmas de um MP de câmara única – de forma muito genérica: bradicardia sintomática em que se considera não ser necessário eletrodo atrial ( Tabela 13 ).

**Tabela 13:** – Recomendações para implante de marca-passo (MP) sem eletrodo

	Classe de recomendação	Nível de evidência
*Leadless pacemaker* é recomendado em pacientes com complicação de dispositivos convencionais (fratura de eletrodo, após extração por infecção), nos quais existem obstruções venosas críticas	IIa	C
*Leadless pacemaker* é uma opção aceitável em pacientes com fibrilação atrial com baixa resposta ventricular como primeira opção desde que discutidas com o paciente as vantagens e as limitações	IIb	B
*Leadless pacemaker* é uma opção aceitável em pacientes em ritmo sinusal, com indicação de MP com expectativa de pouca estimulação, desde que discutidas com o paciente as vantagens e as limitações (p. ex., pausas sinusais raras ou BAV paroxístico raro) *Leadless pacemaker * pode ser considerado em pacientes com BAVT em que se consideraria estimulação unicameral ventricular (muito idosos, pouca atividade, acamados, desde que discutidas as vantagens e as limitações)	IIb	C
*Leadless pacemaker* de câmara única ventricular em pacientes com BAVT, em ritmo sinusal, candidatos à estimulação convencional de dupla câmara quando é desejável a manutenção do sincronismo atrioventricular	III	C
*Leadless pacemaker* não está indicado em crianças e jovens (incerteza quanto à conduta quando o gerador chega ao fim de vida útil)	III	C

*BAV: bloqueio atrioventricular; BAVT: bloqueio atrioventricular total; MP: marca-passo.*

Um dos principais fatores limitantes à utilização desse tipo de dispositivo é o preço elevado, como ficou demonstrado em *survey* recente da EHRA.^154^

Conceitualmente, os melhores candidatos seriam aqueles com contraindicações relativas aos dispositivos convencionais, por exemplo, por ausência de acessos vasculares, pacientes com risco de nova cirurgia, hemodiálise e após infecção de sistemas convencionais. Existem estudos (a maioria de centro único) que demostram eficácica do *leadless pacemaker* nesses subgrupos.^155 - 157^ Esses pacientes estão também representados nos estudos de “vida real” já referidos.

A técnica de implante é distinta da técnica convencional; por isso, é importante ter experiência em estimulação cardíaca, em acessos vasculares femorais, em manipulação de bainhas de grande calibre e manipulação de eletrodos no VD. A perfuração cardíaca não é frequente (pode ocorrer no VD, mas também no átrio e na aurícula) e, quando ocorre, faz com que exija habitualmente resolução cirúrgica de emergência.^158 , 159^

## 3. Recomendações para Implante de Marca-passo Multissítio/Terapia de Ressincronização Cardíaca (TRC)

### 3.1. Paciente em Ritmo Sinusal

A despeito da TFO, muitos pacientes com IC com FEVE reduzida (ICFEr) evoluem com persistência de sintomas e importante disfunção sistólica do VE. Os fatores mais comuns relacionados com a baixa resposta à TFO são a insuficiência mitral moderada ou grave, reserva funcional miocárdica reduzida e a dissincronia ventricular (DV). Com relação a esta última, a TRC, por meio da estimulação cardíaca atriobiventricular, associada ou não ao CDI, tem sido considerada excelente opção terapêutica para pacientes com BRE. A TRC tem o propósito de corrigir disfunções eletromecânicas em pacientes com ICFEr que apresentem a DV e, por consequência, melhorar a *performance* do VE.

O ECG de superfície é o método de eleição na pesquisa de DV e seleção de pacientes para a TRC. Apesar de os métodos de imagem, como o ecocardiograma, serem capazes de detectar a DV mecânica, o estudo PROSPECT (Predictors of Response to CRT) demonstrou que o doppler tecidual não conseguiu identificar os pacientes respondedores à TRC.^160^ O estudo COMPANION (Comparison of Medical Therapy, Pacing, and Defibrillation in Heart Failure)^161^ e o CARE-HF (Cardiac Resynchronisation in Heart Failure Study)^162^ foram os primeiros estudos randomizados de larga escala testando a TRC em desfechos clínicos de mortalidade total e taxa de hospitalização. Os achados desses estudos demonstraram aumento da sobrevida proporcionado pelo acréscimo da TRC à TFO.

Esses resultados foram confirmados por metanálise publicada em 2006, que incluiu oito ensaios clínicos com o total de 3.380 pacientes.^163^ Em seguimento médio de 29,4 meses, foram observados 524 óbitos, com redução marcante da mortalidade (OR:0,72, IC95% 0,59 a 0,88) e da taxa de hospitalizações por IC (OR: 0,55, 95%IC 0,44 a 0,68) com a TRC. Em todos os estudos incluídos, houve melhora significativa da qualidade de vida (3 a 6 meses), apesar da heterogeneidade dos critérios de tempo de avaliação. Ademais, o número necessário para tratar (NNT) foi estimado em 11 (necessário implantar 11 dispositivos para salvar 1 vida em 2,5 anos). Considerando-se a longevidade média dos aparelhos de TRC (6 anos), seria necessário implantar 5 dispositivos para evitar 1 óbito.^164^

Esses estudos embasaram as primeiras indicações da TRC como terapêutica coadjuvante à TFO na ICFEr avançada (FEVE ≤ 35% e classe funcional (CF) NYHA III ou IV a despeito da TFO por mais de 3 meses) que apresentassem DV, constatada pela presença de distúrbio da condução intraventricular (DCIV) no ECG. Ressalta-se que o estudo COMPANION também demonstrou maiores benefícios clínicos com o acréscimo do CDI à TRC (TRC-D).

Subsequentemente, foram publicados os estudos MADIT-CRT (Multicenter Automatic Defibrillator Implantation Trial With Cardiac Resynchronization Therapy),^165^ REVERSE (Resynchronization Reverses Remodeling in Systolic Left Ventricular Dysfunction)^166^ e RAFT (Resynchronization–Defibrillation for Ambulatory Heart Failure Trial).^167^ Esses estudos compararam o TRC-D *versus* o CDI isolado, em pacientes com FEVE ≤ 40% (REVERSE) ou ≤ 30% (MADIT-CRT e RAFT) e CF NYHA I-II (REVERSE e MADIT-CRT) ou II-III (RAFT). Os resultados, ressaltados em metanálise, permitiram comprovar os benefícios da TRC, não só como terapêutica adicional à TFO, mas também ao CDI na redução da mortalidade total.^168^

Em relação aos pacientes com ICFEr assintomáticos (CF NYHA I) ou em CF NYHA II incluídos em cinco estudos clínicos randomizados, metanálise demonstrou redução significativa da mortalidade total e taxa de hospitalização por IC nos pacientes com CF NYHA II.^165 - 167 , 169 , 170^ Destaca-se que apenas 9% dos pacientes estudados tinham CF NYHA I e, nessa população, a TRC reduziu significativamente a taxa de hospitalização por IC, mas não a mortalidade total.^171^ Esses resultados demonstram que a TRC, instituída precocemente nos pacientes com ICFEr assintomáticos, pode reduzir a progressão da IC, possivelmente por meio do remodelamento reverso ventricular. Entretanto, os potenciais benefícios da TRC em pacientes com ICFEr e CF NYHA I devem ser cuidadosamente avaliados em relação aos possíveis eventos adversos e custos associados ao implante da TRC.

A duração do QRS ≥ 120ms como ponto de corte na indicação da TRC foi baseada nos critérios de inclusão dos estudos COMPANION^161^ e CARE-HF.^162^ Porém, em 2013, houve as publicações dos resultados do estudo ECHO CRT que demonstraram aumento da mortalidade cardiovascular no subgrupo de pacientes com QRS < 130ms submetidos a TRC.^172 , 173^ Esses achados foram corroborados em metanálise que demonstrou pouco benefício da TRC nos pacientes com QRS < 140ms. De fato, quanto maior a duração do QRS, melhor é a resposta à TRC.^174 , 175^ Ademais, os pacientes com BRE e duração do QRS ≥ 150ms são os que mais se beneficiam da TRC.^165 , 176 , 177^ Esse dado também foi confirmado na metanálise que reuniu 12.638 pacientes de 13 grandes estudos, corroborando o benefício da TRC em pacientes com BRE e o maior risco de morte por falência da bomba cardíaca nos pacientes com QRS mais alargados.^175^

Em relação ao tipo de DCIV, subanálise de grandes estudos sugerem que pacientes com QRS largo não BRE apresentam pior resposta à TRC. Dados do estudo REVERSE, comparando o ressincronizador cardíaco ligado *versus* desligado, mostrou que pacientes não BRE apresentaram ausência de remodelamento reverso do VE, independentemente da duração do QRS.^178^ Da mesma forma, estudo utilizando a população do MADIT-CRT também demonstrou ausência de benefícios clínicos da TRC-D em 537 pacientes com ICFEr leve e não BRE, independentemente da morfologia e da duração do QRS.^179^

Importante ressaltar que poucos pacientes com bloqueio do ramo direito (BRD) foram incluídos nos grandes estudos, dificultando uma conclusão definitiva em relação aos efeitos da TRC nessa população.^180^

Por outro lado, apesar de as subanálises desses estudos não observarem benefícios da TRC em pacientes não BRE, recente estudo observacional, de mundo real, utilizando informações do National Cardiovascular Data Registry (EUA), avaliou a resposta clínica da TRC-D *versus* CDI em 11.505 pacientes não BRE. Este estudo demonstrou que, em pacientes com DCIV inespecífico com QRS ≥ 150ms, a TRC reduziu mortalidade e taxa de hospitalização por IC, enquanto aqueles com QRS < 150ms tiveram má resposta clínica à TRC, com aumento de mortalidade e taxa de hospitalização.^181^ Finalmente, esse mesmo estudo constatou aumento no risco de morte e na taxa de hospitalização por IC em pacientes com BRD tratados com a TRC-D. Essa conclusão foi consistente com resultados de Bilchick et al.,^182^ publicado em 2010, que utilizaram as informações do Medicare. Adicionalmente, Pastore et al.^183^ demonstraram que pacientes com BRD típico, definido classicamente como QRS > 120ms de duração, padrão rsr’, rsR’ ou rSR’ na derivação V1 ou V2 e onda S maior que onda R ou > 40ms de duração nas derivações D1 e aVL,^184^ apresentavam má resposta à TRC. Portanto, os dados disponíveis na literatura até o momento apontam para a indicação da TRC em pacientes com ICFEr e DCIV inespecífico com duração do QRS ≥ 150ms, e reforçam a necessidade de maior cautela na indicação desta terapêutica nos pacientes com BRD típico.

As indicações de TRC em pacientes em ritmo sinusal estão listadas na Tabela 14 .

**Tabela 14:** – Recomendações para indicação de terapia de ressincronização cardíaca (TRC) em pacientes com ritmo sinusal

	Classe de recomendação	Nível de evidência
TRC é recomendada para pacientes com ICFEr sintomática em ritmo sinusal, com BRE e QRS ≥150ms, com FEVE ≤ 35% a despeito da TFO	I	A
TRC é recomendada para pacientes com ICFEr sintomática em ritmo sinusal, com BRE e QRS entre 130 e 149ms, com FEVE ≤ 35% a despeito da TFO	IIa	B
TRC pode ser útil em pacientes com ICFEr com CF NYHA III ou IV ambulatorial, ritmo sinusal, com distúrbio de condução intraventricular não BRE e QRS ≥150ms, com FEVE ≤35% a despeito TFO	IIa	B
TRC pode ser útil em pacientes com ICFEr com CF NYHA III ou IV ambulatorial, ritmo sinusal, com distúrbio de condução intraventricular não BRE e QRS entre 130-149ms, com FEVE ≤35% a despeito de TFO	IIb	B
TRC não é recomendada em pacientes com QRS <130ms, sem outra indicação para estimulação de ventrículo direito	III	A

*TRC: terapia de ressincronização cardíaca; ICFEr: insuficiência cardíaca com fração de ejeção reduzida; BRE: bloqueio de ramo esquerdo; FEVE: fração de ejeção do ventrículo esquerdo; TFO: terapia farmacológica otimizada; NYHA: New York Heart Association; CF: classe funcional.*

### 3.2. Paciente com Fibrilação Atrial

A prevalência de FA em pacientes com IC varia de acordo com a gravidade, ocorrendo em 5% dos pacientes em classe funcional I (NYHA) e chegando a 40% em pacientes em classe funcional IV (NYHA).^185^

Os dados sobre a TRC em pacientes com FA e IC são limitados, mas sugerem benefícios ainda que sejam menores que em pacientes em ritmo sinusal. Isso ocorre devido a algumas peculiaridades relacionadas com a FA, tais como perda do sincronismo atrioventricular, maior risco de falha da estimulação ventricular sincronizada devido à dificuldade de controle da frequência cardíaca e ocorrência de batimentos de fusão e pseudofusão, maior incidência de disparo de choques de CDI (apropriados ou inapropriados), além de maior incidência de hospitalização e mortalidade.^186 - 190^

Ritmo de FA ocorre em cerca de um quarto dos pacientes submetidos à TRC; entretanto, a maioria dos ensaios clínicos controlados e randomizados que demonstraram o benefício da TRC excluiu pacientes com FA (p. ex., CARE-HF, COMPANION).^191^

No estudo CARE-HF, que comparou TRC com TFO, embora a mortalidade tenha sido mais elevada em pacientes que desenvolveram FA durante o seguimento, esses pacientes se beneficiaram da TRC quando considerados os principais objetivos do estudo.^192^

A associação de TRC com CDI (TRC-D) não foi superior ao CDI isoladamente no subgrupo de 229 pacientes com FA do estudo RAFT. Entretanto, menos de um terço dos pacientes recebeu mais que 95% de estimulação biventricular nos 6 meses de seguimento.

Uma metanálise^193^ que incluiu cinco estudos (quatro coortes prospectivas e o ensaio clínico randomizado MUSTIC^194^) comparou as respostas à TRC em 797 pacientes em ritmo sinusal e 367 pacientes em FA (ablação de nó AV foi realizada em 56 pacientes). Não houve diferença na melhora da classe funcional (NYHA) entre os pacientes, embora os resultados do teste de caminhada de 6 minutos e qualidade de vida (escore de Minnesota) tenham sido melhores no grupo em ritmo sinusal.

Em outra metanálise,^195^ com 23 estudos observacionais, que incluiu 7.495 pacientes submetidos à TRC, os pacientes com FA (25%) tiveram maior taxa de não respondedores (34,5% *vs.* 26,7%) e maior taxa de mortalidade (10,8% *vs.* 7,1%) quando comparados com pacientes em ritmo sinusal. Ademais, a presença de FA foi associada com menor impacto da TRC na qualidade de vida, no teste de caminhada de 6 minutos e no volume diastólico final do ventrículo esquerdo, mas com resultado semelhante na melhora da fração de ejeção do ventrículo esquerdo.

O estudo MUSTIC-AF^196^ incluiu 59 pacientes com IC e FA com bradicardia (estimulação ventricular com QRS estimulado ≥ 200ms), randomizados para estimulação de VD ou biventricular, com *crossover* no terceiro mês. Ablação do nó AV foi realizada em 63% dos pacientes. O estudo teve como limitação um número baixo de *crossover* , o que limitou qualquer conclusão (somente 39%). Não houve diferença significativa na tolerância ao esforço e pico de consumo de oxigênio entre os dois grupos quando se considerou a intenção de tratar. Entretanto, ao considerar os 37 pacientes que receberam terapia adequada (97% a 100% de estimulação biventricular), encontrou-se um aumento significativo na distância da caminhada em 6 minutos e no pico de consumo de oxigênio.

Os estudos PAVE,^197^ OPSITE^198^ e AVIL CLS/CRT^199^ mostraram que a TRC acrescentou um modesto, mas significante, efeito na qualidade de vida, classe funcional (NYHA) e FEVE em comparação com estimulação apical de VD, em pacientes com FA submetidos à ablação do nó AV, com vários graus de disfunção de VE.

O registo ADHERE^200^ comparou pacientes com IC, QRS ≥ 120ms e FEVE ≤ 35% submetidos a TRC-CDI (n = 4.471) com pacientes que não foram submetidos a implante de dispositivo (n = 4.888). O uso de TRC-D foi associado a menor risco de mortalidade e internação hospitalar. Esta associação foi observada também no subgrupo de 3.357 pacientes com FA.

O benefício da TRC requer estimulação biventricular na maior parte do tempo, evitando ao máximo a condução intrínseca. Em pacientes com FA com condução atrioventricular rápida, essa condição pode ser dificultada.

Boriani et al.^201^ avaliaram 1.404 pacientes submetidos à TRC em seguimento médio de 18 meses. Todos estavam em ritmo sinusal no momento da inclusão no estudo, tendo sido documentada FA em 443 pacientes (32%). A duração dos episódios variou de >10 minutos a semanas e ocorreu tanto em pacientes sem história (22%) quanto em pacientes com história de FA (16%). A porcentagem de estimulação biventricular no grupo que apresentou FA foi de 95% *versus* 98% no total de pacientes. Quando os pacientes que apresentaram em FA estavam em ritmo sinusal, a porcentagem de estimulação biventricular foi de 98% *versus* 71% durante os episódios de FA (p < 0,001). Estimulação biventricular < 95% foi definida como subótima, a qual foi correlacionada com a ocorrência de FA persistente ou permanente (p < 0,001), e frequência ventricular não controlada (p = 0,002). A porcentagem de estimulação biventricular foi inversamente proporcional à frequência cardíaca em pacientes com FA, reduzindo em 7% para cada aumento de 10 batimentos na frequência ventricular.

A importância da alta taxa de estimulação biventricular foi confirmada em uma grande coorte de 36.395 pacientes que participaram do US LATITUDE Patient Management System, na qual os pacientes foram seguidos por monitoramento remoto.^202^ A mortalidade foi inversamente proporcional à porcentagem de estimulação biventricular, tanto em ritmo sinusal quanto em FA ou estimulação atrial. A maior redução na mortalidade foi observada com estimulação biventricular > 98%. Pacientes com estimulação biventricular > 99,6% tiveram redução de mortalidade de 24% (p < 0,001), enquanto os que tiveram estimulação biventricular < 94,8% tiveram aumento de 19%. O tempo ideal de estimulação biventricular foi ≥ 98,7%.

No estudo APAF, prospectivo e multicêntrico, foram incluídos 186 pacientes com FA permanente sintomática com frequência ventricular não controlada ou IC refratária, disfunção sistólica de VE e QRS largo.^203^ Todos foram submetidos a ablação do nó AV e implante de MP multissítio e randomizados para TRC guiada por ecocardiograma (97 pacientes) ou estimulação apical de VD (89 pacientes). No seguimento médio de 20 meses, a TRC reduziu o evento composto por hospitalização ou morte por IC, ou piora da IC (11% no grupo TRC *vs.* 26% no grupo estimulação apical de VD). A TRC reduziu hospitalização e piora da IC. A mortalidade total foi similar entre os grupos.

A ablação do nó atrioventricular (AV) elimina a condução intrínseca, resultando em estimulação biventricular em 100% do tempo em pacientes com TRC. Esta estratégia foi avaliada em uma série de 673 pacientes (FEVE ≤ 35%, NYHA ≥ II, QRS > 120ms).^187^ Entre os 162 pacientes com FA permanente dessa coorte, 48 receberam medicação para controle da frequência cardíaca e 114 pacientes foram submetidos à ablação do nó AV. Em 4 anos de seguimento, o remodelamento reverso e a tolerância ao esforço foram similares entre os pacientes com FA e ritmo sinusal. Entre pacientes com FA, o benefício da TRC foi observado somente nos pacientes que tinham sido submetidos à ablação do nó AV. A despeito da estimulação biventricular >85% do tempo, os pacientes com controle medicamentoso da FC não tiveram melhora na função do VE e da capacidade funcional.

Gasparini e colaboradores demonstraram, em um estudo observacional, que ablação do nó AV associada à TRC melhorou significativamente a sobrevida quando comparada à TRC isolada.^204^ Entre os 1.285 pacientes avaliados, 243 estavam em FA. O controle da FC (85% de estimulação biventricular) foi realizado por ablação do nó AV em 188 pacientes, e terapia medicamentosa em 55. No seguimento de 34 meses, a mortalidade foi significativamente menor em pacientes submetidos à ablação do nó AV (4,3% *vs.* 15,2% HR = 0,26 para mortalidade geral e 0,15 para mortalidade por IC). Esses resultados sugerem que a meta em TRC deve ser 100% de estimulação biventricular, visando ao benefício máximo da terapia.

O estudo CERTIFY ratificou a importância da ablação do nó AV em pacientes com FA submetidos à TRC. O estudo comparou a evolução clínica dos pacientes com FA permanente submetidos à TRC combinada com ablação do nó AV (n = 443) ou controle de frequência com medicamentos (n = 895) com pacientes em ritmo sinusal (n = 6.046). Os resultados mostraram, em seguimento médio de 37 meses, mortalidade por todas as causas (6,8 *vs* . 6,1 por 100 pessoas ano) e mortalidade cardiovascular (4,2 *vs.* 4,0) similares nos pacientes com FA + ablação do nó AV e ritmo sinusal. Em contraste, os pacientes com FA + controle medicamentoso tiveram maior mortalidade total e cardiovascular (11,3 e 8,1, respectivamente; p < 0,001) ( Tabela 15 ).

**Tabela 15:** – Recomendações para indicação de terapia de ressincronização cardíaca (TRC) em pacientes com fibrilação atrial

	Classe de recomendação	Nível de evidência
TRC deve ser considerada em pacientes com FA permanente, FEVE reduzida (< 50%) e indicação de ablação do nó AV para controle de frequência cardíaca	I	B
TRC deve ser considerada em pacientes com FA permanente, FEVE ≤ 35% e CF NYHA III e IV a despeito da terapia medicamentosa otimizada, com BRE e QRS ≥ 130ms, assegurando estratégia que permita estimulação biventricular > 95%	IIa	B
Ablação da junção atrioventricular deve ser considerada em casos de incompleta estimulação biventricular (< 95%)	IIa	B

*CF: classe funcional; FEVE: fração de ejeção do ventrículo esquerdo; NYHA: New York Heart Association; TRC: terapia de ressincronização cardíaca.*

### 3.3. *Upgrade* de Marca-passo Convencional

Pacientes submetidos à estimulação crônica do VD por MP convencional ou CDI podem evoluir com disfunção sistólica progressiva do VE em decorrência de dissincronia elétrica e mecânica. Dados de registros indicam que a disfunção do VE induzida por MP pode ocorrer em 12 a 30% dos pacientes.^205 , 206^ Para esse diagnóstico, deve-se documentar alta taxa de estimulação ventricular, afastadas outras causas de disfunção de VE, como isquemia miocárdica, valvopatias e arritmias sem controle adequado.

Estudos não randomizados indicam que a TRC pode reverter a disfunção induzida por MP.^207 - 209^ A melhora ou recuperação da FEVE observada nesses estudos ocorreu em até 86% dos pacientes. Assim, portadores de MP ou CDI com alta taxa de estimulação ventricular, que apresentam piora clínica e/ou ecocardiográfica, podem ser considerados para *upgrade* de sistema para TRC ( Tabela 16 ).

**Tabela 16:** – Recomendações para indicação de terapia de ressincronização cardíaca (TRC) *upgrade* em portadores de marca-passo convencional

	Classe de recomendação	Nível de evidência
TRC para pacientes com MP convencionais, com alta taxa de estimulação ventricular direita (> 40%), que evoluem com piora clínica e/ou ecocardiográfica, mesmo com tratamento otimizado, afastadas outras causas de disfunção ventricular esquerda (FEVE ≤ 35%).	IIa	B

*MP: marca-passo; TRC: terapia de ressincronização cardíaca.*

### 3.4. Na Indicação de Maca-passo Antibradicardia (1º Implante)

O possível efeito deletério da estimulação ventricular com MP convencional pode justificar a indicação de TRC como opção de escolha para o tratamento de bradicardias.

Estudos sugerem que a disfunção de VE induzida por MP (12 a 30%) é maior quando a taxa de estimulação ventricular excede a 40%, ou até mesmo 20%. A FEVE prévia ao implante e a duração do QRS também foram preditores da ocorrência de disfunção ventricular induzida por MP.

Ensaios clínicos randomizados avaliaram se a TRC seria superior à estimulação convencional do VD para reduzir a ocorrência de remodelamento do VE e desfechos clínicos em pacientes com FEVE > 35%.^210 , 211^ Esses estudos foram avaliados conjuntamente em metanálise que comparou a estimulação convencional do VD com a estimulação biventricular ou do feixe de His.^212^ A estimulação biventricular, em comparação com a estimulação do VD, foi associada a maior FEVE e redução de volumes finais sistólico e diastólico do VE (maior probabilidade de benefício com FEVE entre 36% e 52%). A melhora no teste de caminhada de 6 minutos ocorreu de modo significativo apenas em um estudo que incluiu pacientes com FA permanente que foram submetidos à ablação de nó AV (pacientes com FEVE < 45% e com IC NYHA II/III apresentaram melhor resultado no teste). Os dados relacionados à estimulação do feixe de His estão apresentados em outra sessão deste documento.

Importantes estudos não foram inseridos nesta metanálise por incluírem também pacientes com FEVE < 35%. O estudo BLOCK HF incluiu pacientes com indicação de MP por bloqueio AV, com IC NYHA I-III e FEVE ≤ 50%.^56^ Os pacientes foram randomizados para estimulação convencional do VD ou estimulação biventricular. A FEVE média foi de 43%, sendo que 87% apresentavam FEVE > 35%. O desfecho primário composto de morte por qualquer causa, atendimento de urgência por IC com necessidade de diurético intravenoso e aumento de 15% ou mais no volume sistólico final do VE ocorreu mais frequentemente no grupo de estimulação do VD. O estudo APAF incluiu pacientes com FA permanente submetidos à ablação de nó AV e implante de TRC.^204^ Os pacientes foram randomizados para estimulação biventricular ou estimulação de VD. O desfecho primário composto por morte por IC, hospitalização ou piora da IC ocorreu em 26% dos pacientes no grupo VD e 11% no grupo TRC. O benefício da estimulação biventricular ocorreu independentemente da FEVE e da duração do QRS. A FEVE média dos pacientes foi de 38%, sendo que 53% apresentavam FE > 35%.

Um estudo brasileiro (COMBAT^55^), prospectivo, multicêntrico, randomizado, duplo-cego e cruzado, incluiu 60 pacientes com CF II-IV (NYHA), FEVE < 40% e bloqueio AV como indicação de MP. Todos os pacientes foram submetidos a implante de ressincronizador cardíaco; no entanto, a cada 3 meses, alternaram estimulação biventricular com estimulação convencional de VD (grupo A: VD-TRC-VD e grupo B: TRC-VD-TRC). Ao final de cada trimestre, os pacientes foram avaliados, tendo sido encontrado melhora significativa da qualidade de vida, CF, e volume sistólico final do VE com a TRC em comparação com a estimulação do VD. Ademais, a taxa de mortalidade foi maior com estimulação de VD.

As recomendações de TRC para pacientes com indicação de MP convencional para tratamento de bradiarritmias estão resumidas na Tabela 17 .

**Tabela 17:** – Recomendações para indicação de terapia de ressincronização cardíaca (TRC) a pacientes com indicação de marca-passo convencional

	Classe de recomendação	Nível de evidência
TRC para pacientes com bloqueio atrioventricular, com indicação de implante de marca-passo definitivo e disfunção sistólica de VE (FEVE < 40%).	I	A

*FEVE: fração de ejeção do ventrículo esquerdo; TRC: terapia de ressincronização cardíaca.*

### 3.5. Na Indicação de Cardio-desfibrilador Implantável (TRC-D)

O implante de CDI está indicado em diferentes condições clínicas como estratégia de prevenção primária e secundária de morte súbita (ver item 4). Muitos desses pacientes apresentam também disfunção de VE e bloqueio de ramo esquerdo (BRE), atendendo a critérios de indicação para TRC (ver item 3). Desta forma, há pacientes que podem se beneficiar de ambas as terapias (TRC-D).

Ensaios clínicos randomizados avaliaram os resultados do implante de CDI ou TRC-D em pacientes com disfunção de VE e DCIV. Nos estudos CONTAK, MIRACLE ICD,^213^ MIRACLE ICD II e RethinQ,^214^ todos os pacientes foram submetidos a implante de TRC-D, sendo que um grupo permanecia com a TRC ativada, e o outro, não.

No estudo CONTAK, foram incluídos 490 pacientes com indicação convencional para implante de CDI, com FEVE ≤ 35%, NYHA II-IV, QRS ≥ 120ms. Não houve diferença significativa no desfecho primário composto incluindo morte por qualquer causa, hospitalização por IC e arritmia ventricular com necessidade de intervenção do CDI. A TRC-D melhorou de modo significativo o consumo de O_2_, teste da caminhada e FEVE. O MIRACLE ICD incluiu 369 pacientes, também com indicação convencional para implante de CDI, com FEVE ≤ 35%, NYHA III-IV, QRS ≥ 130ms. A TRC-D foi associada a melhora da qualidade de vida e consumo de O_2_. No MIRACLE ICD II, foram incluídos 186 pacientes, indicação convencional de CDI, FE ≤ 35%, NYHA II, QRS ≥ 130ms. A TRC-D foi associada à redução dos volumes do VE, melhora da FEVE e da classe funcional (NYHA). No estudo RethinQ, foram incluídos 172 pacientes com indicação e CDI, FE ≤ 35%, NYHA III, QRS ≤ 130ms e evidência de dissincronia por ecocardiograma. Não houve benefícios da TRC-D para o desfecho de consumo O_2_.

Já nos estudos MADIT CRT^165^ e RAFT,^167^ os pacientes foram submetidos a implante de TRC-D ou de CDI isolado. No MADIT CRT, participaram 1.820 pacientes com FEVE ≤ 30%, NYHA I-II, QRS ≥ 130ms. Houve redução significativa do desfecho combinado de morte por qualquer causa ou evento não fatal de IC com a TRC-D. No estudo RAFT, foram incluídos 1.798 pacientes com FEVE ≤ 30%, NYHA II-III, QRS ≥ 120ms ou QRS estimulado por MP ≥ 200ms. A TRC-D reduziu significativamente o desfecho combinado de morte por qualquer causa ou internação por IC. Como desfecho secundário, foram analisados separadamente morte e internações por IC, havendo redução significativa dos dois desfechos.

Análise conjunta dos resultados desses ensaios clínicos evidenciou que a TRC-D reduziu mortalidade total em comparação com CDI isolado (risco relativo 0,84; IC95% 0,73-0,96).^215^ Também foi demonstrada redução significativa de hospitalizações (RR 0,75; IC95% 0,64-0,88).

A decisão clínica sobre implante de TRC-D em pacientes com indicação de CDI deve levar em consideração o padrão de bloqueio intraventricular e a duração do complexo QRS. Resultados de metanálises apontam que os benefícios da TRC se restringem principalmente a pacientes que apresentam padrão de BRE.^216 , 217^ Em análise do estudo RAFT,^167^ o benefício sobre desfecho primário ocorreu apenas nos pacientes com QRS > 150ms. Nos pacientes com padrão de BRE, houve relação contínua entre duração do QRS e benefício clínico. Nos pacientes com padrão não BRE, o benefício ocorreu apenas com QRS > 160ms ( Tabela 18 ).

**Tabela 18:** – Recomendações para indicação de terapia de ressincronização cardíaca (TRC) em pacientes com indicação de cardioversor-desfibrilador implantável (CDI)

	Classe de recomendação	Nível de evidência
Em pacientes com indicação de CDI que apresentem BRE com QRS ≥ 150ms, FEVE reduzida e IC sintomática sob TMO	I	A
TRC para pacientes com bloqueio atrioventricular, com indicação de implante de CDI e disfunção sistólica de VE	IIa	B

*BRE: bloqueio de ramo esquerdo; CDI: cardioversor-desfibrilador implantável; FEVE: fração de ejeção do ventrículo esquerdo; IC: insuficiência cardíaca; TMO: terapia medicamentosa otimizada; TRC: terapia de ressincronização cardíaca; VE: ventrículo esquerdo.*

### 3.6. Estimulação Direta do Sistema Excito-condutor Cardíaco

A TRC é um tratamento não farmacológico bem estabelecido para tratamento de pacientes com IC sintomáticos, FEVE reduzida e QRS largo. A despeito do avanço dessa modalidade terapêutica, ainda se observa taxa de não respondedores de 20 a 40%.^218^ Neste cenário, a estimulação direta do sistema de excito-condutor cardíaco (feixe de His ou ramo esquerdo) pode ser a alternativa útil.^219 - 225^

Em 2012, Pichardo *et al* ., descreveram uma série de 16 pacientes com IC grave em que houve falha na estimulação do VE através do seio coronário. Nessa série, foi possível obter-se a correção do distúrbio de condução (BRE) com a estimulação direta do feixe de His em 81% dos casos.^226^ Lustgarten *et al* . apresentaram outro estudo que envolveu 29 pacientes, randomizados para estimulação do feixe de His ou TRC convencional. Os resultados quanto ao teste de caminhada de 6min, classe funcional, questionário de qualidade de vida e FEVE foram semelhantes entre os grupos.^227^

Em uma metanálise que envolveu 11 estudos e 494 pacientes submetidos à estimulação do feixe de His, observou-se sucesso do implante em 82,4%. Foram incluídos pacientes com FA em programação de ablação do nó AV e indicação de TRC. A estimulação hissiana demonstrou, em pequenos estudos observacionais, resultados promissores apontando a necessidade de estudos randomizados.^228^

Um recente estudo randomizado comparou a estimulação hissiana com a estimulação biventricular convencional em 41 pacientes. Dos 21 pacientes randomizados para estimulação do feixe de His, 10 (48%) necessitaram de *crossover* para o grupo convencional. O principal motivo para a troca da terapia foi a falta de correção do distúrbio de condução, mais distal, com a estimulação do feixe de His. Este achado está em linha com estudos subsequentes que demonstraram correção do bloqueio de ramo em torno de 60% dos casos com a estimulação hissiana. Não obstante, 26% dos pacientes do grupo de TRC convencional também cruzaram para o grupo da estimulação do His devido a dificuldades técnicas. Os pacientes do grupo de estimulação hissiana tiveram maior estreitamento do QRS (174 ± 18ms para 125 ± 22ms, p < 0,001 *vs.* 165 ± 17ms para 164 ± 30ms, p = 0,82) e melhora de parâmetros ecocardiográficos superior em comparação ao grupo TRC (80% dos pacientes de estimulação hissiana apresentaram aumento absoluto > 5% FEVE). As medidas de diâmetros cardíacos e volumes foram semelhantes em ambos os grupos. Estes achados sugerem que as terapias possam ser complementares, principalmente devido a dificuldades anatômicas do seio coronário e impossibilidade de correção do distúrbio da condução em todos os pacientes com BRE.^229^

Zhang *et al* . descreveram uma série de 11 pacientes consecutivos com BRE e indicação clássica de TRC, que foram submetidos à estimulação direta do ramo esquerdo. Observou-se expressivo encurtamento do QRS (129,09 ± 15,94ms em comparação ao QRS nativo de 180,00 ± 15,86ms, p < 0,01). Os autores concluíram que a estimulação septal profunda em pacientes com disfunção sistólica e BRE é factível, resultando em melhora funcional e remodelamento reverso.^230 , 231^ Huang *et al* . acompanharam uma coorte prospectiva de 63 pacientes com miocardiopatia dilatada, BRE e FEVE < 35% submetidos à estimulação do ramo esquerdo, em que foi possível corrigir o distúrbio de condução em 61 pacientes. Em 1 ano de seguimento, houve aumento significativo da FEVE, remodelamento reverso e melhora da classe funcional. Ademais, 75% dos pacientes foram classificados como hiper-respondedores, com normalização da FEVE.^232^

Wu *et al* ., em estudo não randomizado, avaliaram 137 pacientes com IC sintomática e QRS largo acompanhados por 1 ano. Nesse estudo, 49 pacientes foram submetidos á estimulação do feixe de His, 32 do ramo esquerdo, e 54 pacientes foram submetidos à estimulação biventricular. Os pacientes submetidos à estimulação do sistema excito-condutor (feixe de His ou ramo esquerdo) obtiveram maior incremento da FEVE em comparação à estimulação biventricular (23,9%, 24% e 16,7% respectivamente, p < 0,05). A melhora funcional foi semelhante nos dois grupos de estimulação do sistema excito-condutor; contudo, os limiares de estimulação foram menores com a estimulação do ramo esquerdo (0,49 *vs.* 1,35, p < 0,001).^233^

Em recente e elegante revisão sobre estimulação direta do sistema excito-condutor cardíaco, Sharma *et al* . propõem que essa modalidade terapêutica possa ser utilizada como estratégia inicial para TRC ou como terapia de resgate nos casos de insucesso da técnica convencional, por cateterizarão do seio coronário. Também consideram a associação dos métodos (estimulação simultânea His-TRC) como um novo conceito de estimulação para TRC em casos selecionados com distúrbios mais difusos da condução intraventricular^234^ ( Tabela 19 ).

**Tabela 19:** – Recomendações para indicação de estimulação direta do sistema excito-condutor cardíaco como alternativa à terapia de ressicronização cardíaca (TRC)

	Classe de recomendação	Nível de evidência
BAV e indicação de MP e FEVE < 50%, é razoável a estimulação fisiológica	IIa	B
IC sintomática, FEVE ≤ 35%, QRS ≥ 130ms é possível considerar a estimulação direta do sistema de condução como alternativa à TRC convencional	IIa	C
Em não respondedores à TRC, é razoável considerar a estimulação direta do sistema de condução como terapia de resgate	IIb	C

*BAV: bloqueio atrioventricular; FEVE: fração de ejeção do ventrículo esquerdo; IC: insuficiência cardíaca; MP: marcapa-passo; TRC: terapia de ressicronização cardíaca.*

#### 3.6.1. Modulação de Contratilidade Cardíaca

A modulação da contratilidade cardíaca (MCC) é uma opção terapêutica utilizada para IC em pacientes que não têm indicação convencional para a TRC, com QRS estreito (< 130ms) e FEVE entre 25% e 45%. Nessa modalidade de terapia, promove-se a estimulação artificial do septo interventricular direito com alta voltagem 30 a 40ms após a ativação dos cardiomiócitos durante o período refratário absoluto. Em teoria, essa estimulação otimiza a dinâmica do cálcio, aumentando a contratilidade ventricular, resultando em melhora na tolerância ao exercício e capacidade funcional dos pacientes.^235^ Não está claro ainda, entretanto, se a MCC deve ser indicada rotineiramente em pacientes que não são bons candidatos (QRS estreito) ou são não respondedores à TRC, ou, ainda, se deve ser usada em conjunto com os ressincronizadores cardíacos convencionais.^236^ Os estudos até então publicados são pequenos e, talvez por isso, incapazes para detectar diferenças significativas.^237^

## 4. Recomendações para Indicação de Cardioversor-desfibrilador Implantável

### 4.1. Prevenção Primária de Morte Súbita

#### 4.1.1. Miocardiopatia Isquêmica

A morte súbita (MS) causada por arritmias ventriculares é uma das principais causas de morte em pacientes com IC com FEVE reduzida (ICFER), em especial na população de pacientes com cardiopatia isquêmica em que a incidência de fibrose miocárdica ventricular e, consequentemente, circuitos de reentrada é mais prevalente.^238 , 239^

É importante frisar que os estudos que avaliaram o impacto do CDI na cardiopatia isquêmica definiram essa doença, de forma geral, como disfunção ventricular secundária a pelo menos uma lesão severa em uma das três principais artérias coronárias, ou história prévia documentada de IAM.^240 - 242^

Vários estudos importantes testaram o impacto do CDI na profilaxia primária para MS em pacientes com cardiopatia isquêmica. Os estudos MADIT (Multicenter Automatic Defibrillator Implantation Trial)^243^e MUSTT (Multicenter Unsustained Tachycardia Trial)^244^ testaram o CDI em pacientes com TVNS, disfunção ventricular esquerda e com indução de arritmias ventriculares sustentadas no EEF. O estudo MADIT incluiu pacientes com FEVE < 35%, classe funcional (NYHA) I, II e III, história prévia de IAM, TVNS assintomáticas registradas e arritmias ventriculares induzíveis ao EEF, refratárias ao uso de procainamida, ou antiarrítmico equivalente. Os 196 pacientes incluídos foram randomizados para implante de CDI *versus* tratamento médico otimizado (TMO), seguimento médio de 27 meses. A mortalidade no grupo CDI foi 15,7% e, no grupo TMO, 38,6%, com redução de risco relativo para mortalidade total o grupo CDI de 64% (RC 0,46; IC 95%, 0,26-0,82; p = 0,009). O estudo MUSTT incluiu pacientes com FE ≤ 40%, CF I, II e III e registro de TVNS assintomáticas. O objetivo inicial do estudo MUSTT foi comparar a eficácia de fármacos antiarrítmicos capazes de suprimir as arritmias ventriculares no EEF *versus* placebo. Devido aos resultados do MADIT, o protocolo foi modificado para implante de CDI nos casos em que havia indução de arritmias ventriculares e falha na reversão com pelo menos um antiarrítmico. O resultado do estudo MUSTT demonstrou que a diminuição na mortalidade não foi significativa com o uso de antiarrítmico, mas sim com o CDI com redução relativa de risco de morte de 76% (RC 0,24; IC95%: 0,13-0,45; p < 0,001).

Embora o estudo eletrofisiológico não seja utilizado de forma corrente para indicação de CDI devido ao baixo valor preditivo negativo, os estudos MADIT e MUSTT apresentaram resultados importantes nessa população (NNT para redução de mortalidade total = 4,2).

O estudo MADIT II randomizou pacientes com FE ≤ 30%, CF I, II e III e IAM há mais de 30 dias, para TMO *vs* CDI. Foram randomizados 1.232 pacientes e, em um seguimento médio de 20 meses, a mortalidade no grupo CDI foi 19% e, no grupo TMO, 24%, com redução relativa de risco de mortalidade total de 31% (RC 0,69; IC95% 0,51-0,93; p = 0,016).^245^ O estudo SCD-HeFT ampliou os critérios de inclusão, randomizando pacientes com FE ≤ 35%, CF II e III, isquêmicos e não isquêmicos. Durante seguimento médio de 45,5 meses, o grupo CDI teve redução relativa da mortalidade de 23% (RC 0,77; IC95% 0,62-0,96; p = 0,007), sendo que 52% da população incluída foi de pacientes com cardiopatia isquêmica.^246^

Por outro lado, os estudos que testaram o implante precoce do CDI após revascularização ou eventos isquêmicos miocárdicos foram neutros e até mesmo negativos em alguns desfechos secundários. O estudo CABG (Coronary Artery Bypass Graft) Patch randomizou 900 pacientes para implante de CDI profilático no intraoperatório de cirurgia de revascularização miocárdica, em pacientes com < 80 anos, FE < 36% e alterações no ECG de alta resolução. Após seguimento médio de 32 ± 16 meses, o estudo foi neutro em relação à mortalidade total (6,7% no grupo CDI e 4,6% no grupo controle, RC 1,07; IC95% 0,81-1,42; p = 0,64).^247^ Análise posterior demonstrou aumento da taxa de infecção no grupo do CDI (2,2% *vs.* 0,4%; p < 0,05).^246^ O estudo DINAMIT (Defibrillator after Acute Myocardial Infarction) randomizou 332 pacientes para implante de CDI e 342 para o grupo sem CDI, 6 a 40 dias após IAM.^243^ Os pacientes incluídos deveriam apresentar FE ≤ 35% e diminuição da variabilidade cardíaca ao *Holter* . Durante seguimento de 30 ± 13 meses, não houve diferença na mortalidade total entre os grupos, com 62 mortes no grupo CDI e 58 mortes no grupo controle (RC 1,08; IC95% 0,76-1,55; p = 0,66).

Desde a publicação do estudo MERIT-HF (Metoprolol CR/XL Randomized Intervention Trial in Congestive Heart Failure), sabe-se que a taxa de MS decresce de forma proporcional com a piora da CF (NYHA).^248^ Na análise de subgrupos dos estudos SCD-HeFT e MADIT II, pacientes em CF I e II foram os que mais se beneficiaram do CDI, ao passo que, em pacientes em CF III, o benefício não foi tão importante. Não há ensaios clínicos robustos que demonstrem benefício do CDI em pacientes em CF IV (apenas dados de coortes retrospectivas de pacientes em lista de transplante ou submetidos a implante de dispositivos de assistência ventricular [DAV]). Em um estudo retrospectivo que incluiu 1.089 pacientes em lista de transplante cardíaco, 550 possuíam CDI (216 para prevenção primária, 334 para prevenção secundária). Durante seguimento médio de somente 8 meses, morreram 39 pacientes (18%) do grupo CDI (profilaxia primária), 89 (27%) do grupo CDI (profilaxia secundária) e 162 (30%) do grupo sem CDI. Na análise multivariada, a presença do CDI foi preditor independente para diminuição de mortalidade (RC 0,4; IC95% 0,19-0,85; p = 0,016).^248^ O mesmo achado foi encontrado analisando a população do UNOS (United Network for Organ Sharing) em seguimento de 1999 a 2014, em que foram incluídos 32l.599 pacientes, com seguimento médio de 154 dias. Nessa população, 3.638 pacientes (11%) morreram na lista de transplante cardíaco, tendo sido de 9% a mortalidade no grupo CDI e 15% no grupo sem CDI, com redução relativa de risco de 13% (RC 0,87; IC 95% 0,80-0,94 p < 0,0001).^249^ Nesse mesmo estudo de coorte, no subgrupo de pacientes submetidos a implante de DAV (9.478 pacientes), a presença do CDI foi associada à redução de risco relativo de mortalidade de 19% (RC 0,81; IC95% 0,70 -0,94). Uma revisão sistemática analisou o CDI em pacientes com DAV, incluindo 937 pacientes (em 93% dos casos, o DAV foi utilizado como ponte para transplante). Durante seguimento médio de 7 meses, 16% dos pacientes morreram no grupo CDI e 26% no grupo sem CDI, com redução relativa de risco para morte de 39% (RC 0,61; IC95% 0,46-0,82; p < 0,01)^250^ ( Tabela 20 ).

**Tabela 20:** – Recomendações para indicação de cardioversor-desfibrilador implantável (CDI) na prevenção primária na cardiopatia isquêmica

	Classe de recomendação	Nível de evidência
CDI é recomendado em pacientes com história de IAM > 40 dias ou cardiopatia isquêmica crônica, sob tratamento farmacológico ótimo, sem isquemia miocárdica passível de tratamento por revascularização cirúrgica ou percutânea e expectativa de vida de pelo menos 1 ano e que apresentem FEVE ≤ 35% e CF II-III, ou FEVE ≤ 30% e CF I, II ou III	I	A
CDI é recomendado em pacientes com história de IAM > 40 dias ou com cardiopatia isquêmica crônica, sob tratamento farmacológico ótimo, sem isquemia miocárdica passível de tratamento por revascularização cirúrgica ou percutânea e expectativa de vida de pelo menos 1 ano, que apresentem FEVE ≤ 40%, TVNS espontânea e indução de TVS no EEF	I	B
CDI pode ser considerado em pacientes com história de IAM > 40 dias ou com cardiopatia isquêmica crônica, sob tratamento farmacológico ótimo, sem isquemia miocárdica passível de tratamento por revascularização cirúrgica, ou percutânea e candidatos à lista de transplante ou implante de DAV	IIa	B
CDI não é indicado em pacientes com IAM < 40 dias de evolução, ou indicação de revascularização	III	B
CDI não é indicado em pacientes com ICFEr CF IV refratários ao tratamento e que não sejam candidatos a transplante ou implante de DAV	III	C

*CDI: cardioversor-desfibrilador implantável; CF: classe funcional; IAM: infarto agudo do miocárdio; DAV: dispositivo de assistência ventricular; EEF: estudo eletrofisiológico; FEVE: fração de ejeção do ventrículo esquerdo; TVNS: taquicardia ventricular não sustentada; TVS taquicardia ventricular sustentada.*

#### 4.1.2. Miocardiopatia Não Isquêmica

A IC é uma condição clínica muito prevalente, com elevada morbidade e mortalidade. Em 20% a 30% dos casos, a etiologia é definida como não isquêmica, o que significa que há ausência de lesões significativas na angiografia coronariana ou resultado negativo em método de imagem para investigação de isquemia. A causa da disfunção ventricular esquerda pode ser desconhecida, sendo chamada miocardiopatia dilatada idiopática, ou pode ser atribuída a fatores como infecção viral, hipertensão arterial sistêmica, exposição a agentes potencialmente tóxicos (quimioterápicos, álcool), doença de Chagas, doenças infiltrativas, periparto, valvulopatias, doenças genéticas e autoimunes.

Mesmo que avanços na terapêutica da miocardiopatia não isquêmica (MNI) tenham trazido redução significativa da mortalidade nas últimas décadas, a morte súbita cardíaca (MSC) permanece como problema importante, sendo responsável por 30% dos óbitos.^251^As estratégias de prevenção primária de MSC nos pacientes com MNI incluem tratamento farmacológico, CDI e TRC. Ensaios clínicos randomizados demonstraram que o emprego de fármacos (betabloqueadores, sacubitril/valsartana e espironolactona) reduz significativamente as taxas de MSC neste grupo de pacientes.^252^

A estratificação de risco inclui avaliação clínica e laboratorial. Quanto pior a classe funcional (CF, NYHA), maior o risco absoluto de mortalidade geral e de MSC. A MSC é a causa de 64% dos óbitos de pacientes em CF II, 50% em CF III e 33% em CF IV (progressão da IC é a causa de 50% dos óbitos em CF IV). A ocorrência de síncope é importante fator de risco para MSC em pacientes com MNI.^253^ Outras variáveis clínicas associadas a maior risco de eventos arrítmicos nesta população são o não uso de betabloqueadores e a pressão arterial sistólica.^254 , 255^ Exames laboratoriais, como hemoglobina, ácido úrico e peptídio natriurético atrial (BNP), aparecem como preditores de mortalidade e eventos arrítmicos em alguns estudos.^256^

A redução da FEVE é considerada o principal fator de risco para MSC e mortalidade total em pacientes com IC. Poucos trabalhos, porém, avaliaram a FEVE como fator de risco para MSC em pacientes com MNI. O estudo MACAS (Marburg Cardiomyopathy Study), coorte prospectiva com 343 pacientes com MNI, demonstrou que, para cada 10% de redução da FEVE, houve risco relativo de 2,28 para eventos arrítmicos maiores (pacientes em ritmo sinusal).

A prevalência de QRS largo em pacientes com IC varia de 20% a 50%, estando associado ao aumento de MSC e mortalidade total; entretanto, nos estudos de coorte específicos de pacientes com MNI, não foi demonstrada relação significativa entre prolongamento do QRS e aumento do risco de MSC.^257 , 258^

O *Holter* pode ser útil na avaliação de risco por meio da análise de presença de taquicardia ventricular não sustentada (TVNS) e medidas da atividade autonômica (variabilidade da frequência cardíaca [VFC] e turbulência da frequência cardíaca [TFC]). A incidência de TVNS em pacientes com MNI varia de 30% a 79% e sua utilização na estratificação de risco de eventos arrítmicos é controversa.^259^

Dados de metanálise indicam que variáveis derivadas do teste ergoespirométrico como consumo de oxigênio (VO_2_), inclinação do equivalente ventilatório de CO_2_ (VE/VCO_2_ *slope* ) e presença de ventilação periódica indicam de modo independente aumento do risco de eventos combinados (incluindo mortalidade total, mortalidade cardíaca, transplante cardíaco, hospitalização e necessidade de dispositivo de assistência ventricular).^260 , 261^

Na cardiopatia isquêmica, o estudo eletrofisiológico (EEF) com estimulação ventricular programada mostrou-se capaz de identificar pacientes com risco de eventos arrítmicos graves.^246 , 248^Por outro lado, na MNI, os resultados são controversos e as diretrizes geralmente não recomendam a realização rotineira do EEF para estratificação de risco nessa população.

Há vários estudos avaliando a associação entre mutações genéticas com a fisiopatologia e o prognóstico de pacientes com MNI, particularmente naqueles com doença familiar.^262^ Entre as condições melhor investigadas, estão as mutações do gene da lâmina A/C (LMNA). Tais mutações são encontradas em 6% a 8% dos casos de MNI, podendo chegar a 30% nos casos de associação com doença do sistema de condução e envolvimento da musculatura esquelética.

A fibrose miocárdica, importante substrato arritmogênico, está presente em cerca de 44% dos pacientes com MNI, de acordo com recente metanálise que incluiu 34 estudos e 4.554 pacientes.^263^Esses pacientes apresentam maior mortalidade, arritmias ventriculares e hospitalizações por IC.^264^ Para cada percentual de aumento no volume de realce tardio, há um aumento estimado de risco para mortalidade ou eventos arrítmicos de 3% a 20% (realce tardio de 8%: razão de chances em análise univariada de 8,23-2,84-23,8). Gulati et al.^265^ publicaram os resultados da maior coorte de pacientes com MNI submetidos à RM, em que o desfecho combinado MSC e PCR recuperada ocorreu em 29,6% dos pacientes com fibrose miocárdica e em 7,0% dos pacientes sem fibrose. Para esse desfecho, a presença de fibrose apresentou *hazard ratio* de 4,61 (IC95% 2,75-7,74; p < 0,001) e a extensão da fibrose um *hazard ratio* de 1,10 (IC95% 1,05-1,16; p < 0,001). Esses resultados indicam que a RM pode ser útil na estratificação de risco de pacientes com MNI.

Diversos estudos avaliaram o impacto do CDI em pacientes com MNI. O maior deles foi o SCD-HeFT (Sudden Cardiac Death in Heart Failure Trial),^245^que incluiu pacientes com cardiopatia isquêmica e não isquêmica, com IC NYHA II-III. No seguimento dos pacientes que implantaram CDI, 33,2% receberam algum choque, 22,4% receberam choque apropriado e 10,7% receberam apenas choques inapropriados.^266^No DANISH Trial, estudo randomizado com pacientes com MNI que tinha como desfecho primário mortalidade por qualquer causa, 556 receberam CDI e 560 receberam somente tratamento clínico otimizado.^267^ Após mediana de seguimento de 67,6 meses, o desfecho primário ocorreu em 21,6% dos pacientes no grupo CDI, e 23,4% no grupo controle, sem diferença significativa (p = 0,28).

As recomendações atuais para implante de CDI na MNI estão listadas na Tabela 21 .

**Tabela 21:** – Recomendações para indicação de cardioversor-desfibrilador implantável (CDI) na prevenção primária na cardiopatia não isquêmica

	Classe de recomendação	Nível de evidência
Paciente com FEVE ≤ 35%, tratamento clínico otimizado, CF NYHA II-III e expectativa de vida >1 ano	I	B
Deve ser considerado na presença de alterações genéticas de alto risco (especialmente Lamina A/C) associado a dois ou mais fatores: FEVE ≤ 45%, TVNS, mutação de alto risco e sexo masculino	IIa	B
IC CF NYHA IV refratária sem perspectiva de transplante ou DAC	III	C

*CDI: cardioversor-desfibrilador implantável; CF: classe funcional; DAC: doença arterial coronária; FEVE: fração de ejeção do ventrículo esquerdo; IC: insuficiência cardíaca; NYHA: New York Heart Association; TVNS: taquicardia ventricular não sustentada.*

#### 4.1.3. Cardiomiopatia Hipertrófica

A cardiomiopatia hipertrófica (CMH) é uma doença genética causada por uma mutação autossômica dominante em genes que codificam as proteínas dos sarcômeros, com prevalência em torno de 1:500 indivíduos.^268 , 269^ É caracterizada pela presença de graus variados de hipertrofia ventricular esquerda assimétrica, na ausência de condições que possam resultar em sobrecarga que expliquem as alterações, podendo ocasionar IC diastólica, obstrução da via de saída do VE, arritmias atriais e ventriculares e, em alguns casos, a morte súbita cardíaca (MSC).^270 , 271^ A maioria dos pacientes não apresenta qualquer sintoma, e a MSC, não raramente, é a primeira manifestação da doença.^272 - 276^

Teare publicou, em 1958, os primeiros relatos de uma série de oito pacientes com hipertrofia miocárdica assimétrica (na época, ainda sem nomenclatura bem-definida) e considerou a possibilidade diagnóstica de hamartoma muscular, tendo correlacionado os achados anatômicos com maior ocorrência de MSC em adultos jovens. O aspecto anatomopatológico era de desarranjo grosseiro dos feixes musculares com hipertrofia da fibra muscular individualmente e dos seus núcleos.^277^

Os pacientes com diagnóstico de CMH apresentam aproximadamente 1% de risco anual para MSC; porém, alguns pacientes podem ter esse risco bem maior de acordo com determinadas características de risco.^278^ Na era “pré-CDI”, a taxa de mortalidade girava em torno de 1,5% ao ano; com a introdução do CDI, essa taxa tem sido reduzida para 0,5% ao ano.^279 , 280^

Maron *et al.* publicaram, em 2019, um estudo longitudinal e unicêntrico, envolvendo uma grande coorte de 2.094 pacientes com CMH com seguimento de 17 anos. Dentre 527 pacientes que tinham pelo menos um fator de risco convencional e que receberam CDI para prevenção primária, 15,6% apresentaram terapias apropriadas (TV/FV), correspondendo a quase 50 vezes o número de eventos em comparação com o grupo sem CDI.^281^

A CMH é a causa mais comum de MSC em indivíduos < 40 anos de idade, e a maioria dos episódios é devido à fibrilação ventricular; dessa forma, a abordagem mais efetiva para reduzir a mortalidade de pacientes de alto risco é com o implante de CDI, que não é isento de complicações e pode ocasionar desconforto, estresse psicológico e tem custos elevados.^282 , 283^ A seleção de pacientes pode ser difícil em virtude de características individuais, manifestações clínicas, história famíliar e definições dos fatores de risco, além de a MSC ser um achado infrequente na prática clínica.^284 , 285^ A maior probabilidade de benefício do CDI é baseada em testes não invasivos incluindo a história clínica, ECG, teste de esforço, *Holter* , ecocardiograma e ressonância magnética do coração (RMC). Os fatores de risco de MSC convencionais são: história familiar de MSC relacionada à CMH, síncope inexplicada ocorrendo em até 6 meses da avaliação, taquicardia ventricular não sustentada (TVNS), espessura septal ≥ 30mm e modificadores de risco incluindo resposta hipotensora ao teste de esforço, fibrose do VE e IC com FEVE < 50%.^286^ Recomenda-se que a estratificação de risco para indicação de CDI deve ser realizada periodicamente a cada 1 ou 2 anos em pacientes com CMH.^287^

A indicação de CDI na CMH não está embasada em estudos clínicos randomizados, mas, sim, em dados de estudos observacionais. Adicionalmente, estudos com pacientes com CMH portadores de CDI demonstraram que a taxa de eventos potencialmente fatais, com terapias apropriadas do dispositivo, ocorrem em 12%/ano na prevenção secundária e em 4%/ano na prevenção primária.^288^ Neste contexto, a probabilidade de ocorrência de terapias apropriadas parece ser similar em pacientes com 1, 2, 3 ou mais fatores de risco convencionais (prevenção primária), levando à conclusão de que a presença de um único marcador pode justificar o implante do CDI. Dentre os fatores de risco convencionais, a história familiar de MSC, definitivamente, ou provavelmente, relacionada a CMH, em parentes de primeiro grau com idade ≤ 50 anos, principalmente na infância e adolescência, tem importância bastante significativa.^289^ Outro marcador de risco de MSC consiste na extensão e na magnitude da hipertrofia, sendo mais importante quando ≥ 30mm; espessamento limítrofe (28 a 29mm) pode ser considerado a critério do cardiologista. Spirito *et al.* demonstraram, em 480 pacientes, que a incidência de MSC foi aproximadamente duas vezes maior a cada aumento de 5mm na espessura miocárdica ventricular, sendo de 1,8%/ano em casos com espessura ≥ 30mm.^290^

A presença de síncope inexplicada, sendo improvável ou afastada a possibilidade de síncope vasovagal e não relacionada à obstrução da via de saída do VE, foi fortemente associada ao risco de MSC em um estudo com pacientes com CMH, principalmente se ocorreu até 6 meses da avaliação inicial, com o risco 5 vezes maior do que aqueles sem síncope. Episódios remotos (superior a 5 anos da avaliação inicial) não se correlacionaram a aumento no risco de MSC.^291^

A TVNS é definida com a presença de 3 ou mais episódios com 3 ou mais batimentos ventriculares repetitivos e/ou 1 ou mais episódios prolongados com 10 ou mais batimentos a 130bpm ou mais, detectados em *Holter* de 24 a 48 horas. A incidência de TVNS tem sido relatada em 20% a 46% de pacientes com CMH. Episódios de taquicardia ventricular (TV) estão claramente associados a MSC em pacientes com CMH; entretanto, os dados são menos robustos em demonstrar que a presença isolada de TVNS seja um fator de risco independente. Por outro lado, o risco aumenta na presença de modificadores de risco, especialmente fibrose do VE.^292 , 293^

O aconselhamento genético é importante em pacientes com CMH. A identificação do portador de mutação genética específica pode auxiliar na investigação da doença em parentes próximos. Indivíduos com genes positivos podem desenvolver CMH; por isso, devem ser acompanhados com atenção para a presença de características da doença ou fatores de risco ao longo do tempo.^294^

Existe evidência cada vez mais forte da relação entre fibrose miocárdica (RMC) e risco de MSC, sendo considerado modificador de risco.^295 - 297^Em estudo brasileiro envolvendo pacientes com CMH de alto risco portadores de CDI, Shiozaki *et al.* demonstraram que a presença de fibrose miocárdica foi identificada em 96,4% desses pacientes, com taxa de fibrose média de 15,96%, sugerindo que esse fator possa ter maior sensibilidade em relação aos demais marcadores de risco convencionais.^298^ Chan RH *et al.* demonstraram na CMH que a detecção de área de fibrose maior que 15% da massa ventricular esquerda se associou ao dobro do risco de MSC em pacientes considerados inicialmente de baixo risco.^299^

Klopotowski *et al.* avaliaram prospectivamente 328 pacientes com CMH submetidos à RMC com o objetivo de avaliar a importância da localização da fibrose como ferramenta auxiliar na estratificação de risco de MSC. Áreas de realce tardio indicando a presença de fibrose além da região do septo interventricular em paciente com CMH foram associadas a maior risco de MSC ou equivalente, como TV instável ou terapia apropriada do CDI. Esse estudo sugere, considerando a calculadora de risco elaborada pela Sociedade Europeia de Cardiologia (ESC), que, em pacientes classificados de risco intermediário, a presença de fibrose além da região do septo interventricular pode identificar o paciente com maior benefício ao uso do CDI, favorecendo a indicação do dispositivo.^300 - 302^

O uso da calculadora de escore de risco na CMH tem sido incentivado pela ESC, mas tem baixa sensibilidade para a decisão de implante de CDI em pacientes de alto risco. A estratégia da sociedade americana de cardiologia de analisar fatores de risco individualmente ou associado a modificadores de risco em cada paciente com CMH apresenta sensibilidade de 95% em predizer eventos de TV potencialmente fatais, sendo superior ao modelo matemático do escore de risco da ESC que apresenta sensibilidade em torno de 34%. Por outro lado, a calculadora da ESC apresenta maior sensibilidade em identificar os pacientes verdadeiramente de baixo risco, com menor probabilidade de eventos (em torno de 92% comparado a 78% da sociedade americana), evitando implantes de CDI desnecessários.

Resposta anormal ou hipotensão ao exercício ocorre em 1 a cada 3 pacientes com CMH. O mecanismo reflete queda exacerbada na resistência vascular sistêmica devido à disfunção autonômica e/ou obstrução dinâmica da via de saída do VE. Em pacientes jovens, a resposta anormal da PA está associada a aumento do risco de MSC.^303^

Aneurisma apical de VE identificado por ecocardiografia ou RMC, independentemente do tamanho, pode estar associado a maior risco de TV sustentada monomórfica.^304^ Rowin *et al.* avaliaram, retrospectivamente, 1.940 pacientes com CMH e identificaram aneurisma de VE em 93 pacientes (4,8%) com taxa de eventos adversos de 6,4%/ano, correspondendo a 3 vezes mais que nos pacientes sem aneurisma, incluindo MSC, terapia apropriada do CDI, eventos tromboembólicos e evolução terminal de IC com FEVE < 50%. O CDI foi indicado como prevenção primária em 54 destes pacientes, considerando o aneurisma isolado como fator de risco em 19 casos, ocorrendo terapia apropriada do CDI para TV/FV em 20%. A taxa de eventos arrítmicos foi de aproximadamente 5%/ano, sendo mais que 5 vezes a taxa de ocorrência em pacientes sem aneurisma, sugerindo equivalência a outros marcadores de risco convencionais nos pacientes com CMH de alto risco.^305^

O CDI subcutâneo tem vantagens potenciais especialmente em jovens, uma vez que preserva o sistema venoso e evita complicações crônicas dos cabos-eletrodos (desde que não seja necessário estimulação ventricular). Por outro lado, a eficácia do CDI subcutâneo em abortar a FV em pacientes com CMH ainda permanece incerta.^306^

**Figura 2:**
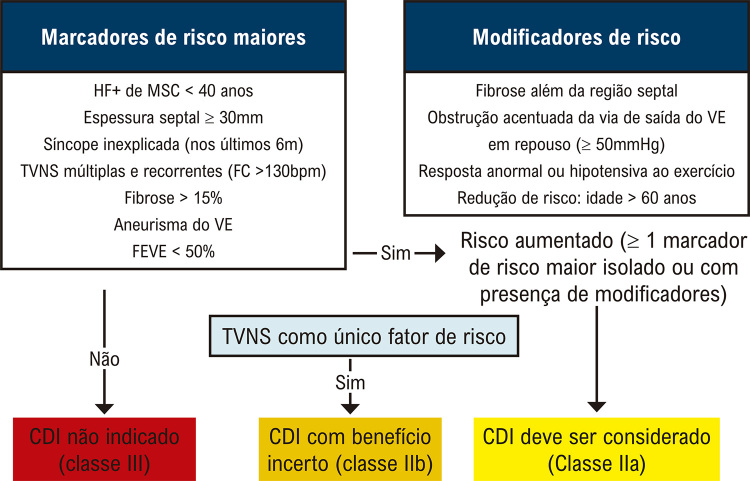
– Algoritmo de prevenção primária da MSC no portador de CMH. fração de ejeção do ventrículo esquerdo; HF+: história familiar positiva; m: meses; MSC: morte súbita cardíaca; PA: pressão arterial; TVNS: taquicardia ventricular não sustentada; VE: ventrículo esquerdo.

Por fim, as pesquisas que avaliaram o papel do estudo eletrofisiológico (EEF) na estratificação de risco para MSC em pacientes com CMH não observaram benefício nessa estratégia; com isso, não deve ser indicado com essa finalidade^307^ ( Figura 2 e Tabela 22 ).

**Tabela 22:** – Recomendações para indicação de cardioversor-desfibrilador implantável (CDI) na prevenção primária na cardiomiopatia hipertrófica

	Classe de recomendação	Nível de evidência
Pacientes com pelo menos 1 fator de risco maior	IIa	B
Pacientes com TVNS ou hipotensão arterial anormal no esforço, na presença de fatores de risco adicionais é razoável indicar CDI desde que expectativa de vida >1 ano	IIa	B
Estratificação de risco invasiva com EEF	III	B

*Fatores de risco: Espessura de parede > 30mm; História familiar de MS; TVNS; presença de realce tardio na RM; síncope há menos de 5 anos; aneurisma de VE; FEVE <50%. CDI: cardioversor-desfibrilador implantável; EEF: estudo eletrofisiológico; TVNS: taquicardia ventricular não sustentada.*

#### 4.1.4. Cardiomiopatia Chagásica

A doença de Chagas (DCh) é causada pelo protozoário parasita *Trypanosoma cruzi* , transmitido aos seres humanos pelas fezes de um inseto hematófago, da família *Triatominae* , na maioria dos casos.^308^ Geralmente, a infecção ocorre na infância e a fase aguda tem período de incubação de 1 a 2 semanas, podendo durar até 3 meses. Segue-se a fase crônica, na qual, por muito tempo (2 a 4 décadas, na maioria das vezes), os pacientes apresentam apenas sorologia positiva, sem sintomas ou outros sinais de doença clinicamente aparente.^309 , 310^Tais pacientes apresentam, portanto, a chamada forma indeterminada da DCh, cujo prognóstico é essencialmente benigno.^311 , 312^Enquanto, por mecanismos patogenéticos ainda incompletamente entendidos,^313^ muitos pacientes permanecem por toda a vida com essa forma da doença, cerca de 30% a 50% dos indivíduos infectados evoluem para as formas determinadas: cardíaca, digestiva ou mista.

A cardiopatia chagásica crônica (CCC) tem peculiaridades fisiopatológicas muito acentuadas e constitui a forma clínica mais comum e mais grave da DCh, sendo responsável por expressiva morbimortalidade na América Latina e em países com expressiva imigração.^314^

Estima-se que 8 a 10 milhões de pessoas estejam infectadas pelo *Trypanosoma cruzi* na na América Latina e em outros países.^315 - 317^ Considerando o pior cenário, com base nas estimativas anteriores, pode-se deduzir que 3 a 5 milhões de indivíduos infectados manifestarão formas clínicas da doença em sua fase crônica.

A taxa de mortalidade anual média atribuída à CCC é estimada em 4%, podendo variar de 1% a 10% conforme estratificação de risco embasada em características clínicas e exames cardiológicos simples.^318^

Além dos critérios empregados na estratificação de risco, diversos marcadores de pior prognóstico têm sido identificados por vários autores, especialmente no que se refere à MSC em diversos contextos clínicos.^319 - 338^Características como pré-síncope e síncope, disfunção ventricular esquerda e IC, taquicardia ventricular sustentada (TVS) ou não sustentada (TVNS), bradiarritmia grave (DNS e BAV) e parada cardíaca recuperada foram identificadas como marcadores de risco de MSC. Por outro lado, extrassístoles ventriculares isoladas ( *Holter* ) e bloqueio de ramo direito não interferem significativamente no prognóstico da CCC.

A MSC, responsável por aproximadamente 55% a 65% de todas as causas de óbitos, frequentemente se associa a manifestações de IC, mas pode também ocorrer em pacientes com disfunção de VE assintomática.^339 - 341^ A IC refratária é causa de morte em cerca de 25% a 30% dos pacientes. A correlação entre os estágios da CCC e causas de mortalidade foi descrita recentemente: a MSC é mais prevalente no estágio III da doença, enquanto prevalência de morte por IC aumenta progressivamente do estágio I a IV.

O principal mecanismo de morte súbita na CCC é arritmogênico, sendo que a TVS com fibrilação ventricular (FV) subsequente é responsável pela imensa maioria dos eventos letais.^342^ Nesse sentido, as anormalidades estruturais da CCC (com inflamação, morte celular e fibrose reativa e reparativa) constituem o substrato anatômico ideal porque promovem bloqueios unidirecionais e áreas de condução lenta propícias para desencadeamento de reentrada elétrica. Os disparadores que incidem sobre esse substrato anatômico, as extrassístoles ventriculares, também invariavelmente presentes, completam os elementos essenciais para a instalação da taquiarritmia ventricular por reentrada.^314 , 329 , 331 , 333 , 336 , 338 , 345^ Assim, TVNS pode ocorrer em cerca de 40% dos pacientes com CCC e alterações regionais da mobilidade segmentar, e em praticamente todos os pacientes com disfunção sistólica global de VE e IC.^343^ A TVS, de prognóstico mais ominoso, ocorre espontaneamente e pode ser reproduzida em cerca de 80% a 85% dos pacientes durante estudo eletrofisiológico.^319 , 328 , 329^

O BAV total é outra causa, mas menos comum, de MSC na CCC, como consequência da degeneração necrótica e fibrose difusa, predominantemente na região atrioventricular.^330^

Como já referido, a MSC também pode ser resultante de tromboembolismo pulmonar maciço ou de tromboembolismo sistêmico em órgãos vitais. Excepcionalmente, a MSC pode ocorrer em consequência de rompimento de aneurisma apical de VE.

Rassi *et al.* desenvolveram um escore para estratificação de risco para mortalidade de pacientes com CCC, com base em variáveis clínicas e exames cardiológicos básicos.^322^Este escore também foi aplicado por outros investigadores^344^ em coorte retrospectiva (149 pacientes) que também propuseram que a presença de TV (teste ergométrico ou *Holter* ), FEVE < 0,50 e QRS > 150ms (ECGAR) possa identificar pacientes com CCC e risco de morte em 5 anos. A ausência ou presença de um fator caracterizaria grupo de baixo risco; risco intermediário quando ocorrem dois fatores; e alto risco quando há presença dos três fatores.

A prevenção primária de MSC em pacientes com CCC inclui, em tese, o uso de amiodarona ou CDI.^345^ Nao há, entretanto, evidência científica que sustente a indicação de CDI na prevenção primária de MSC na CCC. Há muitas particularidades patogenéticas e fisiopatológicas que dificultam qualquer comparação direta com os resultados da literatura em outras cardiopatias.^317^A peculiaridade mais marcante é a de que muitos pacientes com CCC, mesmo com função de VE preservada, já possuírem substrato para arritmias potencialmente letais^331 , 345 - 348^( Tabela 23 ).

**Tabela 23:** – Recomendações para indicação de cardioversor-desfibrilador implantável (CDI) na prevenção primária na cardiopatia chagásica crônica

	Classe de recomendação	Nível de evidência
TVS estável com FEVE <35% em tratamento clínico otimizado	I	C
TVS estável com FEVE >35% em tratamento clínico otimizado	IIa	C
TVNS; FEVE <35% em tratamento clínico otimizado	IIb	C
Pacientes em CF NYHA IV refratária não candidatos a transplante cardíaco	III	C

*CDI: cardioversor-desfibrilador implantável; CF: classe funcional; FEVE: fração de ejeção do ventrículo esquerdo; NYHA: New York Heart Association; TVS: taquicardia ventricular sustentada; TVNS: taquicardia ventricular não sustentada.*

O estudo CHAGASICS, em andamento, é um ensaio clínico randomizado, multicêntrico, nacional e aberto, que tem como objetivo comparar a eficácia do CDI *versus* amiodarona na prevenção primária de morte por todas as causas, em pacientes com CCC e TVNS, estratificados pelo escore de Rassi.^322^O CHAGASICS deverá ser a evidência científica necessária para definir os critérios para uso da amiodarona e/ou indicação de CDI em pacientes com CCC que não apresentaram desfechos clínicos potencialmente fatais.

#### 4.1.5. Cardiomiopatia Arritmogênica do Ventrículo Direito

A cardiomiopatia arritmogênica do VD (CAVD) é uma cardiomiopatia de herança autossômica dominante e penetrância variável, que provoca mutação de genes que codificam proteínas de adesão celular, os desmossomos. A CAVD afeta predominantemente o VD, mas pode afetar o VE em cerca de 0,5% dos casos, determinando substituição do tecido miocárdico por fibrose e tecido adiposo. Tais alterações estruturais frequentemente causam arritmias ventriculares e morte súbita cardíaca (MSC).^346 , 347^A prevalência estimada da CAVD varia de 1:1.000 a 1:5.000 pessoas na população geral, e é importante causa de MSC em atletas e adultos jovens.^348^ Arritmias ventriculares, síncope e MSC ocorrem particularmente na 2ª e 3ª décadas de vida, geralmente durante atividade física. Síncope é relatada em 16% a 39% dos pacientes com CAVD no momento do diagnóstico, está frequentemente relacionada à atividade física e tem sido associada a maior risco de arritmias.^349^

Extrassístoles ventriculares frequentes, taquicardia ventricular não sustentada (TVNS) e taquicardia ventricular sustentada (TVS) são preditores importantes de eventos cardíacos. A ocorrência de TVS é preditora importante de MSC e de terapias apropriadas do CDI. A MSC pode ser a primeira manifestação da CAVD.^350 , 351^

As arritmias ventriculares têm origem geralmente no VD (morfologia de BRE), mas o eixo do QRS durante a TVS geralmente difere da via de saída do VD (VSVD); muitos pacientes podem ter QRS de múltiplas morfologias.^352^

As regiões de tecido fibrogorduroso criam áreas de ativação ventricular retardada, causando deflexões fracionadas no final do complexo QRS (ondas *épsilon* ) e potenciais tardios ao ECGAR. Em pacientes com suspeita de CAVD, o ECGAR pode ser útil para diagnóstico e estratificação de risco (Classe IIa, nível de evidência B). Achados anormais ao ECGAR estão relacionados com a GRAVIDADE da CAVD na RMC e ocorrência de eventos adversos.^353^

A RMC fornece informações quanto à função ventricular, tamanho das cavidades cardíacas, anormalidades segmentares e extensão da fibrose com a técnica de realce tardio com gadolíneo. Com esta técnica, foi demonstrado acometimento biventricular em 34% a 56% dos pacientes e acometimento isolado do VE em 4% a 9%. Áreas de fibrose pela RMC têm relação com a localização do substrato da arritmia ventricular identificado pelo mapeamento eletrofisiológico endocárdico e epicárdico.^354^

O estudo eletrofisiológico (EEF) tem valor incerto na CAVD assintomática como preditor do risco de MSC (Classe IIb, nível de evidência B). Em pacientes com CDI para prevenção primária, a indução de TVS não é preditor de choques apropriados.^355^Na Tabela 24 , estão listados os critérios para diagnóstico da CAVD.^355^

**Tabela 24:** – Critérios revisados para diagnóstico da CAVD

I. Disfunção regional ou global e alterações estruturais	
Critério Maior	Ecocardiograma
	Acinesia segmentar de VD, discinesia ou aneurisma e um dos seguintes (final da diástole):
	VSVD no ELPE ≥ 32mm (≥ 19mm/m^2^ corrigido para ASC)
	VSVD no ECPE ≥ 36mm (≥ 21mm/m^2^ corrigido para ASC)
	Mudança na área fraccional ≤ 33%
	Ressonância magnética cardíaca
	Acinesia segmentar de VD ou discinesia ou contração dissincrônica do VD e um dos seguintes:
	Razão do volume diastólico final do VD pela ASC ≥ 110mL/m^2^ (homens) ou ≥ 100mL/m^2^ (mulheres)
	Fração de ejeção do VD ≤ 40%
	Angiografia do VD
	Acinesia segmentar de VD, discinesia ou aneurisma
Critério Menor	Ecocardiograma
	Acinesia segmentar de VD ou discinesia e um dos seguintes (final da diástole):
	VSVD no ELPE ≥ 29 a < 32mm (≥ 16 a < 19mm/m^2^ corrigido para ASC)
	VSVD no ECPE ≥ 32 a < 36mm (≥ 18 a < 21mm/m^2^ corrigido para ASC)
	Mudança na área fraccional > 33% a ≤ 40%
	RMC
	Acinesia segmentar de VD ou discinesia ou contração dissincrônica do VD e um dos seguintes:
	Razão do volume diastólico final do VD pela ASC ≥ 100 a < 110mL/m^2^ (homens) ou ≥ 90 a < 100 mL/m^2^ (mulheres)
	Fração de ejeção do VD >40% a ≤45%
**II. Caracterização do tecido da parede**	
Critério maior	Menos que 60% de miócitos residuais pela análise morfométrica (ou < 50% se estimado), com substituição fibrosa da parede livre do VD em mais de uma amostra, com ou sem substituição gordurosa na biópsia endomiocárdica
Critério menor	60% a 75% de miócitos residuais pela análise morfométrica (ou 50% a 65% se estimado), com substituição fibrosa da parede livre do VD em mais de uma amostra, com ou sem substituição gordurosa na biópsia endomiocárdica
**III. Alterações na repolarização**	
Critério maior	Ondas T invertidas nas derivações precordiais (V1, V2 e V3) ou além em indivíduos > 14 anos (na ausência de BRD completo QRS ≥ 20ms)
Critério menor	Ondas T invertidas nas derivações V1 e V2 em indivíduos > 14 anos (na ausência de BRD completo) ou em V4, V5 e V6
	Ondas T invertidas nas derivações V1, V2, V3 e V4 em indivíduos > 14 anos na presença de BRD completo
**IV. Alterações na despolarização/condução**	
Critério maior	Onda épsilon (sinais de baixa amplitude entre o final do QRS e o início da onda T) nas derivações V1 a V3
Critério menor	Potenciais tardios no ECGAR em ≥ 1 dos 3 parâmetros na ausência de duração QRS ≥ 110ms no ECG
	Duração do QRS filtrado ≥ 114ms
	Duração do QRS terminal <40 µV (duração do sinal de baixa amplitude) ≥ 38ms
	Voltagem da raiz quadrada média dos 40ms terminais ≤ 20 µV
	Duração da ativação terminal do QRS ≥ 55ms medido do nadir da onda S ao final do QRS, incluindo R', nas derivações V1, V2 ou V3 na ausência de BRD completo
**V. Arritmias**	
Critério maior	TVNS ou TVS com morfologia de BRE e eixo superior (QRS negativo ou indeterminado nas derivações DII, DIII e aVF e positivo em aVL)
Critério Menor	TVNS ou TVS da VSVD, morfologia de BRE e eixo inferior (QRS positivo nas derivações DII, DIII e aVF e negativo em aVL) ou eixo desconhecido
	> 500 extrassístoles ventriculares em 24 horas (Holter)
**VI. História familiar**	
Critério maior	CAVD confirmada em parente de primeiro grau diagnosticado conforme esses critérios
	CAVD confirmada em necrópsia ou cirurgia em parente de primeiro grau
	Identificação de mutação patogênica categorizada como associada ou provavelmente associada à CAVD
Critério menor	História de CAVD em parente de primeiro grau, o qual não é possível determinar se este familiar preenche estes critérios diagnósticos
	Morte súbita em em parente de primeiro grau < 35 anos com suspeita de CAVD
	CAVD confirmada patologicamente por estes critérios em parente de segundo grau

*ASC: área de superfície corpórea; BRD: bloqueio de ramo direito; BRE: bloqueio de ramo esquerdo; CAVD: cardiomiopatia arritmogênica do ventrículo direito; ECG: eletrocardiograma; ECGAR: eletrocardiograma de alta resolução; ECPE: eixo curto para-esternal; ELPE: eixo longo para-esternal; RMC: ressonância magnética do coração; TVNS: taquicardia ventricular não sustentada; TVS: taquicardia ventricular sustentada; VD: ventrículo direito; VSVD: via de saída do ventrículo direito.*

Os testes genéticos realizados em probandos com suspeita de CAVD são positivos em 30% a 54%. Importante ressaltar que um teste negativo não exclui a doença, e um teste positivo não define a terapêutica. A CAVD é detectada em aproximadamente 35% a 40% dos parentes de primeiro grau, e o rastreamento clínico com ECG, Holter, teste ergométrico e exames de imagem cardíaca pode identificar familiares sob risco de CAVD.^356^

Pacientes assintomáticos e sem arritmias ventriculares devem receber apenas betabloqueadores e avaliados periodicamente quanto a função ventricular e ocorrência de arritmias.^357^Não há estudos randomizados avaliando a melhor opção de antiarrítmicos para tratamento da TVS. Um estudo observacional demonstrou supressão de TV induzida em 58% dos pacientes e apenas 10% apresentaram recorrência da arritmia com o uso de sotalol.^358^ Em outro registro observacional, betabloqueadores ou sotalol não estiveram associados à redução de arritmias ventriculares; amiodarona foi superior para sua prevenção em pequena coorte.^359^Ablação de TVS reduz a sua recorrência, mas não elimina a necessidade de implante de CDI.^360^

Pacientes com história de MSC abortada, TVS mal tolerada e síncope têm maior risco de MSC, com taxa anual >10%, sendo indicado implante de CDI. Fatores de risco para MSC ou choque apropriado reportados em diferentes coortes são: TVS, síncope inexplicada, TVNS frequente, história familiar de MSC precoce, comprometimento extenso do VD, QRS muito prolongado, realce tardio na RMC, disfunção de VE e indução de TVS no EEF.^361^Revisão sistemática recente incluindo 610 pacientes seguidos por 3,8 anos (média) demonstrou taxa anual de 9,5% e 3,7% de choques apropriados e inapropriados, respectivamente.^362 , 363^

**Figura 3:**
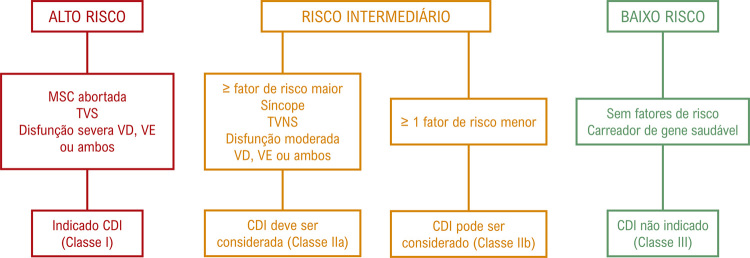
– Fluxograma de estratificação e indicação de implante de CDI na CAVD. CDI: cardioversor-desfibrilador implantável; CAVD: cardiopatia arritmogênica do ventrículo direito; MSC: morte súbita cardíaca; TVS: taquicardia ventricular sustentada; TVNS: taquicardia ventricular não sustentada; VD: ventrículo direito; VE: ventrículo esquerdo.

A indicação de CDI na CAVD para prevenção primária é medida de difícil avaliação e deve contar com uma avaliação clínica detalhada considerando história familiar, severidade da disfunção de VD e VE, complicações a longo prazo do CDI, impacto psicológico e econômico. A Tabela 25 e o Figura 3 listam as indicações de CDI na CAVD para prevenção primária.

**Tabela 25:** – Recomendações para indicação de cardioversor-desfibrilador implantável (CDI) na prevenção primária na cardiopatia arritmogênica do ventrículo direito

	Classe de recomendação	Nível de evidência
CAVD e MSC abortada ou TVS se sobrevida esperada maior que 1 ano	I	B
CAVD e disfunção importante de VD ou FEVE ≤ 35% se sobrevida esperada maior que 1 ano	I	B
CAVD e síncope presumidamente por arritmia ventricular, CDI pode ser útil se sobrevida esperada maior que 1 ano	IIa	B
CAVD e TVS bem tolerada, avaliando o benefício e risco de complicações a longo prazo do CDI	IIa	C

*CAVD: cardiopatia arritmogênica do ventrículo direito; CDI: cardioversor-desfibrilador implantável; FEVE: fração de ejeção do ventrículo esquerdo; MSC: morte súbita cardíaca; TVS: taquicardia ventricular sustentada; VD: ventrículo direito.*

Um novo modelo preditor de arritmias ventriculares na CAVD foi recentemente publicado.^364^ As variáveis preditoras foram: sexo masculino, idade, síncope nos últimos 6 meses, TVNS prévia, número de extrassístoles ventriculares no *Holter* -24h, número de derivações com onda T invertida nas derivações inferiores e anteriores e fração de ejeção do VD. Esse novo modelo determinou maior refinamento na seleção de pacientes para implante de CDI quando comparado ao Fluxograma da International Task Force, publicado em 2015, reduzindo em 20,6% a indicação de implante.^365^Os autores do novo modelo disponibilizaram uma calculadora de risco *online* (www.arvcrisk.com) que calcula o risco de arritmia ventricular em 5 anos; embora não determine o limiar de risco aceitável para implante do CDI, acredita-se que o modelo auxilia no processo de decisão para prevenção primária.

#### 4.1.6. Miocardiopatia Não Compactada

A miocardiopatia não compactada (MNC) é uma rara anormalidade congênita, caracterizada pela formação de trabeculações proeminentes e recessos intertrabeculares profundos nos ventrículos esquerdo (VE) e direito (VD), que ocorre durante a fase de embriogênese do endomiocárdio (entre a 5ª e 8ª semana de vida fetal), atingindo mais comumente o ápice do VE.^363^ O comprometimento de ambos os ventrículos ocorre em 22% a 38% dos pacientes. A não compactação do VE ocorre em associação com outras cardiopatias congênitas ou de forma isolada.^366^

A herança genética surge padrão autossômico dominante em pelo menos 30% a 50% dos pacientes; vários genes que causam não compactação do VE já foram identificados. Esses genes geralmente codificam proteínas do sarcômero (aparelho contrátil) ou citoesqueléticas.^367^

A apresentação clínica da MNC é heterogênea, variando de casos totalmente assintomáticos até casos com manifestações graves e fatais como IC, tromboembolismo, BAV e intraventricular, arritmia ventricular e morte súbita cardíaca (MSC). Idade, diâmetro diastólico final do VE, IC sintomática, FA permanente ou persistente, bloqueio de ramo e doença neuromuscular associada são preditores de maior mortalidade.^368^

O ecocardiograma (ECO) é rotineiramente realizado na investigação inicial, e o uso de contraste pode melhorar a sensibilidade do diagnóstico.^369^ A RMC possibilita a visibilização da proporção de miocárdio não compactado e compactado e permite a identificação de trombos e fibrose miocárdica.^370^

A MSC é a principal causa de morte na MNC, podendo ocorrer em qualquer idade; não existem ferramentas diagnósticas precisas para a estratificação de risco nesses pacientes. Arritmias ventriculares são relatadas em 38% a 47% e a MSC ocorre em 13% a 18% dos pacientes.^371^ O exame histológico evidencia continuidade entre o endocárdio ventricular e os recessos intertrabeculares profundos que podem facilitar a arritmogênese pela formação de circuitos reentrantes subjacentes ao tecido cicatricial, predominando no ápice do VE e segmentos médio-apicais.^372^

Steffel *et al* . demonstraram que a indução de TV sustentada no EEF para estratificação de risco tem pouco valor na MCN; por outro lado, a não indução pode identificar pacientes de baixo risco.^373^A ablação por cateter endo e/ou epicárdica parece ser útil em portadores de CDI com arritmias ventriculares frequentes.^374 , 375^A taxa de choques apropriados nesses pacientes, na prevenção secundária, é de 33% a 37% em seguimento médio de 34 a 40 meses.^376^

Não existem dados convincentes demonstrando que a não compactação do VE, por si só, seja suficiente para indicação de implante de CDI. Tal indicação deve ser guiada pela gravidade da disfunção sistólica do VE e presença de arritmia ventricular sustentada (como na cardiomiopatia dilatada idiopática).^377 , 378^No entanto, Caliskan *et al* ., em estudo que incluiu 77 pacientes com MNC, demonstraram que, entre os pacientes que receberam CDI para prevenção secundária, a disfunção de VE ou sua dilatação não eram proeminentes, tornando esses critérios frágeis para a indicação de prevenção primária. Por outro lado, a presença de TVNS foi mais frequente na prevenção secundária em relação aos pacientes que receberam CDI para prevenção primária ou não foram submetidos ao implante de CDI. Outros fatores de risco que devem ser considerados são história familiar e ocorrência de síncope.^378^

Apesar de não haver estudos prospectivos que abordem a prevenção de MSC em MNC, existem dados de estudos observacionais suficientes que suportam a indicação do CDI como estratégia razoável para prevenção de MSC nesses pacientes^379^( Tabela 26 ).

**Tabela 26:** – Recomendações para indicação de cardioversor-desfibrilador implantável (CDI) na Prevenção Primária na Miocardiopatia não compactada

	Classe de recomendação	Nível de evidência
Pacientes com MNC que apresentem FEVE ≤35%, classe funcional de NYHA II-III e expectativa de vida de pelo menos 1 ano devem receber o CDI como estratégia de prevenção da MSC, baseado em recomendações para CMD	I	B
Pacientes com diagnóstico de MNC com função ventricular normal com fatores de risco incluindo: TVNS, história familiar de MSC e síncope podem receber o CDI como estratégia de prevenção da MSC	IIb	C
Pacientes com MNC com função ventricular normal e sem fatores de risco não devem ser considerados candidatos a implante de CDI	III	C
Realização de EEF para estratificação de risco	III	C

*CDI: cardioversor-desfibrilador implantável; CMD: cardiomiopatia dilatada; EEF: estudo eletrofisiológico; FEVE: fração de ejeção do ventrículo esquerdo; MNC: miocárdio não compactado; NYHA: New York Heart Association; TVNS: taquicardia ventricular não sustetantada.*

#### 4.1.7. Síndrome do QT Longo e Síndrome do QT Curto Congênito

A síndrome do QT longo congênito é caracterizada por prolongamento do intervalo QT e arritmias ventriculares polimórficas frequentemente deflagradas por estímulo adrenérgico.^380^

Centenas de mutações já foram descritas em mais de 13 genes diferentes responsáveis por canais iônicos restauradores do potencial de repouso da célula miocárdica. O padrão de herança pode ser autossômico dominante ( *Romano-Ward* ), como nos tipos LQT 1 a 6; autossômico recessivo, associado a surdez congênita e intervalos QT muito prolongados ( *Lange-Nielsen* ); e autossômico dominante, associado a defeitos extracardíacos em associação a dismorfismos e paralisia periódica hipo ou hipercalemica ( *Andersen-Tawil* , LQT7).^379 , 381^

O risco de morte súbita depende de uma série de fatores como o tipo de mutação responsável pelo fenótipo, a duração do intervalo QT e a presença de sintomas. Pacientes com intervalo QT muito prolongado (QTc > 500ms) ou com síncope recorrente podem ter risco anual de morte súbita de até 5%.

A síndrome do QT curto é definida por intervalo QTc < 340ms ou < 360ms em sobreviventes de parada cardíaca por FV/TV, história familiar de morte súbita com menos de 40 anos de idade, presença de mutação confirmada ou história familiar de QT curto. Trata-se de doença rara em que mutações em genes de canais de potássio podem ser encontrados em até 20% dos casos.^382^

Alguns pacientes com QT curto podem se beneficiar do uso de quinidina. Sobreviventes de parada cardíaca devem ser submetidos a implante de CDI, enquanto os assintomáticos devem ser acompanhados. As recomendações para implante de CDI para prevenção primária na síndrome do QT longo e QT curto estão listadas na Tabela 27 .

**Tabela 27:** – Indicações de cardioversor-desfibrilador implantável (CDI) na prevenção primária no QT longo e QT curto

	Classe de recomendação	Nível de evidência
Pacientes com QT longo refratário a betabloqueadores e/ou a simpatectomia, com síncope ou TV polimórfica recorrente	I	B
Pacientes assintomáticos com QTc > 500ms associado a betabloqueador podem se beneficiar de CDI, associado ou não à simpatectomia	IIb	C
QT longo com estudo genético comprovando LQT2 ou LQT3, associado a betabloqueador	IIb	B
Pacientes com QTc < 330ms assintomáticos e história de morte súbita familiar	IIb	C

*CDI: cardioversor-desfibrilador implantável; TV: taquicardia ventricular.*

#### 4.1.8. Síndrome de Brugada

A síndrome de Brugada é caracterizada pelo supradesnivelamento do segmento ST > 2mm nas derivações V1 e V2 ( *coved type* ) no 2º, 3º ou 4º espaço intercostal, espontâneo ou induzido por bloqueador de canal de sódio (ajmalina, procainamida), associado à ocorrência de arritmia ventricular polimórfica, síncope ou parada cardíaca.

Pacientes com o padrão eletrocardiográfico típico (“tipo 1”) espontâneo associado à síncope inexplicada ou parada cardíaca recuperada são os que apresentam maior risco de morte súbita. O implante de CDI está associado à redução do risco nesses pacientes sintomáticos^383^ ( Tabela 28 ).

**Tabela 28:** – Indicações de cardioversor-desfibrilador implantável (CDI) na prevenção primária na síndrome de Brugada

	Classe de recomendação	Nível de evidência
TVS ou síncope de provável causa arrítmica e ECG com padrão Brugada tipo 1 espontâneo.	I	B
Síncope de provável causa arrítmica e ECG com padrão Brugada tipo 1 induzido farmacologicamente.	IIa	B
TVS induzida por EVP com um ou dois extraestímulos em dois sítios, em pacientes assintomáticos com padrão Brugada tipo 1 espontâneo ao ECG	IIb	C

*CDI: cardioversor-desfirbrilador implantável; ECG: eletrocardiograma; EVP: estimulação ventricular programada; TVS: taquicardia ventricular sustentada.*

O aspecto fenotípico da síndrome de Brugada está associado a defeitos genéticos detectáveis em até 30% dos casos. O gene SCN5A está envolvido na maioria das alterações encontradas, mas a pesquisa genética negativa não afasta o diagnóstico.^384^

Diversos fatores podem deflagrar as manifestações eletrocardiográficas ou precipitar episódios arrítmicos, como febre, agentes anestésicos e diversos psicotrópicos (www.brugadadrugs.org).

Pacientes assintomáticos têm menor risco de morte súbita. O papel da estimulação ventricular programada (EVP) na estratificação de risco é controversa. Brugada *et al* . encontraram associação entre a indução de TV polimórfica com aplicação de até 2 extraestímulos no VD e o risco de morte em pacientes assintomáticos. Já a indução de arritmia com 3 extraestímulos reduz a especificidade e deve ser evitada. Outros autores encontraram redução do valor preditivo positivo da EVP ao longo do tempo.^385 - 387^

Pacientes com tempestade elétrica e disparo e terapias de choque pelo CDI podem se beneficiar de controle clínico com quinidina e com ablação epicárdica de áreas de ativação anormal do VD identificados no mapeamento eletroanatômico.^388 , 389^

#### 4.1.9. Taquicardia Ventricular Polimórfica Catecolaminérgica (TVPC)

A TVPC é uma rara doença arritmogênica, de origem genética, caracterizada por taquicardia ventricular bidirecional e polimórfica, adrenérgico-dependente. A prevalência estimada é de 1 em 10.000 indivíduos. Já foram identificados dois tipos de genes: uma variante dominante secundária à mutação no gene do receptor cardíaco do rianodinio (RyR2) e uma rara variante recessiva causada por mutação do gene da calsequestrina cardíaca (CASQ2).^390^

A manifestação clínica usualmente ocorre na 1ª ou 2ª década de vida, sendo desencadeada por exercício físico ou estresse emocional. Em geral, o ECG e o ecocardiograma são normais, mas o teste ergométrico desencadeia arritmias atriais e ventriculares (bidirecional e polimórfica).

O tratamento de escolha é o uso de betabloqueador em dose máxima tolerável. A flecainida e a simpatectomia cardíaca esquerda podem ser associadas como terapias coadjuvantes.

O CDI deve ser considerado nos casos que apresentam parada cardíaca, síncopes recorrentes ou taquicardia ventricular a despeito da TFO^391^ ( Tabela 29 ).

**Tabela 29:** – Indicações de cardioversor-desfibrilador implantável (CDI) na prevenção primária na taquicardia ventricular polimórfica catecolaminérgica

	Classe de recomendação	Nível de evidência
TVPC com síncope ou TVS, apesar do uso de betabloqueador em dose máxima tolerada, ou que tenham contraindicação ao uso de betabloqueador e expectativa de vida > 1 ano	IIa	C
TVPC assintomática que apresente boa resposta ao tratamento com betabloqueador	III	C

*CDI: cardioversor-desfirbrilador implantável; TVPC: taquicardia ventricular polimórfica catecolaminérgica; TVS: taquicardia ventricular sustentada.*

Habitualmente, o CDI deve ser programado com longo intervalo de detecção, porque a dor e o estresse do choque podem desencadear mais arritmias e, consequentemente, tempestade elétrica. A decisão do implante deve levar em conta a alta probabilidade de disparo de choques (apropriados e inapropriados), além da chance de complicações devido à baixa faixa etária dos pacientes.

Em revisão sistemática recentemente publicada, a incidência de choque foi de 40%, tempestade elétrica 19,6%, mortalidades após implante atingiu 1,4% e complicações adicionais chegaram a 32,4% (fratura de eletrodo, endocardite e revisões cirúrgicas), em um faixa etária média de 15 anos (11 a 21 anos).^392^

#### 4.1.10. Taquicardia Ventricular Idiopática

Arritmias ventriculares em pacientes com coração estruturalmente normal são, na grande maioria das vezes, benignas. Contudo, uma pequena parcela de pacientes pode apresentar formas malignas de taquicardia ventricular monomórfica, polimórfica e até mesmo fibrilação ventricular.

Muitas dessas taquicardias são desencadeadas por ectopias ventriculares originadas em regiões muito similares àquelas de caráter benigno (via de saída, cúspide aórtica, sistema His-Purkinge, anel mitral e músculos papilares). O mecanismo exato dessas arritmias ventriculares malignas ainda não é completamente esclarecido. A anisotropia associada a condução lenta e bloqueio funcional causados por focos arritmogênicos rápidos provavelmente causa degeneração do ritmo para TV polimórfica e FV.

Características de alto risco estão relacionadas com a ocorrência de síncope ou parada cardíaca e achados eletrocardiográficos de intervalo de acoplamento curto da primeira ou segunda extrassístole, TVNS com ciclos curtos, QRS alargado (em TV ou sinusal) e TV polimórfica.^393 , 394^ A Tabela 30 lista as recomendações para prevenção primária com implante de CDI em pacientes com arritmias ventriculares idiopáticas.

**Tabela 30:** – Indicações de cardioversor-desfibrilador implantável (CDI) na prevenção primária na taquicardia ventricular idiopatica

	Classe de recomendação	Nível de evidência
Pacientes com síncope e TVNS com características malignas e expectativa de vida >1 ano	IIb	C
Pacientes com TV idiopática assintomática, com características benignas, que apresentem boa resposta ao tratamento farmacológico ou ablativo	III	C

*CDI: cardioversor-desfibrilador implantável; TVNS: taquicardia ventricular não sustentada; TV: taquicardia ventricular.*

## 4.2. Prevenção Secundária de Morte Súbita

### 4.2.1. Recuperados de Parada Cardíaca ou Taquicardia Ventricular Sustentada

#### 4.2.1.1. Recuperados de Parada Cardíaca ou Taquicardia Ventricular Sustentada na Presença de Cardiopatia Estrutural

A parada cardiorrespiratória (PCR) por taquicardia ventricular ou fibrilação ventricular (TV/FV) e, subsequentemente, a morte súbita cardíaca (MSC) constituem um grave problema de saúde pública, representando aproximadamente 50% de todas as mortes cardiovasculares. Adicionalmente, dos pacientes que apresentam PCR fora do ambiente hospitalar, a taxa de sobrevida é bastante baixa, entre 6 e 10%.^395^ Além disso, esses poucos pacientes que conseguem sobreviver à PCR por TV/FV apresentam alto risco de recorrência de taquiarritmias potencialmente fatais, tornando fundamental a adoção de medidas preventivas, que inclui o tratamento da cardiopatia de base, das comorbidades, do uso de fármacos antiarrítmicos e da seleção de pacientes para implante de CDI.^396 , 397^

Nos pacientes com cardiopatia isquêmica ou dilatada, é bastante conhecido o papel protetor de fármacos como os betabloqueadores, inibidores da enzima conversora de angiotensina (IECA), bloqueadores do receptor da angiotensina (BRA) e estatinas, que reduzem a mortalidade total, a mortalidade cardiovascular e a mortalidade súbita.^398^

Uma metanálise^399^ envolvendo mais que 35.000 pacientes com disfunção ventricular esquerda (FEVE < 40%), demostrou que fármacos como betabloqueadores, IECA, BRA e antagonistas dos receptores mineralocorticoides reduzem o risco de MSC quando comparados ao placebo (HR: 0,89, IC95%: 0,82-0,98, p = 0,02). Quando associado à TFO, o implante de CDI traz benefício adicional ao tratamento clínico, reduzindo ainda mais a taxa de MSC (HR; 0,39, IC95%: 0,30-0,51, p < 0,0001). Mais recentemente, a combinação de um inibidor (LCZ696) da neprilisina com BRA (sacubitril/valsartana) mostrou-se ainda mais eficiente que o enalapril na redução tanto da mortalidade por IC quanto da mortalidade arrítmica.^258^ Questiona-se se o LCZ696 teria ação antiarrítmica primária ou se a redução das arritmias cardíacas seria resultado de melhora clínica da IC.^400^

O emprego de fármacos antiarrítmicos foi, durante muitos anos, a principal estratégia de prevenção secundária de MSC, ainda que fundamentado em poucos estudos e com elevada taxa de recorrência de eventos. Até o início dos anos 1990, aceitava-se que antiarrítmicos da Classe I (p. ex., quinidina, flecainida, encainida) reduziam as extrassístoles ventriculares e a mortalidade. Com a demonstração subsequente dos efeitos deletérios desses fármacos pós-IAM e na IC, a amiodarona passou a ser a escolha para esses pacientes. O estudo CASCADE envolveu 228 pacientes recuperados de PCR que foram randomizados para tratamento empírico com amiodarona ou fármacos da classe I, orientadas por EEF ou *Holter* -24h.^401^ Em seguimento de 6 anos, a sobrevida livre de eventos (morte cardíaca ou TV) foi de 41% no grupo amiodarona *versus* 20% no grupo de terapia convencional. No entanto, a falta de grupo placebo não permite concluir se os resultados decorreram de benefício do uso da amiodarona ou dos riscos associados às outras drogas antiarrítmicas.

O CDI é considerado o principal avanço para a prevenção secundária da MSC. Seus benefícios foram avaliados em uma série de ensaios clínicos randomizados. O estudo AVID^402^ comparou a terapia antiarrítmica (amiodarona ou sotalol) *versus* CDI em 1.016 pacientes recuperados de PCR por TV/FV, com TV associada à síncope ou com instabilidade hemodinâmica e FEVE < 40%. A sobrevida foi significativamente maior no grupo CDI em 1 ano (89,3% *vs.* 82,3%), 2 anos (81,6% *vs.* 74,7%) e 3 anos (75,4% e 64,1%), p < 0,02. A principal crítica a esse estudo refere-se ao maior número de pacientes em uso de betabloqueador no grupo CDI em relação ao grupo de terapia antiarrítmica. Em análise posterior, observou-se que o benefício do CDI ocorreu principalmente nos pacientes com FEVE mais baixa.^403^ Nos pacientes com FEVE > 35%, não houve diferença de sobrevida significativa. Nos pacientes com FEVE entre 20% e 34%, a sobrevida em 1 ano foi de 89,6% *versus* 79,8% e em 2 anos de 82,5% *versus* 71,8% (p < 0,05). Nos pacientes com FEVE < 20%, a sobrevida em 1 ano foi de 82,4% *versus* 73% e, em 2 anos 71,6% *versus* 63,8%, sem diferença significativa.

O estudo CIDS^404^ avaliou o uso de amiodarona *versus* CDI em 659 pacientes com FV documentada, PCR recuperada, TV associada à síncope, TV > 150 bpm/min com pré-síncope ou angina e FEVE < 35% ou síncope associada à TV indutível ou com episódio de TV espontânea documentada. A mortalidade total após seguimento médio de 4 anos foi de 27% no grupo CDI e 33% no grupo amiodarona, sendo que essa diferença não foi significativa. Em análise subsequente, foi demonstrado que, em pacientes com dois dos seguintes critérios: FEVE < 35%, CF III ou IV e idade > 70 anos, o implante do CDI foi superior.^405^ Após acompanhamento médio de 5,6 ± 2,6 anos, a mortalidade foi de 47% no grupo amiodarona comparada a 27% no grupo CDI (p = 0,002).

O estudo CASH^406^ incluiu 349 pacientes recuperados de PCR que foram randomizados para tratamento com propafenona, amiodarona, metoprolol ou implante de CDI. O tratamento com propafenona foi suspenso após uma análise interina verificar aumento de mortalidade em comparação aos pacientes com CDI. Após seguimento médio de 2 anos, a mortalidade total foi de 12,1% no grupo CDI *versus* 19,6% nos grupos amiodarona e metoprolol combinados, sendo que a diferença igualmente não foi significativa.

A metanálise que avaliou os resultados desses três estudos demonstrou 50% de redução relativa na mortalidade arrítmica (p < 0,0001) e 28% na mortalidade total entre os pacientes com CDI em comparação aos que receberam tratamento antiarrítmico, com NNT = 29 (p < 0,00006).^407^ O benefício foi maior em pacientes com FEVE < 35% e considerando seguimento de 6 anos (pacientes com CDI tiveram aumento de sobrevida de 4,4 meses).

O estudo MAVERIC comparou terapia guiada por EEF (antiarrítmicos, CDI) *versus* amiodarona empírica em pacientes com TV sustentada ou PCR recuperada.^408^ Os resultados demostraram redução de mortalidade no grupo de pacientes com CDI. A realização de EEF não demonstrou benefício.

As recomendações para implante de CDI na prevenção secundária são baseadas nesses estudos ( Tabela 31 ).

**Tabela 31:** – Indicações de cardioversor-desfibrilador implantável (CDI) para pacientes recuperados de parada cardíaca ou taquicardia ventricular sustentada na presença de cardiopatia estrutural

	Classe de recomendação	Nível de evidência
Parada cardíaca por TV/FV de causa não reversível, com FEVE ≤35% e expectativa de vida de pelo menos 1 ano	I	A
TVS espontânea com comprometimento hemodinâmico ou síncope, de causa não reversível com FEVE ≤35% e expectativa de vida de pelo menos 1 ano	I	A
Sobreviventes de parada cardíaca, por TV/FV de causa não reversível, com FEVE ≥35% e expectativa de vida de pelo menos 1 ano	IIa	B
Pacientes com TVS espontânea, de causa não reversível, com FEVE ≥ 35%, refratária a outras terapêuticas e expectativa de vida de pelo menos 1 ano	IIa	B
Pacientes com síncope de origem indeterminada com indução de TVS hemodinamicamente instável e expectativa de vida de pelo menos 1 ano	IIa	B
TV incessante	III	C

*CDI: cardioversor-desfibrilador implantável; FEVE: fração de ejeção do ventrículo esquerdo; FV: fibrilação ventricular; TV: taquicardia ventricular; TVS: taquicardia ventricular sustentada.*

#### 4.2.1.2. Recuperados de Parada Cardíaca ou Taquicardia Ventricular Sustentada na Ausência de Cardiopatia Estrutural

As canalopatias representam um grupo de patologias geneticamente determinadas que envolvem diversos tipos de disfunções dos canais iônicos dos cardiomiócitos, seja aumentando ou reduzindo suas funções e gerando desequilíbrio iônico que aumentam o risco de taquiarritmias potencialmente fatais e de MSC.^409^

A gama de mutações genéticas é extremamente ampla, com grande sobreposição de expressões fenotípicas. São classificadas como canalopatias a síndrome do QT longo congênito (SQTL), síndrome de Brugada (SB), taquicardia ventricular catecolaminérgica (TVC), síndrome do QT curto (SQTC), síndrome do ponto J (SPJ), repolarização precoce (RP). A fibrilação ventricular idiopática, a síndrome de morte súbita arrítmica e a síndrome de morte súbita na infância, ainda que possam ter outros mecanismos envolvidos que não somente os genéticos, são discutidos também nessa categoria, pela manifestação arrítmica predominante de TV/FV na ausência de cardiopatia estrutural. Não está no escopo desta Diretriz a discussão detalhada de particularidades de cada expressão fenotípica das canalopatias, sendo possível encontrar extensa referência na literatura.^410 , 411^

Pacientes com canalopatias que sobreviveram à PCR apresentam elevado risco de novo episódio de MSC. O implante de CDI nesses pacientes reduz o risco de MSC, sendo observada taxa de terapia apropriada nessa população entre 8% e 33%. Ainda, de maneira geral, pacientes com canalopatias e que apresentem síncope ou taquicardia ventricular apesar do uso de medicações apropriadas para cada condição, usualmente também têm indicação para implante de CDI, a menos que condições específicas, como idade muito prematura ou baixo peso, sejam considerados de risco para o implante do dispositivo.^412^ A decisão clínica para o implante de CDI também deve considerar outras opções terapêuticas ou terapias coadjuvantes em situações especiais, como a denervação simpática esquerda na síndrome do QT longo ( Tabela 32 ).

**Tabela 32:** – Indicações de cardioversor-desfibrilador implantável (CDI) para pacientes recuperados de parada cardíaca ou taquicardia ventricular sustentada na ausência de cardiopatia estrutural

	Classe de recomendação	Nível de evidência
Pacientes com canalopatia e PCR por TV/FV, com expectativa de vida de pelo menos 1 ano	I	B
Pacientes com canalopatia, que evoluem com TVS ou síncope, apesar do uso de fármacos, com expectativa de vida de pelo menos 1 ano	IIa	B
Pacientes com SQTLc que evoluem com síncope ou TVS, apesar do uso de betabloqueador em dose máxima tolerada	IIa	B
Pacientes com SB e alterações eletrocardiográficas espontâneas, síncope presumidamente de causa arrítmica e expectativa de vida de pelo menos 1 ano	IIa	C
Pacientes com SB e documentação de TVS, com ou sem síncope e expectativa de vida de pelo menos 1 ano	IIa	C
Pacientes com SB e alterações eletrocardiográficas induzidas por fármacos, síncope de origem indeterminada e expectativa de vida de pelo menos 1 ano	IIb	C
Pacientes com TVPC que evoluem com síncope ou TVS, apesar do uso de betabloqueador em dose máxima tolerada e expectativa de vida de pelo menos 1 ano	IIa	C

*CDI: cardioversor-desfibrilador implantável; FV: fibrilação ventricular; PCR: parada cardiorrespiratória; SB: síndrome de Brugada; SQTLc: síndrome do QT longo congênito; TV: taquicardia ventricular; TVPC: taquicardia ventricular polimórfica catecolaminérgica; TVS: taquicardia ventricular sustentada.*

#### 4.2.2. Síncope e Taquicardia/Fibrilação Ventricular no Estudo Eletrofisiológico

A síncope é um sintoma raro, exuberante e com grande diversidade de etiologia, mecanismos fisiopatológicos e prognósticos, podendo ser o único sintoma que precede a morte súbita. É definida como a perda súbita e transitória da consciência devido à hipoperfusão cerebral, com restabelecimento rápido, espontâneo e completo da consciência.

Na presença de cardiopatia, a ocorrência de síncope, mesmo de origem indeterminada, pode indicar a necessidade de implante de CDI para prevenção de MSC, devido à grande relação dessa manifestação com arritmias ventriculares potencialmente fatais.^413^

Em algumas situações o EEF pode ser útil para confirmar a causa arrítmica da síncope. Na cardiopatia isquêmica com FEVE reduzida, a indução de taquicardia ventricular sustentada (TVS) é preditor de risco de MSC;^414^a indução de fibrilação ventricular é considerada achado inespecífico.^415^ Por outro lado, a não indução de TVS no EEF identifica um grupo de menor risco^416^ ( Tabela 33 ).

**Tabela 33:** – Indicações de CDI para pacientes com síncope e taquicardia fibrilação ventricular induzidas no EEF

	Classe de recomendação	Nível de evidência
Paciente com cardiomiopatia isquêmica e FEVE > 35% apresentando síncope de origem indeterminada, com indução de TVS ao EEF^29-30^	I	B
Paciente com cardiomiopatia não isquêmica sem indicação para prevenção primária de MS, apresentado síncope, com indução de TVS ao EEF^31-32^	IIa	B

*CDI: cardioversor-desfibrilador implantável; EEF: estudo eletrofisiológco; FEVE: fração de ejeção do ventrículo esquerdo; TVS: taquicardia ventricular sustentada.*

## 4.3. Crianças, Adolescentes e Cardiopatia Congênita

As indicações de CDI em crianças não têm sido adequadamente contempladas nas últimas diretrizes internacionais devido ao limitado número de trabalhos publicados sobre o assunto até o momento. Dessa forma, algumas publicações de pequenas séries têm direcionado a maioria das indicações atuais.^417^Frequentemente, as indicações de CDI na faixa etária pediátrica seguem critérios similares aos adultos, tanto para prevenção primária como para prevenção secundária, em que pese a importância do bom senso na avaliação desses pacientes.^418^

Recentemente, SOBRAC e DCC-CP publicaram a Diretriz de Arritmias Cardíacas em Crianças e Cardiopatias Congênitas, a qual veio normatizar o diagnóstico e o tratamento de crianças com arritmias cardíacas.^419^

É extremamente importante que o médico busque alternativas para evitar a indicação de CDI em crianças, sem que isso represente aumento de risco. Deve-se esgotar o tratamento clínico adequado e, quando indicada, a ablação dos focos arritmogênicos deve ser considerada.

Não existem evidências que suportam a utilização rotineira de CDI na população pediátrica com base apenas na disfunção ventricular esquerda, como ocorre em algumas situações da população adulta. Devido à dimensão do gerador de pulsos e calibre do cabo-eletrodo de choque, a indicação de CDI nessa população deve ser exceção em virtude de vários fatores: dificuldade técnica para implante devido à dimensão do corpo da criança, limitação para confecção da loja do gerador (muitas vezes no abdome), reduzidas opções de via de acesso, risco de tromboses/obstruções venosas e maior risco de extrusão.^420^

Para o seguimento clínico e eletrônico, além do ECG e da avaliação rotineira por telemetria, é fundamental a avaliação radiológica periódica, para acompanhar o comportamento do cabo-eletrodo de acordo com o crescimento da criança. Esse cabo deve ser implantado deixando uma curva redundante para permitir o crescimento do paciente sem a necessidade de múltiplas intervenções.^421^

A programação eletrônica também apresenta diferenças em relação ao adulto. A função de MP deve considerar frequência básica de estimulação apropriada à idade e à cardiopatia, habitualmente variando de 90 a 160ppm (adolescentes costumam acompanhar protocolos de adultos). Outros fatores importantes são a programação de intervalo AV adaptativo e período refratário atrial pós-ventricular curto, mas adequado para prevenir a ocorrência de taquicardia por reentrada eletrônica, uma vez que esses pacientes costumam ter boa condução ventriculoatrial.

A programação eletrônica do CDI e o seguimento clínico devem ser ainda mais cuidadosos.^422^ A facilidade que as crianças apresentam em desenvolver taquicardia sinusal com frequência elevada pode resultar em choques inapropriados com grande impacto psicológico. Choques inapropriados, principalmente logo após o implante, podem afetar a confiança no equipamento e na equipe médica, com desenvolvimento de *síndrome do pânico* de difícil controle.^423 - 428^

Os critérios de discriminação de arritmias e a frequência de detecção devem ser rigorosamente bem ajustados. É recomendável que as terapias sejam restritas a choques, evitando-se o uso de terapias antitaquicardia (ATP; do inglês, *antitachycardia pacing* ), visto que são raras as situações em que as arritmias potencialmente fatais se apresentam como TV monomórfica.^429 - 431^ Quando ocorrem TV monomórficas, devem ser tratadas com ablação sempre que possível.^432 , 433^As TV polimórficas respondem melhor ao disparo precoce de choques, além disso, essas arritmias facilmente degeneram para FV durante as tentativas de ATP.

Em adolescentes, o CDI subcutâneo pode ser opção bastante interessante, visto que esse sistema não utiliza cabos-eletrodos intravasculares.^434 - 437^ Essa opção, porém, pode gerar desconforto local e problemas estéticos em pacientes magros devido ao maior tamanho do gerador e presença do cabo-eletrodo paraesternal. Outra limitação do CDI subcutâneo é a incapacidade de estimular cronicamente o coração para tratar bradicardia. Choques inapropriados têm sido descritos com essas próteses em virtude de sua maior suscetibilidade à detecção de ruídos extracardíacos.^438 , 439^

Na Tabela 34 , estão dispostas as principais indicações de implante de CDI em crianças e adolescentes.

**Tabela 34:** – Recomendações para implante de cardioversor-desfibrilador implantável (CDI) em crianças, adolescentes e na presença de cardiopatia congênita

	Classe de recomendação	Nível de evidência
Recuperado de PCR, excluídas causas reversíveis;	I	B
TV instável com disfunção ventricular, excluídas causas reversíveis		A
TV sintomática após avaliação hemodinâmica e anatômica, afastada a possibilidade de correção cirúrgica ou por ablação que pode mudar ou eliminar o substrato arritmogênico		C
Síncope recorrente com disfunção ventricular ou TV induzida	IIa	B
Síncope recorrente associada a SQTL ou TV polimórfica catecolaminérgica em uso de betabloqueador em doses otimizadas		B
QT longo congênito, assintomático, não aderente ao tratamento ou história familiar de morte súbita		C
Cardiomiopatia hipertrófica obstrutiva, assintomática, com critérios de alto risco		C
Cardiopatia arritmogênica de VD com envolvimento ventricular extenso (VD e/ou VE), com TV, história familiar de morte súbita ou síncope indeterminada		C
TV incessante ou por causas reversíveis	III	C

*PCR: parada cardiorrespiratória; SQTL: síndrome do QT longo; TV: taquicardia ventricular; VD: ventrículo direito; VE: ventrículo esquerdo.*

Atualmente, devido à evolução do tratamento cirúrgico das cardiopatias congênitas, adultos jovens têm apresentado maior sobrevida. Esses pacientes podem apresentar arritmias ventriculares complexas ou até mesmo a MSC, devido à presença de cicatriz miocárdica secundária à própria cardiopatia congênita de base ou devido a manipulação cirúrgica.

Existe extensa correlação entre anormalidades hemodinâmicas residuais e a ocorrência de TV em pacientes submetidos à correção cirúrgica de *tetralogia de Fallot (T4F).* Hipertrofia e dilatação do VD, além da obstrução ou regurgitação residual da via de saída do VD, são consideradas fatores de risco para a ocorrência de TV e MSC.^440 - 446^ Abordagem híbrida, combinando a estratégia cirúrgica para o reparo das alterações estruturais com ablação da arritmia guiada pelo mapeamento pré-operatório ou intraoperatório, tem sido utilizada com sucesso na tentativa de reduzir a incidência de arritmias.^447 - 449^ A troca da válvula pulmonar isoladamente nos pacientes com *T4F* resulta em melhora hemodinâmica e funcional, mas não elimina o risco de TV, sendo necessária avaliação pós-operatória de risco de MSC e eventual indicação de CDI.^450 , 451^

Aproximadamente 50% dos implantes de CDI em adultos com cardiopatia congênita são indicados para prevenção secundária, entre 36 e 41 anos de idade.^452 , 453^ Esses pacientes apresentam choques apropriados entre 3 e 6% ao ano e taxas de complicações (26% a 45%) e choques inapropriados (15% a 25%) maiores que outras populações.^450 , 451 , 454 - 457^ Por isso, o custo-benefício e o impacto psicológico devem ser considerados quando da indicação de CDI nesta população.

Os desafios do implante de CDI em adultos com cardiopatia congênita incluem a complexidade anatômica, os desvios intracardíacos e acesso vascular limitado ao coração. O CDI subcutâneo pode ser uma boa opção para esses pacientes.^456^

A indicação de CDI para prevenção primária em pacientes com cardiopatia congênita é controversa. Kairy *et al* . propuseram um escore de risco para pacientes submetidos à correção cirúrgica de *T4F* em que a pontuação > 5 seria suficiente para a indicação de CDI. Os critérios considerados nesse escore incluíram: cirurgia de desvio sistêmico-pulmonar paliativo prévia (2), indução de TVS ao EEF (2), QRS ≥ 180ms (1), ventriculotomia (2), TVNS (2) e pressão diastólica final do VE ≥ 12mmHg (3).^457^

Os pacientes com reparo cirúrgico de *T4F* representam cerca de 50% dos implantes de CDI na população de cardiopatia congênita do adulto. Nessa população, choques apropriados ocorrem em até 7,7%/ano na prevenção primária e 9,8%/ano na prevenção secundária.^458^ A TVS induzida no EEF em pacientes com cardiopatia congênita parece não se correlacionar com a ocorrência de choques apropriados.^458^A ablação por cateter de TVS monomórfica recorrente pode ser alternativa eficaz, dispensando o implante de CDI em alguns casos.^459 - 463^

O maior risco de MSC em pacientes com cardiopatia congênita operada, de acordo com dados de grandes coortes, está na população com transposição das grandes artérias com *switch* atrial, anomalia da válvula tricúspide tipo *Ebstein* , estenose aórtica e fisiologia univentricular.^464 - 467^

Os pacientes com antecedente de cirurgia de *Senning* ou *Mustard* apresentam maior risco de MSC principalmente durante o esforço físico. Nesses pacientes, o *switch* atrial pode resultar em aumento do volume e consequente estenose das veias pulmonares e aumento das pressões diastólicas finais.^468^ Além disso, isquemia e infarto do VD já foram identificados em estudos de perfusão miocárdica em mais de 40% desses pacientes.^469 , 470^ Os fatores de risco para PCR em pacientes com *switch* atrial incluem o fechamento prévio de CIV, sintomas de IC, arritmia atrial, FEVD < 30% a 35% e QRS > 140ms.^471 , 472^Em estudo multicêntrico que avaliou pacientes com *switch* atrial após implante de CDI, a falta de betabloqueadores foi associada a alto risco de terapia apropriada do CDI.^464^As arritmias atriais frequentemente precedem a TVS em pacientes com transposição, devendo o tratamento para taquicardia atrial ser intensificado.^473 , 474^

O risco de MSC é maior entre os pacientes com doença cardíaca congênita do adulto ( Tabela 35 ) em comparação com a população geral; a idade média de óbito varia de 30 a 49 anos.^473 - 476^ Pacientes com cardiopatia congênita de complexidade moderada ou grave apresentam risco ainda maior de MSC, correspondendo a aproximadamente 25% das causas de morte cardíaca.^477 , 478^

**Tabela 35:** – Cardiopatia congênita e risco de morte súbita cardíaca

Cardiopatia congênita	Incidência de morte súbita	Características de alto risco
**Simples**		
Defeito do septo atrial	< 1,5 %	Estimulação ventricular; dilatação do ventrículo direito; hipertensão pulmonar; gene NKX2.5
Defeito do septo ventricular	< 3%	Estimulação ventricular; dilatação do ventrículo direito; hipertensão pulmonar; gene NKX2.5
**Moderada**		
Tetralogia de Fallot	1,4% a 8,3%	Síncope inexplicada; arritmia ventricular complexa ou taquicardia Ventricular sustentada; QRS >180ms; indução de taquicardia ventricular sustentada; taquicardia atrial; disfunção do ventrículo esquerdo; dilatação importante do ventrículo direito; insuficiência ou estenose pulmonar severa
Estenose aórtica	3% a 20 %	Síncope inexplicada; hipertrofia do VE importante; estenose aórtica com gradiente médio >40mmHg; disfunção ventricular
Coarctação da aorta	2%	Aneurisma no local do reparo; estenose aórtica; hipertensão arterial sistêmica; doença coronariana precoce
Anomalia de Ebstein	3% a 6 %	Cardiomegalia; fibrilação atrial; taquicardia com complexo QRS largo; insuficiência mitral; dilatação da via de saída do VD
**Severa **		
Transposição das grandes artérias	*Swicht* atrial 3% a 9,5%; *Swicth* arterial 1%; Corrigida congenitamente 17% a 25%	*Swicht* atrial mustard; fechamento prévio de comunicação interventricular; síncope inexplicada; taquicardia atrial; estenose do óstio coronariano; disfunção ventricular sistêmica; insuficiência tricúspide
Truncus Arteriosus	4%	Múltiplas cirurgias de reparo; anomalia das coronárias; disfunção e/ou hipertrofia ventricular
Cirurgia de Fontan para fisiologia de ventrículo único	2,8 a 5,4 %	Taquicardia atrial; sobrevida longa; hepatopatia perdedora de proteína; ascite

*VD: ventrículo direito; VE: ventrículo esquerdo.*

Defeitos septais com história familiar, cardiomiopatia ou bloqueio do sistema de condução podem estar relacionados com mutação do gene NKX2-5, que está associado a risco de MSC precoce, justificando implante de CDI quando o teste genético é positivo.^479 - 481^

Pacientes com formas complexas de cardiopatia congênita e várias intervenções cirúrgicas nas primeiras décadas de vida e aqueles que apresentam hipertrofia com subsequente isquemia subendocárdica apresentam maior risco de arritmias ventriculares potencialmente fatais. Outros fatores de risco para MSC em pacientes com cardiopatia congênita incluem maior complexidade da cardiopatia, arritmias ventriculares e supraventriculares, aumento progressivo da duração do QRS, disfunção ventricular sistêmica e disfunção ventricular subpulmonar. História de síncope inexplicada em adultos com cardiopatia congênita de complexidade moderada ou grave pode ser indício de risco de MSC, devendo ser considerado o EEF para avaliar a necessidade de CDI.^457^

Os adultos na faixa etária de 40 a 50 anos de idade representam 40% a 67% dos pacientes com cardiopatia congênita que recebem CDI para prevenção primária. Nesses pacientes, choques apropriados ocorrem em 14% a 22% nos primeiros 3 a 5 anos de acompanhamento.^482^ Em pacientes sem acesso vascular ou cirurgia de *Fontan* prévia, o risco de implante de CDI epicárdico pode superar os benefícios potenciais, devendo-se considerar a possibilidade de CDI subcutâneo ou transplante cardíaco.^478^

A segurança em relação ao uso de fármacos antiarrítmicos em pacientes com cardiopatia congênita pode ser influenciada pela presença de hipertrofia e disfunção ventricular. O uso de flecainida foi associado à pró-arritmia em 5,8% dos pacientes e MSC em 3,9% em um estudo.^482^ Por outro lado, a amiodarona é geralmente reservada para pacientes com arritmias sintomáticas ou para prevenir piora da função ventricular.^483 , 484^

As principais recomendações para indicação de CDI em pacientes adultos com cardiopatia congênita estão listadas na Tabela 36 .

**Tabela 36:** – Recomendações para indicação de cardioversor-desfibrilador implantável (CDI) em pacientes adultos com cardiopatia congênita

	Classe de recomendação	Nível de evidência
Pacientes adultos com cardiopatia congênita e arritmia ventricular complexa ou sustentada na presença de lesões hemodinâmicas residuais importantes, o tratamento da lesão residual, se possível, é indicado antes da consideração do implante de CDI	I	B
Em pacientes adultos com cardiopatia congênita e TV instável, recomenda-se o implante de CDI após avaliação e tratamento adequado das lesões residuais/disfunção ventricular, com expectativa de sobrevida superior a 1 ano	I	B
Em pacientes adultos com reparo de tetralogia de Fallot e TV ou FV induzida ou TVS espontânea, o implante de um CDI é razoável ao se esperar uma sobrevida superior a 1 ano	IIa	B
Em pacientes adultos com doença cardíaca congênita de alta complexidade e que tenha sido submetido a reparo cirúrgico apresentando arritmia ventricular frequente e/ou complexa, betabloqueador pode ser benéfico para reduzir o risco de morte súbita	IIa	B
Em pacientes adultos com doença cardíaca congênita, antecedente de reparo cirúrgico de complexidade moderada ou grave, com síncope inexplicada e disfunção ventricular moderada ou hipertrofia acentuada, o CDI ou EEF indicando CDI por TVS induzível é razoável se houver sobrevida esperada superior a 1 ano	IIa	B
Em pacientes adultos com cardiopatia congênita, FEVE < 35% e sintomas de IC, mesmo com fatores de risco adicionais, o implante de CDI pode ser considerado se houver expectativa de sobrevida significativa superior a 1 ano	IIb	B
TV incessante ou de causas reversíveis	III	C

*CDI: cardioversor-desfibrilador implantável; EEF: estudo eletrofisiológico; FEVE: fração de ejeção do ventrículo esquerdo; FV: fibrilação ventricular; IC: insuficiência cardíaca; TV: taquicardia ventricular; TVS: taquicardia ventricular sustentada.*

## 4.4. Escolha do Tipo de CDI e Modo de Estimulação

Uma vez indicado o implante de CDI para prevenção de MSC, as próximas etapas consistem em escolher a técnica de implante (transvenosa, epimiocárdica ou subcutânea) e escolher o modo de estimulação (ventricular, atrioventricular, biventricular ou atriobiventricular).

### 4.4.1. Técnica de Implante

Na ausência de comunicação intracardíaca, em pacientes com peso superior a 15kg, habitualmente, a preferência é pela técnica transvenosa.^485^ Caso não seja necessário o suporte de bradicardia com indicação de MP (BAV, DNS), o CDI subcutâneo pode ser boa opção.

### 4.4.2. Modo de Estimulação

Nos pacientes que necessitam da função MP, a escolha do modo de estimulação é fundamental. Modos que priorizam a preservação da condução atrioventricular e intraventricular espontâneas estão associados a menor incidência de FA e remodelamento ventricular relacionado ao BRE induzido pelo estímulo do VD.^486^ Ademais, em pacientes que já apresentam remodelamento do VE no momento do implante (FEVE ≤ 40% e diâmetro diastólico final do VE ≥ 60mm) e que necessitarão de estimulação ventricular, a estimulação biventricular é superior à estimulação isolada do VD.^487 , 488^

Assim sendo, diante da indicação de CDI, a escolha do modo de estimulação deve considerar se o cronotropismo e a condução atrioventricular são normais, se a condução intraventricular ocorre com padrão de BRE espontâneo ou induzido por MP e se existe ou não remodelamento do VE.

Em pacientes com DNS e condução atrioventricular e intraventricular normais, pode-se optar tanto por CDI unicamerais quanto bicamerais, desde que sejam programados algoritmos de busca de condução intrínseca para se evitar a dissincronia induzida pelo MP.

## 4.5. Custo-efetividade do CDI na Prevenção Primária e Secundária de Morte Súbita

Os CDI aumentam a sobrevida de pacientes com disfunção ventricular esquerda e risco de morte súbita cardíaca (MSC). No entanto, os custos dessa terapia são elevados e constituem limitação para sua aplicação. Esses custos referem-se ao dispositivo propriamente, gastos hospitalares, honorários médicos, complicações, reinternações e trocas de gerador de pulsos e cabos-eletrodos.

A análise de custo-efetividade é definida pelo custo em moeda corrente por QALY ( *quality-adjusted life years* ou anos de vida ajustados por qualidade).^489^ Outra forma de análise de custo-efetividade refere-se ao custo por anos de vida ganhos.^490^ Essas análises variam de acordo com as condições socioeconômicas e culturais da população estudada.

A custo-efetividade deve ser analisada, a partir de trabalhos multicêntricos, em termos de mortalidade. O CDI, por exemplo, não pode ser custo-efetivo se não proporcionar melhora de sobrevida; dessa forma, a perspectiva de sobrevida deve ser fator primordial nessa análise.

O risco de morte por causas não arrítmicas também deve ser levado em conta, uma vez que os CDI não podem preveni-las. Implantes em pacientes com alta morbimortalidade podem ter relação de custo-efetividade desfavorável. A relação de custo-efetividade torna-se desfavorável quando a taxa de sobrevida dos candidatos a CDI for <1 ano. Em pacientes idosos, alguns estudos sugerem que a sobrevida deva ser >5 anos para alcançar boa custo-efetividade.^491^ Não existem estudos de custo-efetividade do CDI em doenças menos frequentes como cardimiopatia hipertrófica e canalopatias.

## 4.5.1. Prevenção Primária

Sanders et al.,^492^ analisando os resultados dos estudos de prevenção primária MADIT, MADIT II,^247^ COMPANION,^161^ MUSTT,^493^ SCD-HeFT^245^ e DEFINITE,^494^ estimaram que o uso do CDI adicionaria 1,01 a 2,99 QALYs a um custo de US$ 68.300,00 a US$ 101.500,00. Em comparação com populações controles e considerando-se uma troca do dispositivo a cada 5 anos, a custo-efetividade foi estimada em US$ 30.000,00 a 70.200,00 por cada QALY ganho. Os autores estimaram que essa custo-efetividade seria mantida abaixo de U$ 100.000,00 dólares por QALY, contanto que os CDI mantenham a redução da mortalidade por um período de 7 anos ou mais.

Nos estudos DINAMIT e CABG Patch trial,^250^ também analisados por Sanders et al., as populações tratadas com CDI não tiveram redução de mortalidade em comparação com os controles e, portanto, o CDI não foi custo-efetivo. No estudo DINAMIT, o CDI foi testado em população com 6 a 40 dias pós infarto do miocárdio, com FEVE ≤ 35% e baixa variabilidade da frequência cardíaca. O desfecho primário (morte por qualquer causa) não foi diferente entre o grupo que recebeu CDI e o grupo controle. No estudo CABG Patch, a população consistiu em pacientes com doença aterosclerótica coronariana com FE ≤ 35%, ECG de alta resolução alterado e que se submeteram à cirurgia de revascularização miocárdica (CRVM). O implante profilático de CDI durante a CRVM também não mostrou redução do desfecho primário de morte por qualquer causa. Dessa forma, o implante profilático de CDI em pacientes com alto risco de morte súbita (FE ≤ 35%, ECG de alta resolução alterado, variabilidade de RR deprimida) não foi custo-efetivo nos primeiros 40 dias pós infarto ou imediatamente após CRVM.

Um estudo brasileiro de Ribeiro et al., publicado em 2010, avaliou a custo-efetividade em pacientes com IC sob a perspectiva da saúde pública e de saúde suplementar (a efetividade foi aferida em QALY).^495^ A relação de custo-efetividade foi de R$ 68.318 por QALY no cenário da saúde pública e R$ 90.942 por QALY na saúde suplementar. Os autores concluíram que o custo do CDI, o tempo para troca do gerador e a efetividade do CDI foram as variáveis mais influentes na análise realizada. Em cenário com pacientes mais complexos, como os do estudo MADIT, a custo-efetividade foi bem mais favorável no cenário público (R$ 23.739,00 por QALY) do que no privado (R$ 33.592,00).^496^

Outro trabalho brasileiro, de Matos et al. (2007), analisou a relação de custo-efetividade do CDI comparado com o tratamento medicamentoso.^497^ O estudo utilizou a unidade de custo por ano de vida ganho (AVG). O custo por AVG alcançado foi de R$ 20.530,00 (US$ 9.550,00) na época. Esse indicador de efetividade foi calculado com base nos parâmetros de custo incremental de R$ 54.200,00 e expectativa de vida de 2,64 anos, decorrente do uso do CDI comparado com tratamento clínico. Concluiu-se que o índice de custo-efetividade foi favorável sob as condições da realidade brasileira.

No Reino Unido, Buxton et al. (2006) encontraram valores de £ 57.000 por AVG e £ 76.000 por QALY em longo período de acompanhamento.^498^ Os autores concluíram que o uso do CDI poderia ser custo-efetivo se utilizado para pacientes com baixa FEVE, incorporando subgrupos de mais alto risco, mas não para uso generalizado.

Em 2009, Cowie et al. realizaram metanálise de estudos de prevenção primária, no contexto europeu, em pacientes com FEVE reduzida e indicações conforme a diretrizes europeias. Nesse cenário, os autores também encontraram boa relação de custo-efetividade.^499^ A estimativa média de AVG e QALY foi de 1.88 e 1.57, respectivamente, e a estimativa média de custo por QALY foi de € 31.717. Tais achados foram reproduzidos em outro registro europeu em análise de prevenção primária.^500^

Gialama et al. (2014), em revisão sistemática de avaliação econômica sobre o assunto, mostraram que o CDI pode apresentar boa custo-efetividade em grupos selecionados, sendo comparável a outras terapias cardiovasculares e não cardiovasculares já estabelecidas.^496^ Fatores como eficácia e segurança, impacto na qualidade de vida, custo do dispositivo (implante e trocas), características dos pacientes e risco de MSC foram as variáveis influenciadoras nessa análise.

## 4.5.2. Prevenção Secundária

Larsen *et al* . analisaram a custo-efetividade do CDI no estudo AVID, em que o CDI foi comparado ao tratamento antiarrítmico (principalmente amiodarona) em pacientes que sobreviveram à TVS ou FV.^501^ A custo-efetividade do CDI por “anos de vida ganhos” foi calculada em U$ 66.677,00 dólares (IC95% US$ 30.761,00 a US$ 154.768,00) comparada ao tratamento antiarrítmico para o período de 3 anos do estudo. A projeção para 6 e 20 anos manteve os custos estimados em cerca de US$ 68.000 e US$ 80.000 por anos de vida ganhos. Em análise de subgrupo, o CDI foi mais custo-efetivo nos pacientes com FV e menos com FE >35%.

Thjissen *et al* .^502^avaliaram a custo-efetividade do CDI e demonstraram resultado aceitável em comparação com outros tratamentos pelo sistema de saúde pública, como eritropoetina em pacientes dialíticos, certas quimioterapias para leucemia em idosos, transplante de pulmões e neurocirurgias para tumores malignos intracranianos. O custo por QALY foi semelhante ao de transplante de coração, hemodiálise e diálise peritoneal. Deve-se considerar, ainda, que alguns fatores podem reduzir consideravelmente a custo-efetividade, como complicações, infecções e comorbidades que reduzam a sobrevida do paciente e a longevidade do CDI.

Choques apropriados ou inapropriados podem reduzir a sobrevida e a qualidade de vida e, portanto, a custo-efetividade. Vários estudos avaliaram a importância da programação do CDI, com tempo de detecção de TVS mais prolongado e frequência cardíaca de detecção mais elevada. Essas programações foram capazes de prevenir choques inapropriados e choques “desnecessários”, com melhora de sobrevida e/ou redução de hospitalização.^503 , 504^ Dessa forma, programações menos agressivas são capazes de melhorar a custo-efetividade dos CDI.

Mealings et al. analisaram 13 estudos de custo-efetividade de CDI e ressincronizadores e usaram um método analítico para adaptação do tratamento aos custos do Reino Unido.^505^ Os autores avaliaram a custo-efetividade em vários subgrupos de pacientes, com base em critérios clínicos como a classe funcional, a duração do QRS, a idade, a presença de BRE e a etiologia isquêmica. Considerando-se um custo aceitável até o limite de £ 30.000 por QALY, os CDI foram custo-efetivos em pacientes com IC e disfunção sistólica ventricular esquerda, em classe funcional NYHA < IV e QRS < 120ms. Para pacientes com QRS entre 120 e 149ms, o CDI foi custo-efetivo apenas nas NYHA I e II. Para pacientes em NYHA IV, a custo-efetividade só foi comprovada para CDI associado ao ressincronizador em pacientes com BRE e QRS >120ms.

Em relação aos pacientes muito idosos, particularmente > 80 anos, a eficácia clínica e a custo-efetividade do CDI são duvidosas. A idade média de entrada dos pacientes nos estudos de prevenção primária e secundária foi de 58 a 66 anos e 58 a 65 anos, respectivamente. No entanto, estima-se que cerca de 28% dos pacientes elegíveis para implante de CDI tenham mais de 80 anos.^506^ Dados de mundo real revelam que cerca de 8% a 12% dos implantes nos EUA e Canadá ocorrem em pacientes com mais de 80 anos. A relação morte súbita/morte por qualquer causa decresce com a idade, sendo de 0,51 em idade < 50 anos e de 0,26 para > 80 anos.^507^Como o número de acionamentos apropriados do CDI é semelhante em todas as faixas etárias, tanto na prevenção primária quanto na secundária, a relação morte súbita/morte por qualquer causa diminui no idoso devido ao aumento das mortes relacionadas às demais comorbidades.

Pellegrini et al. estudaram o impacto da idade no momento do implante do CDI na sobrevida.^491^ Os pacientes foram estratificados para faixa etária < 65 anos, 65 a 75 anos e > 75 anos.^501^ Os pacientes com mais de 75 anos tiveram sobrevida média de 5,3 anos após o implante do CDI (metade dos outros dois grupos). Os autores calcularam que, para uma sobrevida menor que 5 anos, o custo por QALY subiria de U$ 34.000 a U$ 70.200 (no estudo de Sanders^4^) para U$ 90.000 a U$ 250.000. Nesse caso, o CDI não seria custo-efetivo se o paciente morrer em menos de 5 anos após o implante.

A relação de custo-efetividade para o CDI no Brasil e em países em desenvolvimento precisa ser analisada dentro do contexto socioeconômico, levando-se em conta aspectos locais, PIB, eficácia e complicações. Nesse sentido, deve-se priorizar situações que incluam pacientes de maior risco de morte por arritmia,função ventricular esquerda mais comprometida e menos comorbidades.

A redução do preço dos dispositivos e baterias mais duradouras pode aumentar significativamente a relação de custo-efetividade. Da mesma forma, todos os esforços devem ser realizados para se evitar choques inapropriados ou desnecessários, o que melhora a qualidade de vida (impacto positivo na avaliação do índice QALY) e aumenta a longevidade da bateria.

## 5. Recomendações para Monitor de Eventos (Loop Recorder) Implantável

O monitor de eventos implantável é um dispositivo que possibilita o monitoramento contínuo do ritmo cardíaco independentemente da participação ativa do paciente. Com capacidade de armazenamento de eventos diversos (bradicardia, taquiarritmia, pausas) e bateria com durabilidade de até cerca de 3 a 4 anos, o monitor de eventos ou *looper* implantável é uma ferramenta diagnóstica bastante atrativa para investigação de sintomas pouco frequentes (p. ex., menos que 1 vez por mês) com características suspeitas de serem atribuídos a bradi ou taquiarritmias.^508^

Em pacientes com síncope inexplicada, em que a investigação não invasiva inicial com ECG, Holter de 24 horas ou monitorização estendida não tenham esclarecido a natureza dos sintomas, o *looper* implantável demonstrou ser superior à estratégia convencional de investigação, incluindo o Tilt Test e o estudo eletrofisiológico invasivo. Particularmente em pacientes idosos e com distúrbio da condução intraventricular, a principal causa encontrada nesses estudos foi por bradiarritmia. Nesses casos, bradiarritmia foi encontrada em até 41% dos casos, sendo 70% delas BAVT intermitente.^509^

Em pacientes com AVC isquêmico criptogênico, em que não há documentação de FA, a busca ativa com ECG seriado e monitorização prolongada pode detectar episódios silenciosos de FA em até cerca de 23% dos casos.^510^A detecção de FA nesses pacientes pode determinar mudança no tratamento, o que pode significar respaldo para anticoagulação plena por tempo indeterminado. Contudo, faltam estudos randomizados que corroborem a eficácia da terapia anticoagulante em pacientes com FA silenciosa detectada por monitorização prolongada no AVC criptogênico.

O estudo Crystal AF randomizou 441 pacientes com AVC criptogênico após investigação inicial, para implante de monitor de eventos ou rastreamento convencional.^511^ Em 6 meses de seguimento, 8,9% dos pacientes com monitor implantado tiveram registro de FA com mais de 30s de duração. Em 12 meses, esse número chegou a 12%, enquanto, no grupo de seguimento convencional, foi detectado FA em 2% (p < 0,001).

O STROKE-AF trial incluiu 496 pacientes acima de 50 anos de idade e foi apresentado no International Stroke Conference (ISC) 2021 ( *late-breaking abstract* 6). Entre os pacientes com monitor implantado, episódios de FA com duração superior a 2 minutos ocorreram em 12% *versus* 1,8% (p < 0,001). Pacientes com monitor implantado receberam maior número de terapia de anticoagulação e menor recorrência de AVC. Esses dados, embora limitados, pois não são estudos controlados para avaliar a comparação entre duas estratégias de intervenção terapêutica, sugerem algum benefício na monitorização prolongada.

As recomendações atuais para implante de Looper estão listadas na Tabela 37 .

**Tabela 37:** – Indicações de monitor de eventos implantáveis ( *loop recorder* )’

	Classe de recomendação	Nivel de evidência
Síncope recorrente de origem indeterminada, sem indicação formal de MP ou CDI, após investigação clínica e laboratorial inconclusiva	I	A
Em pacientes com palpitações recorrentes de provável causa arrítmica, em que outros métodos diagnósticos não demonstraram correlação com os sintomas	IIa	B
AVC criptogênico para detecção de FA em pacientes com investigação não invasiva negativa ou inconclusiva	IIa	B
Suspeita de síncope reflexa recorrente, com episódios frequentes e severos	IIa	B
Epilepsia cujo tratamento é ineficaz	IIb	B
Quedas inexplicadas	IIb	B

*AVC: acidente vascular cerebral; CDI: cardiovesor-desfibrilador implantável; FA: fibrilação atrial; MP: marca-passo.*

## 6. Recomendações para Avaliação e Programação Eletrônica dos DCEI

### 6.1. Marca-passo Convencional

A programação eletrônica de MP deve seguir os seguintes princípios básicos:^502 , 512^

Restituir ou preservar a frequência cardíaca basal de repouso e adaptá-la ao esforço, restringindo a estimulação artificial à condição para a qual o MP foi indicado, evitando sua operação em condições em que não há benefício comprovado.
Preservar a condução atrioventricular intrínseca, quando possível.
Aumentar a longevidade da bateria do gerador de pulsos, sem perda de segurança e com benefício clínico ao paciente.
Detectar arritmias e disfunções do sistema.

A abordagem de portadores de MP consiste em avaliação clínica e eletrônica. Além da pesquisa de antecedentes pessoais (incluindo medicamentos em uso) e familiares, sintomatologia e exame físico, a avaliação clínica deve incluir o ECG de 12 derivações, fundamental para avaliar funções de sensibilidade, captura e arritmias. O ecocardiograma, habitualmente realizado antes do implante, pode ser essencial durante o seguimento para monitorar o remodelamento do VE em virtude de possíveis efeitos deletérios da estimulação crônica do VD e da síndrome de MP.

A avaliação eletrônica é realizada por meio de telemetria e deve contemplar o gerador de pulsos, os cabos-eletrodos e a recuperação de informações armazenadas na memória do dispositivo, principalmente eventos arrítmicos e disfunções.

A interrogação do sistema permite avaliar a duração da bateria do gerador, integridade dos cabos-eletrodos e medidas dos limiares de estimulação e sensibilidade. A inibição temporária do MP confirma o ritmo intrínseco, fundamental para a melhor programação do sistema. Dados estatísticos relacionados a cada câmara cardíaca e os eventos arrítmicos devem ser acessados, bem como o registro dos eletrogramas intracavitários.

A escolha do modo de estimulação deve considerar o ritmo intrínseco do paciente: sinusal normal, FA, DNS e/ou BAV.

#### 6.1.1. Doença do Nó Sinusal

A estimulação unicameral ventricular (VVI) foi amplamente utilizada inicialmente, independentemente do tipo de bradicardia, devido a simplicidade e segurança. Mais de um quarto dos pacientes em estimulação VVI, contudo, desenvolve síndrome do MP (estimulação ventricular que ocasiona condução atrial retrógrada, resultando em sintomas como dispneia, palpitações, tonturas e sinais de baixo débito cardíaco), com comprometimento significativo da qualidade de vida. Dessa forma, na DNS, a estimulação atrial permite condução AV e IV espontâneas, evitando perda do sincronismo atrioventricular, síndrome de MP e dissincronia IV secundária à estimulação do VD.^513 , 514^

A estimulação atrial pode ser realizada em modo AAI ou DDD; neste último, pode-se preservar a condução intrínseca por meio de algoritmos específicos. O modo DDD tem mais complicações relacionadas a desposicionamento de cabos-eletrodos quando comparado ao VVI; por outro lado, dispositivos AAI apresentam o dobro de reoperações em relação ao modo DDD, muitas vezes, devido ao desenvolvimento de BAV (progressão da lesão). BAV em pacientes com DNS ocorre em 0,6% a 1,9% ao ano, resultando em necessidade de mudança do sistema para DDD.^515 , 516^

O modo AAI está relacionado a menor ocorrência de FA e eventos tromboembólicos em comparação ao modo VVI em pacientes com DNS. Resultados similares são observados em modo DDD, que também se associa a menores taxas de FA e melhor qualidade de vida que o modo VVI. Tais benefícios, no entanto, não impactam em desfechos de mortalidade, IC ou morte cardiovascular.^33 - 35^

O efeito deletério da estimulação artificial do VD pode resultar em IC e pior sobrevida como consequência de dissincronia induzida. Por isso, em MP bicameral, é fundamental a programação de algoritmos de preservação da condução AV intrínseca, que prolongam automaticamente o intervalo AV ou promovem a mudança do modo de estimulação para AAI (com *back-up* ventricular), a fim de evitar estimulação desnecessária de VD em pacientes com condução AV preservada. Dados iniciais com esses algoritmos apontam redução significativa do percentual de estimulação ventricular (99% para 9%, p < 0,001) e de redução de FA (40%). Pacientes com BAV de 1º grau associado a DNS podem perder esses benefícios quando o intervalo PR é muito prolongado.^517^

No estudo DANPACE, que comparou 1.415 pacientes com DNS, o modo DDDR com IAV até 220ms foi associado a menor ocorrência de FA paroxística; IAV muito longo se associou a regurgitação mitral, aumento de pré-carga e FA, sugerindo que há um limite para o prolongamento do IAV. Dessa forma, habitualmente, não é recomendado programar IAV superior a 220ms.

Outra ferramenta importante na doença DNS é o acionamento do sensor de variação de frequência (R). Esses sensores têm por objetivo aumentar FC em situações de aumento da demanda metabólica, como o exercício físico. Três pequenos estudos demonstraram melhora na qualidade de vida e tolerância ao esforço com a ativação do sensor, cujos resultados não foram reproduzidos no estudo ADEPT.^507 , 518^

A função *automatic mode switch* (AMS) consiste na reversão do modo DDD(R) para VVI(R), em caso de surgimento de FA. Apesar de não haver fortes evidência comprovando seu benefício, recomenda-se a programação, principalmente em pacientes com FA paroxística, para alívio de sintomas.^519^

#### 6.1.2. Bloqueio Atrioventricular

No BAV, a estimulação do VD, necessária, é usualmente realizada em modo DDD ou VVI. O modo DDD mantém sincronismo AV mas está relacionado a mais complicações (6,2% *vs.* 3,2%), especialmente desposicionamento, aumento de limiares e infecção.^520^

Em estudos que comparam os modos DDD e VVI em pacientes com BAVT e DNS (PASE, CTOPP), o modo DDD não se associou a redução de mortalidade e internações cardiovasculares. O estudo CTOPP evidenciou redução de FA com o modo DDD (benefício maior em pacientes com DNS); entretanto, 26% dos casos em modo VVI apresentaram síndrome de MP, com necessidade de *crossover* . Esses pacientes apresentaram melhora significativa após reprogramação para modo DDD.^31 , 33^ Em pacientes acima de 70 anos de idade, o modo DDD parece não ser superior ao VVI em pacientes com BAVT durante seguimento de 3 anos (inclusive síndrome de MP); dessa forma, esse modo é alternativa aceitável para idosos com baixa expectativa de vida e restrição a atividades físicas.

#### 6.1.3. Fibrilação Atrial

Em FA permanente, quando não há perspectiva de reversão para ritmo sinusal, apenas a câmara ventricular necessita de estimulação. Neste caso, o modo VVI(R) é recomendado. O sensor de variação de frequência se associa a melhor capacidade funcional e qualidade de vida em pequenos estudos.^521 , 522^

#### 6.1.4. Síncope Neuromediada e Síndrome do Seio Carotídeo

Síncope neuromediada com resposta cardioinibitória caracteriza-se por períodos de bradicardia intermitente, necessitando de curtos períodos de estimulação artificial, com frequência básica elevada para compensar a súbita instabilidade que ocorre durante o evento. Nesses casos, a estimulação deve ser de curta duração, apenas durante os episódios sintomáticos (função histerese). Os modos utilizados podem ser DDI, DVI ou DDD com algoritmo para preservação de condução intrínseca. O modo VVI esteve mais associado à ocorrência de síncope e pré-síncope que a estimulação bicameral (DDD e DVI) em alguns estudos.^72 , 73^

Algoritmos como *Rate Drop Response* (RDR)^®^ e *Sudden Bradi Response* (SBR)^®^ identificam reduções abruptas da frequência cardíaca, instituindo frequência de intervenção acelerada a intervalos programáveis. Tais algoritmos são eficazes na redução de sintomas em pacientes com síncope neuromediada (cardioinibitória), em comparação a tratamento convencional sem MP. Embora não tenham sido testados contra outros modos de estimulação, tais algoritmos são eficazes e possibilitam que o MP seja programado para manter-se inibido a maior parte do tempo. No estudo ISSUE III, a estimulação DDD + RDR reduziu em 57% a chance de recorrência da síncope. O RDR foi programado para intervir quando a FC chegasse a 40bpm ou apresentasse queda de 20 batimentos em relação à FC basal (90bpm por 1 minuto).^523^

O algoritmo *Closed Loop Selection* (CLS)^®^ utiliza a bioimpedância intramiocárdica para avaliar a variação da contratilidade miocárdica para prever o início da síncope e instituir a intervenção (o aumento da contratilidade miocárdica ocorre na fase inicial dos episódios de síncope).^520 , 524^

A função de busca automática de limiar de captura tem se mostrado segura, podendo prolongar a longevidade do gerador de pulsos em 60%, reduzindo custos em 42% em 10 anos. Por isso, salvo exceções, deve ser rotineiramente habilitado^525^ ( Tabela 38 ).

**Tabela 38:** – Recomendações de programação eletrônica de marca-passo (MP) convencional

	Classe de recomendação	Nível de evidência
Modo DDD, com IAV máximo 220ms com algoritmos de busca da condução intrínseca em DNS	I	A
Modo DDD em BAVT, para evitar síndrome de MP	I	B
Modo VVIR em FA permanente	I	C
*Automatic mode-switch* deve ser rotineiramente programado	I	C
Sensor de variação de frequência em pacientes com incompetência cronotrópica	IIa	B
Busca automática de limiar de estimulação deve ser rotineiramente programada para prolongar sobrevida do gerador	IIa	B
Algoritmos específicos, histerese e IAV prolongado para evitar estimulação desnecessária em SN e HSC	IIa	B

*BAVT: bloqueio atrioventricular total; DNS: doença do nó sinusal; FA: fibrilação atrial; HSC: hipersensibilidade do seio carotídeo; IAV: intervalo atrioventricular; MP: marca-passo; SN: síncope neuromediada.*

#### 6.2. Terapia de Ressincronização Cardíaca

A avaliação de portadores de TRC deve seguir os princípios de um MP convencional, acrescentando-se a abordagem de parâmetros específicos à correção da dissincronia. Dessa forma, o ECG periódico permite avaliar se a estimulação biventricular está ativa, e o ecocardiograma, realizado após 90 dias do implante e repetido ao longo do seguimento, deve documentar o remodelamento reverso nos respondedores.

**Figura 4:**
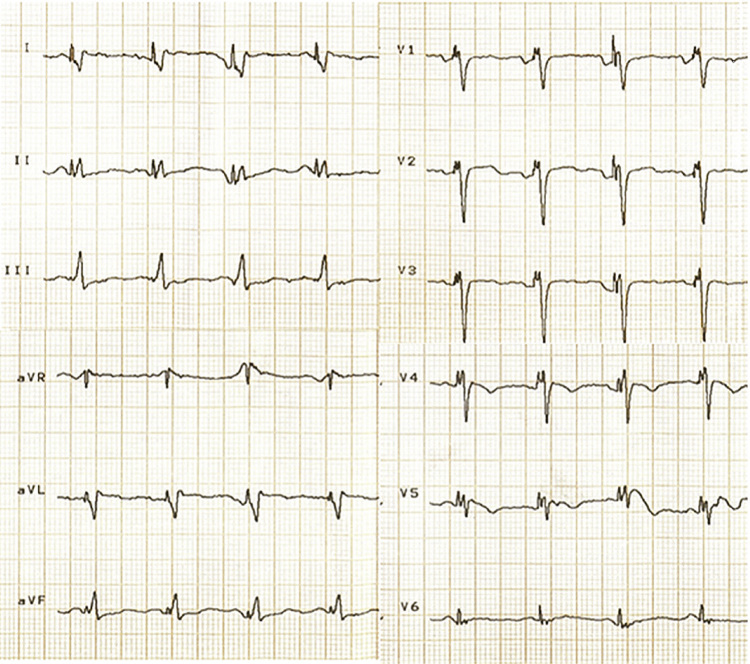
– Padrão QS em D1 e r inicial em V1 comprovando ativação precoce da parede lateral do VE em estimulação biventricular.

O padrão de ativação ventricular dependerá do posicionamento dos cabos-eletrodos, assim como da precocidade de ativação de cada câmara. A ativação do VE resulta em eixo à direita, com padrão qR ou Qr em D1 e ondas r ou R em V1 (ver Figura 4 ). Na estimulação biventricular, ocorre fusão da estimulação de ambos os ventrículos. O padrão qR ou Qr em D1 é visualizado em 90% das estimulações biventriculares. A perda da onda q ou Q em D1 é altamente sugestiva de perda de captura do VE. Ondas r ou R em V1 estão presentes em 65% a 93% das estimulações biventriculares.^526 , 527^

A perda da ressincronização pode ocorrer ao esforço, na presença de FA de alta resposta e devido ao encurtamento do intervalo AV; *Holter* -24h e teste ergométrico podem ser úteis para identificar tais cenários. Aumento do limiar de captura do VE é responsável por 10% dos casos de perda da ressincronização.^517^ Essa alteração deve ser suspeitada pela perda do padrão Qr ou qR em D1 ao ECG12 e confirmada com o teste do limiar de estimulação. Os sistemas atuais dispõem da função de busca automática do limiar de captura que ajudam na identificação de variações dos limiares não detectados em avaliação convencional.

A programação dos parâmetros básicos deve seguir os princípios dos protocolos dos grandes estudos de TRC: DDD 50bpm, IAV sentido 100 a 120ms, intervalo VV 0ms (estimulação biventricular simultânea), com sensor de variação de frequência desligado. Pacientes com FA devem ser programados em DDI na presença de eletrodo atrial, VVI se não houver eletrodo atrial, e DDD se houver FA paroxística. Nesses casos, a frequência sugerida é de 60bpm na ausência de incompetência cronotrópica.

O modo de estimulação utilizado na maioria dos grandes estudos foi DDD ou VDD 35 a 60bpm, com objetivo de reduzir estimulação atrial, o que poderia comprometer o sincronismo atrioventricular em casos de condução intra-atrial retardada, prejudicando o enchimento ventricular. Diretrizes de IC recomendam o uso de fármacos para redução da FC quando está acima de 70bpm apesar de betabloqueadores, corroborando a programação de frequências básicas baixas e sensores de frequência desligados rotineiramente.^528^

Intervalo AV curto, 100 a 120ms, visa atingir estimulação biventricular próximo a 100%, evitando perda de captura biventricular associada a encurtamento do intervalo PR. O ajuste dos intervalos AV e VV pelo ecocardiograma, ou por outros métodos, é habitualmente reservado aos não respondedores, uma vez que falta consenso quanto à real utilidade desses métodos quando aplicados rotineiramente.^529^

A frequência máxima de sincronismo atrioventricular deve ser programada ao máximo, considerando-se a FC máxima predita para idade e eventuais limitações relacionadas à cardiopatia de base.

Pacientes com estimulação acima de 93% têm redução de 44% nas taxas de mortalidade e internação por IC (desfechos combinados), sendo os melhores resultados alcançados acima de 98%. Batimentos de fusão e pseudofusão podem superestimar o percentual de estimulação biventricular. Em pacientes com FA, quando o controle da FC não é alcançado com o tratamento clínico otimizado, a ablação do nó AV deve ser realizada, uma vez que se associa à redução de mortalidade.^206^A ablação da FA paroxística e persistente (isolamento das veias pulmonares) deve ser considerada em pacientes com IC.^520^ A análise detalhada das situações clínicas que favorecem a indicação de ablação foge do escopo desta diretriz.

Extrassístoles ventriculares estão relacionadas à redução na taxa de estimulação biventricular e redução do remodelamento reverso, mesmo com incidência relativamente baixa. Fármacos antiarrítmicos e, eventualmente, ablação devem ser considerados em pacientes não respondedores.^530^

Intervalos atrioventriculares curtos resultam em sístole ventricular precoce, não permitindo que a fase de contração atrial ocorra integralmente (onda A truncada). Nesses casos é necessário prolongar o intervalo AV até que a onda A fique evidente. Intervalos AV prolongados, inversamente, geram fusão das ondas E e A. Nesses casos, é necessário encurtamento do intervalo AV.

Dois métodos são habitualmente recomendados para o ajuste do intervalo AV: o método interativo e o método de Ritter. No método interativo, programa-se intervalo AV longo (200ms) e reduz-se gradativamente (20ms por vez) até 60ms analisando o fluxo mitral. O menor intervalo AV capaz de manter as ondas E e A sepradas (sem fusão), sem deformação da onda A e mantendo 40ms de distância do final da onda A ao início do QRS é o intervalo AV ótimo. O método de Ritter é realizado aferindo o intervalo QA (início do QRS ao final da onda A) em dois intervalos AV diferentes, um curto (60ms) e um longo (200ms). O intervalo AV ideal é calculado pela fórmula IAV = IAV longo – (QA[IAVcurto] – QA[IAV longo]). Assim, recomenda-se realizar ECO com avaliação do fluxo mitral após implante para analisar o sincronismo atrioventricular: se as ondas E e A se apresentarem separadas e o intervalo do final da onda A acima de 40ms, não há necessidade de ajuste de intervalo AV.

O intervalo VV pode ser programado empiricamente, ou ajustado também por ECO, ECG e algoritmos específicos. Com ECO, o ajuste é realizado testando-se diversos intervalos e avaliando a dissincronia. O intervalo que resultar em menor dissincronia deve ser o intervalo VV programado. Deve-se testar estimulação simultânea, estimulação precoce no VE e diferentes intervalos VV, 60, 40, 20ms. Posteriormente, deve-se testar os mesmos intervalos com estimulação precoce do VD. Os métodos mais utilizados são o modo M, com ou sem doppler tissular, e a velocidade de encurtamento longitudinal do VE, aferida pelo Doppler tissular.

Assim como o ajuste do intervalo AV, a programação do intervalo VV guiado por ECO deve ser realizada em não respondedores e em condições específicas.

Alguns dispositivos dispõem de algoritmos automáticos de ajuste de IAV e IVV, cuja eficácia ainda é controversa. De qualquer forma, não parecem ser inferiores ao ajuste empírico ou guiado pelo ECO.

Correlação entre diminuição da duração do QRS com a estimulação biventricular e a taxa de respondedores foi evidenciada em estudos retrospectivos, dando suporte à hipótese de que ajuste de intervalo AV e VV visando a QRS mais curto pode aumentar a taxa de resposta à TRC.^531^

A estimulação multiponto fundamenta-se no princípio de estimular as regiões com ativação mais tardia do VE, por meio de eletrodo quadripolar, especialmente áreas basais e apicais, permitindo ativação de maior massa ventricular de forma mais rápida e homogênea. O estudo MPP trial comparou a estimulação multipontos com eletrodo quadripolar *versus* estimulação convencional, com resultados semelhantes entre as duas formas de programação, significância estatística para não inferioridade. No entanto, entre os pacientes com estimulação multiponto com 30mm de distância entre os dois pontos de estimulação de VE e com o menor intervalo (5ms), houve menor taxa de não respondedores. Esses resultados foram reproduzidos na primeira fase do estudo MORE-CRT e, mais uma vez, os pacientes com programação de 30mm de distância entre os pontos de estimulação do VE associado ao menor intervalo intra e interventricular apresentaram melhores resultados.

#### 6.3. Cardioversor-desfibrilador Implantável

A programação do CDI deve ser voltada a quatro princípios básicos: 1) reduzir a mortalidade por meio da terapia efetiva na reversão de arritmias ventriculares potencialmente fatais; 2) priorizar a reversão de arritmias ventriculares por meio das terapias antitaquicardia sem choque (ATP), sempre que possível; 3) evitar choques inapropriados; e 4) reduzir ao máximo o percentual de estimulação artificial do VD (terapia antibradicardia).

As terapias apropriadas para reversão de FV e TV sustentadas são os pilares da redução de mortalidade na intervenção com CDI. Para isso, devem ser programadas terapias escalonadas em diferentes zonas de frequência, classificadas como TV (1 ou 2 zonas) e FV. As terapias programáveis incluem choques (até 35 ou 40J) e terapias com estimulação artificial de 3 a 20 pulsos com frequência superior à da taquicardia ( *antitachycardia pacing* [ATP]) que podem reverter TV monomórficas sem a aplicação de choques, de modo indolor e com redução do dano miocárdico eventualmente provocado pelos choques.

A efetividade dos choques na reversão de arritmias ventriculares costumava ser testada no intraoperatório, por meio da realização de limiar de desfibrilação (indução de FV, seguida de disparo de choque para reversão da circulação efetiva, com energia pelo menos 10J abaixo na energia máxima programável). Estudos subsequentes comprovaram ser desnecessária essa estratégia, uma vez que medidas intraoperatórias normais (limiar de estimulação, impedância e onda R) se correlacionam com efetividade adequada na reversão de arritmias de ocorrência espontânea.^532^ Assim, é possível evitar eventuais intercorrências decorrentes da indução de FV no intraoperatório e dano miocárdico provocado pelos choques. A programação da zona de FV, portanto, deve incluir choques de energia máxima possível, com inversão da polaridade entre um choque e o outro. Nas zonas de TV, podem ser programados choques de energia mais baixa, que habitualmente são precedidos por tentativas de ATP.^533^

É bem conhecida a eficácia do ATP como primeira linha nas arritmias ventriculares. Taquicardias ventriculares monomórficas, organizadas, com ciclo estável e especialmente sem repercussão hemodinâmica, podem ser facilmente revertidas com ATP, incluindo pulsos com intervalo fixo ( *Burst* ) ou com aceleração entre os pulsos ( *Ramp* ).

Algumas arritmias instáveis, mesmo em zonas de alta frequência (na faixa de FV), podem ser interrompidas com ATP antes do disparo de choques programados naquela zona. Nesse caso, uma tentativa de ATP durante ou antes do carregamento da energia de choque é programada; em caso de reversão da arritmia, o choque é abortado. O estudo PainFREE II utilizou o ATP como primeira linha de tratamento em zona de 188 a 250bpm, com redução significativa de 71% do risco relativo de choque, sem comprometimento da segurança dos pacientes.^534^

A programação adequada de detecção e terapia escalonada é capaz de reduzir choques inapropriados, proporcionar mais terapia apropriada com ATP e reduzir a mortalidade.^535^Para isso, os princípios básicos de programação devem incluir:

1. Zona de FV programada com frequência acima de 233bpm (nos dispositivos Medtronic, acima de 188), com pelo menos 30 batimentos em 40 (x em y) para detecção. Essa estratégia permite evitar choques em arritmias não sustentadas e choques inapropriados em situações de ruídos intermitentes, dupla contagem ou extrassistolia isolada.
2. Em pacientes de prevenção primária, uma única zona de detecção de FV pode ser suficiente. Zonas de monitoramento de TV sem terapias (monitor) podem ser programadas a critério médico. Nos casos de prevenção secundária, terapias focadas para TV devem ser programadas com corte de detecção de 10-20bpm menores que a frequência da taquicardia documentada. De acordo com o critério clínico, podem ser programada zonas de terapia com frequência mais baixa, na dependência do risco de TV mais lenta, sempre priorizando o ATP.^536^
3. Algoritmos de monitoramento de ruídos e danos dos cabos-eletrodos devem ser programados, assim como recursos de autoajuste e supressão de *oversensing,* como a detecção de onda T.
4. Programação adequada dos algoritmos de discriminação de arritmias supraventriculares, especificamente no critério de avaliação da morfologia nos dispositivos unicamerais e na avaliação de algoritmos baseados na relação atrioventricular nos dispositivos bicamerais. É interessante que os limitadores de tempo, como SRD ( *Boston* *Scientific* ) e *Timeout* , sejam desabilitados, pois esses recursos ignoram a discriminação de um evento classificado como TSV após o período preestabelecido e liberam a terapia que seria inapropriada.

Finalmente, é preciso avaliar cuidadosamente a necessidade concomitante de estimulação cardíaca artificial para bradiarritmia. A maioria dos portadores de CDI não requer terapia antibradicardia, especialmente nos casos de prevenção primária. Contudo, é sabido que a estimulação convencional do VD aumenta o risco de disfunção ventricular e a mortalidade. A programação deve, sempre que possível, priorizar a diminuição do percentual de estimulação artificial do VD. Para isso, em CDI unicameral, deve-se programar em modo VVI com 40ppm e, nos bicamerais, deve-se priorizar a estimulação atrial isolada através de algoritmos de minimização de estimulação ventricular (p. ex., RYTHMIQ, MVP, IRS plus) ou da programação de intervalo AV longo o suficiente para evitar a estimulação ventricular desnecessária. Em pacientes com necessidade de estimulação ventricular por bloqueio da condução AV, deve-se considerar, de acordo com a função ventricular, a possibilidade de estimulação de sítios alternativos como a estimulação biventricular (TRC) ou do sistema excito-condutor (His/ramo esquerdo).^537^

## 6.4. Monitor de Eventos Implantável ( *Loop Recorder* )

O monitor de eventos implantável deve ser programado de maneira adequada a detectar a atividade elétrica ventricular, sem perdas de sensibilidade sinal ( *undersensing* ) e sem *oversensing* de ruídos que prejudiquem a identificação do ritmo.

A capacidade de detecção automática, independentemernte do acionamento do paciente, a memória com capacidade de armazenamento de eletrogramas e a disponibilidade de bateria com autonomia de até 3 a 4 anos tornam essa ferramenta muito útil para o esclarecimento de eventos arrítmicos não registrados em exames usuais.

A técnica de implante é semelhante entre os modelos atualmente disponíveis, sendo necessário confirmar, no intraoperatório, a adequação do sinal elétrico na posição escolhida para o posicionamento do dispositivo. Após a confirmação da captação adequada de sinal, é necessário ajustar a programação para detecção de arritmias de forma individualizada.^538^Alguns fabricantes sugerem programação inicial empírica, conforme:

- Pausas: 3s
- Bradicardia: FC ≤ 30bpm por mais de 4 batimentos consecutivos
- Taquicardia: FC acima da FC máxima predita para a idade (220- idade) por 16 ou mais batimentos;
- Fibrilação atrial: > 2 minutos classificado como ritmo de FA.

É necessária a adjudicação cuidadosa dos episódios registrados no monitor, uma vez que falsas detecções podem estar presentes na memória. Episódios classificados como FA, por exemplo, podem ser mal classificados em virtude de variações de intervalo RR por extrassistolia ventricular ou *undersensing* intermitente do QRS. Em estudo recente, a análise de 695 transmissões espontâneas ou agendadas, Afzal et al. encontraram até 81% de falsos eventos.^539^A programação adequada do critério de detecção e a análise posterior dos traçados é fundamental na otimização do monitoramento.

## 6.5. Monitoramento Remoto (Via *Web* )

O monitoramento remoto via *web* é uma realidade no seguimento dos portadores de DCEI. A transmissão de dados é possível via conexão do dispositivo a uma banda larga de internet ou, em dispositivos mais recentes, via *bluetooth* conectado a um smartphone. Por meio dessa tecnologia, é possível acessar diversos parâmetros de programação como frequência e modo de estimulação, energia de estimulação, parâmetros de detecção e sensibilidade, assim como registros de diagnósticos e *status* da bateria.

A transmissão de dados precisa ser ajustada, pois algumas informações serão transmitidas de forma ativa após acionamento de algum alarme, ou de forma passiva através do acionamento pelo paciente ou, ainda, de forma programada, mediante cronograma de transmissões. O acesso remoto às informações é disponibilizado ao serviço que acompanha o paciente mediante acesso privado ao servidor do sistema e resguardadas as garantias de privacidade de dados do paciente.

## 7. Recomendações para Prevenção e Tratamento de Infecções e Explante de DCEI

### 7.1. Prevenção e Tratamento de Infecções

Publicações recentes têm mostrado aumento da incidência de processos infecciosos relacionados a DCEI. Fatores demográficos e clínicos, como o envelhecimento populacional e comorbidades, podem influenciar tanto as contaminações hematogênicas quanto aquelas diretamente relacionadas ao implante e troca dos dispositivos. Um levantamento recente conduzido pela EHRA demonstrou que infecções relacionadas a DCEI são mais frequentes após reoperações, inclusive trocas isoladas de gerador de pulsos.^540^

Os consensos mais recentes têm chamado a atenção para a necessidade de padronização de condutas e para a formação de times de especialistas para a abordagem desse tipo particular e pouco frequente de infecção, com a finalidade de mitigar as controvérsias entre especialistas, ainda frequentes.^541 - 546^

Processos infecciosos que envolvem DCEI manifestam-se de duas formas principais: envolvimento da loja do gerador de pulsos ou exclusivamente intravascular. O acometimento exclusivo da loja é mais frequente, ocorrendo em aproximadamente 60% dos casos (geralmente por contaminação durante a cirurgia ou manipulação subsequente). A erosão da pele tardiamente pode ocorrer devido a/ou resultar em infecção da loja; em ambos os casos, a infecção pode progredir para infecção sistêmica. O acometimento da loja associado à infecção intravascular ocorre em aproximadamente 20% das infecções e habitualmente é secundário à demora ou a condutas inadequadas. O acometimento intravascular exclusivo também ocorre em cerca de 20% dos casos, por contaminação sanguínea na maior parte das vezes.^547^Essa contaminação pode ocorrer durante bacteremia causada por foco infeccioso distante, como tromboflebite séptica, osteomielite, pneumonia, infecção do sítio cirúrgico, cateteres vasculares contaminados ou infecção bacteriana originada da pele, boca, trato gastrointestinal ou urinário.

Um consenso de especialistas, encabeçado pela EHRA e endossado por outras sociedades internacionais, teve como objetivo principal definir a terminologia que deve ser utilizada em registros e estudos clínicos para a abordagem terapêutica das infecções e remoção de DCEI.^548^ Na Tabela 39 , encontram-se as adaptações para a língua portuguesa da terminologia recomendada para a diversas apresentações clínicas.

**Tabela 39:** – Tipos de infecção relacionadas ao DCEI

Tipos de infecção relacionados ao DCEI		
	Cenários clínicos	Definição
Local	Infecção incisional superficial	Envolve apenas pele e tecido subcutâneo sem acometer o DCEI
	Infecção isolada da loja	Presença de sinais clínicos de inflamação restritos à loja do gerador de pulsos (eritema, calor, flutuação, deiscência da ferida, fragilidade tecidual ou drenagem purulenta) com hemoculturas negativas
	Erosão de loja	Extrusão completa ou parcial de gerador de pulsos ou de cabo-eletrodo através da pele
Sistêmica	Bacteremia	Hemoculturas positivas associadas ou não a sintomas ou sinais de infecção sistêmica
	Infecção ou erosão de loja com bacteremia associada	Infecção ou erosão de loja de gerador com hemoculturas positivas, na ausência de vegetação valvar ou aderida a cabo-eletrodo
	Endocardite relacionada ao DCEI sem infecção de loja	Bacteremia e vegetação valvar ou aderida a cabo-eletrodo, na ausência de infecção de loja
	Endocardite relacionada ao DCEI com infecção de loja	Bacteremia e vegetação valvar ou aderida a cabo-eletrodo, na presença de infecção de loja
	Bacteremia oculta com provável infecção relacionada ao DCEI	Bacteremia em portador de DCEI, quando excluídas outras causas infecciosas

*DCEI: dispositivo cardíaco eletrônico implantável.*

O diagnóstico definitivo de infecção relacionada a DCEI baseia-se em três achados principais: 1) presença de coleção purulenta ou exteriorização do DCEI ao exame clínico; 2) crescimento de microrganismos em hemoculturas e 3) presença de vegetações na valva tricúspide ou em cabos-eletrodos evidenciada pelo ecocardiograma transesofágico (ETE). Quando não se consegue definir o diagnóstico da infecção relacionada ao DCEI com esses critérios, outros exames complementares (como PET-CT) podem ser necessários. Os critérios modificados da Duke University para diagnóstico de infecção de DCEI estão elencados nas Tabelas 40 e 41.

**Figura 5:**
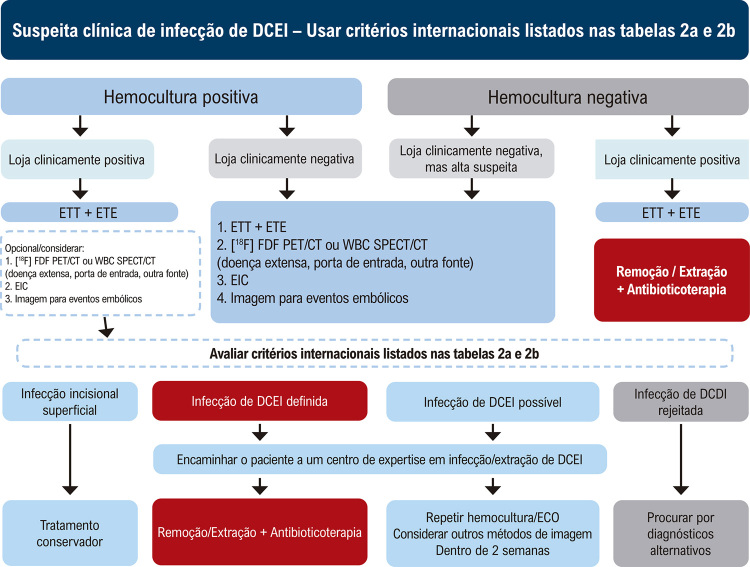
– Fluxograma para diagnóstico e do tratamento de infecção de DCEI. DCEI: dispositivo cardíaco eletrônico implantável; ECO: ecocardiograma; ETT: ecocardiograma transtorácico; ETE: ecocardiograma transesofágico; FDG: fluordeoxiglicose; PET/CT: tomografia computadorizada por emissão de pósitrons; SPECT/CT WBC: tomografia computadorizada por emissão de fóton único (single photon emission computed tomography) com leucócitos marcados.

A comprovação de que o DCEI está definitivamente contaminado é fundamental para o tratamento adequado do paciente, uma vez que, comprovada a contaminação, sua remoção completa será fundamental para o sucesso do tratamento. Por outro lado, se não houver o DCEI, estiver livre de contaminação e o processo infeccioso estiver relacionado a outro foco, a remoção desnecessária do dispositivo implicará custo desnecessário e risco cirúrgico relacionado à extração dos cabos-eletrodos. O fluxograma para diagnóstico e tratamento de infecções de DCEI está representado na Figura 5 .

Exames de imagem são importantes tanto para o diagnóstico quanto para a condução do tratamento. Nesse sentido, algumas informações obtidas por imagem podem ser relevantes: 1) identificação do tipo de DCEI; 2) identificação de cabos-eletrodos abandonados; 3) achado de vegetações intracardíacas e seu tamanho; 4) sinais sugestivos de embolização séptica para os pulmões.

No caso de febre em portador de DCEI em que não se consegue definir infecção pela avaliação da loja do gerador, hemoculturas ou ecocardiografia transesofágica e exames de imagem baseados na captação de radiofármacos podem ser importantes.

**Figura 6:**
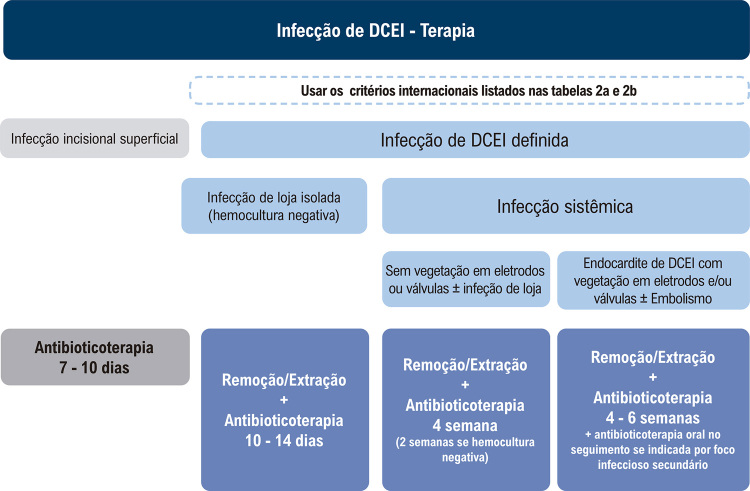
– Duração do tratamento e necessidade de remoção do sistema nas diferentes apresentações de infecção relacionada a DCEI. DCEI: dispositivo cardíaco eletrônico implantável.

Embora a remoção completa do gerador de pulsos e de todos os cabos-eletrodos seja essencial, o tratamento da infecção deve ser feito, fundamentalmente, com o uso de antimicrobianos. A escolha do antibiótico deve ser estabelecida a partir das culturas de sangue, de fragmentos da loja e dos cabos-eletrodos removidos. Quando não é possível a definição do microrganismo, o uso empírico de antibióticos deve ser definido por critérios clínicos. Da mesma forma, o tempo de tratamento também deve ser definido em função do quadro clínico, sempre contado a partir da remoção completa do DCEI ( Figura 6 ).

A remoção completa do DCEI é fundamental para evitar a recorrência da infecção. A extração dos cabos-eletrodos, entretanto, raramente deve ser considerada emergência, mesmo em choque séptico. À exceção dos implantes recentes que costuma ser tecnicamente mais fácil, a extração somente deverá ser realizada quando o paciente estiver com boas condições hemodinâmicas e com o quadro infeccioso estabilizado, face aos riscos associados ao procedimento (aderências às veias e ao coração).

A técnica de extração dos cabos-eletrodos deve ser preferencialmente transvenosa, exceto quando os cabos-eletrodos são epicárdicos ou quando houver vegetação intracavitária maior que 2,5cm em seu maior diâmetro. As recomendações para remoção do gerador de pulsos e dos cabos-eletrodos estão listadas na Tabela 42 .

**Tabela 40:** – Critérios modificados para o diagnóstico de infecções relacionadas a DCEI

Infecção de loja do gerador confirmada	Presença de um dos seguintes achados: 1) edema, eritema, calor, dor e secreção purulenta; 2) formação de fístula; 3) deformação, aderência e erosão iminente da pele; 4) exposição do gerador ou de cabo-eletrodo
Endocardite relacionada ao DCEI confirmada	Presença de: 1) dois critérios maiores; 2) um critério maior e três menores
Endocardite relacionada ao DCEI provável	Presença de: 1) um critério maior e um menor; 2) TRÊS critérios menores
Endocardite relacionada ao DCEI descartada	Ausência dos critérios anteriormente mencionados

*DCEI: dispositivo cardíaco eletrônico implantável.*

**Tabela 41:** – Critérios maiores e menores para diagnóstico de infecção relacionada a DCEI

CRITÉRIOS MAIORES	
Microbiologia	A. Hemoculturas positivas para microrganismos típicos encontrados na infecção relacionada ao DCEI e/ou EI (estafilococos coagulase-negativos, *S. aureus* )
	B. Microrganismos compatíveis com o EI em duas hemoculturas separadas:
	a. *Streptococus Viridans, Streptococcus gallolyticus* ( *S. bovis* ), grupo HACEK, *S. aureus*
	b. Enterococos adquiridos na comunidade, na ausência de um foco primário
	C. Microrganismos compatíveis com o EI de hemoculturas persistentemente positivas:
	a. ≥2 hemoculturas positivas de amostras de sangue colhidas >12 h
	b. Três amostras ou a maioria de ≥ 4 culturas de sangue separadas (primeira e última amostras coletadas com ≥ 1 hora de intervalo)
	c. Hemocultura positiva única para *Coxiella burnetii* ou título de anticorpo IgG de fase I >1: 800
Imagem positiva para infecção relacionada ao DCEI e/ou EI	A. Ecocardiograma (incluindo intracardíaco) positivo para:
	a. Infecção relacionada ao DCEI:
	I. Infecção clínica do gerador/loja
	II. Vegetação em cabo-eletrodo
	b. EI em válvula
	I. Vegetações
	II. Abscesso, pseudoaneurisma, fístula intracardíaca
	III. Perfuração valvar ou aneurisma
	IV. Nova deiscência parcial de prótese valvar
	B. FDG PET/CT (deve-se tomar cuidado em caso de implantes recentes) ou detecção radioativa de SPECT/CT WBC de atividade anormal no local da loja do gerador, ao longo dos condutores ou no local da válvula
	C. Vazamento para-valvar definido por TC cardíaca
**CRITÉRIOS MENORES **	
	a. Predisposição: como condições cardíacas predisponentes (p. ex., defeitos cardíacos estruturais preexistentes, insuficiência valvar tricúspide recente) ou uso de drogas injetáveis
	b. Febre (temperatura > 38°C)
	c. Fenômenos vasculares (incluindo os detectados apenas por imagem): embolia arterial importante, embolia pulmonar séptica, aneurisma infeccioso (micótico), hemorragia intracraniana, hemorragias conjuntivais e lesões de Janeway
	d. Evidência microbiológica: hemocultura positiva que não atende a um critério principal, como observado anteriormente, ou evidência sorológica de infecção ativa por organismo compatível com EI ou cultura de loja de gerador ou cultura de cabo-eletrodo (extraída por loja não infectada).

*EI: endocardite infecciosa; DCEI: dispositivo cardíaco eletrônico implantável; EIC: ecocardiograma com ultrassom intracardíaco; PET/CT: tomografia computadorizada por emissão de pósitrons; SPECT/CT WBC: tomografia computadorizada por emissão de fóton único (single photon emission computed tomography) com leucócitos marcados; lesões de Janeway: lesões hemorrágicas, indolores, embolização séptica.*

**Tabela 42:** – Recomendações para a remoção de DCEI

	Classe de recomendação	Nível de evidência
Remoção completa do sistema em pacientes com infecção relacionada ao DCEI (sistêmica ou local) confirmada	I	B
Remoção completa do DCEI nos casos de bacteremia por *S. aureus, CoNS, Cutibacterium* spp. ou fungemia por *Candida* spp., afastados outros sítios de infecção	I	C
Remoção completa do DCEI nos casos de bacteremia por *Pneumococcus* spp. ou bactéria gram-negativa que não *Pseudomonas* ou *Serratia* , quando houver bacteremia recorrente/persistente, apesar da antibioticoterapia apropriada, na ausência de outra fonte identificável da recorrência ou da persistência da infecção	I	C
Remoção completa do DCEI em pacientes com endocardite infecciosa, com ou sem envolvimento definitivo do sistema DCEI	IIa	C
Remoção completa do DCEI nos casos de bacteremia por *Streptococcus* sp. alfa ou beta-hemolítico ou *Enterococcus* spp. como tratamento de primeira escolha e, no caso de bacteremia recorrente/persistente, apesar da antibioticoterapia apropriada, como tratamento de segunda escolha	IIb	C

*DCEI: dispositivo cardíaco eletrônico implantável.*

O implante do novo DCEI deve ser realizado somente após remissão completa do processo infeccioso, e deve ser definido em função do quadro clínico. Até que o quadro infeccioso seja totalmente debelado, pacientes dependentes de estimulação artificial devem ser mantidos com MP temporário. Pacientes não dependentes devem permanecer sob monitoramento do ritmo cardíaco, até que o implante seja realizado. Em alguns casos, o implante do novo DCEI pode não ser necessário devido à alteração no padrão da doença ou por mudança de conduta. Por isso, a reavaliação da necessidade do DCEI é sempre fundamental. As recomendações relacionadas ao implante do novo DCEI estão listadas na Tabela 43 .

**Tabela 43:** – Recomendações para implante de novo DCEI, após extração.

	Classe de recomendação	Nível de evidência
Reavaliação da indicação para o reimplante após a extração do dispositivo	I	C
O novo dispositivo deve ser implantado, preferencialmente, em outro sítio, podendo ser contralateral, femoral ou epicárdico	I	C
O reimplante deve ser evitado ou postergado, sempre que possível, até que os sintomas e sinais de infecção sistêmica e local estejam resolvidos	IIa	C
Cabo-eletrodo com mecanismo de fixação ativa para estimulação cardíaca temporária, ipsilateral ao sistema removido, em pacientes dependentes de marca-passo enquanto aguardam o reimplante	IIa	C

*DCEI: dispositivo cardíaco eletrônico implantável.*

Vários fatores de risco para o desenvolvimento de infecção relacionada ao DCEI têm sido detectados. Esses fatores podem estar relacionados ao próprio indivíduo, a procedimentos médicos realizados ou ao próprio DCEI. Os principais fatores para infeção de DCEI estão listados na Tabela 44 .

**Tabela 44:** – Fatores de risco que predispõem à infecção cardíaca relacionada a DCEI

Fatores relacionados ao paciente
Doença renal terminal (DRT)^a^
História de infecção de dispositivo
Febre antes do implante
Uso de corticosteroides
Insuficiência renal^b^
Doença pulmonar obstrutiva crônica (DPOC)
Classe NYHA ≥ 2
Doenças de pele
Malignidade
Diabetes melito
Ponte de heparina
Insuficiência cardíaca (IC)
Anticoagulantes orais
**Fatores relacionados ao procedimento**
Duração do procedimento
Hematoma
Reposicionamento de cabos
Operador inexperiente^c^
Estimulação temporária
Troca do gerador/revisão/ *upgrade*
Troca do gerador
Antibioticoprofilaxia^d^
**Fatores relacionados ao dispositivo**
Cabo(s) eletrodo(s) epicárdico(s)
Loja abdominal
Presença ≥ 2 cabos-eletrodos
Dispositivo dupla-câmara

*DPOC: doença pulmonar obstrutiva crônica; DPOC: doença pulmonar obstrutiva crônica; DRT: doença renal terminal; IC: insuficiência cardíaca; NA: não disponível; NYHA: New York Heart Association. Parâmetros de risco que foram estatisticamente significativos para dados retrospectivos e prospectivos são mostrados. Análises restritas a dados prospectivos apenas para os mesmos parâmetros (se disponível) também são mostradas. Adaptada de Polyzos et al.^549 a^Taxa de filtração glomerular (TFG) < 60 mL/min ou depuração da creatinina (CrCL) < 60 mL/min. ^b^GFR ≤ 15 mL/min ou hemodiálise ou diálise peritoneal. ^c^ < 100 procedimentos anteriores. ^d^A estimativa do efeito combinado dos estudos randomizados foi de 0,26 [0,13, 0,52].*

**Figura 7:**
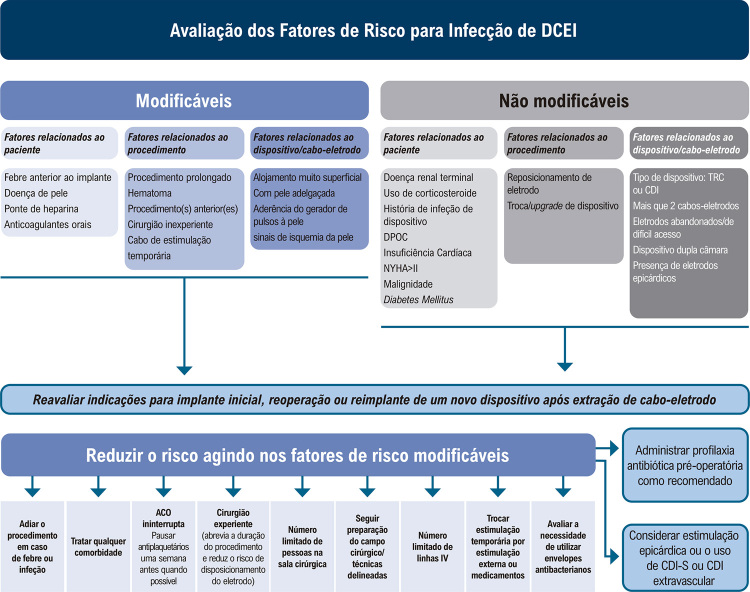
– Medidas preventivas para redução da infecção relacionada a DCEI considerando-se os fatores de risco, modificáveis ou não, já determinados na literatura. ACO: anticoagulação oral; CDI: cardioversor-desfibriador implantável; DCEI: dispositivo cardíaco eletrônico implantável; DPOC: doença pulmonar obstrutiva crônica; NYHA: New York Heart Association; TRC: terapia de ressincronização cardíaca.

Cuidados preventivos são fundamentais para a redução da ocorrência de infecções relacionadas a procedimentos médicos nesses indivíduos. Na Tabela 45 e na Figura 7 , estão listadas as principais medidas preventivas recomendadas,

**Tabela 45:** – Lista de medidas preventivas recomendadas para infecção relacionada ao DCEI

	Classe de recomendação	Nível de evidência
**Medidas pré-operatórias**		
Postergar o implante de DCEI em pacientes com infecção	I	C
Evitar estimulação transvenosa temporária e acesso venoso central sempre que possível. Caso utilizados, idealmente, devem ser removidos antes da introdução de nova prótese	I	A
Medidas para evitar hematoma de loja: interromper sempre que possível o uso de antiplaquetários e, diante de anticoagulantes orais, evitar a " ponte" com heparina, interrompendo o uso durante o implante, caso possivel	I	B
A profilaxia antibiótica é recomendada no espaço de tempo de 30 minutos a 1 hora antes da incisão cirúrgica para cefazolina e de 90 a 120 minutos para vancomicina	I	A
**Medidas perioperatórias**		
Uso de envelope antibiótico em situações de alto risco de infecção*	IIa	B
Instilar antisséptico e/ou antibióticos em loja de gerador	IIb	C
Medidas pós-operatórias		
Uso de antibioticoterapia pós-operatória	IIb	B
Drenagem ou evacuação do hematoma (exceto na presença de tensão tecidual, deiscência da ferida ou dor intensa)	III	B

* Conforme definido pelo estudo WRAP-IT:^550^ pacientes submetidos a revisão de loja ou cabo-eletrodo, troca de gerador, mudança de modo de estimulação, implante inicial de CRT-D e os com fatores de risco listados na Tabela 44. DCEI: dispositivo cardíaco eletrônico implantável.

**Tabela 46:** – Definições para abordagens e técnicas de extração

Técnica	Definição
**Transvenosa**	Extração de cabo-eletrodo por via intravascular (percutânea) realizada através de uma veia central (subclávia, jugular ou femoral)
- Abordagem pelo local de entrada do cabo-eletrodo na veia	Extração de cabo-eletrodo, por tração ou contratração, utilizando-se a mesma veia e local por onde o cabo-eletrodo foi introduzido
- Abordagem transjugular ou transfemoral	Extração com ferramenta de captura intravascular introduzida pelas veias jugular ou femoral, direita ou esquerda
**Transtorácica**	Extração de cabo-eletrodo com abertura da cavidade torácica por esternotomia mediana, toracotomia lateral, incisão subxifoide, inclusive por abordagem híbrida, com ou sem circulação extracorpórea

Idosos, crianças e adultos com cardiopatias congênitas representam subgrupos que merecem atenção especial quanto à possibilidade de infecção de DCEI. Loja submuscular em pacientes com pouco tecido subcutâneo para prevenção de erosão da pele é fundamental. Em pacientes pediátricos, especialmente com cardiopatia congênita, o operador deve ter experiência em abordagens cirúrgicas múltiplas e alternativas. CDI extravascular ou subcutâneo deve ser considerado em criança de menor idade, pacientes com cardiopatia congênita e com acesso venoso limitado ou inexistente.^551 , 552^

Curioso destacar que a análise de registros retrospectivos^553 , 554^ demonstra taxa de infecção de DCEI superior ao reportado em estudos prospectivos^537 , 555 - 558^ (3,4% *vs.* 1,2%). Este fenômeno pode estar relacionado à maior adesão a procedimentos preventivos nos estudos clínicos em comparação ao que ocorre na prática clínica diária. Dentre os fatores operatórios mais relacionados ao risco de infecção, destacam-se o hematoma de loja, os procedimentos de longa duração e as reintervenções para reposicionamento de cabo-eletrodo. Especificamente sobre reoperações para troca de gerador de pulsos, correção de disfunção de cabos-eletrodos ou mudança de modo de estimulação, o adequado tratamento da loja do gerador de pulsos, seja pela remoção completa da carapaça fibrosa ou pelo uso de envelope antibacteriano, reduz a ocorrência de infecções.

A profilaxia antibiótica pré-operatória, com o uso de uma dose de cefalosporina de primeira geração (cefazolina), é fortemente recomendável, o que não ocorre quanto ao uso sistemático de antibiótico no período pós-operatório.^11^

O intervalo de tempo entre o diagnóstico e o tratamento adequado da infecção relacionada a DCEI é fundamental. Dados da literatura mostram que se a remoção do dispositivo for feita até 3 dias após a hospitalização, tanto o tempo de internação quanto a mortalidade hospitalar são significativamente reduzidos. Nesse sentido, quando não existem dados suficientes para o estabelecimento do diagnóstico de infecção, baseada na tríade composta por sinais infecciosos na loja do gerador de pulsos, crescimento bacteriano nas hemoculturas e identificação de vegetações no ecocardiograma esofágico, outros recursos devem ser utilizados.

Recomenda-se a varredura por [18F] PET/CT FDG ou cintilografia WBC radiomarcada ou TC com contraste se houver suspeita de endocardite infecciosa relacionada ao DCEI, hemoculturas positivas e ecocardiografia negativa ou nos casos de bacteremia por *S. aureus* na presença de DCEI. Punção aspirativa e desbridamento cirúrgico em casos de infecção da loja do gerador, como tentativa para evitar a extração de cabo-eletrodo, devem ser fortemente desencorajados.

Para monitorar o número de casos de infecção relacionada a DCEI e o resultado das atitudes preventivas e terapêuticas, é fundamental a implementação de grandes registros, não voluntários, de fácil preenchimento e com grande qualidade. Cada serviço deve estabelecer rotinas para o diagnóstico preciso e tratamento em tempo adequado. A reavaliação constante do desempenho de cada centro é altamente recomendável.

### 7.2. Remoção de Cabos-eletrodos de Dispositivos Cardíacos Eletrônicos Implantáveis

A necessidade de remover cabos-eletrodos de DCEI cresceu nos últimos anos em função, principalmente, de dois fatores: 1) crescimento da taxa de infecções relacionadas a DCEI e 2) desenvolvimento dos MP e CDI multissítios, que utilizam maior número de cabos-eletrodos.

As indicações para a remoção de cabos-eletrodos podem ser: (a) obrigatórias, como no tratamento de infecções; (b) necessárias, para obtenção de acesso para novos cabos-eletrodos em pacientes com oclusões venosas; ou (c) opcionais, como em pacientes com acesso venoso adequado, quando são submetidos à substituição de cabo-eletrodo.

Como a maioria dos dispositivos necessita de acesso venoso para o implante dos cabos-eletrodos, as técnicas de extração transvenosa são as mais utilizadas. Na atualidade, a abertura do tórax para a remoção de cabos-eletrodos é muito pouco utilizada, sendo necessária, quase exclusivamente, para a retirada de cabos-eletrodos epicárdicos ou para a correção de complicações que ocorrem em extrações transvenosas.

O panorama atual da extração de cabos-eletrodos mostra indicações e técnicas operatórias bem estabelecidas. As ferramentas utilizadas estão bem desenvolvidas e os resultados dos diferentes procedimentos são bem conhecidos, com altas taxas de sucesso. Complicações catastróficas, entretanto, podem ocorrer durante procedimentos de extração. Tais complicações, embora raras, são potencialmente letais e costumam requerer cirurgia a céu aberto de emergência.

Neste item, são apresentadas as recomendações para extração em pacientes não infectados, uma vez que o manejo de infecções já foi abordado.

A remoção de cabos-eletrodos epicárdicos é feita, obrigatoriamente, pela reabertura da cavidade torácica, preferencialmente pelo mesmo acesso por onde o eletrodo foi implantado. A remoção de cabos-eletrodos transvenosos deve ser feita, preferencialmente, por acesso intravascular. Excepcionalmente, pode ser necessária uma abordagem transtorácica, com ou sem o auxílio de circulação extracorpórea, como na falha da extração transvenosa ou quando existem grandes vegetações aderidas aos cabos-eletrodos. A escolha do tipo de abordagem para a extração transvenosa de cabos-eletrodos depende, fundamentalmente, da possibilidade de se obter acesso ao cabo-eletrodo alvo da remoção. Infelizmente, muitos pacientes são portadores de cabos-eletrodos completamente intravasculares, por ter apresentado fratura espontânea do cabo ou por iatrogenia durante procedimentos de remoção.

Quando o cabo-eletrodo a ser removido está íntegro ou apresenta um segmento extravascular, por menor que esse segmento seja, devemos utilizar a abordagem pelo local de entrada do eletrodo na veia ( *venous entry site approach* ). Essa abordagem consiste na introdução na veia de uma bainha que é guiada pelo próprio cabo-eletrodo a ser removido. Essa bainha é utilizada para desfazer as aderências que se formam entre o cabo-eletrodo e o endotélio venoso ou o endocárdio. Quando o paciente tem mais que um cabo-eletrodo transvenoso implantado, frequentemente, existem aderências entre os cabos. Após se desfazerem todas as aderências e se atingir o local onde o eletrodo está fixado ao coração, essa mesma bainha é utilizada para se fazer pressão contra o músculo cardíaco enquanto se traciona o cabo-eletrodo (manobra de contratração). Existem várias ferramentas especificamente desenvolvidas para esse tipo de abordagem, a saber:

Guias de travamento ( *locking stylets* ), que são estiletes revestidos por uma fina malha de aço que se expande na luz do cabo-eletrodo conferindo a este a sustentação necessária para sua tração.
Bainhas para dissecção de aderência e contratração:
*Bainhas mecânicas não energizadas* ( *non-powered sheaths* ): conjuntos de tubos rígidos metálicos e tubos flexíveis de teflon ou polipropileno, utilizados para desfazer aderências por dissecção romba, com intensidade determinada pela força da mão do médico que faz o procedimento.
*Bainhas mecânicas com lâminas rotacionais:* seccionam as aderências e são ativadas por um gatilho na mão do operador ou por um motor elétrico.
*Bainhas energizadas por raios LASER* : seccionam as aderências por foto-ablação.

Quando o cabo-eletrodo que precisa ser removido não apresenta um segmento extravascular, torna-se obrigatória a sua captura intravascular. Existem ferramentas com o formato de laço ou de cesta, feitas com arames metálicos muito maleáveis, desenvolvidas para capturar esses fragmentos. Tais ferramentas são introduzidas habitualmente por punção das veias femoral ou jugular. Uma vez capturado, o cabo-eletrodo pode ser tracionado diretamente. Em casos específicos, pode ser necessário associar manobra de contratração após a captura do cabo-eletrodo.^559^ As Tabelas 46 e 47 mostram as definições recomendadas para as ferramentas e para as abordagens usadas em extração de cabos-eletrodos.

O termo *remoção* tem sido utilizado, genericamente, para designar a retirada de cabos-eletrodos de DCEI, independentemente do tipo de abordagem. Pode ser realizada pela simples tração de um cabo-eletrodo transvenoso, sem a utilização de nenhuma ferramenta; por toracotomia, para a retirada de cabo-eletrodo epicárdico ou por toracotomia com o auxílio de circulação extracorpórea para a retirada de cabo-eletrodo transvenoso. O termo *extração* deve ser utilizado exclusivamente para os casos em que técnicas e ferramentas são utilizadas para: 1) dilatar o trajeto por onde o cabo-eletrodo passa no interior das veias; 2) desfazer aderências; 3) realizar manobra de contratração ou 4) capturar fragmentos de cabos-eletrodos no interior dos vasos ou das cavidades cardíacas.

A conclusão de um procedimento de remoção ou de extração de cabo-eletrodo pode resultar em: 1) retirada completa do cabo-eletrodo-alvo; 2) retirada parcial; ou 3) insucesso da retirada. Dependendo do tipo de indicação para a retirada do cabo-eletrodo, pode-se considerar que houve sucesso clínico do procedimento, mesmo que nem todo o cabo-eletrodo tenha sido removido. Considera-se que houve falha do procedimento quando: 1) não é obtido o sucesso clínico; 2) ocorre qualquer complicação permanentemente incapacitante ou 3) ocorre a morte do paciente. Na Tabela 48 , estão listadas as definições estabelecidas pelo Consenso EHRA 2018 com a devida concordância das demais sociedades representativas dessa área do conhecimento.

**Tabela 47:** – Definições usadas para as diversas ferramentas de extração

Ferramentas e técnica	Definição
**Bainha para dissecção e contratração**	
- Mecânica não energizada	Conjuntos compostos por tubos rígidos metálicos e tubos flexíveis de teflon ou polipropileno
- Mecânica rotacional^548^	Lâminas cortantes metálicas movidas por ativação manual ou elétrica
- LASER^560^	Construídas com microtubos de vidro, condutores da luz produzida por fonte (externa) de emissão do Excimer-LASER
**Guia de travamento (Locking stylet)**	Estiletes revestidos por malha de aço que dão rigidez e sustentação aos cabos-eletrodos
**Ferramentas de captura intravascular**	Cateteres em forma de laço^561^ ( *snares* ), cesta ( *baskets* ) e outras ferramentas utilizadas para capturar fragmentos intravasculares
**Balões para oclusão** ^562^ ** e dilatação vascular**	Conjuntos compostos por guias e balões para dilatação ou oclusão venosa.
**Acessórios complementares**	Extensor de cabo, espiral de compressão^563^

**Tabela 48:** – Definições para procedimentos realizados e resultados da remoção de cabos-eletrodos

Conceito	Definição
**Procedimento**	
Remoção de cabo-eletrodo	Retirada de cabo-eletrodo realizando-se apenas a tração direta, sem utilização de ferramentas
wExtração de cabo-eletrodo	Retirada de pelo menos um cabo-eletrodo com o uso de ferramentas (listadas na Tabela 47)
**Resultado**	
**Relacionado ao cabo-eletrodo**	
- Retirada completa do cabo-eletrodo	Remoção ou extração de cabo-eletrodo com sua retirada integral
- Retirada incompleta do cabo-eletrodo	Remoção ou extração de cabo-eletrodo em que parte deste fica retida no paciente
**Relacionado ao paciente**	
- Sucesso cirúrgico completo	Retirada de todos os cabos-eletrodos alvos do procedimento, sem qualquer complicação permanentemente incapacitante ou morte relacionada ao procedimento
- Sucesso clínico	Retenção de uma pequena parte de um cabo-eletrodo, que não afete negativamente o resultado do procedimento. Esse fragmento pode ser a ponta ou uma pequena parte (<4cm) do cabo-eletrodo (condutor, isolante ou ambos), desde que esse não represente risco de perfuração, evento embólico ou perpetuação de infecção, na ausência de qualquer complicação permanentemente incapacitante ou morte relacionada ao procedimento
- Falha do procedimento	Impossibilidade de alcançar sucesso clínico, ocorrência de qualquer complicação permanentemente incapacitante ou morte relacionada ao procedimento.

Diversos motivos podem justificar a desativação de um cabo-eletrodo: 1) perda da capacidade de estimular adequadamente o coração; 2) necessidade de se mudar o tipo de dispositivo e 3) problemas relacionados à sua fabricação.

Cabos-eletrodos não infectados podem ser abandonados *in loco* a critério da equipe médica que opera o paciente. Existem, entretanto, desvantagens de se abandonar um cabo-eletrodo: 1) risco de fenômenos trombóticos; 2) restrições para realização de exames de RM; 3) aumento do risco em extração futura, uma vez que, quanto maior for o tempo de permanência do cabo-eletrodo, maior será o risco de insucesso da extração. O principal argumento para se abandonar *in loco* um cabo-eletrodo não infectado é o risco de complicações graves associadas ao procedimento de extração. Uma publicação recente mostra que a expectativa de sobrevivência no primeiro ano que se segue a um procedimento de troca de eletrodo é semelhante para os casos em que se realiza a extração ou o abandono *in loco* do cabo-eletrodo.^564^ Diante disso, na atualidade, a decisão de se extrair ou não um cabo-eletrodo que será desativado depende, essencialmente, da *expertise* de cada serviço. Na Tabela 49 , encontram-se as definições estabelecidas pelo consenso EHRA 2018 para cabos-eletrodos não infectados.

**Tabela 49:** – Definições de termos para cabos eletrodos não infectados

Cabos eletrodos não infectados	Definições
Função do cabo-eletrodo	Qualquer função do cabo-eletrodo, incluindo estimulação, sensibilidade e/ou desfibrilação
Falência do cabo-eletrodo	Perda total da função do cabo-eletrodo
Disfunção do cabo-eletrodo	Cabo-eletrodo não utilizável para estimulação e/ou desfibrilação devido à perda de sua integridade funcional
Cabo-eletrodo abandonado	Cabo-eletrodo deixado no coração e não conectado a um DCEI. Pode ser normofuncionante ou não
Recall^558,565-568^	Retirada ou desativação de um cabo-eletrodo por problemas relacionados à sua fabricação, por exigência de agências regulatórias ou por orientação voluntária do fabricante
- *Recall* classe 1	Produtos perigosos ou defeituosos com probabilidade razoável de causar sérios problemas de saúde ou morte (p. ex., curto-circuito sem aviso)
- *Recall* classe 2	Produtos que podem causar um problema de saúde temporário ou com baixa probabilidade de causar ameaça de natureza grave (p. ex., esgotamento prematuro da bateria)
- Recall classe 3	Produtos com pouca probabilidade de causar qualquer reação adversa à saúde, mas que violam as normas regulatórias

*DCEI: dispositivo cardíaco eletrônico implantável.*

A durabilidade de um DCEI depende tanto de aspectos relacionados à sua fabricação (material e/ou desenho) quanto de seu modo de utilização. Cabos-eletrodos, especificamente, sofrem influência direta da técnica operatória utilizada, que pode influenciar negativamente sua durabilidade. Independentemente desses fatos, estratégias específicas de tecnovigilância devem ser adotadas pelos fabricantes e pelas agências regulatórias para avaliar a durabilidade dos componentes dos DCEI.

Ao se optar pelo abandono *in loco* de cabos-eletrodos por disfunção, necessidade de mudança do modo de estimulação ou, de maneira inadequada, para o tratamento de uma infecção relacionada a DCEI, cuidados devem ser seguidos, uma vez que a forma como esse cabo-eletrodo é abandonado pode dificultar sua extração no futuro. Recomendações para o abandono de cabos-eletrodos estão listadas na Tabela 50 .

**Tabela 50:** – Recomendações para o manuseio de cabos-eletrodos abandonados, desnecessários ou disfuncionais

	Classe de recomendação	Nível de evidência
Ao abandonar um cabo-eletrodo, deve-se deixá-lo em condição que evite sua retração dentro da veia e que permita sua extração no futuro	I	C
Diante de um cabo-eletrodo clinicamente desnecessário ou disfuncional, tanto a opção de abandono como a de extração podem ser consideradas dentro da estratégia cirúrgica^569-571^	IIa	B

Em determinadas situações clínicas, a retirada de um cabo-eletrodo não infectado pode ser obrigatória, tais como: 1) para o tratamento da síndrome da veia cava superior provocada pela presença de cabos-eletrodos; 2) para o tratamento de arritmia cardíaca grave provocada mecanicamente por um fragmento de cabo-eletrodo; 3) para evitar uma lesão cardíaca por um cabo-eletrodo fraturado ou 4) para permitir o tratamento radioterápico na região em que o dispositivo está implantado.

Outras vezes, a extração de um cabo-eletrodo pode ser necessária, como nos casos em que oclusão ou obstrução venosa grave impedem a passagem de um novo cabo-eletrodo.

Em muitos casos, entretanto, a extração dos cabos-eletrodos é opcional, e pode ser definida por um conjunto de fatores menos objetivos, tais como: 1) idade do paciente ou sua expectativa de vida; 2) necessidade futura de realizar exames de RM; 3) risco de desenvolver obstruções venosas graves; ou 4) risco de infecção pela via hematogênica, como ocorre em pacientes com insuficiência renal dialítica. Nessas ocasiões, a *expertise* do profissional que realiza o procedimento é mandatória para a definição da conduta. Na Tabela 51 , estão listadas as recomendações para a extração de cabos-eletrodos não infectados.

**Tabela 51:** – Recomendações para a retirada de cabos-eletrodos por causas não infecciosas

	Classe de recomendação	Nível de evidência
Trombose/Comprometimento vascular		
Eventos embólicos clinicamente significativos atribuíveis à presença de cabo-eletrodo ou fragmento deste^572-574^	I	C
Estenose ou oclusão de VCS^575^ que impeça a passagem de um novo cabo-eletrodo	I	C
Necessidade de tratamento com *stent* para evitar o aprisionamento do cabo-eletrodo^576,577^	I	C
**Outros**		
Arritmias induzidas por cabo-eletrodos ou fragmento^578^	I	C
Dispositivo implantado em local que interfira no tratamento de uma doença maligna^57^9	IIa	C
Superpopulação de eletrodos: > 4 eletrodos de um lado ou > 5 eletrodos através da VCS^569,580,581^	IIa	C
Eletrodos abandonados que causem interferência no funcionamento de um dispositivo implantado^582,583^	IIa	C
Eletrodos que, devido ao seu *design* ou falha, representam uma ameaça ou malefício ao paciente, se deixados no local^584-587^	IIb	C
Condições que contraindiquem a ressonância magnética: cabos-eletrodos ou fragmentos abandonados; cabos-eletrodos não condicionais para ressonância magnética^588-593^	IIb	C
Retirada definitiva do dispositivo por decisão compartilhada	IIb	C

*VCS: veia cava superior.*

Durante um procedimento de extração transvenosa de cabos-eletrodo, veias ou estruturas cardíacas podem ser lesionadas. Lesões das veias axilares ou subclávias, dos troncos venosos braquiocefálicos ou da veia cava superior podem provocar hemorragia grave com necessidade de transfusão sanguínea ou até mesmo correção cirúrgica. Avulsão muscular do átrio direito ou do VD, assim como perfuração de veia tributária do seio coronário, pode acarretar tamponamento cardíaco. A laceração da veia cava superior em seu trajeto extrapericárdico, entretanto, é a complicação catastrófica mais frequente e mais letal. Outras complicações, como arritmias cardíacas autolimitadas, pneumotórax ou retenção de fragmentos de cabos-eletrodos, também podem ocorrer e necessitar de cuidados específicos.

De maneira geral, as complicações são agrupadas em maiores e menores, em função de sua gravidade e do tipo de correção que demandam. Na Tabela 52 , estão listadas as complicações conforme sua classificação e incidência.^577^

**Tabela 52:** – Classificação e incidência das complicações perioperatórias mais frequentes*

Complicações	Incidência %
**Maiores**	**0,19–1,80**
Morte	0,19–1,20
Avulsão cardíaca	0,19–0,96
Laceração vascular	0,16–0,41
Parada respiratória	0,20
Acidente vascular cerebral	0,07–0,08
Derrame pericárdico que requer intervenção	0,23–0,59
Hemotórax que requer intervenção	0,07–0,20
Parada cardíaca	0,07
Tromboembolismo que requer intervenção	0,07
Lesão valvar tricúspide com repercussão hemodinâmica grave	0,03
Embolia pulmonar maciça	0,08
**Menores **	**0,06–6,20**
Derrame pericárdico sem necessidade de intervenção	0,07–0,16
Hematoma que requer drenagem	0,90–1,60
Trombose venosa que requer intervenção médica	0,10–0,21
Necessidade de reparo vascular no local do acesso venoso	0,07–0,13
Migração de fragmento de cabo-eletrodo sem sequelas	0,20
Sangramento que requer transfusão de sangue	0,08–1,00
Fístula arteriovenosa que requer intervenção	0,16
Pneumotórax que requer drenagem torácica	1,10
Agravamento da insuficiência valvar tricúspide	0,02–0,59
Embolia pulmonar	0,24–0,59

* Fonte: adaptada de Kusumoto et al., 2017.^594^

Vários estudos têm sido desenhados para identificar fatores de risco determinantes da morbimortalidade relacionada à extração transvenosa de cabos-eletrodos. Esses estudos têm demonstrado baixa taxa de ocorrência de complicações catastróficas e de morte perioperatória, não permitindo a identificação de fatores de risco para esses eventos. Por outro lado, vários fatores demográficos, clínicos e cirúrgicos estão associados à mortalidade nos 30 primeiros dias que se seguem a um procedimento de extração. Fatores associados a complicações e morte tardias também foram identificados.^30 , 595 - 603^ Na Tabela 53 , estão listados os fatores de risco já identificados e seu impacto na morbimortalidade.

**Tabela 53:** – Fatores de risco para morte e complicações associadas à remoção de cabos-eletrodos

Fator	Risco associado ao fator
Idade	Aumenta a mortalidade em 1,05 vez
Sexo feminino	Aumenta o risco de complicações maiores em 4,5 vezes
Baixo índice de massa corporal (< 25kg/m^2^)	Aumenta a mortalidade de 30 dias em 1,8 vez Aumenta o número de complicações relacionadas à extração
História de AVC	Aumenta o risco de complicações maiores em 2,0 vezes
Disfunção grave do VE	Aumenta o risco de complicações maiores em 2,0 vezes
IC avançada	Aumenta o risco de mortalidade em 30 dias de 1,3 a 8,5 vezes Aumenta a mortalidade em 1 ano de seguimento em 3,0 vezes
Disfunção renal	Disfunção renal terminal aumenta o risco de morte em 30 dias em 4,8 vezes Creatinina > 2,0 aumenta a mortalidade hospitalar e mortalidade em 1 ano em 2,0 vezes
Diabetes melito	Aumenta a mortalidade hospitalar e mortalidade geral em 1,7 vez
Coagulopatia	INR elevado aumenta risco de complicações maiores em 2,7 vezes e a mortalidade de 30 dias em 1,3 vez Uso de anticoagulantes aumenta mortalidade de 1 ano em 1,8 vez
Contagem de plaquetas	Plaquetopenia aumenta risco de complicações maiores em 1,7 vez
Anemia	Aumenta 3,3 vezes o risco de morte em 30 dias
Número de cabos-eletrodos extraídos	Aumenta 3,5 vezes o risco de qualquer complicação Aumenta em 1,6 vez a mortalidade a longo prazo
Presença de CDI de duplo *coil*	Aumenta 2,7 vezes a mortalidade em 30 dias
Extração por infecção	Aumenta em 2,7 a 30 vezes a mortalidade em 30 dias Aumenta de 5,0 a 9,7 vezes a mortalidade em 1 ano PCR >7,2mg/L aumenta a mortalidade em 30 dias Aumenta 3,5 vezes a mortalidade geral
Experiência do operador	Aumenta 2,6 vezes a número de complicações relacionadas ao procedimento
Cirurgia cardíaca prévia	Reduz a incidência de complicações maiores

*AVC: acidente vascular cerebral; CDI: cardioversor-desfibrilador implantável; IC: insuficiência cardíaca; INR: Razão Normalizada Internacional; PCR: proteína C reativa; VE: ventrículo esquerdo.*

Diante da dificuldade de se prever a ocorrência de complicações catastróficas perioperatórias, torna-se fundamental a prevenção da morte relacionada a esses eventos, o que implica treinamento dos profissionais envolvidos e capacitação técnica dos serviços que realizam extração de cabos-eletrodo. Uma revisão sistemática recente^604^ mostra a estreita relação entre o número de procedimentos realizados pelo médico e a taxa de complicações associadas à extração de eletrodos. Operadores em início de experiência devem realizar os primeiros 40 procedimentos de extração transvenosa sob a supervisão de operadores mais experientes. Recomenda-se um volume mínimo de 20 procedimentos de extração transvenosa por ano, a fim de manutenção da capacidade técnica, para todos os operadores.

## 8. Recomendações para prevenção de interferências eletromagnéticas

### 8.1. Cirurgia com Uso de Eletrocautério

A eletrocirurgia compreende o uso de corrente alternada de alta frequência (200 KHz a 2,2 MHz), que é convertida em calor ao sofrer resistência ao passar pelos tecidos, o que permite efetuar os efeitos desejáveis: coagulação e corte. O bisturi elétrico é usado na maioria das especialidades cirúrgicas.

Por ser mais efetiva, a eletrocirurgia monopolar é a mais empregada na prática. Nessa modalidade, o eletrodo ativo está no sítio cirúrgico (haste do bisturi), enquanto o eletrodo indiferente é uma placa colocada em contato com a pele do paciente, em local afastado. A corrente flui entre os eletrodos, atravessando o corpo.

Um número crescente de pacientes com DCEI é submetido a intervenções cirúrgicas, o que expõe esses pacientes às interferências eletromagnéticas. A eletrocirurgia monopolar pode provocar várias anormalidades nos DCEI, tais como reprogramação do gerador de pulsos, inibição temporária da estimulação, deflagração de estimulação em frequência elevada, depleção da bateria e falha de estimulação, dano ao circuito, aumento de limiares, e disparo de terapia inapropriada (choques) em caso de CDI.^605^

Para minimizar os riscos do uso do eletrocautério, alguns cuidados devem ser tomados no perioperatório: (1) a aplicação do bisturi monopolar deve ser intermitente, em pulsos de curta duração e com menor energia; (2) a placa indiferente deve ser posicionada em local de modo que a energia não flua através do gerador ou eletrodos.

Em geral, quando o sítio cirúrgico é localizado acima da cicatriz umbilical ou a uma distância menor que 15cm do gerador, o uso de bisturi monopolar deve ser evitado. Nesse cenário, deve-se preferir o bisturi bipolar, que é seguro, mas que também não deve ser aplicado diretamente sobre o gerador.^606 , 607^ Em cirurgia de cabeça e pescoço, a placa indiferente do bisturi monopolar deve ser colocada na parte posterior do ombro contralateral à da loja do dispositivo. Assim, por exemplo, no caso de gerador implantado na região infraclavicular esquerda, a placa do bisturi deve ser colocada sob o ombro direto.

Com a finalidade de proteger o paciente e o DCEI dos efeitos indesejáveis do eletrocautério, duas abordagens têm sido empregadas: aposição de magneto sobre o gerador de pulsos e reprogramação do dispositivo antes do procedimento. Em caso de MP, o uso do magneto durante a cirurgia é opção quando o gerador desativar o circuito de sensibilidade sob efeito magnético (modo assíncrono) e apresentar bateria em bom estado.^608 , 609^ A aplicação do ímã sobre a loja do gerador causa a reversão do MP para o modo assíncrono, ou seja, desabilita a sensibilidade e modifica a frequência de estimulação para a frequência magnética, que é geralmente maior que a frequência básica programada.

Em caso de CDI, um estudo randomizado comparou a aplicação do magneto *versus* a reprogramação em pacientes com CDI submetidos à cirurgia utilizando eletrocautério monopolar, a uma distância maior de 15 cm do gerador; os autores concluíram que as duas estratégias são seguras.^610^ A aplicação do magneto sobre a loja do CDI desabilita somente a terapia para as taquiarritmias, não alterando a função de MP. No paciente com CDI e dependente de MP, o dispositivo deve ser reprogramado para modo assíncrono, antes do procedimento. A reprogramação do DCEI deve ser realizada logo antes da intervenção cirúrgica e revertida para a programação basal logo após o término do procedimento ( Tabela 54 ).

**Tabela 54:** – Recomendações para prevenção de interferências eletromagnéticas em cirurgia com uso de eletrocautério

	Classe de recomendação	Nível de evidência
A eletrocirurgia monopolar pode provocar várias anormalidades nos DCEI, e deve-se usar, preferencialmente, o eletrocautério bipolar No paciente com CDI e não dependente de marca-passo, pode-se realizar a aplicação do magneto sobre a loja do CDI com segurança, para desabilitar as terapias para as taquiarritmias e evitar choques inapropriados	I	A
Em caso de marca-passo, o uso do magneto durante a cirurgia é uma opção quando o gerador desativar o circuito de sensibilidade sob efeito magnético (modo assíncrono) e apresentar bateria em bom estado	I	B
Em cirurgia de cabeça e pescoço, a placa indiferente, em se tratando de bisturi monopolar, deve ser colocada na parte posterior do ombro contralateral à da loja do dispositivo No paciente com CDI e dependente de marca-passo, as terapias são desativadas, e o dispositivo deve ser reprogramado para um modo assíncrono antes do procedimento A reprogramação do DCEI deve ser realizada logo antes da intervenção cirúrgica e revertida para a programação basal logo após o término do procedimento	I	C
Em casos de cirurgias em sítio acima do umbigo, deve-se utilizar um bisturi bipolar. Se não for possível evitar o uso do bisturi monopolar, o gerador deve ser reprogramado	IIa	C

*CDI: cardioversor-desfibrilador implantável; DCEI: dispositivos cardíacos eletrônicos implantáveis.*

### 8.2. Ressonância Magnética

A ressonância magnética (RM) tem se apresentado como ferramenta diagnóstica cada vez mais útil e acessível, com crescente relevância para avaliação diagnóstica e prognóstica.

O número de exames de RM apresentou um crescimento substancial nos últimos 20 anos, com mais de 60 milhões de exames anualmente ao redor do mundo. Estima-se que um paciente tem 50% a 75% de probabilidade de receber indicação de realização de RM após implante de um DCEI durante o tempo de vida do dispositivo.^594 , 611^

O ambiente de realização do exame pode ser dividido em zonas, conforme descrito por Kanal *et al* .^612^ e adotado pelo *2017 HRS expert consensus.* ^593^ A zona 4 refere-se à sala do exame, sendo o espaço com maior risco para pacientes e equipe de saúde, incluindo o risco potencial de deslocamento de objetos metálicos. A zona 3 é o espaço fora da sala de exame, incluindo a sala de controle. Existe, nessa área, um risco potencial, devendo ser reservada a pessoal treinado. A zona 2 inclui área de recepção, e a zona 1 corresponde às regiões acessíveis ao público geral.

Com relação aos DCEI durante a realização de RM, podem ser definidos como:^613^

*Seguros* : não representam perigo para realização da RM.
*Condicionados* : não representam perigo para realização da RM, desde que atendidas determinadas especificações. Tais condições podem incluir parâmetros como: região do corpo a ser examinada, força do campo magnético, gradiente espacial, tempo de exposição ao campo magnético, campo de radiofrequência e taxa de absorção específica. Condições adicionais podem ser requeridas, incluindo uso de combinações específicas de gerador e eletrodos, bem como modo de programação do dispositivo. As condições específicas podem variar entre fabricantes e entre aparelhos de um mesmo fabricante.
*Não condicionados* : representam perigo para realização da RM. Incluem todos os sistemas de estimulação cardíaca que não são condicionados para realização do exame. Isso inclui geradores condicionados associados a eletrodos não condicionados ou sistemas totalmente condicionados implantados em pacientes que não preenchem todas as condições específicas de uso, como aqueles com eletrodos abandonados.

Os DCEI não são classificados como *seguro* , e os novos DCEI que foram construídos com tecnologia apropriada são considerados *condicionados* para realização do exame.

Na prática clínica, os aparelhos precisam ser programados antes da realização de RM, com um nível de segurança bastante aceitável.

A interação do campo magnético estático, gradiente magnético e radiofrequência sobre o DCEI pode prejudicar o funcionamento de componentes eletrônicos, causar migração ou deslocamento de componentes do sistema, geração de corrente de energia que pode danificar o aparelho e/ou o miocárdio e provocar *oversensing, undersensing* e arritmias. A influência desses parâmetros nos DCEI pode ser dividida em dois grupos: os que prejudicam o funcionamento do DCEI de maneira transitória e os que o fazem de modo permanente.^614 , 615^

As respostas a essas fontes de interferência podem ser variadas:

*Campo magnético estático* : deslocamento do dispositivo, ativação de sensores, perda súbita da função do dispositivo, mudanças no ECG.
*Gradiente de campo magnético* : indução de arritmias (raro); o *versensing* ou *undersensing* .
*Campo de radiofrequência* : aquecimento tissular adjacente aos eletrodos; indução de arritmias (raro); reprogramação do dispositivo ( *reset* ); interações de *oversensing* ou *undersensing* .
*Efeitos combinados* : perda súbita da função do dispositivo; alteração de função (parâmetros); forças mecânicas (vibração); *reset* do dispositivo; dano do gerador e/ou dos eletrodos.
*Relacionados à imagem* : artefatos que prejudiquem a imagem adequada do dispositivo.

As interações potenciais entre DCEI e interferência eletromagnética pela RM incluem:

*Campo magnético induzido e torque devido a materiais ferromagnéticos* : movimento de gerador é extremamente improvável pelo confinamento e pelo tecido subcutâneo adjacente. Eletrodos não contêm material ferromagnético suficiente para causar movimentos.
*Corrente elétrica induzida por gradiente de campo magnético* : gradientes de campo magnético podem induzir corrente, o que pode levar a captura miocárdica e, potencialmente, causar arritmias atriais ou ventriculares.
*Calor e dano tecidual* : campos de radiofrequência podem levar a aquecimento de componentes não condicionados, levando a aquecimento e dano térmico ao tecido adjacente (ablação funcional). Mudanças de sensibilidade e limiar de captura podem ocorrer como resultado do dano tecidual próximo aos eletrodos.
*Efeitos na atividade do dispositivo* : o dispositivo de estimulação cardíaca pode ser programado por meio da colocação de um ímã, liberando o dispositivo para a interação. Os campos magnéticos podem, portanto, afetar a atividade de um dispositivo condicional, com possibilidade de alterar a programação do dispositivo.
*Reset* *elétrico* : a interferência eletromagnética de alta energia pode levar a *reset* elétrico. Pode ocorrer acionamento de um modo de demanda de *backup* . Os parâmetros de *reset* de energia variam de acordo com o fornecedor e o tipo de dispositivo e podem incluir um conjunto de variações. Inibição da função de estimulação por sinais gerados por RM ou energia de estimulação abaixo do limiar (bipolar ou unipolar) em um paciente dependente de MP pode ocorrer. Além disso, o *status* da bateria pode ser afetado, principalmente para dispositivos próximos de um intervalo de substituição eletiva (ERI), que pode resultar em funções não confiáveis.
*Funções inapropriadas e terapias* : interferências eletromagnéticas por pulsos de radiofrequência ou mudanças rápidas de gradientes de campo magnético podem causar *oversensing* que podem levar à inibição inapropriada da estimulação e possibilidade de assistolia em pacientes dependentes de estimulação do MP, ou indução de terapias levando a choques inapropriados em pacientes com CDI.

Esses efeitos são influenciados por vários fatores, incluindo força do campo magnético, potência de RF, posição do paciente e do dispositivo em relação à máquina de RM, características do dispositivo e tamanho do paciente.

Tradicionalmente, a realização de RM em portadores de DCEI costumava ser contraindicada. O primeiro sistema condicionado para a realização de RM foi introduzido na Europa em 2010 e liberado pelo Food and Drug Administration (FDA) em 2011, nos EUA.^613 , 616^

Para tornarem-se condicionados para RM, os DCEI sofreram mudanças estruturais (p. ex., uso de materiais não ferromagnéticos) e alterações de *software* que reduzem ou eliminam potenciais efeitos adversos. Uma vez acionada a programação especial (modo RM), o aparelho reverte o modo de estimulação para assíncrono e aumenta a energia de estimulação para evitar inibição da estimulação ou falha de captura durante o exame. Nos CDI, a função antitaquicardia é desabilitada temporariamente. No momento do exame, portanto, esses pacientes estão desprotegidos em caso de arritmias ventriculares.

A decisão de realizar RM em um paciente com DCEI envolve riscos e benefícios; assim, os fatores potenciais de risco devem ser identificados. Em pacientes com DCEI condicionais, o exame pode ser realizado sem riscos adicionais ao paciente, seguindo recomendações e protocolos estabelecidos.^617 , 618^

Antes da realização do exame, é importante identificar o ritmo de base do paciente e se o paciente é dependente de MP, ativar a programação específica para a realização de RM; confirmar se todo o sistema é condicional para RM; verificar presença de eletrodos abandonados ou epicárdicos.

De modo geral, a maioria dos sistemas é aprovada para exames de RM de 1,5T, gradiente com taxa de variação de 200 T/m/s, SAR máxima de SAR 2 W/kg, número limitado de sequências e comprimento de imagens. Em novos dispositivos, o exame pode ser também seguro sob condições mais amplas. A maioria dos sistemas novos permite o exame de RM do corpo inteiro.

Um sistema condicional de RM consiste em uma combinação de eletrodos e gerador que foi especificamente testada para garantir condições de uso seguras durante o exame. A presença de qualquer componente do dispositivo que não atenda aos critérios de condicionalidade de RM o classifica como não condicional. Isso inclui um gerador condicional de RM combinado com componentes não condicionais e sistemas de dispositivos que combinam componentes individuais de eletrodos e gerador condicionais de RM de vários fabricantes, dado que essas não são combinações especificamente testadas em conjunto para a segurança do exame.

A rotulagem de condicional também especifica a localização do gerador: localização peitoral para sistemas transvenosos. Outros exemplos de componentes não condicionais incluem eletrodos epicárdicos, eletrodos abandonados, eletrodos fraturados ou um dispositivo não cardíaco ativo.

A programação do dispositivo fora do modelo de programação condicional para RM também torna o dispositivo não condicional. O estado da bateria deve estar adequado para considerar o dispositivo condicional ( Tabela 55 ).

**Tabela 55:** – Recomendações para prevenção de interferências eletromagnéticas com relação à ressonância magnética

	Classe de recomendação	Nível de evidência
DCEI condicionais para RM devem ser assim considerados apenas quando a especificação do produto for respeitada, o que inclui a programação do “modo RM” apropriado e a realização do exame com os pré-requisitos especificados para o dispositivo A RM em um paciente com um sistema condicional deve sempre ser realizada no contexto de um fluxo de trabalho institucional padronizado, rigorosamente aplicado, seguindo as condições técnicas apropriadas Recomenda-se aos pacientes com sistema condicional de RM que ECG e monitoração por oximetria de pulso continuem até a o término da observação do paciente, ou até que outras configurações clinicamente apropriadas do dispositivo sejam restauradas	I	A
Recomenda-se para o paciente com DCEI não condicional que a avaliação do dispositivo seja realizada imediatamente antes e após a RM, com documentação de limiar de estimulação, amplitude das ondas P e R e impedância de cabo-eletrodo utilizando um protocolo padronizado Um desfibrilador/monitor (com função de estimulação externa) e um sistema de programação de DCEI específico do fabricante devem estar imediatamente disponíveis na área adjacente à sala de RM, enquanto o dispositivo não condicional estiver programado para aquisição das imagens. Recomendam-se que monitoramento contínuo do ECG e oximetria de pulso sejam utilizados enquanto o dispositivo não condicional da RM estiver programado para aquisição imagens. Recomenda-se pessoal com a habilidade para suporte avançado de vida para acompanhamento do paciente com DCEI não condicional para RM, até que seja avaliado e declarado estável para retornar a *status* não monitorado Para pacientes com um DCEI não condicional para RM dependente de estimulação (MP ou CDI), recomendam-se que: a) um médico com capacidade para implantar MP temporário esteja imediatamente disponível nas instalações; b) um médico com a capacidade de programar DCEI esteja imediatamente disponível nas instalações Recomenda-se que o paciente com um DCEI não condicional para RM e dependente de estimulação seja programado para um modo de estimulação assíncrona com desativação de recursos adaptativos (sensor de resposta à frequência) durante o exame. A frequência de estimulação deve ser apropriada para evitar estimulação competitiva Todas as detecções de taquicardia para pacientes com CDI devem ser desativadas antes da RM	I	B
Recomenda-se aos pacientes com sistema condicional para RM a assistência de pessoal com habilidade para realizar suporte avançado de vida. No caso de atendimento ao paciente, este deve ocorrer pelo período até o DCEI ser reprogramado, ou até ser avaliado e declarado estável para retornar ao *status* não monitorado O médico responsável pela RM deve ser informado da presença de paciente com um DCEI não condicional Recomenda-se que o monitoramento do ECG e da oximetria de pulso seja continuado até o final do exame ou até que as configurações do dispositivo sejam reprogramadas Todos os esforços de reanimação e tratamentos de emergência que envolvam o uso de um desfibrilador/monitor, sistema de programação de DCEI ou qualquer outro equipamento inseguro para RM devem ser realizados após a transferência do paciente para fora da zona 4	I	C
É razoável que pacientes com um sistema não condicional sejam submetidos à RM se não houver eletrodo fraturado, epicárdico ou abandonado, e a RM é o melhor teste para a condição. Deve existir um protocolo institucional e um médico responsável pela RM e pelo DCEI É razoável a realização de RM imediatamente após o implante de um eletrodo ou gerador de um dispositivo não condicional, se for clinicamente justificado Para um paciente com DCEI não condicional e que não depende da estimulação, é possível programar o dispositivo para qualquer modo sem estimulação (OVO/ODO) ou para um modo inibido (DDI/VVI), com desativação de recursos avançados ou adaptativos durante o exame de RM	IIa	B
É razoável a realização de RM em paciente com sistema condicional implantado mais recentemente do que o período isento de condicionalidade do sistema, com base na avaliação de risco e benefício para esse paciente Para os pacientes com DCEI não condicionals, é possível realizar RM repetidas quando necessário e sem restrição quanto ao intervalo mínimo entre os estudos de imagem ou o número máximo de estudos realizados É razoável programar pacientes com DCEI não condicional que não depende da estimulação para um modo de estimulação assíncrona (VOO/DOO) com desativação de recursos avançados ou adaptáveis durante o exame e com taxa de estimulação que evite a estimulação competitiva Para pacientes com DCEI não condicional, é razoável agendar uma avaliação completa do dispositivo dentro de 1 semana nos casos de aumento do limiar de estimulação > 1,0V, diminuição da amplitude da onda P ou da onda R > 50%, alteração da impedância do eletrodo > 50ohms e alteração da impedância do eletrodo de choque > 5ohms.	IIa	C

*CDI: cardioversor-desfibrilador implantável; DCEI: dispositivos cardíacos eletrônicos implantáveis; ECG: eletrocardiograma; MP: marca-passo; RM: ressonância magnética.*

### 8.3. Radioterapia

Número crescente de pacientes que se submetem à RT é portador de DCEI. Embora a ocorrência de disfunções induzidas por RT seja rara, recomendações de segurança são importantes.

A radiação ionizante pode causar interferência em componentes do CMOS (do inglês, *complementary metal-oxide-semiconductor* ) do gerador. A geração secundária de nêutrons é a maior preditora de disfunção dos DCEI no contexto da RT. Os geradores de pulsos mais modernos têm menor consumo de energia e circuitos menores, constituídos de metal semicondutor. Isso leva a maior suscetibilidade desses dispositivos a possíveis danos causados por radiação ionizante.^593 , 619^

Altas doses de radiação, especialmente com energia > 6MV, podem ocasionar erros de *software* e *hardware* . Esses distúrbios são geralmente transitórios, como inibição da estimulação, alterações na sensibilidade ou estimulação inapropriada na frequência máxima do sensor. A reversão para o modo de *backup* ( *reset* ), que pode ser corrigida com a reprogramação, é uma das disfunções mais relatadas. Defeitos permanentes também podem ocorrer, como a perda da telemetria e depleção prematura da bateria. A falência do dispositivo, com perda completa da função do DCEI, já foi descrita *in vitro* .^593 , 619 - 621^

É importante considerar, ainda, que os defeitos nos DCEI podem aparecer semanas ou meses após o término da RT (defeitos latentes).^622^Disfunções dos dispositivos são relatadas em até 3% dos cursos de RT. Eventos clinicamente relevantes são muito raros, dependem do tipo de dispositivo e da tolerância do paciente às alterações. Por exemplo, o paciente dependente de MP pode apresentar bradicardia e sintomas relacionados.^593 , 619^

O planejamento da RT deve considerar as especificações do DCEI, bem como as características dos pacientes (dependente ou não de estimulação, histórico de taquicardia ou fibrilação ventricular (TV/FV) ( Tabela 56 ).

**Tabela 56:** – Recomendações para prevenção de interferências eletromagnéticas relacionadas à radioterapia

	Classe de indicação	Nível de evidência
Antes de iniciar a RT, completa avaliação do DCEI deve ser efetuada e a equipe responsável pelo tratamento deve ser informada: Se o dispositivo é um MP ou CDI Se o paciente é dependente da estimulação Frequência mínima de estimulação programada Frequências máximas programadas ( *tracking* e de sensor) Tratamento com RT que não produz nêutrons é preferível àquela que produz nêutron, pelo menor risco de causar disfunções do DCEI, como *reset* do gerador Avaliação completa do DCEI deve ser realizada semanalmente, em paciente submetido à RT que produz nêutrons Avaliação completa do DCEI deve ser realizada ao término de um curso de tratamento radioterápico	I	B
São recomendadas a interrogação e a avaliação do DCEI a 1, 3 e 6 meses após o término da RT pelo risco de danos latentes	I	C
A relocação do DCEI pode ser indicada se o gerador estiver situado no caminho do feixe da radiação	IIa	C
O reposicionamento do DCEI não é recomendado quando a dose cumulativa recebida pelo dispositivo é <5Gy	III	B

*CDI: cardioversor-desfibrilador implantável; DCEI: dispositivos cardíacos eletrônicos implantáveis; MP: marca-passo; RT: radioterapia.*

## 9. Conclusão

Muitas evidências científicas surgiram desde a publicação das últimas diretrizes brasileiras de DCEI da SBC/SOBRAC. A evolução da tecnologia e do próprio conhecimento deve estar alinhada com a prática clínica e com a atenção à saúde pública. Dessa maneira, o presente documento destaca a evolução do tratamento das arritmias cardíacas, mas não se furta em destacar a necessidade premente do uso racional dos recursos financeiros em favor do bem maior, qual seja, a saúde coletiva.
