# Supplementary material for: Diretriz Brasileira de Dispositivos Cardíacos Eletrônicos Implantáveis – 2023
Source: Arq Bras Cardiol. 2023 Jan 16;120(1):e20220892. [Article in Portuguese] doi: 10.36660/abc.20220892 (PMC10389103; doi:10.36660/abc.20220892)
Supplement: Translation

# Translation: Brazilian Guidelines for Cardiac Implantable Electronic Devices – 2023

**Authors:** Ricardo Alkmim Teixeira, Alexsandro Alves Fagundes, José Mário Baggio, Júlio César de Oliveira, Paulo de Tarso Jorge Medeiros, Bruno Pereira Valdigem, Luiz Antônio Castilho Teno, Rodrigo Tavares Silva, Celso Salgado de Melo, Jorge Elias, Antonio Vitor Moraes, Anisio Alexandre Andrade Pedrosa, Fernando Mello Porto, Hélio Lima de Brito, Thiago Gonçalves Schroder e Souza, José Carlos Pachón Mateos, Luis Gustavo Belo de Moraes, Alexander Romeno Janner Dal Forno, Andre Luiz Buchele D’Avila, Diogo Alberto de Magalhães Cavaco, Ricardo Ryoshim Kuniyoshi, Mauricio Pimentel, Luiz Eduardo Montenegro Camanho, Eduardo Benchimol Saad, Leandro Ioschpe Zimerman, Eduardo Bartholomay Oliveira, Mauricio Ibrahim Scanavacca, Martino Martinelli, Carlos Eduardo Batista de Lima, Giselle de Lima Peixoto, Francisco Carlos da Costa Darrieux, Jussara de Oliveira Pinheiro Duarte, Silas dos Santos Galvão, Eduardo Rodrigues Bento Costa, Enrique Indalécio Pachón Mateo, Sissy Lara De Melo, Thiago da Rocha Rodrigues, Eduardo Arrais Rocha, Denise Tessariol Hachul, Adalberto Menezes Lorga, Silvana Angelina D’Orio Nishioka, Eduardo Barreto Gadelha, Roberto Costa, Veridiana Silva de Andrade, Gustavo Gomes Torres, Nestor Rodrigues de Oliveira, Fernando Antonio Lucchese, Henrique Murad, José Wanderley, Paulo Roberto Slud Brofman, Rui M. S. Almeida, João Carlos Ferreira Leal

**Affiliations:** 1 Brazil Hospital Renascentista, Pouso Alegre, MG – Brazil; 2 Brazil Hospital Ana Nery, Salvador, BA – Brazil; 3 Brazil Instituto de Cardiologia do Distrito Federal, Brasília, DF – Brazil; 4 Brazil Universidade Federal de Mato Grosso (UFMT),Cuiabá, MT – Brazil; 5 Brazil Instituto Dante Pazzanese de Cardiologia, São Paulo, SP – Brazil; 6 Brazil Clínica Cardiovascular Ribeirão Preto, Ribeirão Preto, SP – Brazil; 7 Brazil Universidade de Franca (UNIFRAN), Franca, SP – Brazil; 8 Brazil Centro Universitário Municipal de Franca (Uni-FACEF), Franca, SP – Brazil; 9 Brazil Clínica de Marca-passos Cardíacos, Uberaba, MG – Brazil; 10 Brazil Universidade Federal do Espírito Santo (UFES), Vitória, ES – Brazil; 11 Brazil Santa Casa de Ribeirão Preto, Ribeirão Preto, SP – Brazil; 12 Brazil Unimed de Ribeirão Preto, Ribeirão Preto, SP – Brazil; 13 Brazil Instituto do Coração (Incor) do Hospital das Clínicas da Faculdade de Medicina da Universidade de São Paulo (FMUSP), São Paulo, SP – Brazil; 14 Brazil Pontifícia Universidade Católica de Campinas, Campinas, SP – Brazil; 15 Brazil Universidade Federal de Juiz de Fora, Juiz de Fora, MG – Brazil; 16 Brazil Hospital Universitário da Universidade Federal de Juiz de Fora (UFJF), Juiz de Fora, MG – Brazil; 17 Brazil Hospital Universitário Clementino Fraga Filho, Universidade Federal do Rio de Janeiro (UFRJ), Rio de Janeiro, RJ – Brazil; 18 Brazil Hospital SOS Cárdio, Florianópolis, SC – Brazil; 19 Portugal Hospital de Santa Cruz, Lisboa – Portugal; 20 Brazil Centrocor Vitória, Vitória, ES – Brazil; 21 Brazil Vitória Apart Hospital, Vitória, ES – Brazil; 22 Brazil Hospital de Clínicas de Porto Alegre, Universidade Federal do Rio Grande do Sul (UFRGS), Porto Alegre, RS – Brazil; 23 Brazil Hospital Pró-Cardíaco, Rio de Janeiro, RJ – Brazil; 24 Brazil Hospital Samaritano, Rio de Janeiro, RJ – Brazil; 25 Brazil Universidade Federal do Rio Grande do Sul (UFRGS), Porto Alegre, RS – Brazil; 26 Brazil Hospital Mãe de Deus, Porto Alegre, RS – Brazil; 27 Brazil Hospital Universitário da Universidade Federal do Piauí (UFPI), Teresina, PI – Brazil; 28 Brazil Empresa Brasileira de Serviços Hospitalares (EBSERH), Brasília, DF – Brazil; 29 Brazil DentCor Clínica Médica e Odontológica, Santo André, SP – Brazil; 30 Brazil Hospital Universitário Professor Edgard Santos, Universidade Federal da Bahia (UFBA), Salvador, BA – Brazil; 31 Brazil Centro Avançado de Ritmologia e Eletrofisiologia (CARE), Vista, SP – Brazil; 32 Brazil CardioRitmo, Clínica de Arritmias Cardíacas, São José do Rio Preto, RP – Brazil; 33 Brazil Serviço de Eletrofisiologia, Marca-passos e Arritmias (SEMAP),São Paulo, SP – Brazil; 34 Brazil Hospital Felício Rocho, Belo Horizonte, MG – Brazil; 35 Brazil Hospital Universitário Walter Cantídio, Universidade Federal do Ceará (UFC), Fortaleza, CE – Brazil; 36 Brazil Instituto de Moléstias Cardiovasculares (IMC), São José do Rio Preto, SP – Brazil; 37 Brazil Hospital Dom Helder Camara, Recife, PE – Brazil; 38 Brazil Faculdade de Medicina da Universidade de São Paulo (FMUSP), São Paulo, SP – Brazil; 39 Brazil Universidade Federal de São Paulo (UNIFESP), São Paulo, SP – Brazil; 40 Brazil Hospital Universitário Onofre Lopes, Universidade Federal do Rio Grande do Norte (UFRN), Natal, RN – Brazil; 41 Brazil Irmandade Santa Casa de Misericórdia de Porto Alegre, Porto Alegre, RS – Brazil; 42 Brazil Universidade Federal do Rio de Janeiro (UFRJ), Rio de Janeiro, RJ – Brazil; 43 Brazil Universidade Federal de Alagoas (UFAL), Maceió, AL – Brazil; 44 Brazil Santa Casa Curitiba, Curitiba, PR – Brazil; 45 Brazil Centro Universitário Fundação Assis Gurgacz, Cascavel, PR – Brazil; 46 Faculdade de Medicina de São José do Rio Preto (FAMERP), São José do Rio Preto, SP – Brazil

**Table:** 

Brazilian Guidelines for Cardiac Implantable Electronic Devices – 2023	
The report below lists declarations of interest as reported to the SBC by the experts during the period of the development of these statement, 2019/2022.	
Expert	Type of relationship with industry
Adalberto Menezes Lorga Filho	Financial declaration A - Economically relevant payments of any kind made to (i) you, (ii) your spouse/partner or any other person living with you, (iii) any legal person in which any of these is either a direct or indirect controlling owner, business partner, shareholder or participant; any payments received for lectures, lessons, training instruction, compensation, fees paid for participation in advisory boards, investigative boards or other committees, etc. From the brazilian or international pharmaceutical, orthosis, prosthesis, equipment and implants industry: - Vatis / Libbs; Lixiana / Daiichi Sankyo; Eliquis / Pfizer: Atrial fibrillation. Other relationships Funding of continuing medical education activities, including travel, accommodation and registration in conferences and courses, from the brazilian or international pharmaceutical, orthosis, prosthesis, equipment and implants industry: - Daiichi Sankyo: Lixiana
Alexander Romeno Janner Dal Forno	Financial declaration A - Economically relevant payments of any kind made to (i) you, (ii) your spouse/partner or any other person living with you, (iii) any legal person in which any of these is either a direct or indirect controlling owner, business partner, shareholder or participant; any payments received for lectures, lessons, training instruction, compensation, fees paid for participation in advisory boards, investigative boards or other committees, etc. From the brazilian or international pharmaceutical, orthosis, prosthesis, equipment and implants industry: - Biotronik: Pacemaker, Abbott: Atrial fibrillation. B - Research funding under your direct/personal responsibility (directed to the department or institution) from the brazilian or international pharmaceutical, orthosis, prosthesis, equipment and implants industry: - Proadi.
Alexsandro Alves Fagundes	Financial declaration A - Economically relevant payments of any kind made to (i) you, (ii) your spouse/partner or any other person living with you, (iii) any legal person in which any of these is either a direct or indirect controlling owner, business partner, shareholder or participant; any payments received for lectures, lessons, training instruction, compensation, fees paid for participation in advisory boards, investigative boards or other committees, etc. From the brazilian or international pharmaceutical, orthosis, prosthesis, equipment and implants industry: - Bayer: Xarelto, Medtronic, Sankyo, Libbs. Other relationships Funding of continuing medical education activities, including travel, accommodation and registration in conferences and courses, from the brazilian or international pharmaceutical, orthosis, prosthesis, equipment and implants industry: - Boston.
Andre Luiz Buchele D’Avila	Nothing to be declared
Anisio Alexandre Andrade Pedrosa	Financial declaration A - Economically relevant payments of any kind made to (i) you, (ii) your spouse/partner or any other person living with you, (iii) any legal person in which any of these is either a direct or indirect controlling owner, business partner, shareholder or participant; any payments received for lectures, lessons, training instruction, compensation, fees paid for participation in advisory boards, investigative boards or other committees, etc. From the brazilian or international pharmaceutical, orthosis, prosthesis, equipment and implants industry: - Medtronic / Biomedical / Biocath / Imagem: Proctor. Other relationships Funding of continuing medical education activities, including travel, accommodation and registration in conferences and courses, from the brazilian or international pharmaceutical, orthosis, prosthesis, equipment and implants industry: - Medtronic, Biocath, Biomedical.
Antonio Vitor Moraes Júnior	Financial declaration A - Economically relevant payments of any kind made to (i) you, (ii) your spouse/partner or any other person living with you, (iii) any legal person in which any of these is either a direct or indirect controlling owner, business partner, shareholder or participant; any payments received for lectures, lessons, training instruction, compensation, fees paid for participation in advisory boards, investigative boards or other committees, etc. From the brazilian or international pharmaceutical, orthosis, prosthesis, equipment and implants industry: - Biotronik. Other relationships Funding of continuing medical education activities, including travel, accommodation and registration in conferences and courses, from the brazilian or international pharmaceutical, orthosis, prosthesis, equipment and implants industry: - Xarelto / Bayer: Atrial fibrillation.
Bruno Pereira Valdigem	Nothing to be declared
Carlos Eduardo Batista de Lima	Financial declaration A - Economically relevant payments of any kind made to (i) you, (ii) your spouse/partner or any other person living with you, (iii) any legal person in which any of these is either a direct or indirect controlling owner, business partner, shareholder or participant; any payments received for lectures, lessons, training instruction, compensation, fees paid for participation in advisory boards, investigative boards or other committees, etc. From the brazilian or international pharmaceutical, orthosis, prosthesis, equipment and implants industry: - Daiichi Sankyo / Lixiana: Atrial fibrillation. C - Personal research funding paid by the brazilian or international pharmaceutical, orthosis, prosthesis, equipment and implants industry: - RIVER AF trial, Thrombosis Research Institute / Brazilian Clinical Research.
Celso Salgado de Melo	Nothing to be declared
Denise Tessariol Hachul	Nothing to be declared
Diogo Alberto de Magalhães Cavaco	Financial declaration A - Economically relevant payments of any kind made to (i) you, (ii) your spouse/partner or any other person living with you, (iii) any legal person in which any of these is either a direct or indirect controlling owner, business partner, shareholder or participant; any payments received for lectures, lessons, training instruction, compensation, fees paid for participation in advisory boards, investigative boards or other committees, etc. From the brazilian or international pharmaceutical, orthosis, prosthesis, equipment and implants industry: - Boston Scientific/S-ICD.
Eduardo Arrais Rocha	Financial declaration A - Economically relevant payments of any kind made to (i) you, (ii) your spouse/partner or any other person living with you, (iii) any legal person in which any of these is either a direct or indirect controlling owner, business partner, shareholder or participant; any payments received for lectures, lessons, training instruction, compensation, fees paid for participation in advisory boards, investigative boards or other committees, etc. From the brazilian or international pharmaceutical, orthosis, prosthesis, equipment and implants industry: - ABBOTT: Remote monitoring class. Other relationships Funding of continuing medical education activities, including travel, accommodation and registration in conferences and courses, from the brazilian or international pharmaceutical, orthosis, prosthesis, equipment and implants industry: - Biotronik / Bayer: Congress. Any economically relevant equity interest in companies in the healthcare or education industry or in any companies competing with or supplying to SBC: - Health Area.
Eduardo Barreto Gadelha	Nothing to be declared
Eduardo Bartholomay Oliveira	Financial declaration A - Economically relevant payments of any kind made to (i) you, (ii) your spouse/partner or any other person living with you, (iii) any legal person in which any of these is either a direct or indirect controlling owner, business partner, shareholder or participant; any payments received for lectures, lessons, training instruction, compensation, fees paid for participation in advisory boards, investigative boards or other committees, etc. From the brazilian or international pharmaceutical, orthosis, prosthesis, equipment and implants industry: - Biotronik.
Eduardo Benchimol Saad	Financial declaration A - Economically relevant payments of any kind made to (i) you, (ii) your spouse/partner or any other person living with you, (iii) any legal person in which any of these is either a direct or indirect controlling owner, business partner, shareholder or participant; any payments received for lectures, lessons, training instruction, compensation, fees paid for participation in advisory boards, investigative boards or other committees, etc. From the brazilian or international pharmaceutical, orthosis, prosthesis, equipment and implants industry: - Biosense Webster: Catheter ablation.
Eduardo Rodrigues Bento Costa	Nothing to be declared
Enrique Indalécio Pachón Mateo	Nothing to be declared
Fernando Antônio Lucchese	Nothing to be declared
Fernando Mello Porto	Nothing to be declared
Francisco Carlos da Costa Darrieux	Financial declaration A - Economically relevant payments of any kind made to (i) you, (ii) your spouse/partner or any other person living with you, (iii) any legal person in which any of these is either a direct or indirect controlling owner, business partner, shareholder or participant; any payments received for lectures, lessons, training instruction, compensation, fees paid for participation in advisory boards, investigative boards or other committees, etc. From the brazilian or international pharmaceutical, orthosis, prosthesis, equipment and implants industry: - Bayer / Boehringer Ingelheim / Pfizer / Libbs / Biolab: Anticoagulation and atrial fibrillation. Other relationships Funding of continuing medical education activities, including travel, accommodation and registration in conferences and courses, from the brazilian or international pharmaceutical, orthosis, prosthesis, equipment and implants industry: - Bayer / Pfizer: Congresses and Virtual Courses.
Giselle de Lima Peixoto	Nothing to be declared
Gustavo Gomes Torres	Financial declaration A - Economically relevant payments of any kind made to (i) you, (ii) your spouse/partner or any other person living with you, (iii) any legal person in which any of these is either a direct or indirect controlling owner, business partner, shareholder or participant; any payments received for lectures, lessons, training instruction, compensation, fees paid for participation in advisory boards, investigative boards or other committees, etc. From the brazilian or international pharmaceutical, orthosis, prosthesis, equipment and implants industry: - Biotronik.
Hélio Lima de Brito Júnior	Other relationships Any economically relevant equity interest in companies in the healthcare or education industry or in any companies competing with or supplying to SBC: - Health Area.
Henrique Murad	Nothing to be declared
Jorge Elias Neto	Nothing to be declared
João Carlos Ferreira Leal	Financial declaration B - Research funding under your direct/personal responsibility (directed to the department or institution) from the brazilian or international pharmaceutical, orthosis, prosthesis, equipment and implants industry: - Braile Biomédica: Inovare® Alpha - Biological Valve Prosthesis / Artivion-Neomex: Evita open plus, hybrid stent.
José Carlos Pachón Mateos	Nothing to be declared
José Mário Baggio Junior	Financial declaration A - Economically relevant payments of any kind made to (i) you, (ii) your spouse/partner or any other person living with you, (iii) any legal person in which any of these is either a direct or indirect controlling owner, business partner, shareholder or participant; any payments received for lectures, lessons, training instruction, compensation, fees paid for participation in advisory boards, investigative boards or other committees, etc. From the brazilian or international pharmaceutical, orthosis, prosthesis, equipment and implants industry: - Abbott: Webinar Lecture; Biotronik: Satellite Symposium at Congress; Medtronic: Satellite Symposium at Congress. Other relationships Funding of continuing medical education activities, including travel, accommodation and registration in conferences and courses, from the brazilian or international pharmaceutical, orthosis, prosthesis, equipment and implants industry: - Medtronic: Training on leadless pacing and physiological stimulation; Biotronik: Congress; Abbott: Product training.
José Wanderley Neto	Nothing to be declared
Júlio César de Oliveira	Financial declaration A - Economically relevant payments of any kind made to (i) you, (ii) your spouse/partner or any other person living with you, (iii) any legal person in which any of these is either a direct or indirect controlling owner, business partner, shareholder or participant; any payments received for lectures, lessons, training instruction, compensation, fees paid for participation in advisory boards, investigative boards or other committees, etc. From the brazilian or international pharmaceutical, orthosis, prosthesis, equipment and implants industry: - Infinity.
Jussara de Oliveira Pinheiro Duarte	Nothing to be declared
Leandro Ioschpe Zimerman	Financial declaration A - Economically relevant payments of any kind made to (i) you, (ii) your spouse/partner or any other person living with you, (iii) any legal person in which any of these is either a direct or indirect controlling owner, business partner, shareholder or participant; any payments received for lectures, lessons, training instruction, compensation, fees paid for participation in advisory boards, investigative boards or other committees, etc. From the brazilian or international pharmaceutical, orthosis, prosthesis, equipment and implants industry: - Bayer/Xarelto; Daiichi Sankyo/Lixiana; Libbs/Propafenona e Amiodarona; Pfizer/Eliquis.
Luis Gustavo Belo de Moraes	Other relationships Any economically relevant equity interest in companies in the healthcare or education industry or in any companies competing with or supplying to SBC: - Sócio cotista da Cardioritmo Serviços Médicos Ltda e Luis Belo Serviços Médicos.
Luiz Antônio Castilho Teno	Nothing to be declared
Luiz Eduardo Montenegro Camanho	Nothing to be declared
Martino Martinelli Filho	Nothing to be declared
Mauricio Ibrahim Scanavacca	Financial declaration A - Economically relevant payments of any kind made to (i) you, (ii) your spouse/partner or any other person living with you, (iii) any legal person in which any of these is either a direct or indirect controlling owner, business partner, shareholder or participant; any payments received for lectures, lessons, training instruction, compensation, fees paid for participation in advisory boards, investigative boards or other committees, etc. From the brazilian or international pharmaceutical, orthosis, prosthesis, equipment and implants industry: - Johnson & Johnson. B - Research funding under your direct/personal responsibility (directed to the department or institution) from the brazilian or international pharmaceutical, orthosis, prosthesis, equipment and implants industry: - Milestone Pharmaceuticals: etripamil NS for the treatment of Paroxysmal. C - Personal research funding paid by the brazilian or international pharmaceutical, orthosis, prosthesis, equipment and implants industry: - Medtronic, J&J and Abbott.
Mauricio Pimentel	Financial declaration A - Economically relevant payments of any kind made to (i) you, (ii) your spouse/partner or any other person living with you, (iii) any legal person in which any of these is either a direct or indirect controlling owner, business partner, shareholder or participant; any payments received for lectures, lessons, training instruction, compensation, fees paid for participation in advisory boards, investigative boards or other committees, etc. From the brazilian or international pharmaceutical, orthosis, prosthesis, equipment and implants industry: - Bayer: Anticoagulation.
Nestor Rodrigues de Oliveira Neto	Nothing to be declared
Paulo Roberto Slud Brofman	Nothing to be declared
Paulo de Tarso Jorge Medeiros	Nothing to be declared
Ricardo Alkmim Teixeira	Financial declaration A - Economically relevant payments of any kind made to (i) you, (ii) your spouse/partner or any other person living with you, (iii) any legal person in which any of these is either a direct or indirect controlling owner, business partner, shareholder or participant; any payments received for lectures, lessons, training instruction, compensation, fees paid for participation in advisory boards, investigative boards or other committees, etc. From the brazilian or international pharmaceutical, orthosis, prosthesis, equipment and implants industry: - Boehringer-Ingelheim: Pradaxa, Jardiance; Daiichi-Sankyo: Lixiana; Abbott: Implantable Electronic Heart Devices; Biotronik: Implantable Electronic Heart Devices; Medtronic: Implantable Electronic Heart Devices; Biomedical: Lead Extraction from Implantable Electronic Cardiac Devices. Other relationships Funding of continuing medical education activities, including travel, accommodation and registration in conferences and courses, from the brazilian or international pharmaceutical, orthosis, prosthesis, equipment and implants industry: - Biomedical: Lead Extraction from Implantable Electronic Cardiac Devices.
Ricardo Ryoshim Kuniyoshi	Nothing to be declared
Roberto Costa	Financial declaration A - Economically relevant payments of any kind made to (i) you, (ii) your spouse/partner or any other person living with you, (iii) any legal person in which any of these is either a direct or indirect controlling owner, business partner, shareholder or participant; any payments received for lectures, lessons, training instruction, compensation, fees paid for participation in advisory boards, investigative boards or other committees, etc. From the brazilian or international pharmaceutical, orthosis, prosthesis, equipment and implants industry: - Boston Scientific: Speaker and consultant. Other relationships Participation in procurement committees for supplies or drugs in health institutions or any similar roles taken: - Reviewer of the auctions of the Hospital das Clínicas of FMUSP.
Rodrigo Tavares Silva	Nothing to be declared
Rui M. S. Almeida	Other relationships Any economically relevant equity interest in companies in the healthcare or education industry or in any companies competing with or supplying to SBC: - Health and Education Area.
Silas dos Santos Galvão Filho	Nothing to be declared
Silvana Angelina D’Orio Nishioka	Other relationships Funding of continuing medical education activities, including travel, accommodation and registration in conferences and courses, from the brazilian or international pharmaceutical, orthosis, prosthesis, equipment and implants industry: - Abbott. Performance, in the previous year, as a medical auditor for health insurance companies or the like: - For medical classes and training only.
Sissy Lara de Melo	Nothing to be declared
Thiago da Rocha Rodrigues	Financial declaration A - Economically relevant payments of any kind made to (i) you, (ii) your spouse/partner or any other person living with you, (iii) any legal person in which any of these is either a direct or indirect controlling owner, business partner, shareholder or participant; any payments received for lectures, lessons, training instruction, compensation, fees paid for participation in advisory boards, investigative boards or other committees, etc. From the brazilian or international pharmaceutical, orthosis, prosthesis, equipment and implants industry: - Bayer / Pfizer: eliquis; Daichi-Sanchio: lixiana; Abbott: rivacrist; Bhoeringer-Ingelheim: jardiance; Libbs: Vatis.
Thiago Gonçalves Schroder e Souza	Nothing to be declared
Veridiana Silva de Andrade	Nothing to be declared

## Contents

1. General Recommendations 8

1.1 Operating Room 8

**1.1.1. Human Resources** 8

**1.1.2. Material Resources** 9

**1.1.2.1. Fluoroscopy** 9

**1.1.2.2. Monitoring** 9

**1.1.2.3. Surgical Instruments** 9

1.2. CIED Evaluation and Programming Clinic 9

1.3. Clinical Evaluation Prior to CIED Implantation 9

1.4. Surgical Procedure and Types of CIED 9

2. Recommendations for Conventional Pacemaker Implantation 10

2.1. Sinus Node Disease 10

2.2. Atrioventricular and Intraventricular Blocks 12

**2.2.1. Atrioventricular Blocks** 12

**2.2.2. Intraventricular Blocks with 1:1 Atrioventricular Conduction** 14

2.3. Carotid Sinus Syndrome 14

2.4. Vasovagal Syndrome 16

2.5. Hypertrophic Cardiomyopathy 16

2.6. Neuromuscular Diseases 17

2.7 Obstructive Sleep Apnea Syndrome 18

2.8. Congenital Long QT Syndrome 18

2.9. Cardiac Transplantation 19

2.10. Choosing the Type of Pacemaker and Pacing Mode 20

2.11. Direct Stimulation of the Cardiac Conduction System (His Bundle, Left Bundle Branch) 20

2.12. Leadless Pacing 21

3. Recommendations for Multisite Pacemaker Implantation/Cardiac Resynchronization Therapy 22

3.1. Patients in Sinus Rhythm 22

3.2. Patients with Atrial Fibrillation 24

3.3. Conventional PM Upgrade 25

3.4. Indication for Antibradycardia Pacing (First Implant) 26

3.5. Indication for Implantable Cardioverter-defibrillator Combined with Cardiac Resynchronization Therapy 26

3.6. Direct Stimulation of the Cardiac Conduction System 27

**3.6.1. Cardiac Contractility Modulation** 28

4. Recommendations for Placement of Implantable Cardioverter-defibrillators 28

4.1. Primary Prevention of Sudden Death 28

**4.1.1. Ischemic Cardiomyopathy** 28

**4.1.2. Nonischemic Cardiomyopathy** 29

**4.1.3. Hypertrophic Cardiomyopathy** 30

**4.1.4. Chagas Cardiomyopathy** 32

**4.1.5. Arrhythmogenic Right Ventricular Cardiomyopathy** 33

**4.1.6. Noncompaction Cardiomyopathy** 34

**4.1.7. Congenital Long and Short QT Syndromes** 37

**4.1.8. Brugada Syndrome** 37

**4.1.9. Catecholaminergic polymorphic ventricular tachycardia** 38

**4.1.10. Idiopathic Ventricular Tachycardia** 38

4.2. Secondary prevention of sudden death 38

**4.2.1. Recovered cardiac arrest or sustained ventricular tachycardia** 38

**4.2.1.1. Recovered cardiac arrest or sustained ventricular tachycardia in the presence of structural heart disease** 38

**4.2.1.2. Survivors of Cardiac Arrest or Sustained Ventricular Tachycardia in the Absence of Structural Heart Disease** 40

**4.2.2. Syncope and Ventricular Tachycardia/Fibrillation on Electrophysiology Study** 40

4.3. Children, Adolescents, and Congenital Heart Disease 41

4.4. Choosing Implantable Cardioverter-defibrillator Type and Pacing Mode 44

**4.4.1. Implantation Technique** 44

**4.4.2. Pacing Mode** 44

4.5. Cost-effectiveness of Implantable Cardioverter-defibrillators in Primary and Secondary Prevention of Sudden Death 44

**4.5.1. Primary Prevention** 44

**4.5.2. Secondary Preventio** n 45

5. Recommendations for Implantable Loop Recorders 45

6. Recommendations for CIED Evaluation and Programming 46

6.1. Conventional Pacemakers 46

**6.1.1. Sinus Node Disease** 46

**6.1.2. Atrioventricular Block** 47

**6.1.3. Atrial Fibrillation** 47

**6.1.4. Neurally Mediated Syncope and Carotid Sinus Syndrome** 47

**6.2. Cardiac Resynchronization Therapy** 47

**6.3. Implantable Cardioverter-defibrillator** 49

**6.4. Implantable Loop Recorder** 50

**6.5. Remote Monitoring (Online)** 50

7. Recommendations for Prevention and Treatment of CIED infecTions and for System Removal 51

7.1. Prevention and Treatment of Infections 51

7.2. Lead Removal from Cardiac Implantable Electronic Devices 55

8. Recommendations for the prevention of electromagnetic interference 58

8.1. Surgery Using Electrocautery 59

8.2. Magnetic Resonance Imaging 60

8.3. Radiotherapy 62

9. Conclusion 62

References 64

## 1. General Recommendations

Although implantation techniques for cardiac implantable electronic devices (CIEDs) have been standardized and simplified, adequate settings and materials, as well as medical knowledge and surgical experience, remain necessary. Electrocardiographic (ECG) knowledge, especially of cardiac arrhythmias and the principles of cardiac electrophysiology, is essential.

### 1.1. Operating Room

Surgical procedures involving artificial cardiac pacing are performed by cardiovascular surgeons or cardiologists with cardiac pacing training (SOBRAC/SBC, ABEC/SBCCV, AMB). The procedures should be performed in an operating room or a catheterization/electrophysiology laboratory. The operating room should have adequate size, lighting, and ventilation, a sink for surgical hand antisepsis, and a dual-voltage electrical system (with a grounding system that prevents electromagnetic interference and protects the materials).

#### 1.1.1. Human Resources

Professionals involved in surgical procedures for CIED implantation include:

- Attending and assisting physicians with cardiac pacing training
- Anesthesiologist
- Scrub nurse preferably with cardiac pacing training
- Nursing professional preferably with cardiac pacing training
- Pacemaker (PM) technician
- Radiology technician

#### 1.1.2. Material Resources

##### 1.1.2.1. Fluoroscopy

A fluoroscopy system (with an image intensifier) remains essential during CIED procedures. The equipment may be fixed, as in catheterization laboratories, or portable (C-arm). Image quality and image recording and mirroring resources facilitate the procedure, especially for cardiac resynchronization therapy (CRT). The image intensifier should allow visualization of small-caliber guidewires and movements from different views (including oblique views).

##### 1.1.2.2. Monitoring

Continuous ECG monitoring should be performed, and ECG tracings may be stored. Available leads should allow proper intraoperative assessment of CRT and physiological pacing of the conduction system (His bundle, left bundle branch [LBB], deep septal). In these cases, an intracavitary ECG (polygraph) should be analyzed.

Noninvasive monitoring of blood pressure and pulse oximetry should be available.

##### 1.1.2.3. Surgical Instruments

- A tray with the adequate surgical instruments
- Electrocautery device
- External cardioverter-defibrillator
- Temporary external PM
- Advanced life support system
- Materials and drugs for anesthesia and cardiovascular stability (analgesics, anesthetics, antiarrhythmics, vasoactive drugs, antibiotics etc.)
- Pulse generator, leads, introducers, and sheaths for catheterization of the coronary sinus and conduction system
- CIED-specific programmer and analyzer being used or to be implanted
- Ultrasound for venous access may reduce complications related to deep vein puncture (eg, accidental arterial puncture, pneumothorax)
- Transesophageal echocardiography: helpful during percutaneous lead extraction for early diagnosis of cardiac tamponade

## 1.2. CIED Evaluation and Programming Clinic

The physician in charge of a CIED follow-up clinic should have cardiac pacing training (SOBRAC/SBC, ABEC/SBCCV, AMB). The clinic’s structure should include:

- ECG machine
- CIED programmer devices from different manufacturers
- Uninterruptible power supply
- External cardioverter-defibrillator with transcutaneous PM
- Magnet
- Transthoracic echocardiogram
- Access to additional tests such as cardiac stress test, 24-hour Holter monitoring, and imaging tests (radiography, magnetic resonance imaging [MRI], myocardial scintigraphy, computed tomography). The tilt table test must be available at the clinic or at a referral facility.
- Access to an engineer specializing in cardiac pacing

## 1.3. Clinical Evaluation Prior to CIED Implantation

a) Initial clinical evaluation prior to CIED implantation should include:

Patient history and physical examination

Patient history should investigate signs and symptoms of cardiac arrhythmias, such as syncope, presyncope, dizziness, and palpitations, and signs and symptoms of heart failure (HF). A family history of sudden death is highly relevant, especially if occurring prematurely or affecting first-degree relatives.

Physical examination should include inspection, peripheral pulse palpation, blood pressure measurement, cardiac and carotid auscultation, heart rate, and peripheral perfusion.

If possible, oral anticoagulants and antiplatelets should be temporarily interrupted before the surgical procedure.^1^Preventive interruption of other drugs is generally unnecessary. Patients with signs of active infection must not undergo device implantation until the infection is resolved.

b) Preoperative additional tests

- Resting ECG;
- Chest radiograph (posteroanterior view plus left profile);
- Laboratory tests: complete blood count and coagulation profile tests are required for all patients. For procedures that require intravenous contrast (eg, CRT and venous obstruction), a renal function evaluation with electrolyte measurement should be performed. In patients with diabetes, fasting glucose should be assessed. Urinalysis and urine culture tests are indicated for patients with urinary complaints;
- Tests such as echocardiogram, 24-hour Holter monitoring, electrophysiology study (EPS), and upper-limb ultrasound or venography should be performed only if the clinical condition requires.

Patients should fast for at least 6 to 8 hours before surgery depending on the complexity of the procedure and type of anesthesia. Trichotomy, local antisepsis, and prophylactic antibiotic therapy^2^should follow institutional protocols.

## 1.4. Surgical Procedure and Types of CIED

**a) Surgical procedures**

Before surgery, medical and nursing staff must follow safe surgery protocols by checking patient’s name, date of birth, hospital record number, and laterality. The staff should also confirm the indication for the procedure and check preoperative test results.

CIED procedures should be performed in an operating room or a catheterization/electrophysiology laboratory under fluoroscopic guidance with continuous ECG monitoring, pulse oximetry, and intermittent or continuous blood pressure measurement. Devices such as subcutaneous implantable cardioverter-defibrillators (ICDs) and implantable loop recorders do not require the use of an image intensifier during implantation.

Anesthesia is either local, preferably combined with sedation, or general. The type of anesthesia depends on the complexity of the procedure, access route, and patient’s clinical status.

The choice of surgical access should consider the following: pulse generator implantation site, heart access for lead implantation (intravenous or epicardial route), and the possibility of implanting devices without transvenous leads (leadless PM and subcutaneous ICD). The use of a temporary PM and venous catheters, previous thoracic surgeries, need for radiotherapy, anatomical characteristics, skin infections, and dominant arm may also influence the choice of surgical strategy.

The pulse generator pocket is generally created in the pectoral region, although it may be abdominal in specific situations, and the device is placed in a subcutaneous or submuscular position. Venous access is achieved by cephalic vein dissection or puncture of the axillary, subclavian, jugular, or femoral vein. The number of leads varies according to the type of implanted device, usually ranging from one to three. Active-fixation (screw-in) leads are currently preferred to passive-fixation leads according to professional experience.

During the surgical procedure, pacing and sensing thresholds and lead impedance parameters should be obtained, and an endocavitary or epicardial electrogram should be performed. For patients undergoing ICD implantation, shock impedance should be measured; defibrillation threshold testing is optional for patients undergoing transvenous ICD implantation (usually unnecessary) but recommended for patients undergoing right pectoral subcutaneous ICD implantation.^3 - 5^Multisite pacing systems with lead implantation for left ventricular (LV) pacing through the carotid sinus require specific tools, such as sheaths, electrophysiology catheter, and venography catheter for choosing the best tributary vein for lead implantation.

The CIED implantation report should include patient identification, surgery description, technical data regarding the system, and if there were any complications (eg, pneumothorax, hemothorax, failure to capture and/or sense, bad lead-generator connection, lead dislodgement, right ventricle (RV) perforation, diaphragmatic stimulation, pocket hematoma, contamination, and arrhythmias). The Brazilian Pacemaker Registry must be completed.

*Postoperative and inpatient evaluation*

After CIED implantation, the patient should be clinically evaluated, and the implanted device should undergo electronic assessment. ECG and chest radiography should be performed to confirm proper device function and lead position and to identify any dysfunctions or complications.

Patients are generally hospitalized for 12 to 24 hours. Those undergoing procedures that do not require intravascular access (pulse generator replacement or subcutaneous device implantation) usually remain under postoperative observation for 6 to 12 hours (day hospital).

**b) Types of CIED**

The main types of CIED and their characteristics are summarized in Chart 1.

**Chart 1:** – Main types of CIED and their characteristics

Device	Characteristics
Pacemaker (PM)	✓ Multiprogrammable electronic cardiac device capable of monitoring and promoting electrical cardiac stimulation, restoring atrioventricular synchrony and heart rate variability, detecting and recording arrhythmias, and treating atrial arrhythmias using mechanisms of atrial overpacing ✓ PMs usually consist of a pulse generator, an electronic circuit, and leads that connect the generator to the endocardial surface. The devices may be single- or dual-chamber ✓ Recently introduced in the market, leadless PMs are electrical stimulation systems in which all device units are contained in a single intracardiac component implanted via a transvenous sheath system ✓ Bradyarrhythmias are the focus of PM therapy
Cardiac resynchronization therapy (CRT)	✓ Cardiac device with antibradycardia pacing function similar to a PM but allowing multisite cardiac pacing, ie, left and right ventricular (biventricular) pacing, for intraventricular dyssynchrony correction ✓ Adjuvant treatment of advanced heart failure is the primary focus of CRT
Implantable cardioverter-defibrillator (ICD)	✓ Electronic cardiac device with the same functions of a conventional PM plus the ability to detect, record, and automatically treat potentially fatal ventricular tachyarrhythmias via shock therapy (cardioversion or defibrillation) or programmed ventricular pacing (overdrive or antitachycardia pacing) ✓ Potentially fatal ventricular tachyarrhythmias associated or not with bradyarrhythmias may be treated with ICD
CRT + ICD	✓ Devices that combine CRT and ICD functions in a single device
Implantable loop recorder	✓ Subcutaneous cardiac device for prolonged cardiac monitoring (3 to 4 years) with the aim of detecting intermittent and sporadic cardiac arrhythmias in cases of unexplained syncope or cryptogenic stroke via continuous recording of the heart’s electrical activity

## 2. Recommendations for Conventional Pacemaker Implantation

### 2.1. Sinus Node Disease

Symptomatic sinus node dysfunctions are named sinus node disease (SND) and tend to be the most common indication for artificial cardiac pacing worldwide, accounting for approximately half of permanent PM implants.^6^

From an ECG perspective, SND is characterized by the presence of one or more of the following manifestations: sinus bradycardia, sinus pause or arrest, sinoatrial block, and atrial tachyarrhythmias (especially flutter or atrial fibrillation [AF]) associated with bradyarrhythmias (sinus pauses), such as tachycardia-bradycardia syndrome and chronotropic incompetence (inadequate heart rate response to exercise or stress).^7 - 9^

SND symptoms are related to low heart rate or to the duration of sinus pause. The most common symptoms include palpitation, tiredness, dyspnea, dizziness, and presyncope or syncope. Syncope is a common clinical symptom and may affect approximately 50% of patients referred to PM implantation due to SND.^10^

Although SND may occur at any time in life, the incidence increases with age, affecting 1 in 600 patients older than 65 years. Men and women are affected equally.^11 - 13^

SND pathophysiology is diverse and usually involves complex electrophysiological and structural remodeling.^14 , 15^Etiology can be divided into intrinsic and extrinsic factors.

Intrinsic causes of SND include inflammatory, infectious, and immunological processes, degenerative fibrosis, ion channel dysfunction, and sinoatrial node remodeling. Age-related idiopathic degenerative fibrosis is the most common intrinsic cause of SND. However, recent studies have demonstrated that an inherited ion channel dysfunction may also play a role in the genesis of age-related sinus dysfunction.^16 - 19^In addition, baroreflex response and heart rate variability are reduced in older patients.^20 , 21^

Other intrinsic mechanisms of SND include infiltrative (eg, hemochromatosis, amyloidosis) and inflammatory (eg, sarcoidosis) diseases, HF and AF (anatomical and structural changes along the crista terminalis), and chronic coronary heart disease (sinoatrial nodal artery involvement). Genomic analyses have identified loci in proteins that interact with ion channels and channels related to normal and abnormal resting heart rates, providing insights into the mechanisms that control heart rate.^22 - 24^

The main extrinsic causes of SND include pharmacological agents, metabolic disorders, and autonomic dysfunction. Betablockers, calcium channel blockers, digitalis, antiarrhythmics, and sympatholytic drugs are the pharmacological agents most commonly associated with the development of SND.^24^Lithium may also be associated with SND, often permanent.^25^

Autonomic dysfunction with cardioinhibitory manifestations can mimic or intensify SND in vasovagal syncope and carotid sinus hypersensitivity.^26^

Another rare cause of recurrent bradycardia and syncope is cardiac asystole induced by temporal lobe epilepsy. Although temporary PM implantation may be fundamental in the acute phase of this condition, specific management (surgical or pharmacological) of epilepsy may often regulate sinus dysfunction and/or atrioventricular (AV) block with no need for permanent PM implantation. The same considerations are applicable to another rare condition, namely glossopharyngeal neuralgia associated with cardiac asystole and syncope.^27 , 28^

Metabolic abnormalities, such as severe systemic acidosis, hyperkalemia, hypokalemia, and hypocalcemia, may cause sinus bradycardia, which is uncommon in acute cases. Other possible extrinsic factors include hypothyroidism, hypoxia, hypothermia, and toxins. The association between Brugada syndrome and the occurrence of SND has also been described.^29^

Patients with SND are usually asymptomatic in the initial phases of the disease and develop symptoms after several years. It is also important to identify cases of asymptomatic functional bradycardia, such as nocturnal bradycardia, in healthy young people and athletes, although this condition poses no health risks and requires no intervention.

Documenting a correlation between ECG changes and clinical manifestations is essential to SND diagnosis. Twelve-lead ECG or other methods, such as Holter monitoring (24-48 hours, 7 days) and external or implantable loop recorders, may be used.^30^Invasive EPS should not be regularly used in clinical practice because there are no conclusive data on the true indication for permanent PM implantation in patients with abnormal sinus node recovery time or sinoatrial conduction time.

Before choosing the best approach and deciding if PM implantation should be performed in patients with SND, establishing a correlation between bradycardia and clinical symptoms is crucial as well as identifying reversible causes.

In symptomatic SND without a reversible cause, permanent PM implantation is the treatment of choice, although there is no evidence that artificial cardiac pacing has any impact on survival or risk of sudden death in these patients compared with the general population.^30^However, PM implantation significantly increases quality of life, possibly reduces the risk of AF and systemic thromboembolism, allows the use of antiarrhythmics that may cause bradycardia, and provides continuous monitoring of heart rhythm.^31^The recommendations for permanent PM implantation in SND are described in Table 1 .

**Table 1:** – Recommendations for permanent pacemaker implantation in sinus node disease

	Class of recommendation	Level of evidence
Spontaneous SND (sinus bradycardia, sinus pause/arrest, sinoatrial block, or tachycardia-bradycardia syndrome) without a treatable cause or induced by necessary and irreplaceable drugs with documented symptoms of bradycardia (syncope, presyncope, dizziness, or tiredness/fatigue)	I	C
Spontaneous SND (sinus bradycardia, sinus pause/arrest, sinoatrial block, or tachycardia-bradycardia syndrome) without a treatable cause or induced by necessary and irreplaceable drugs without documented symptoms (syncope, presyncope, dizziness, or tiredness/fatigue) after a recommended investigation (presumptive diagnosis) Unexplained syncope with evidence of SND on EPS	IIa	C
Unexplained syncope with documented asymptomatic sinus pause > 6s	IIb	C
Asymptomatic patient with documented symptoms that are clearly unrelated to bradycardia; symptomatic patient with bradycardia due to reversible causes, including nonessential drugs	III	C

*EPS: electrophysiology study; SND: sinus node disease.*

Regarding pacing mode, major randomized studies have reported no improved survival with atrial (AAI) or AV (DDD) pacing compared with ventricular (VVI) pacing; however, benefits such as reductions in AF, syncope, and PM syndrome rates have been observed.^32 - 35^A systematic review of these major randomized studies identified a significant reduction in stroke (hazard ratio [HR]: 0.81) and AF (HR: 0.80) rates with AAI and/or DDD pacing compared with VVI pacing.^36^The effect of pacing mode on HF and stroke prevention and quality-of-life improvement is less evident. The Danish Multicenter Randomized Trial on Single Lead Atrial Pacing versus Dual Chamber Pacing in Sick Sinus Syndrome (DANPACE)^10^analyzed AAI vs DDD pacing in patients with SND and showed that AAIR pacing was associated with a higher incidence of paroxysmal AF (HR: 1.24) and a two-fold increase in reoperations compared with DDDR pacing. Reoperations were mostly due to the need for an upgrade from AAIR to DDDR pacing in patients who developed AV block during follow-up. Another relevant aspect in that study is that the benefit of AAI pacing may be attenuated in patients with long PR intervals that may trigger diastolic mitral regurgitation.

Atrial pacing (AAIR or DDDR) is the preferred pacing mode for patients with SND ( Table 2 ).

**Table 2:** – Recommendations for choosing a pacing mode in sinus node disease

	Class of recommendation	Level of evidence
1. AAI(R) in patients with normal AV conduction 2. DDD(R) in patients with advanced AV block	I	A
1. AAI(R) with automatic switch to DDD(R) in patients with intermittent advanced AV block	I	B
1. DDD with an algorithm that reduces ventricular pacing in patients with preserved AV conduction	IIa	B
1. VVI(R) in older people without retrograde VA conduction	IIb	B
1. VVI(R) in patients who may need only short periods of ventricular pacing or patients with significant comorbidities that affect survival or clinical events	IIb	C
1. VVI(R) in patients with retrograde VA conduction; VDD(R) 2. AAI(R) in patients with advanced AV block	III	C

*AV: atrioventricular; VA: ventriculoatrial.*

Most patients with SND present with preserved AV conduction. Conversely, RV pacing has been known to be associated with negative physiological consequences resulting from ventricular dyssynchrony (VD), such as LV remodeling, reduced LV ejection fraction (LVEF), and functional mitral regurgitation.^37^Therefore, algorithms that reduce unnecessary ventricular pacing, such as AV hysteresis and automatic switch from DDD to AAI mode, should be programmed in patients without associated AV block.^38^

Algorithms that suppress AF occurrence, such as continuous atrial pacing (atrial overpacing) or atrial pacing induced by intrinsic atrial activity sensing, either alone or combined, have no proven benefits. Similarly, alternative pacing sites – such as Bachmann bundle pacing and dual-site or multisite atrial pacing – have failed to show consistent effects.^39^

Current devices have one or more mechanisms of frequency response sensors usually based on body movement (piezoelectric crystals or accelerometers) or ventilation/minute volume. The main purpose of the sensors is to physiologically increase heart rate and not necessarily change clinical outcomes. Although devices cannot accurately assess atrial chronotropic response, they can provide indicators of atrial disease progression through rate and arrhythmia histograms, atrial pacing percentage, and patient daily activity. Such data may be useful for sensor programming. There is no evidence that the use of combined sensors (eg, accelerometer and minute ventilation) improves quality of life.

### 2.2. Atrioventricular and Intraventricular Blocks

#### 2.2.1. Atrioventricular Blocks

An electrical stimulus originating in the sinus node is propagated through the myocardium by the conduction system. A delay or failure in propagating the stimulus between atria and ventricles characterizes an AV block. This change in stimulus propagation may be a pathological change or a functional phenomenon resulting from physiological refractoriness (an intrinsic characteristic of conduction system cells).^40^

From an ECG perspective, AV blocks are classified into first-degree, second-degree (Mobitz I, Mobitz II, 2:1, advanced), and third-degree.

First-degree AV block is defined by a delay in stimulus conduction from the atrium to the ventricle with a PR interval > 200ms.

In type I second-degree AV block (Mobitz I), the block occurs after progressive prolongation of the PR interval (Wenckebach phenomenon), with a blocked P wave at the end. In type II second-degree AV block (Mobitz II), the P wave is suddenly blocked, ie, there is no progressive prolongation of the PR interval. When AV conduction occurs with a 2:1 ratio, second-degree block usually cannot be classified unequivocally as type I or type II without the support of autonomic maneuvers, drugs, or even invasive EPS. Advanced AV block refers to the blocking of two or more consecutive P waves with some conducted beats, which indicates some preservation of AV conduction. In AF with prolonged pauses (> 5s), advanced second-degree AV block should be considered.

Finally, third-degree AV block (complete AV block) is defined as the absence of AV conduction (complete dissociation between P waves and QRS complexes with atrial rate > ventricular rate).^41^

There are numerous congenital and, most often, acquired conditions that may affect AV conduction. Degenerative causes are commonly observed in clinical practice and are associated with aging, hypertension, and diabetes mellitus. The most common infectious causes in Brazil are chronic Chagas myocarditis and, to a lesser extent, viral acute myocarditis, which may cause definitive intermittent acute blocks.

AV blocks resulting from inferior-wall ischemia or acute myocardial infarction as well as autonomic nervous system-mediated blocks may be reversible.

Iatrogenic causes, especially from pharmacological action, should also be considered depending on clinical status.

Anatomically, AV blocks are defined as AV nodal, intra-Hisian, or infra-Hisian according to block site. AV nodal block is associated with slower progression, a faster and more reliable junctional escape, and improved response to autonomic manipulation with the administration of atropine, isoproterenol, and epinephrine. In contrast, intra-Hisian or infra-Hisian AV blocks progress more rapidly and are associated with a slower and more unpredictable ventricular escape, a wider QRS with poor response to adrenergic activity, and vagal block. High-degree blocks (advanced or third-degree) have a higher risk of low output and severe asystole and require urgent therapy. In the setting of AF, complete AV block is characterized by a low ventricular response (< 50bpm) and a regular RR interval.

First-degree AV block is generally asymptomatic but may result in fatigue or exertion intolerance if the PR interval is long enough to allow loss of AV synchrony. This change is called pseudo-PM syndrome and may occur when the PR interval is > 300ms.^42^Likewise, type I second-degree AV block is often asymptomatic and affects healthy, active patients with or without a history of heart disease, particularly during parasympathetic activity. However, if it occurs frequently or during exercise, it may cause exertion intolerance or dizziness.^43^

In 61% of patients with syncope and underlying bundle branch block or bifascicular block, significant and clinically relevant conduction abnormalities in the His-Purkinje system may be detected on EPS.^44^

In patients with AV block, clinical evaluation may help identify transient or reversible causes, and treatment or resolution may make permanent artificial pacing unnecessary.

In congenital complete AV block, permanent PM implantation must be indicated when symptoms are present or when the child has a resting heart rate < 55bpm or < 70bpm, if associated with structural heart disease.^45^In asymptomatic patients, regular follow-up with additional tests that assess mean heart rate, QT interval, pauses, ventricular arrhythmia, intraventricular (IV) conduction disorders (IVCD), presence or emergence of structural heart disease, low cognitive development and low weight and height gain, and exercise intolerance should be conducted to evaluate the need for permanent PM implantation.^46^Prophylactic PM or ICD implantation is also recommended in some asymptomatic patients with neuromuscular dysfunctions or other genetic disorders.

In patients with bradycardia indicative of PM implantation and LV dysfunction, ICD implantation should be considered (see item 4).

PM implantation is not indicated for asymptomatic patients with permanent AF and low resting heart rate who have an appropriate chronotropic response while awake, regardless of pause occurrence and duration. Conversely, for symptomatic patients with prolonged pauses (> 3s) that may have resulted from an infranodal block, PM implantation is indicated.^30^

Prophylactic PM implantation may be necessary especially in patients with HF or coronary artery disease (CAD) requiring chronic use of betablockers.^47^

To determine the best type of device and pacing mode for patients with AV block, the following clinical variables should be considered: expected ventricular pacing percentage and LV systolic function (ie, LVEF).

Studies comparing pacing with preservation of sequential AV activation vs single-chamber ventricular pacing in patients with AV block (eg, Pacemaker Selection in the Elderly [PASE], Mode Selection Trial [MOST], The Canadian Trial of Physiological Pacing [CTOPP]) showed no significant reduction in mortality or stroke rates.^34 , 35 , 48^

Conversely, a systematic review revealed that dual-chamber pacing is the preferred choice for reducing AF incidence and PM syndrome prevalence compared with single-chamber ventricular pacing (VVI).^49^However, the United Kingdom Pacing and Cardiovascular Events (UKPACE) trial (patients aged ≥75 years) found no benefits of AV pacing on mortality or AF and HF incidence compared with ventricular pacing. Similar stroke rates and higher rates of complications related to the surgical procedure were also observed in patients undergoing dual-chamber PM implantation (7.8% vs 3.5%; p < 0.001).^50^Therefore, the indication for single-chamber PM (VVI) is reasonable in frail patients or those with significant comorbidities, older age, a very sedentary lifestyle, or reduced daily need for pacing.

In patients with retrograde VA conduction, ventricular pacing may trigger symptoms of PM syndrome. In these cases, dual-chamber pacing should be preferred to avoid AV dyssynchrony.^51^

The deleterious effects of chronic RV pacing have been demonstrated in several studies, although only a minority (5% to 9%) of patients with chronic RV pacing develop severe ventricular dysfunction with symptoms of HF.^52 - 54^Patients with LV dysfunction and indication for PM due to AV block were evaluated in the Conventional versus Multisite Pacing for Bradyarrhythmia Therapy (COMBAT)^55^(LVEF < 35%) and Biventricular versus Right Ventricular Pacing in Heart Failure Patients with Atrioventricular Block (BLOCK-HF)^56^(LVEF ≤ 50%) studies. The studies compared CRT vs conventional RV pacing and demonstrated clinical improvement (New York Heart Association [NYHA] functional class) and reverse LV remodeling (increased LVEF) with CRT, with a significant reduction in primary outcomes.

In patients with AF and LV dysfunction undergoing AV node ablation for heart rate control, CRT or conduction system (His bundle or LBB) pacing seems to be associated with better outcomes compared with conventional RV pacing.^30^

The recommendations for PM implantation in AV block are summarized in Table 3 .

**Table 3:** – Recommendations for permanent pacemaker implantation in atrioventricular block

	Class of recommendation	Level of evidence
Acquired second-degree (Mobitz II), advanced, or third-degree AV block not associated with a reversible or physiological cause, regardless of presence of symptoms Second-degree (Mobitz II), advanced, or third-degree AV block or alternating bundle branch block (even if asymptomatic) persisting for at least 72 hours after AMI Advanced AV block or alternating bundle branch block after TAVI persisting for at least 24-48 hours Advanced AV block after AMI persisting for at least 5 days Symptomatic advanced AV block persisting for at least 5 days after heart valve surgery, revascularization, or AF surgery Symptomatic, congenital third-degree AV block Asymptomatic, congenital third-degree AV block associated with risk factors (pause > 3 times the baseline RR cycle, wide QRS, prolonged QTc, complex ventricular arrhythmia, mean heart rate < 50bpm, ventricular dysfunction)	I	B
Symptomatic, acquired second-degree AV block (Mobitz I) not associated with a reversible cause or nonessential drug Asymptomatic second-degree (Mobitz II), advanced, or third-degree AV block persisting for at least 5 days after heart valve surgery, revascularization, or AF surgery Permanent AF with a poor ventricular response with symptoms attributed to bradycardia	I	C
Congenital third-degree AV block in asymptomatic adults (age > 18 years)	IIa	B
In symptomatic patients clearly affected by significant first-degree AV block (pseudo-PM syndrome)	IIa	C

*AF: atrial fibrillation; AMI: acute myocardial infarction; AV: atrioventricular; PM: pacemaker; QTc: corrected QT interval; TAVI: transcatheter aortic valve implantation.*

#### 2.2.2. Intraventricular Blocks with 1:1 Atrioventricular Conduction

QRS complex abnormalities (eg, fascicular blocks and bundle branch blocks) are caused by a conduction delay or a block within one or more branches of the His-Purkinje system.

Conduction delay or right bundle branch block (RBBB) associated with a block in one fascicle of the LBB is named bifascicular block (the same terminology is used for LBB block [LBBB]). Clinical conditions that may result in IV block include genetic/hereditary, inflammatory, infectious, infiltrative, metabolic, ischemic, and degenerative causes.

The presence of IV block alone is rarely associated with symptoms but may be a marker of structural heart disease; the presence or development of LBBB may result in cardiac dyssynchrony and progressive LV dysfunction. Some studies have demonstrated a correlation between LBBB and CAD and HF.^57 , 58^The prevalence of progression of LBBB or bifascicular block to advanced AV block is low, approximately 1% per year.^59^LBBB is usually associated with higher mortality compared with other IVCDs.^60^In some neuromuscular diseases, PM implantation is recommended in patients with IV block because of the high incidence of complete AV block and sudden cardiac death.

Although an EPS may identify high-risk conduction disorders, this study is characterized by a variable degree of sensitivity and some degree of risks. In patients with syncope, the presence of IV block is a predictor of electrophysiological abnormalities.^61^

Regardless of symptoms, alternating bundle branch blocks (when QRS complex morphology spontaneously alternates between LBBB and RBBB) are also indicative of PM implantation because their presence suggests infranodal conduction system disease with high risk for severe complete AV block.

The recommendations for PM implantation in IV blocks are summarized in Table 4 .

**Table 4:** – Recommendations for permanent pacemaker implantation in intraventricular block

	Class of recommendation	Level of evidence
Permanent PM implantation is recommended in patients with syncope and bundle branch block with H-V interval ≥ 70ms or infranodal block on EPS without documented hemodynamically unstable VT Permanent PM implantation is recommended in patients with alternating bundle branch block regardless of symptoms New, persistent LBBB with QRS > 150ms for more than 48 hours after TAVI if PRi > 240ms	I	C
Patients with bifascicular block and unexplained syncope who did not undergo EPS (older people, frail patients, and recurrent syncope)	IIb	B
New, persistent LBBB with QRS > 150ms for more than 48 hours after TAVI if normal PRi	IIb	C
Asymptomatic patients with IV conduction disorder alone and 1:1 AV conduction in the absence of other indications for PM implantation	III	B

*EPS: electrophysiology study; LBBB: left bundle branch block; PM: pacemaker; PRi: PR interval; TAVI: transcatheter aortic valve implantation; VT: ventricular tachycardia.*

## 2.3. Carotid Sinus Syndrome

Carotid sinus syndrome (CSS) is characterized by a history of syncope associated with an exaggerated reflex response to mechanical stimulation of the carotid sinus occurring either spontaneously or with carotid sinus massage (CSM).^62^Syncope results from significant bradycardia and/or hypertension triggered by head movements or situations that cause involuntary compression of the neck and carotid sinus, although this correlation is not clinically evident in many patients.^63^

CSS is diagnosed by a pause > 3s (sinus node arrest or AV block) or a fall in systolic blood pressure (SBP) ≥ 50mmHg in the absence of conductor system depressants combined with reproduction of syncope during right- and left-sided CSM (5 to 10 s) in patients aged > 40 years. The patient should be placed in the supine position and tilted (tilt table test).^64^Reproducing syncope during CSM increases test specificity, whereas performing CSM in the supine position increases test sensibility.^63^

Reflex responses in CSS can be classified according to their hemodynamic profile into three types: cardioinhibitory (ventricular pause > 3s), mixed (ventricular pause > 3s associated with a fall in SBP ≥ 50mmHg), or vasodepressor (a fall in SBP ≥ 50mmHg alone). The incidence of CSS increases with age (< 50 years: ~0%, 50 to 59 years: 2,4%, 60 to 69 years: 9,1%, 70 to 79 years: 20%, and > 80 years: 40%).^63^

The evidence supporting permanent PM implantation in patients with CSS is based on small, controlled studies and retrospective observational studies.^65 - 68^

A literature review of 12 studies including 601 patients treated with PM and 305 controls found lower rates of syncopal recurrence in treated patients (0% to 20%) compared with controls (20% to 60%), although patient selection, patient position during CSM (horizontal or tilted), follow-up duration, and pacing mode were heterogeneous among studies.^68^A meta-analysis of three controlled studies with a mean follow-up duration of 3.3 years showed a significant reduction (76%) in syncopal recurrence in patients treated with PM compared with controls (9% and 38%, respectively; relative risk [RR]: 0.24; 95% CI 0.12-0.48).^68^

The Syncope And Falls in the Elderly – Pacing And Carotid Sinus Evaluation (SAFE PACE) study^69^evaluated 175 older patients with recurrent unexplained falls, no reported loss of consciousness, and cardioinhibitory response during CSM and suggested that a diagnosis of CSS should be suspected in such cases. The group that was randomly assigned to permanent PM implantation showed a significantly reduced rate of events (syncope: 53%; falls: 70%; physical trauma: 70%) during follow-up.

A study in which the diagnosis of CSS also included spontaneous pauses recorded by an implantable loop recorder found a 98% reduction in syncope burden after PM implantation (1.68 episodes per patient/year [95% CI 1.66-1.70] vs 0.04 episodes per patient/year [95% CI 0.038-0.042]).^70^When initial investigation with CSM and head-up tilt table test is negative, unexplained syncope and vasovagal syncope in patients aged > 40 years may be diagnosed with the help of an implantable loop recorder.^71^The recommendations for PM implantation in CSS are summarized in Table 5 .

**Table 5:** – Recommendations for permanent pacemaker implantation in carotid sinus syndrome and vasovagal syncope

	Class of recommendation	Level of evidence
Patients aged > 40 years with recurrent syncope and documented symptomatic spontaneous pause > 3s (sinus pause and/or AV block) or asymptomatic pause > 6 s	I	A
Patients aged > 40 years with recurrent syncope and cardioinhibitory (pause > 3s, sinus pause, and/or AV block) or mixed (pause > 3s + hypotension) response to CSM in the absence of conduction system depressants	IIa	B
Patients aged > 40 years with recurrent syncope and a symptomatic pause > 3s (sinus pause and/or AV block) induced by the tilt table test Unexplained recurrent falls in patients aged > 40 years with cardioinhibitory response (pause > 3s, sinus pause, and/or AV block) to CSM and no prodromes	IIb	B
Patients with syncope and no documented cardioinhibitory response	III	B
Asymptomatic patients with cardioinhibitory response to CSM	III	C

*AV: atrioventricular; CSM: carotid sinus massage.*

Importantly, asymptomatic older patients may present with carotid sinus hypersensitivity but no characterization of CSS and no indication for PM implantation. Conversely, patients with recurrent unexplained falls and cardioinhibitory response to carotid sinus compression (carotid sinus hypersensitivity) may benefit from a permanent PM.^69^

Regarding pacing mode in CSS, a study evaluating acute hemodynamic changes in patients with CSS undergoing CSM demonstrated that VVI (single-chamber) pacing was associated with an increased fall in SBP and an increased rate of persisting symptoms compared with DVI (dual-chamber) pacing.^72^Subsequent studies comparing long-term single-chamber vs dual-chamber pacing showed a trend towards lower rates of recurrent syncope and presyncope in patients with dual-chamber pacing^73 - 76^( Table 6 ).

**Table 6:** – Recommendations for pacing mode in carotid sinus syndrome and vasovagal syncope

	Class of recommendation	Level of evidence
Dual-chamber PM (DDD, O/R) in CSS and vasovagal syncope	IIa	B

*CSS: carotid sinus syndrome; PM: pacemaker.*

## 2.4. Vasovagal Syndrome

Vasovagal syncope is characterized by a history of loss of consciousness associated with an exaggerated neurally-mediated reflex that progresses with a sudden reduction in cerebral blood flow secondary to vasodilation and/or reduced heart rate. In most cases, syncope is secondary to a sudden and significant fall in blood pressure followed by varying degrees of bradycardia and generally preceded by prodromal manifestations such as malaise, sweating, feeling hot, pallor, dizziness, and then fatigue. Vasovagal syncope is often triggered by significant emotional stress, fear, or pain and is the main cause of syncope, especially in young people. Predisposing factors include prolonged orthostatism, closed or hot environments, venipuncture, and physical trauma, among others.

Based on changes in blood pressure and heart rate, vasovagal response can be classified into three types: type 1, or mixed response (a significant fall in blood pressure followed by a slight reduction in heat rate); type 2, or cardioinhibitory response (a significant reduction in heart rate < 40bpm or asystole > 3s); and type 3, or vasodepressor response (a significant fall in blood pressure with no significant reduction in heart rate).^77^

Despite sometimes being related to physical trauma and inability to perform activities that pose personal or collective risks, vasovagal syncope has a favorable long-term prognosis. Treatment is most often nonpharmacological, including guidance and changes in lifestyle. However, approximately 14% of patients present with severe forms of vasovagal syncope and require additional treatment (very frequent syncopal recurrence associated with impaired quality of life, short or absent prodromes, and increased risk of trauma, or with high-risk occupations such as professional driver, machine operator, airplane pilot, competitive athlete etc.). Patient age is the most important factor for choosing the best therapy.

There are few evidence-based therapeutic options for vasovagal syncope. Artificial cardiac pacing may be effective in patients with vasovagal syncope and dominant cardioinhibitory reflex; thus, clinical investigation should be focused on documenting the correlation between syncope and bradycardia.

The effectiveness of permanent PM insertion has been assessed in some randomized studies. The Vasovagal Pacemaker Study I (VPS-I) evaluated patients with recurrent syncope (≥6 syncopal spells) and a positive tilt table test (bradycardia < 60bpm or pause > 1s) who were randomly assigned to receive a PM (DDD mode with rate-drop response function) or clinical treatment.^78^Syncopal recurrence at 12 months was 22% (6/27) in the PM group vs 70% (19/27) in the clinical treatment group (RR 85.4%; 95% CI 59.7%-94.7%; 2p = 0.00002). The Syncope Diagnosis and Treatment (SYDIT) study randomly assigned patients with recurrent syncope (≥3 syncopal spells) and a positive tilt table test (bradycardia) to receive a PM (DDD mode with rate-drop response function) or clinical treatment (atenolol 100 mg/day).^79^Syncopal recurrence after a median follow-up of 135 days was 4.3% (2/46) in the PM group vs 25.5% (12/47) in the atenolol group (OR 0.133; 95% CI 0.028-0.632; p = 0.004). In the Vasovagal Syncope International Study (VASIS), patients were randomly assigned to receive a PM (DDI mode with rate hysteresis) or no treatment.^80^Syncopal recurrence after a mean follow-up of 3.7 years was 5% (1/19) in the MP group vs 61% (14/23) in the control group (p = 0.0006). The Vasovagal Pacemaker Study II (VPS-II) included patients with recurrent syncope (6 syncopal spells or over), although significant bradycardia during the tilt table test was not an inclusion criterion.^81^After PM implantation, patients were randomized to dual-chamber pacing (DDD) or sensing without pacing (ODO). Syncopal recurrence rates were 33% (16/48) in the DDD group vs 42% (22/52) in the ODO group, with no significant reduction in the risk of syncope (RR 30%; 95% CI -33% to 63%; p = 0.14). In the Third International Study on Syncope of Uncertain Etiology (ISSUE-3), the most important double-blind randomized trial published to date, patients aged > 40 years with spontaneous syncope documented by an implantable loop recorder associated with asystole > 3s or asystole > 6s without syncope were randomly assigned to dual-chamber pacing with rate-drop response function (PM ON) or sensing without pacing (PM OFF).^82^During a mean follow-up of 2 years, there was a significant reduction (57%) in the risk of syncopal recurrence (25% in PM ON arm vs 57% in the PM OFF arm; p = 0.039). All studies used dual-chamber pacing with rate-drop response function.

A small retrospective study compared conventional dual-chamber pacing with closed-loop stimulation (CLS) pacing and reported lower rates of syncopal recurrence in the latter.^83^

Recommendations for permanent PM implantation in vasovagal syncope are summarized in Table 5 . Recommendations for choosing a pacing mode are shown in Table 6 .

## 2.5. Hypertrophic Cardiomyopathy

Hypertrophic cardiomyopathy (HCM) is a common genetic cardiovascular disease characterized by LV hypertrophy in the absence of other cardiac abnormalities or systemic disorders capable of producing a degree of hypertrophy equivalent to that found in patients with this condition.^84^In hypertrophic obstructive cardiomyopathy (HOCM), there is a pressure gradient in the LV outflow tract (LVOT) – larger gradients are associated with more severe symptoms and increased mortality.^85^

For patients with symptoms caused by LVOT obstruction, therapeutic options include negative inotropic drugs, septal myotomy-myectomy operation, alcohol septal ablation, and heart transplantation.^86^In these patients, RV apical pacing changes the pattern of ventricular contraction and generates regional dyssynchrony. This results in late basal septal activation and reduced LV contractility, which reduces both the systolic anterior motion of the mitral valve and the LVOT pressure gradient.^87 , 88^However, VD caused by activation with a wide QRS complex alone reduces LV contractility and may lead to a reduced outflow tract gradient. Thus, in this case, the benefit is related to an adverse effect of the PM.

Reduced LVOT gradients with ventricular pacing have been demonstrated in three small randomized, controlled studies and several observational studies. However, improvement of symptoms and quality of life varied among studies.^89 - 93^A subgroup analysis suggests that patients aged > 65 years are more likely to benefit from dual-chamber pacing.^94^

A Cochrane systematic review has concluded that the benefits of ventricular pacing in HCM are based on gradient measurements and that evidence regarding relevant clinical endpoints are lacking.^95^In addition, gradient reduction is generally smaller in ventricular pacing compared with septal myectomy or ablation. Thus, the indication of dual-chamber pacing solely to reduce the LVOT gradient is restricted to very specific conditions: patients aged > 65 years with moderate hypertrophy, defined symptoms due to LVOT obstruction, and no indication for ICD.^93^

Overall, septal myectomy or ablation should be considered a first-line therapy in patients with HOCM and symptoms refractory to pharmacological treatment. Very severe cases may require heart transplantation.

In patients undergoing PM implantation for LVOT gradient reduction, a short AV interval must be programmed (AV interval in VAT = 100 ± 30 ms) to obtain maximal RV preexcitation without compromising diastolic ventricular filling.^96^In addition, maximal atrial pacing rate should be higher than the maximal rate exhibited by the patient during the stress test. Patients with HCM and very poor tolerance to an elevated heart rate are usually on beta-blockers, which results in a lower maximal rate during exertion; conversely, these patients are more susceptible to developing AF. Thus, automatic mode switching to DDI(R) mode should be used to avoid high-rate ventricular pacing in AF. If the atrial lead is inefficient in detecting AF, the maximal rate should be lowered.

Finally, a significant number of patients with HCM receive an ICD for preventing sudden death. For these patients, a dual-chamber DDD pacing device with a short AV interval may reduce the LVOT gradient and prevent or delay the need for additional interventions.

The recommendations for permanent PM implantation in patients with HCM are described in Table 7 .

**Table 7:** – Recommendations for permanent pacemaker implantation in hypertrophic cardiomyopathy

	Class of recommendation	Level of evidence
Patients in sinus rhythm with advanced, second-degree (Mobitz II), or third-degree AV block, spontaneous or after alcohol septal ablation or surgical myectomy (dual-chamber pacing is recommended)	I	B
Patients with HOCM and ICD indication should be considered for a dual-chamber pacing device	IIa	C
In patients with LVOT obstruction (gradient ≥50 mm Hg), dual-chamber pacing with a short AV interval may be considered in those who are refractory to clinical treatment, have no ICD indication, and cannot undergo alcohol septal ablation, surgical myectomy, or heart transplantation	IIb	C

*AV: atrioventricular; HOCM: hypertrophic obstructive cardiomyopathy; ICD: implantable cardioverter-defibrillator; LVOT: left ventricle outflow tract.*

## 2.6. Neuromuscular Diseases

Some neuromuscular conditions may cause progressive and insidious disease of the cardiac conduction system, such as Duchenne muscular dystrophy, facioscapulohumeral muscular dystrophy, X-linked dystrophy, myasthenia gravis, myotonic dystrophy, and Friedreich ataxia.^97^

Most manifestations are related to infranodal conduction disorders, resulting in fascicular blocks and third-degree AV blocks. Such findings are typical of Kearns-Sayre syndrome (progressive external ophthalmoplegia with pigmentary retinopathy), Guillain-Barré syndrome, myotonic dystrophy, Becker muscular dystrophy, and facioscapulohumeral muscular dystrophy.

Myotonic dystrophy and Kearns-Sayre syndrome are associated with a high incidence of conduction system disease, which often progresses rapidly and cannot be predicted by ECG or intracavitary recordings. The disease almost always affects the His-Purkinje system and can lead to Stokes-Adams attacks or sudden death, except when prevented by PM implantation.

In a study of 49 patients with myotonic dystrophy (mean age: 46 years, HV interval ≥ 70ms), high-degree AV blocks were recorded in 47% of patients after PM implantation, despite no documented evidence of bradycardia at baseline.^98^The authors concluded that permanent PM implantation should be considered in patients with myotonic dystrophy and prolonged HV interval (≥ 70ms) even if they are asymptomatic or if bradycardia is found on ECG.

In patients with neuromuscular diseases, waiting for a documented AV block may result in significant risk of sudden death or syncope. Thus, permanent PM implantation should be considered early in the course of neuromuscular disease upon the presence of conduction abnormalities even if the patient is asymptomatic ( Table 8 ).

**Table 8:** – Recommendations for permanent pacemaker implantation in neuromuscular diseases

	Class of recommendation	Level of evidence
Symptomatic or asymptomatic patients with second-degree (type II or advanced) or third-degree AV block	I	B
Asymptomatic patients with first-degree AV block (PR interval > 240ms) or wide QRS complex (QRS > 120ms) (in myotonic dystrophy)	IIb	C

*AV: atrioventricular.*

ECG abnormalities such as nonsinus rhythm, QRS > 120ms, PRi > 240ms, second- or third-degree AV block, and atrial tachyarrhythmias were independent predictors of sudden death in patients with myotonic dystrophy type I.^99^

## 2.7 Obstructive Sleep Apnea Syndrome

Bradyarrhythmias, such as sinus bradycardia, sinus pauses, advanced or type I second-degree AV block, and junctional escape rhythm, are common during sleep, especially in young healthy people who are physically fit. Direct and indirect data have shown an association with vagal hypertonia. In nearly all cases, these findings are physiological and require no specific treatment. However, cardiac arrhythmias have been observed in obstructive sleep apnea (OSA) syndrome.^100^

The most significant cases are related to Pickwick syndrome, obesity, hypertension, metabolic syndrome, anatomic and/or functional airway obstruction (eg, constitutional, macroglossia, soft palate hypertrophy, and tonsillar and adenoid hypertrophy), chronic lung diseases, and neurological diseases, among others.

During apnea, airway obstruction leads to oxygen desaturation that may result in severe hypoxemia with consequent development of atrial and ventricular brady- and tachyarrhythmias.^101^

Primary treatment is intended for apnea correction and weight loss. Continuous positive airway pressure devices for respiratory support during sleep may be of great importance, as their use has resolved bradyarrhythmias in a large number of cases.^102^Thus, there is no primary indication for PM implantation in OSA-related bradyarrhythmias.

In clinical practice, OSA is commonly observed in a setting characterized by vagal hypertonia, nocturnal bradyarrhythmia, and AF commonly triggered by bradycardia (bradycardia-tachycardia syndrome).^102^When airway obstruction correction is not sufficient to improve the status, radiofrequency ablation may be indicated (cardioneuroablation [CNA] and/or AF ablation). In exceptional cases, MP implantation may facilitate the treatment of AF (beta-blockers or other antiarrhythmics).

Increased vagal tone can be well characterized by temporal and spectral analyses of RR variability on 24-hour Holter monitoring with a comparison of wake and sleep periods. RR variability and the number of pauses > 2.5 s pre- and 1-year post-CNA were compared in a study including 18 patients with a history of OSA and tachycardia-bradycardia syndromes and/or vagal hypertonia. There was a significant reduction in RR variability (pre-CNA standard deviation of the NN intervals 131.2 ± 38 ms vs 91.9 ± 37 ms 11 months after CNA, p = 0.0001). Furthermore, the number of pauses significantly decreased from 6.5 ± 9.4 pre-CNA to 1.1 ± 3 within 11 months of CNA (p = 0.03). No patient received a PM.^103^Thus, PM implantation is currently reserved for patients with a compromised conduction system ( Table 9 ).

**Table 9:** – Recommendations for permanent pacemaker implantation in obstructive sleep apnea syndrome

	Class of recommendation	Level of evidence
Patients with nocturnal bradyarrhythmias (asymptomatic while awake), OSA syndrome, and no significant heart disease who received no specific treatment	III	C

*OSA: obstructive sleep apnea.*

## 2.8. Congenital Long QT Syndrome

Congenital long QT syndrome (LQTS) is a channelopathy caused by abnormal cardiac repolarization and features a prolonged QT interval, ventricular arrhythmias (ventricular extrasystole, polymorphic ventricular tachycardia, torsade de pointes), and a history of syncope and/or sudden death. There is generally a family history of LQTS in close relatives, and the most common pattern of inheritance is autosomal dominant (Romano-Ward syndrome), although the condition may also be autosomal recessive (very rare) with associated deafness. These two types account for 90% of cases; however, there are currently 14 known different types of congenital LQTS.^104^

Some patients with LQST do not present with spontaneous clinical manifestations, although certain conditions, such as medication use, physical stress, and electrolyte changes, may be triggers.

The QT interval (QTi) can be measured in any lead, although leads II and V_5_are more commonly used.^105^The QTi is measured from the beginning of the QRS complex to the end of the T wave, excluding the U wave. Given that QTi physiology changes inversely to heart rate, the original value should be corrected for heart rate, most commonly with the Bazett formula.^106^In this formula, the measured QTi is divided by the square root of the preceding RR interval (the units are measured in seconds), resulting in the corrected QT (QTc) interval. The normal limits are 450ms and 460ms for men and women, respectively. A QTc interval > 480ms 4 minutes after the stress test is highly suggestive of LQST. Approximately 20% of cases with a positive genotype have a normal QTi.

In different types of congenital LQST, a delay in cellular repolarization occurs either due to a reduction in potassium channel function or an increase in sodium channel function (delayed channel inactivation) and manifests as an increased QTi. Electrophysiological abnormalities are apparently heterogeneous and become significantly prominent in the presence of conditions such as autonomic stimulation, physical and mental stress, electrolyte changes, drug action, and ischemia, among others, resulting in electrical instability, extrasystole, polymorphic tachycardia, torsade de pointes, ventricular fibrillation, and sudden death.

Whenever possible, the type of LQTS (specifically types I, II, and III) should be defined according to clinical and ECG manifestations, as there is a recommended treatment for each type.

Types I and II are the most common and originate from a decrease in potassium channel function, whereas type III is caused by an increase in sodium channel function.^107^Congenital LQTS manifestations typically appear during childhood or adolescence and usually affect male patients (adolescence) earlier than female patients (adulthood). Syncope is the most common manifestation and usually occurs between 5 and 15 years of age. A family history of sudden death is a strong predictor of mortality. Overall, the longer the QTc interval, the higher the risk of sudden death.

In congenital LQTS type I, arrhythmias are usually triggered by physical exertion, especially swimming. In type II, they are commonly induced by mental stress caused, for example, by loud noises, especially during rest or sleep. As for type III, arrhythmias typically occur during rest or sleep without a clear relationship with stress.

It is absolutely essential that patients avoid electrolyte disturbances such as hypokalemia and the use of drugs that may trigger fatal arrhythmias. Several websites provide a list of drugs that may potentially increase the QTc interval (http://www.crediblemeds.org) and should always be consulted before taking any medication.

All patients (symptomatic, asymptomatic, and “silent” carriers) should significantly reduce the level of physical activity. Competitive sports are contraindicated. Certain type-related triggers, such as strenuous swimming in LQTS type I and very loud noises in LQTS type II, should be avoided.^108^Noncompetitive recreational sports may be cautiously considered in patients with LQTS type III if there is easy access to an automated external defibrillator. Drugs and substances that prolong repolarization, such as potassium channel blockers, which can induce torsade de pointes even in asymptomatic cases, are absolutely contraindicated. Sympathomimetics should also be avoided. Patients are advised to carry a list of prohibited drugs.

Pharmacological treatment of LQTS types I and II is based on beta-blockers, and propranolol and nadolol are the most effective drugs. Metoprolol appears to be less effective and should be avoided.^109^

Retrospective studies have demonstrated the unquestionable benefits of beta-blockers and denervation surgery (removal of the left stellate ganglion), with a mortality of 9% in the treated group vs 60% in the untreated group.

The QTc interval can be experimentally reduced with potassium-channel activators such as nicorandil in LQTS type I or spironolactone combined with oral potassium in LQTS type II. LQTS type III may benefit from sodium-channel blockers such as mexiletine and flecainide, which can shorten the QTc interval; however, flecainide may induce a Brugada phenotype. There are reports of successful treatment of electrical storms in LQTS type III with mexiletine, which is recommended by some professionals when the QTc interval is very long.

There is currently no primary indication for PM implantation for preventing sudden death in LQTS, given the greater safety provided by ICD.^110^However, a PM may be recommended for patients with AV block or ventricular arrhythmias triggered or aggravated by bradycardia or pauses, provided there is no history of recovered sudden death or high-risk signs such as congenital deafness, syncope, documented complex ventricular arrhythmias, family history of sudden death, female sex, and QTc > 0.60s. PM insertion combined with beta-blocker therapy is occasionally indicated to avoid drug-induced bradycardia. A cardiac pacing rate above the spontaneous sinus rate can reflexively inhibit sympathetic action and be quite helpful in controlling arrhythmic storms. A PM should only stimulate the atrium (avoid VD) and can be programmed to a higher rate (eg, 80 ppm), which shortens QTc duration. Furthermore, activation of the frequency response sensor can ensure chronotropic adaptation, which is impaired by beta-blockers ( Table 10 ).

**Table 10:** – Recommendations for permanent pacemaker implantation in congenital long QT syndrome

	Class of recommendation	Level of evidence
Low-risk patients (no high-risk conditions*) with bradyarrhythmia (sinus or AV block) aggravated or not by beta-blockers, especially in LQTS type III	IIb	C

*High-risk conditions in congenital LQTS: congenital deafness, syncope, documented complex ventricular arrhythmias, family history of sudden death, female sex, corrected QT interval >0.60 s. AV: atrioventricular; LQTS: long QT syndrome.

Denervation surgery is an alternative in patients with recurrent syncope despite the use of regular full-dose beta-blockers or when optimal drug therapy (ODT) cannot be prescribed (eg, asthma).

## 2.9. Cardiac Transplantation

The rates of permanent PM implantation after cardiac transplantation vary between 2% and 24% and have significantly decreased in patients undergoing bicaval anastomosis vs those undergoing biatrial anastomosis.^111 - 113^

SND is the most commonly reported disorder in these patients, and possible causes include surgical trauma, damage to the sinoatrial artery due to trauma or ischemia, prolonged ischemic time of the donor heart, cardiac denervation, and baseline characteristics of the donor heart.^114^

Approximately 10% of patients requiring a PM have an AV conduction disorder (especially second- or third-degree AV blocks), which are probably due to inadequate graft lpreservation.^115^

Bradycardia often occurs in the early postoperative period following cardiac transplantation and affects approximately two thirds of patients, although it tends to remit spontaneously. However, if bradycardia persists for a few weeks and the patient develops symptoms, PM implantation may be necessary.

Studies have shown that several patients with SND and bradycardia implanted with a PM become device-independent after 3 months. However, patients with early AV block often require long-term cardiac pacing^116 , 117^( Table 11 ).

**Table 11:** – Recommendations for permanent pacemaker implantation after cardiac transplantation

	Class of recommendation	Level of evidence
A pacemaker should be considered in symptomatic patients with bradyarrhythmias or chronotropic incompetence that are not likely to resolve spontaneously or, even if transient, may persist for months	IIa	C

## 2.10. Choosing the Type of Pacemaker and Pacing Mode

Randomized clinical trials have not reported any impact of atrial or AV pacing (AAI/DDD) on survival compared with ventricular pacing (VVI) alone; however, AF occurrence, syncopal incidence, and PM syndrome rates were reduced.^32 - 35^A systematic review reported a significant reduction in stroke (HR: 0.81) and AF (HR: 0.80) incidence rates with sequential AV pacing compared with ventricular pacing.^36^

In patients with persistent bradycardia, dual-chamber pacing is the preferred choice. The Danish Multicenter Randomized Trial on Single Lead Atrial Pacing vs Dual Chamber Pacing in Sick Sinus Syndrome (DANPACE) found that AAIR pacing was associated with a higher incidence of paroxysmal AF (HR: 1.24) and a two-fold increase in reoperations compared with DDDR pacing.^118^Ventricular pacing should be avoided in patients in sinus rhythm as it may cause AF and worsening HF^119^( Figure 1 ). Importantly, programming an excessively long AV interval to avoid ventricular pacing in patients with spontaneous AV conduction may be hemodynamically harmful in patients with a marked first-degree AV block and may result in diastolic mitral regurgitation, pseudo-PM syndrome, and lAF.^120^

**Figure 1:** – Algorithm for preservation of intrinsic atrioventricular conduction. AF: atrial fibrillation; AVI: algorithm for intrinsic atrioventricular conduction preservation.

Activation of the rate variation sensor may be beneficial in patients with chronotropic incompetence. The Advanced Elements of Pacing Trial (ADEPT) compared the impact of DDDR vs DDD mode on quality-of-life improvement in 872 patients with chronotropic incompetence. At 6-month follow-up, patients randomly assigned to DDDR mode had a higher peak heart rate compared with those assigned to DDD mode (113.3 ppm x 101.1 ppm; p < 0.0001).^121^However, at 1 year, the Specific Activity Scale scores and the secondary quality-of-life endpoints did not differ significantly between groups.

## 2.11. Direct Stimulation of the Cardiac Conduction System (His Bundle, Left Bundle Branch)

LV remodeling and consequent LV dysfunction promoted by RV pacing-induced dyssynchrony warrants the search for alternative pacing sites in patients with bradyarrhythmias requiring artificial ventricular pacing.

The MOST study reported that RV apical pacing in patients with sinus dysfunction led to a significant increase in AF episodes and HF hospitalizations.^35^More importantly, these adverse events were directly associated with the cumulative percentage of ventricular pacing.

Consequently, the search for a pacing strategy that preserves interventricular and IV synchrony and corrects bradyarrhythmias is a relevant clinical necessity that has led to physiological pacing.^122 , 123^

Direct stimulation of the conduction system is the most physiological form of artificial pacing because it reproduces the natural electrical activation of the heart. The stimulus travels through the specialized conduction pathways (His-Purkinje system) and avoids RV pacing-induced dyssynchrony.^124^

A nonrandomized study of 202 patients who were followed-up for 2 years reported that His bundle pacing was superior to RV apical pacing. In patients with 40% of ventricular pacing, hospitalizations due to HF were significantly reduced (15% vs 2%, p = 0.02).^125^

His bundle pacing has some limitations, including technical difficulties to identify the best pacing site (higher implant times), higher pacing thresholds, lower intracavitary sensing amplitudes, and abnormal inhibition by cross-sensing.^126^

Deep septal pacing with direct capture of the LBB or a nearby area (the lead is inserted with a sheath into the left side of the interventricular septum) is a viable option that preserves narrow QRS complexes and prevents dyssynchrony. From a functional perspective, despite technical differences between direct capture of the LBB and capture of the LBB area, pacing of the LBB area promotes synchronism equivalent to that of direct His bundle pacing.

In a small series of 10 patients with SND, Mafi-Rad et al. demonstrated the feasibility of LV pacing of the interventricular septum resulting in a narrow RBBB-like QRS morphology, stable thresholds, and improved hemodynamic performance (measured by dP/dt) compared with RV apical pacing.^127^Subsequently, Huang et al. demonstrated the feasibility of direct LBB capture via deep septal pacing for LBBB correction. Some criteria for defining LBB capture were established, and patients should meet at least 3 of the following: 1) presence of LBB potential recorded on the lead electrogram; 2) LV activation time < 90ms with a stable pacing output of 2V or 5V; 3) incomplete RBBB on ECG; 4) evidence of selective and nonselective LBB capture; and 5) evidence of direct LBB pacing via a concomitant lead in the His bundle or the left septum.^128 , 129^

Physiological pacing via His bundle or LBB has been effectively used in different settings of bradyarrhythmia requiring ventricular pacing ( Table 12 ).

**Table 12:** – Recommendations for physiological pacing (His bundle, left bundle branch) in the treatment of bradyarrhythmias

	Class of recommendation	Level of evidence
SND with an indication for a conventional pacemaker in patients with delayed intraventricular conduction	IIa	C
Permanent atrial fibrillation with an indication for AV junction ablation for heart rate control	IIa	C
AV block without LV systolic dysfunction	IIb	B

*AV: atrioventricular; LV: left ventricular; SND: sinus node disease.*

Compared with RV pacing, both His bundle and LBB pacing modalities have been shown to be safe and stable, to improve synchrony and QRS duration measurements, and to tend to improve ventricular function. Compared with LBB pacing, direct His bundle pacing generates a normal and apparently more physiological QRS complex, but at the expense of higher implant times, higher thresholds, and lower R-wave amplitudes.^130 , 131^

The evolution of implantable technologies, the availability of generators with specific algorithms for detection of proportional energy expenditure, and the results of long-term controlled studies will potentially establish physiological pacing as the preferred strategy in the near future.^132^

## 2.12. Leadless Pacing

Artificial cardiac pacing is not free of problems. The incidence of complications involving leads and pulse generators, especially in the subcutaneous pocket, increases over the follow-up period and may affect more than 10% of patients with CIEDs.

Implantation of conventional systems is associated with risk of pneumothorax, hemothorax, lead dislodgement, venous occlusion, tricuspid valve insufficiency, and infection (which may occasionally lead to endocarditis). Furthermore, subcutaneous implantation is associated with occurrence of pocket hematoma and infection not only during the first surgical procedure but also during the generator replacement procedure.^133 , 134^Of all complications listed, infective endocarditis warrants special attention because of its association with increased morbidity and mortality.^135 , 136^

Since PM leads are the main source of problems and complications, implantable technologies have naturally advanced towards prioritizing solutions that do not require them. Within this context, leadless pacing has emerged as a technological evolution with potential advantages over conventional pacing. Two leadless systems were initially introduced in the market, Nanostim leadless cardiac pacemaker (Abbott Medical Inc. Abbott Park, IL, EUA) and Micra transcatheter pacing system (Medtronic, Inc., MN, EUA), but only the latter is currently available.

The Micra system is a small “capsule” (26mm in length x 6.7 mm in diameter) containing a lead and a pulse generator which is implanted in the cardiac chamber via transvenous route. The procedure is relatively simple and involves femoral vein catheterization. A large-caliber sheath (24 French) in introduced in the inferior vena cava and advanced into the right atrium. A delivery system is then inserted inside the sheath, allowing deployment of the device in the RV. Initially, the RV apex was the recommended site for device implantation, but interventricular septal implantation is currently recommended.^137^

A multicenter, single-arm, prospective clinical trial evaluated the Micra system and included 725 patients (mean age was 75.9 ± 10.9 years, 58.8% were male) with an indication for single-chamber permanent PM implantation. The primary objective was to evaluate device efficacy (threshold capture at 6-month follow-up) and safety (major complications). Implantation was successful in 99.2% of cases. There were 28 major complications in 3.4% of patients, including perforation or pericardial effusion (1.6%), complications in the venous access site (0.7%), and elevated pacing threshold (0.3%). There were no dislodgements or device emboli. The mean values of R-wave amplitude, pacing threshold, and impedance remained stable. The rate of complications was compared with that of a population of more than 2,000 patients (historical controls from other clinical trials of same-brand conventional PMs). The Micra study found a lower rate of major complications (HR: 0.49, 95% CI 0.33-0.75, p = 0.001), including fewer hospitalizations (2.3% vs 3.9%) and fewer system revisions (0.4% vs 3.5%). There was also a low rate of infections, which were unrelated to the implantation or the device.^138^These results were then confirmed at 1-year follow-up.^139^Another analysis of the same cohort reported improved quality-of-life parameters at 3 and 12 months and high levels of patient satisfaction. The Micra system was also associated with less activity restrictions compared with conventional systems.^140^

The Micra system has also been evaluated in “real-world” registries. The largest is a multicenter registry (96 centers in 20 countries) that aims to include 1,830 patients. The primary endpoint is occurrence of complications within 30 days of implantation. The published results of the first 795 patients showed a high implant success rate (99.6%) and a low rate of major complications (1.5%). Conventional system implantation was contraindicated in approximately 20% of patients, mainly due to vascular access problems. In addition, there were five pericardial effusion events in the study population, two of which had to be drained.^141^Small single-center studies evaluating leadless PMs in real-world settings have confirmed the findings of high implant success rates and low complication rates.^142 - 144^

The safety of leadless system implantation has also been evaluated in specific populations, such as patients undergoing hemodialysis or conventional device extraction due to infection.

The Nanostim device was assessed in a multicenter observational study,^145 , 146^whose results at 6-month follow-up did not differ from those obtained in the Micra studies. However, Nanostim was withdrawn from the market because of unexpected rates of battery failure causing loss of pacing output and PM communication in approximately 0.5% of cases.^147^

In addition to the previously mentioned intraoperative complications, there are two situations that remain uncertain. First, the impact of the device on tricuspid valve function is still unknown. An initial study of 23 patients reported no effect on tricuspid valve function at 2-month follow-up.^148^A more recent study including 53 patients revealed that leadless PMs affect valve function and may cause or worsen tricuspid regurgitation,^149^probably due to mechanical interference of the device in the subvalvular apparatus. Acute complications of the procedure and PM-induced dyssynchrony are the least likely causes. Patients with septal positioning of the leadless PM (currently recommended because of lower risk of perforation) were associated with tricuspid regurgitation, which was probably explained by greater proximity to the valve and the subvalvular apparatus.

Another situation that remains unresolved is how to manage the device at end of service. A worldwide experience with early retrieval of the Micra system – range 1 to 95 days – reported good outcomes and low complication rates.^150^A case report of percutaneous device extraction performed 4 years after implantation has also been published.^151^However, how the device will behave in the long term and whether extraction is possible (or whether another device should be implanted in another site) remain unknown.^152 , 153^

Importantly, with the emergence of new technologies, unreported complications may arise as the follow-up periods become longer. In addition, there are no current randomized studies comparing conventional PMs with leadless PMs.

The currently available device is single-chamber and rate-responsive (VVIR). Overall, the indications for leadless PMs are the same as those for single-chamber PMs – mostly for symptomatic bradycardia not requiring an atrial lead ( Table 13 ).

**Table 13:** – Recommendations for leadless pacemaker implantation

	Class of recommendation	Level of evidence
A leadless PM should be recommended in patients with compromised conventional devices (lead fracture after extraction due to infection) and severe venous obstructions	IIa	C
A leadless PM is an acceptable option in patients with atrial fibrillation and low ventricular response, provided that the advantages and limitations are discussed with the patient	IIb	B
A leadless PM is an acceptable option in patients in sinus rhythm with an indication for PM implantation with limited stimulation, provided that the advantages and limitations are discussed with the patient (eg, rare sinus pauses or rare paroxysmal AV block) A leadless PM may be considered in patients with complete AV block who could be recommended single-chamber ventricular pacing (oldest-old patients, sedentary patients, bedridden patients), provided that the advantages and limitations are discussed with the patient	IIb	C
A leadless single-chamber ventricular PM should be recommended in patients in sinus rhythm with complete AV block who are candidates for conventional dual-chamber pacing when maintenance of AV synchronism is desirable	III	C
A leadless PM is not indicated for children and young people (there is uncertainty about how to manage the device at end of service)	III	C

*AV: atrioventricular; PM: pacemaker.*

One of the main factors limiting the use of leadless PMs is the high cost of the devices, as demonstrated in a recent survey by the European Heart Rhythm Association (EHRA).^154^

Conceptually, the best candidates for leadless PM implantation would be those with contraindications to conventional devices, such as no vascular access, risk of new surgery, hemodialysis, and previous infection of conventional systems. Some studies (mostly single-center) have demonstrated the effectiveness of leadless PMs in this subgroup of patients,^155 - 157^who were also evaluated in the aforementioned “real-life” studies.

The implant technique for leadless PMs is different from that of conventional PMs; thus, physicians should be experienced in cardiac pacing, femoral vascular access, and handling of large-caliber sheaths and RV leads. Cardiac perforation is uncommon but, when it occurs, it may affect the RV or the atrium and auricle and usually requires emergency surgical intervention.^158 , 159^

## 3. Recommendations for Multisite Pacemaker Implantation/Cardiac Resynchronization Therapy

### 3.1. Patients in Sinus Rhythm

Despite ODT, many patients with HF with reduced LVEF (HFrEF) develop persistent symptoms and significant LV systolic dysfunction. Moderate or severe mitral regurgitation, reduced myocardial functional reserve, and VD are the most common factors related to poor response to ODT. In VD settings, CRT alone (through atrio-biventricular pacing) or combined with an ICD has been considered an excellent therapeutic option for patients with LBBB. CRT is intended to correct electromechanical dysfunctions in patients with HFrEF and VD and, consequently, improve LV performance.

A surface ECG is the method of choice when investigating VD symptoms and selecting patients for CRT. Although imaging methods such as echocardiography can detect mechanical VD, tissue Doppler was unable to identify patients who responded to CRT in the Predictors of Response to CRT (PROSPECT) study.^160^The Comparison of Medical Therapy, Pacing, and Defibrillation in Heart Failure (COMPANION)^161^and the Cardiac Resynchronization in Heart Failure (CARE-HF) studies^162^were the first large-scale randomized trials to test the effect of CRT on the clinical outcomes of total mortality and hospitalization rate. The studies found that CRT increased survival when combined with ODT.

Those results were corroborated by a 2006 meta-analysis of 8 clinical trials including a total of 3,380 patients.^163^In a mean follow-up duration of 29.4 months, there were 524 deaths, with significant reductions in mortality (OR: 0.72, 95% CI 0.59-0.88) and HF hospitalization rates (OR: 0.55, 95% CI 0.44 to 0.68) with CRT. All trials reported a significant improvement in quality of life (3 to 6 months), although follow-up criteria were heterogenous among studies. Furthermore, the number needed to treat (NNT) was estimated at 11 (11 devices need to be implanted to save 1 life in 2.5 years). Considering the average longevity of CRT devices (6 years), five devices would need to be implanted to avoid 1 death.^164^

Those trials supported the first recommendations for CRT as an adjunctive therapy to ODT in patients with advanced HFrEF (LVEF ≤ 35% and NYHA functional class III or IV despite ODT for more than 3 months) and VD. VD was confirmed by the presence of IVCD on ECG. It should be noted that the COMPANION study also observed greater clinical benefits when combining CRT with ICD (CRT-D).

The Multicenter Automatic Defibrillator Implantation Trial With Cardiac Resynchronization Therapy (MADIT-CRT),^165^the Resynchronization Reverses Remodeling in Systolic Left Ventricular Dysfunction (REVERSE) study,^166^and the Resynchronization-Defibrillation for Ambulatory Heart Failure Trial (RAFT) were subsequently published.^167^These studies compared CRT-D vs ICD alone in patients with LVEF ≤ 40% (REVERSE) or ≤ 30% (MADIT-CRT and RAFT) and NYHA functional class I-II (REVERSE and MADIT-CRT) or II-III (RAFT). Study results, which were later corroborated by a meta-analysis, confirmed that both CRT plus ODT and CRT-D were beneficial in reducing total mortality.^168^

A meta-analysis of 5 randomized clinical trials including patients with asymptomatic HFrEF (NYHA functional class I) or NYHA functional class II HF demonstrated a significant reduction in total mortality and HF hospitalization rates in patients with NYHA functional class II HF.^165 - 167 , 169 , 170 165 , 166 , 167^Only 9% of patients had NYHA functional class I HF, and CRT significantly reduced HF hospitalization but not total mortality among them.^171^The results show that early CRT in asymptomatic patients with HFrEF may reduce HF progression possibly by means of reverse ventricular remodeling. However, the potential benefits of CRT in patients with NYHA functional class I HFrEF should be carefully weighed against possible adverse events and costs associated with CRT implantation.

A QRS duration ≥ 120ms as a cutoff point for CRT indication is based on the inclusion criteria of the COMPANION^161^and CARE-HF studies.^162^However, the results of the Echocardiography Guided Cardiac Resynchronization Therapy (EchoCRT) study revealed that cardiovascular mortality increased in the subgroup of patients with QRS <130 ms undergoing CRT.^172 , 173^These findings were corroborated by a meta-analysis showing limited benefits of CRT in patients with QRS < 140ms. Indeed, the longer the QRS duration, the better the response to CRT.^174 , 175^Furthermore, patients with LBBB and a QRS duration ≥ 150ms benefit the most from CRT.^165 , 176 , 177^A meta-analysis of 13 large studies including 12,638 patients confirmed the benefit of CRT in patients with LBBB and the higher risk of death from heart pump failure in patients with wider QRS complexes.^175^

As for IVCD type, a subanalysis of large studies suggests that patients with a wide QRS complex and no LBBB respond poorly to CRT. Data from the REVERSE study, which compared an active CRT group (CRT ON) vs a control group (CRT OFF), revealed that patients without LBBB had no reverse LV remodeling regardless of QRS duration.^178^Similarly, a study evaluating the MADIT-CRT population demonstrated no clinical benefits from CRT-D in 537 patients with mild HFrEF and no LBBB, regardless of QRS morphology and duration.^179^

Importantly, only a small number of patients with RBBB were included in the large studies, which precludes any definitive conclusions about the effects of CRT on this patient population.^180^

Conversely, although CRT was not beneficial in patients without LBBB, a recent real-world observational study using information from the National Cardiovascular Data Registry (USA) evaluated the clinical response to CRT-D vs ICD in 11,505 patients without LBBB. In patients with nonspecific IVCD and QRS ≥ 150ms, CRT reduced mortality and HF hospitalization rates, whereas those with QRS < 150ms responded poorly to CRT, with increased mortality and hospitalization rates.^181^There were also increases in risk of death and HF hospitalization rate in patients with RBBB treated with CRT-D. These findings are consistent with those reported by Bilchick et al.,^182^whose study included Medicare data. Additionally, Pastore et al.^183^reported that patients with typical RBBB (classically defined as QRS duration > 120ms; rsr’, rsR’, or rSR’ in leads V_1_or V_2_; and S-wave duration > R wave or > 40ms in leads I and V_6_)^184^responded poorly to CRT. Therefore, the current literature suggests that CRT is indicated in patients with HFrEF, nonspecific IVCD, and a QRS duration ≥ 150ms and that greater caution is required when recommending this therapy for patients with typical RBBB.

Indications for CRT in patients in sinus rhythm are listed in Table 14 .

**Table 14:** – Recommendations for cardiac resynchronization therapy in patients in sinus rhythm

	Class of recommendation	Level of evidence
CRT is indicated in patients in sinus rhythm with symptomatic HFrEF, LBBB, QRS ≥ 150ms, and LVEF ≤ 35% despite ODT	I	A
CRT is indicated in patients in sinus rhythm with symptomatic HFrEF, LBBB, QRS between 130 and 149 ms, and LVEF ≤ 35% despite ODT	IIa	B
CRT may be helpful in outpatients in sinus rhythm with NYHA functional class III or IV HFrEF, non-LBBB intraventricular conduction disorder, QRS ≥ 150ms, and LVEF ≤ 35% despite ODT	IIa	B
CRT may be helpful in outpatients in sinus rhythm with NYHA functional class III or IV HFrEF, non-LBBB intraventricular conduction disorder, QRS between 130 and 149ms, and LVEF ≤ 35% despite ODT	IIb	B
CRT is not indicated in patients with QRS <130ms and no other indication for right ventricular pacing	III	A

*CRT: cardiac resynchronization therapy; HFrEF: heart failure with reduced ejection fraction; LBBB: left bundle branch block; LVEF: left ventricular ejection fraction; NYHA: New York Heart Association; ODT: optimal drug therapy.*

### 3.2. Patients with Atrial Fibrillation

The prevalence of AF in patients with HF varies according to HF severity. The condition affects 5% of patients with NYHA functional class I HF and 40% of those with NYHA functional class IV HF.^185^

Data on the effect of CRT on patients with AF and HF are limited but suggest benefits, although less so than in patients in sinus rhythm. This is explained by some unique AF features, such as loss of AV synchronism, higher risk of synchronized ventricular pacing failure due to difficulties in controlling heart rate and occurrence of fusion and pseudofusion beats, and increased incidence of ICD shock delivery (appropriate or inappropriate), hospitalization, and mortality.^186 - 190^

Approximately one-quarter of patients undergoing CRT develop AF; however, most randomized controlled trials demonstrating the benefit of CRT excluded patients with AF (eg, CARE-HF and COMPANION).^191^

The CARE-HF study compared CRT vs ODT and found that, although mortality was higher in patients who developed AF during follow-up, they still benefited from CRT according to the primary objectives of the study.^192^

CRT-D was not superior to ICD alone in the subgroup of 229 patients with AF in the Resynchronization-Defibrillation for Ambulatory Heart Failure Trial (RAFT). However, less than one-third of patients received more than 95% of biventricular pacing during 6-month follow-up.

A meta-analysis^193^of 5 studies (4 prospective cohorts and the MUSTIC randomized clinical trial^194^) compared the impact of CRT on 797 patients in sinus rhythm vs 367 patients with AF (AV junction [AVJ] ablation was performed in 56 patients). Improvement in NYHA functional class did not differ between groups, although the Minnesota quality-of-life scores and the 6-minute walk test results were significantly improved in the sinus rhythm group.

In a meta-analysis^195^of 23 observational studies including 7,495 patients undergoing CRT, patients with AF (25%) showed higher rates of nonresponse to CRT (34.5% vs 26.7%) and mortality (10.8% vs 7.1%) compared with patients in sinus rhythm. Furthermore, the presence of AF was associated with lower impact of CRT on quality of life, 6-minute walk distance test and LV end-diastolic volume, but with similar result in the improvement of the LVEF.

The Multisite Stimulation in Cardiomyopathies (MUSTIC-AF)^196^study enrolled 59 patients with HF, AF, and bradycardia (ventricular pacing with paced QRS ≥ 200ms) who were randomly assigned to RV pacing or biventricular pacing with 3-month crossover phases. AV node ablation was performed in 63% of patients. Study limitations included the small number of patients that concluded the crossover phases (only 39%), which precludes any conclusions. In the intention-to-treat analysis, exercise tolerance and peak oxygen uptake did not differ between groups. However, in the 37 patients who received effective therapy (97-100% of biventricular pacing), 6-minute walked distance and peak oxygen uptake increased significantly.

In the Post AV Nodal Ablation Evaluation (PAVE),^197^Optimal Pacing Site (OPSITE),^198^and AV

Node Ablation with CLS and CRT Pacing Therapies for Treatment of AF (AVIL CLS/CRT)^199^studies, CRT moderately but significantly improved quality of life, NYHA functional class, and LVEF compared with RV apical pacing in patients with AF and varying degrees of LV dysfunction undergoing AV node ablation.

The Acute Decompensated Heart Failure National Registry (ADHERE)^200^compared patients with HF, QRS ≥ 120ms, and LVEF ≤ 35% undergoing CRT-D implantation (n = 4,471) vs those who did not undergo device implantation (n = 4,888). CRT-D was associated with lower risks of mortality and readmissions. The same association was observed in the subgroup of 3,357 patients with AF.

CRT requires biventricular pacing for most of the time to be beneficial, avoiding intrinsic conduction as much as possible. In patients with AF and rapid AV conduction, this requirement may be difficult to meet.

Boriani et al.^201^evaluated 1,404 patients undergoing CRT for a mean follow-up duration of 18 months. All patients were in sinus rhythm at the time of study enrollment, and 443 patients had documented AF (32%). AF episodes occurred in patients with (22%) and without (16%) a history of AF, lasting from 10 minutes to several weeks. Percent biventricular pacing was 95% in patients with AF vs 98% in the entire patient population. In patients with AF, percent biventricular pacing was 98% during sinus rhythm vs 71% during AF episodes (p < 0.001). Biventricular pacing <95% was defined as suboptimal and was associated with the occurrence of persistent or permanent AF (p < 0.001) and uncontrolled ventricular rate (p = 0.002) The percentage of biventricular pacing was inversely proportional to heart rate in patients with AF, decreasing by 7% for each 10-bpm increase in ventricular rate.

The importance of achieving high percentages of biventricular pacing was demonstrated in a large cohort of 36,395 patients included in the US LATITUDE Patient Management System who were remotely monitored.^202^Mortality was inversely proportional to the percentage of biventricular pacing both in sinus rhythm and AF or atrial pacing. The highest mortality reduction was observed in those with biventricular pacing < 98%. Patients with biventricular pacing > 99.6% had a mortality reduction of 24% (p < 0.001), whereas those with biventricular pacing < 94.8% had a mortality increase of 19%. Optimal percentage of biventricular pacing was set at ≥ 98.7%.

The multicenter, prospective Ablate and Pace in Atrial Fibrillation (APAF) study included 186 patients with symptomatic permanent AF, uncontrolled ventricular rate or refractory HF, LV systolic dysfunction, and a wide QRS complex.^203^All patients underwent AVJ ablation and multisite CRT implantation and were randomly assigned to receive echo-guided CRT (97 patients) or RV apical pacing (89 patients). During a median follow-up period of 20 months, CRT reduced the composite endpoint of death or hospitalization due to HF and worsening HF (11% in the CRT group vs 26% in the RV apical pacing group). CRT reduced hospitalization and worsening HF, while total mortality was similar in both groups.

AVJ ablation eliminates intrinsic conduction, resulting in 100% biventricular pacing in patients with CRT. This strategy was evaluated in a series of 673 patients (LVEF ≤ 35%, NYHA class ≥ II, QRS ≥ 120ms).^187^Among 162 patients with permanent AF in this cohort, 48 received rate control drugs and 114 underwent AVJ ablation. At 4-year follow-up, reverse remodeling and exercise tolerance were similar between patients with AF and those in sinus rhythm. In patients with AF, CRT benefited only those undergoing AVJ ablation. Despite > 85% biventricular pacing time, LV function and functional capacity were not improved in patients on rate control drugs.

In an observational study, Gasparini et al. reported that AVJ ablation combined with CRT significantly improved survival when compared with CRT alone.^204^Among 1,285 patients evaluated, 243 had AF. Rate control (85% biventricular pacing) was achieved by AVJ ablation in 188 patients and drug therapy in 55. At 34-month follow-up, mortality was significantly reduced in patients undergoing AVJ ablation (4.3% vs 15.2%, HR: 0.26 for overall mortality and 0.15 for HF mortality). These results suggest that CRT should achieve 100% biventricular pacing for maximum therapeutic benefit.

The Cardiac Resynchronization Therapy in Atrial Fibrillation Patients Multinational Registry (CERTIFY) corroborated the importance of AVJ ablation in patients with AF undergoing CRT. The study compared patients with permanent AF undergoing CRT combined with AVJ ablation or rate control drugs (n = 895) vs patients in sinus rhythm (n = 6,046). The results showed that, within 37 months, all-cause mortality (6.8 vs 6.1 per 100 person-years) and cardiovascular mortality (4.2 vs. 4.0) were similar in patients with AF plus AVJ ablation and patients in sinus rhythm. In contrast, patients with AF plus rate control drugs had higher total and cardiovascular mortality rates (11.3 and 8.1, respectively; p < 0.001) ( Table 15 ).

**Table 15:** – Recommendations for cardiac resynchronization therapy in patients with atrial fibrillation

	Class of recommendation	Level of evidence
CRT should be considered in patients with permanent AF, reduced LVEF (< 50%), and an indication for AVJ ablation for heart rate control	I	B
CRT should be considered in patients with permanent AF, reduced LVEF ≤ 35%, and NYHA functional class III and IV heart failure despite optimal drug therapy, LBBB, and QRS ≥ 130ms, with a strategy that allows > 95% biventricular pacing	IIa	B
AVJ ablation should be considered if biventricular pacing < 95%	IIa	B

*AVJ: atrioventricular junction; CRT: cardiac resynchronization therapy; LVEF: left ventricular ejection fraction; NYHA: New York Heart Association.*

### 3.3. Conventional PM Upgrade

Patients undergoing permanent RV pacing with a conventional PM or an ICD may develop progressive LV systolic dysfunction due to mechanical and electrical dyssynchrony. Registry data indicate that PM-induced LV dysfunction may occur in 12 to 30% of patients.^205 , 206^This condition is diagnosed by the presence of a high ventricular pacing rate and no other cause of LV dysfunction, such as untreated myocardial ischemia, valvular heart disease, or arrhythmias.

Nonrandomized studies have suggested that CRT may reverse PM-induced dysfunction.^207 - 209^LVEF recovery or improvement occurred in up to 86% of patients in those studies. Thus, patients with a PM or an ICD with high percent ventricular pacing showing worsening clinical and/or echocardiographic status may be candidates for CRT upgrade ( Table 16 ).

**Table 16:** – Recommendations for cardiac resynchronization therapy upgrade in patients with a conventional pacemaker

	Class of recommendation	Level of evidence
CRT is recommended in patients with a high percentage of right ventricular pacing (> 40%) showing worsening clinical and/or echocardiographic status despite optimal treatment, provided that other causes of left ventricular dysfunction are ruled out (LVEF ≤ 35%).	IIa	B

*CRT: cardiac resynchronization therapy.*

### 3.4. Indication for Antibradycardia Pacing (First Implant)

Possible deleterious effects of ventricular pacing with a conventional MP may justify the indication of CRT as an alternative treatment of bradycardias.

Studies have suggested that percent ventricular pacing > 40%, or even > 20%, is more likely to result in PM-induced LV dysfunction (12 to 30%). Pre-implant LVEF and QRS duration were additional predictors of PM-induced LV dysfunction.

Randomized clinical trials have evaluated whether CRT is superior to conventional RV pacing in reducing the occurrence of LV remodeling and improving clinical outcomes in patients with LVEF > 35%.^210 , 211^A meta-analysis of those studies compared conventional RV pacing vs biventricular or His bundle pacing.^212^Biventricular pacing compared with RV pacing was associated with increased LVEF and reduced end-diastolic and end-systolic LV volumes (patients with LVEF between 36% and 52% are more likely to benefit). Only 1 study reported significant improvement in the 6-minute walk distance test, which included patients with permanent AF who underwent AV node ablation (patients with LVEF < 45% and NYHA functional class II/III HF had better results). Data on His bundle pacing are summarized elsewhere in this guideline.

The meta-analysis excluded some important studies because they included patients with LVEF < 35%. The Biventricular versus Right Ventricular Pacing in Heart Failure Patients with Atrioventricular Block (BLOCK HF) study enrolled patients with indication for PM implantation due to AV block, NYHA functional class I-III HF, and LVEF ≤ 50%,^56^and they were randomly assigned to receive conventional RV pacing or biventricular pacing. Mean LVEF was 43%, and 87% of patients had LVEF > 35%. The primary composite outcome of death from any cause, urgent health care visit for HF requiring intravenous diuretic, and an increase ≥ 15% in LV end-systolic was more common in the RV pacing group. The APAF study evaluated patients with permanent AF undergoing AV junction (AVJ) ablation and CTR implantation who were randomly assigned to biventricular pacing or RV pacing.^204^The composite primary outcome of death from HF, hospitalization, or worsening HF occurred in 26% of patients in the RV group and 11% in the CRT group. Biventricular pacing was beneficial regardless of LVEF and QRS duration. Mean LVEF was 38%, and 53% of patients had LVEF > 35%.

COMBAT^55^was a prospective, multicenter, randomized, double-blind, crossover study that included 60 Brazilian patients with NYHA functional class II-IV HF, LVEF < 40%, and AV block as an indication for pacing. All patients underwent biventricular system implantation and were crossed over to conventional RV pacing after every 3 months (group A: RVP-BiVP-RVP and group B: BiVP-RVP-BiVP). Patients were evaluated at the end of each 3-month period. There were significant improvements in quality of life, functional class, and LV end-systolic volume compared with RV pacing. Also, the mortality rate was higher in the RV pacing group.

Recommendations for CRT in patients with a conventional PM indication for the treatment of bradyarrhythmias are summarized in Table 17 .

**Table 17:** – Recommendations for cardiac resynchronization therapy in patients with a conventional pacemaker indication

	Class of recommendation	Level of evidence
CRT is recommended in patients with atrioventricular block, a permanent pacemaker indication, and left ventricular systolic dysfunction (LVEF < 40%).	I	A

*CRT: cardiac resynchronization therapy; LVEF: left ventricular ejection fraction.*

### 3.5. Indication for Implantable Cardioverter-defibrillator Combined with Cardiac Resynchronization Therapy

ICD implantation is indicated in patients with different clinical conditions for primary or secondary prevention of sudden death (see item 4). Many of these patients also have LV dysfunction and LBBB and meet the criteria for CRT indication (see item 3). Thus, there are patients that may benefit from both therapies (CRT-D).

Randomized clinical trials have evaluated the effects of ICD and CRT-D implantation on patients with LV dysfunction and IVCD. All patients participating in the CONTAK CD, Multicenter InSync ICD Randomized Clinical Evaluation (MIRACLE ICD),^213^MIRACLE ICD II, and Cardiac Resynchronization Therapy in Patients with Heart Failure and Narrow QRS (RethinQ) studies^214^underwent CRT-D implantation. The groups were active CRT vs no pacing.

The CONTAK CD study evaluated 490 patients with a standard indication for ICD implantation, LVEF ≤ 35%, NYHA functional class II-IV HF, and QRS ≥ 120ms. There was no significant difference in the composite primary outcome of death from any cause, HF hospitalization, and ventricular arrhythmia requiring ICD intervention. CRT-D significantly improved oxygen uptake, 6-minute hall walk distance, and LVEF. The MIRACLE ICD study included 369 patients who also had a standard indication for ICD implantation, LVEF ≤ 35%, NYHA functional class III-IV HF, and QRS ≥ 130ms. CRT-D was associated with improved quality of life and oxygen uptake. The MIRACLE ICD II study included 186 patients with a standard indication for ICD, ejection fraction (EF) ≤ 35%, NYHA functional class II HF, and QRS ≥130ms. CRT-D was associated with reduced LV volumes and improved LVEF and NYHA functional class. The RethinQ enrolled 172 patients with an indication for ICD, EF ≤ 35%, NYHA functional class III HF, QRS ≤ 130 ms, and echocardiographic evidence of dyssynchrony. CRT-D did not improve the endpoint of peak oxygen consumption.

In the MADIT CRT^165^and RAFT studies,^167^patients were assigned to CRT-D or ICD alone. The MADIT CRT evaluated 1,820 patients with LVEF ≤ 30%, NYHA functional class I-II HF, and QRS ≥ 130ms. The composite endpoint of death from any cause or a nonfatal HF event was significantly reduced with CRT-D. The RAFT study included 1,798 patients with LVEF ≤ 30%, NYHA functional class II-III HF, and QRS ≥120 ms or paced QRS ≥200 ms. CRT-D significantly reduced the combined outcome of death from any cause or HF hospitalization. Mortality and HF hospitalization were analyzed separately as secondary outcomes and were significantly reduced.

A pooled analysis of the trial results showed that CRT-D reduced total mortality compared with ICD alone (RR 0.84; 95% CI 0.73-0.96).^215^There was also a significant reduction in hospitalizations (RR 0.75; 95% CI 0.64-0.88).

The clinical decision on whether to implant a CRT-D device in patients with an indication for ICD should consider the pattern of IV block and the QRS complex duration. Results from meta-analyses suggest that the benefits of CRT are mostly restricted to patients with an LBBB pattern.^216 , 217^The RAFT study^167^reported that CRT-D only reduced the primary outcome of death

from any cause or HF hospitalization in patients with QRS > 150ms. In patients with an LBBB pattern, there was a continuous relationship between QRS duration and clinical benefit, whereas patients with a non-LBBB pattern only benefited when QRS > 160ms ( Table 18 ).

**Table 18:** – Recommendations for cardiac resynchronization therapy in patients with an implantable cardioverter-defibrillator indication

	Class of recommendation	Level of evidence
CRT in patients with an ICD indication, LBBB, QRS ≥ 150ms, reduced LVEF, and symptomatic HF while on optimal drug therapy	I	A
CRT in patients with atrioventricular block, ICD indication, and left ventricular systolic dysfunction	IIa	B

*CRT: cardiac resynchronization therapy; HF: heart failure; ICD: implantable cardioverter-defibrillator; LBBB: left bundle branch block; LVEF: left ventricular ejection fraction.*

### 3.6. Direct Stimulation of the Cardiac Conduction System

CRT is a well-established nonpharmacological treatment for patients with symptomatic HF, reduced LVEF, and wide QRS complex. Although this therapeutic modality has advanced, 20 to 40% of patients still do not respond to CRT.^218^In this setting, direct stimulation of the cardiac conduction system (His bundle or LBB pacing) may be a useful alternative.^219 - 225^

Barba-Pichardo et al. described in 2012 a series of 16 patients with severe HF in whom LV pacing via the coronary sinus was not achievable. Direct His-bundle pacing was able to correct the conduction disorder (LBBB) in 81% of cases.^226^In another study, Lustgarten et al. reported on 29 patients who were randomly assigned to His bundle pacing or conventional CRT. Six-minute hall walk distance, NYHA functional class, quality-of-life score, and LVEF were similar between groups.^227^

A meta-analysis of 11 studies including 494 patients undergoing His bundle pacing reported a successful implantation rate of 82.4%. The studies evaluated patients with AF undergoing AV node ablation and patients with CRT indication. His bundle pacing had promising results in small, observational studies, which suggests the need for randomized trials.^228^

A recent randomized trial compared His bundle pacing vs standard biventricular pacing in 41 patients. Of 21 patients randomly assigned to His bundle pacing, 10 (48%) had to cross over to biventricular pacing. The main reason for therapy crossover was that His bundle pacing failed to correct the conduction disorder. This finding is in line with those of subsequent studies that reported bundle branch block correction with His bundle pacing in approximately 60% of cases. Nevertheless, 26% of patients in the conventional CRT group crossed over to His bundle pacing due to technical difficulties. The His bundle pacing group had greater QRS narrowing (174±18 ms to 125 ± 22ms, p < 0.001 vs 165 ± 17 ms to 164 ± 30 ms, p = 0.82) and greater improvement of echocardiographic parameters compared with the CRT group (80% of patients with His bundle pacing had an absolute LVEF improvement > 5%). Measurements of cardiac diameters and volumes were similar in both groups. These findings suggest that CRT and His bundle pacing may be complementary therapies considering the anatomical challenges of the coronary sinus and the failure to correct the disorder in all patients with LBBB.^229^

Zhang et al. described a series of 11 consecutive patients with LBBB and a conventional indication for CRT undergoing direct LBB pacing. QRS complex was significantly shortened (129.09 ± 15.94 ms compared with native QRS 180.00 ± 15.86 ms, p < 0.01). Deep septal pacing was found to be feasible in patients with systolic dysfunction and LBBB, resulting in functional improvement and reverse remodeling.^230 , 231^In a prospective cohort of 63 patients with nonischemic cardiomyopathy, LBBB, and LVEF < 35% undergoing LBB pacing, Huang et al. reported that LBB pacing was successful in 61 patients. Significant LVEF increase, reverse remodeling, and improved functional class were found at 1-year follow-up. Furthermore, a hyperresponse with normalized LVEF was observed in 75% of patients.^232^

Wu et al. conducted a nonrandomized study of 137 patients with symptomatic HF and wide QRS complex who were followed up for 1 year. Forty-nine patients underwent His bundle pacing, 32 underwent LBB pacing, and 54 patients underwent biventricular pacing. Conduction system pacing (His bundle or LBB) showed greater LVEF improvement compared with biventricular pacing (23.9%, 24%, and 16.7%, respectively, p < 0.05). Functional improvement was similar in both groups of conduction system pacing; however, LBB pacing was associated with lower pacing thresholds (0.49 vs. 1.35, p < 0.001).^233^

A recent review addressing direct stimulation of the cardiac conduction system suggests that His bundle pacing may be used as a primary strategy to achieve CRT or as a rescue strategy in patients in whom the standard technique (via coronary sinus catheterization) is unsuccessful. The combination of both methods (simultaneous His-CRT pacing) is considered a new concept of pacing for CRT in selected patients with more disseminated IVCDs^234^( Table 19 ).

**Table 19:** – Recommendations for direct stimulation of the cardiac conduction system as an alternative to cardiac resynchronization therapy

	Class of recommendation	Level of evidence
Physiological pacing is reasonable in patients with AV block, a pacemaker indication, and LVEF < 50%	IIa	B
Direct stimulation of the cardiac conduction system may be considered an alternative to conventional CRT in patients with symptomatic HF, LVEF ≤ 35%, and QRS ≥ 130ms	IIa	C
Direct stimulation of the cardiac conduction system may be considered as a rescue therapy in patients who do not respond to CRT	IIb	C

*AV: atrioventricular; CRT: cardiac resynchronization therapy; HF: heart failure; LVEF: left ventricular ejection fraction.*

#### 3.6.1. Cardiac Contractility Modulation

Cardiac contractility modulation (CCM) is a therapeutic option for HF in patients with no conventional indication for CRT, narrow QRS complex (< 130ms), and LVEF 25-45%. CCM promotes high-voltage (30 to 50ms) stimulation of the right interventricular septum after myocyte activation during the absolute refractory period. This type of stimulation theoretically optimizes calcium dynamics, resulting in increased ventricular contractility and improved exercise tolerance and functional capacity.^235^However, it remains unclear whether CCM should be routinely indicated in patients who are unfit candidates (narrow QRS) or who do not respond to CRT. It also remains unclear whether CCM should be used in combination with conventional CRTs.^236^The available studies are small and, perhaps because of that, were unable to detect significant differences.^237^

## 4. Recommendations for Placement of Implantable Cardioverter-defibrillators

### 4.1. Primary Prevention of Sudden Death

#### 4.1.1. Ischemic Cardiomyopathy

Sudden death from ventricular arrhythmias is one of the main causes of death in patients with HFrEF, especially in those with ischemic heart disease in whom the incidence of ventricular myocardial fibrosis and, consequently, reentry circuits is more prevalent.^238 , 239^

Importantly, studies evaluating the impact of ICDs on ischemic heart disease have broadly defined this condition as ventricular dysfunction secondary to at least 1 severe lesion in 1 of the 3 main coronary arteries or a previous history of documented AMI.^240 - 242^

Several important studies have evaluated the impact of ICDs on primary prophylaxis of sudden death in patients with ischemic heart disease. The MADIT^243^and MUSTT^244^studies tested ICD placement in patients with nonsustained ventricular tachycardia (NSVT), LV dysfunction, and inducible sustained ventricular arrhythmias (SVA) on EPS. The MADIT study enrolled 196 patients with LVEF < 35%, NYHA functional class I-III HF, a previous history of AMI, recorded asymptomatic NSVTs, and inducible SVAs on EPS refractory to procainamide or an equivalent antiarrhythmic drug. The patients were randomly assigned to ICD implantation vs optimized medical treatment (OMT) for a mean follow-up period of 27 months. The mortality rate was 15.7% in the ICD group and 38.6% in the OMT group, with a 64% relative risk reduction in total mortality in the ICD group (HR 0.46; 95% CI 0.26-0.82; p = 0.009). The MUSTT study evaluated patients with EF ≤ 40%, NYHA functional class I-III HF, and recorded asymptomatic NSVTs. The initial objective of the study was to compare the efficacy of antiarrhythmic medications capable of suppressing ventricular arrhythmias on EPS vs placebo. As a consequence of the MADIT results, the protocol was modified to ICD implantation in patients with inducible ventricular arrhythmias and reverse failure with at least one antiarrhythmic drug. Patients treated with antiarrhythmic medications did not show a significant decrease in mortality, whereas patients treated with an ICD had a 76% relative risk reduction in mortality (HR 0.24; 95% CI 0.13-0.45; p < 0.001).

Although the EPS is not normally used for ICD indication because of the low negative predictive value, the MADIT and MUSTT trials showed important results in this population (NNT for total mortality reduction = 4.2).

The MADIT II study randomly assigned 1,232 patients with EF ≤ 30%, NYHA functional class I-III HF, and AMI for more than 30 days to ODT vs ICD. During a mean follow-up period of 20 months, the mortality rate was 19% in the ICD group and 24% in the ODT group, with a 31% relative risk reduction in overall mortality (HR 0.69; 95% CI 0.51-0.93; p = 0.016).^245^The Sudden Cardiac Death in Heart Failure Trial (SCD-HeFT) expanded the inclusion criteria and enrolled patients with ischemic and nonischemic heart disease, LVEF ≤ 35%, and NYHA functional class II and III HF. During a mean follow-up period of 45.5 months, there was a 23% relative risk reduction in mortality in the ICD group (HR 0.77; 95% CI 0.62-0.96; p = 0.007), in which 52% of patients had ischemic heart disease.^246^

Conversely, studies evaluating early ICD implantation after revascularization or myocardial ischemic events found a neutral or even negative effect on some secondary outcomes. The Coronary Artery Bypass Graft (CABG) Patch trial randomly assigned 900 patients aged < 80 years with EF < 36% and signal-averaged ECG (SAECG) abnormalities to prophylactic ICD implantation at the time of bypass surgery. During a mean follow-up period of 32 ± 16 months, the results regarding overall mortality were neutral (6.7% in the ICD group and 4.6% in the control group, HR 1.07; 95% CI 0.81-1.42; p = 0.64).^247^A subsequent analysis showed an increased rate of infection in the ICD group (2.2% vs 0.4%; p < 0.05).^246^The Defibrillator after Acute Myocardial Infarction (DINAMIT) trial randomly assigned 332 patients to ICD therapy and 342 to no ICD therapy 6 to 40 days after AMI.^243^Inclusion criteria were patients with LVEF ≤ 35% and depressed heart-rate variability on Holter monitoring. In a mean follow-up duration of 30 ± 13 months, total mortality did not differ between groups, with 62 deaths in the ICD group and 58 deaths in the control group (HR 1.08; 95% CI 0.76-1.55; p = 0.66).

Since the publication of the Metoprolol CR/XL Randomized Intervention Trial in Congestive Heart Failure (MERIT-HF), the rate of sudden death has been known to decrease proportionally to worsening HF (NYHA class).^248^In a subgroup analysis of the SCD-HeFT and MADIT II trials, patients with NYHA functional class I and II HF benefited the most from ICD therapy, whereas patients with NYHA functional class III HF did not benefit significantly. There are no robust clinical trials demonstrating the benefit of ICD placement in patients with NYHA functional class IV HF, only data from retrospective cohorts of patients waiting for a transplant or undergoing ventricular assist device (VAD) implantation. A retrospective study including 1,089 patients on the heart transplant waiting list revealed that 550 patients had an ICD implant (216 for primary prevention and 334 for secondary prevention). In a mean follow-up duration of only 8 months, 39 patients (18%) in the primary prophylaxis ICD group, 89 (27%) in the secondary prophylaxis ICD group, and 162 (30%) in the no ICD group died. In multivariate analysis, the presence of an ICD was an independent predictor of reduced mortality (HR 0.4; 95% CI 0.19-0.85; p = 0.016).^248^The same finding was reported by a study that identified patients listed for heart transplantation between 1999 and 2014 in the United Network for Organ Sharing (UNOS) registry. Data from 32,599 patients were analyzed, with a mean follow-up duration of 154 days. A total of 3,638 patients (11%) died while waiting for a heart transplant, with a mortality rate of 9% in the ICD group and 15% in the no ICD group and a relative risk reduction of 13% (HR 0.87; 95% CI 0.80-0.94 p < 0.0001).^249^In the subgroup of patients undergoing VAD implantation (9,478 patients), the presence of an ICD was associated with a 19% relative risk reduction in mortality (HR 0.81; 95% CI 0.70-0 .94). A systematic review analyzed the use of ICDs in 937 patients with VADs (in 93% of the cases, the VAD was used as a bridge to transplantation). During a mean follow-up period of 7 months, 16% of patients in the ICD group and 26% in the no ICD group died, with a 39% relative risk reduction in mortality (HR 0.61; 95% CI 0.46-0.82; p < 0.01)^250^( Table 20 ).

**Table 20:** – Recommendations for placement of implantable cardioverter-defibrillators for primary prevention of ischemic heart disease

	Class of recommendation	Level of evidence
An ICD is recommended in patients with a history of AMI > 40 days or chronic ischemic heart disease receiving optimal drug therapy, no myocardial ischemia that could be treated by surgical or percutaneous revascularization, a life expectancy of at least 1 year, LVEF ≤ 35%, and NYHA functional class II-III HF (or LVEF ≤ 30% and NYHA functional class I-III HF)	I	A
An ICD is recommended in patients with a history of AMI > 40 days or chronic ischemic heart disease undergoing optimal drug therapy, no myocardial ischemia that can be treated by surgical or percutaneous revascularization, a life expectancy of at least 1 year, LVEF ≤ 40%, spontaneous NSVT, and inducible sustained ventricular arrhythmias on EPS	I	B
An ICD may be considered in patients with a history of AMI > 40 days or chronic ischemic heart disease receiving optimal drug therapy, no myocardial ischemia that could be treated by surgical or percutaneous revascularization, and who are candidates for heart transplantation or VAD implantation	IIa	B
ICD is not indicated in patients with AMI < 40 days or an indication for revascularization	III	B
ICD is not indicated in patients with NYHA functional class IV HFrEF refractory to treatment and who are not candidates for heart transplantation or VAD implantation	III	C

*AMI: acute myocardial infarction; EPS: electrophysiology study; ICD: implantable cardioverter-defibrillator; LVEF: left ventricular ejection fraction; NSVT: nonsustained ventricular tachycardia; NYHA: New York Heart Association; SVT: sustained ventricular tachycardia; VAD: ventricular assist device.*

#### 4.1.2. Nonischemic Cardiomyopathy

HF is a highly prevalent clinical condition with significant morbidity and mortality. HF etiology is defined as nonischemic in 20% to 30% of cases, which means that no significant lesions are seen on coronary angiography or that the imaging test result for ischemia is negative. LV dysfunction may result from unknown causes (idiopathic dilated cardiomyopathy) or viral infections, hypertension, exposure to potentially toxic agents (chemotherapeutic drugs, alcohol), Chagas disease, infiltrative diseases, peripartum cardiomyopathy, valvular heart disease, or genetic and autoimmune diseases.

Although advances in the treatment of nonischemic cardiomyopathy (NICM) have significantly reduced mortality in recent decades, sudden cardiac death (SCD) remains a major problem that accounts for 30% of deaths.^251^Primary prevention strategies for SCD in patients with NICM include pharmacological treatment, ICD implantation, and CRT. Randomized clinical trials have shown that drug therapy (beta-blockers, sacubitril/valsartan, and spironolactone) significantly reduces the rates of SCD in this group of patients.^252^

Risk stratification should include clinical and laboratory assessment. The worse the NYHA functional class, the greater the absolute risk of overall mortality and SCD. SCD accounts for 64%, 50%, and 33% of deaths in patients with NYHA functional class II, III, and IV HF, respectively (HF progression accounts for 50% of deaths in NYHA class IV patients). Syncope is an important risk factor for SCD in patients with NICM.^253^Other clinical variables associated with a higher risk of arrhythmic events in this population include not using beta-blockers and SBP.^254 , 255^Laboratory tests, such as hemoglobin, uric acid, and atrial natriuretic peptide (brain natriuretic peptide [BNP]), were predictors of mortality and arrhythmic events in some studies.^256^

LVEF reduction is considered the main risk factor for SCD and total mortality in patients with HF. However, few studies have evaluated LVEF as a risk factor for SCD in patients with NICM. The Marburg Cardiomyopathy Study (MACAS), a prospective cohort of 343 patients with NICM, revealed that for every 10% reduction in LVEF, there was a relative risk of 2.28 for major arrhythmic events (patients in sinus rhythm).

The prevalence of wide QRS complexes among patients with HF ranges from 20% to 50% and is associated with increased SCD and total mortality rates; however, in cohort studies of patients with NICM, there was no significant relationship between QRS prolongation and increased risk of SCD.^257 , 258^

Holter monitoring may be useful for risk assessment as it allows investigating the presence of NSVT and measuring autonomic activities (heart rate variability and heart rate turbulence). The incidence of NSVT among patients with NICM ranges from 30% to 79%, but its use in the risk stratification of arrhythmic events is controversial.^259^

A meta-analysis has suggested that cardiopulmonary exercise testing variables such as oxygen uptake (VO_2_), minute ventilation/carbon dioxide production (VE/VCO_2_) slope, and presence of periodic breathing are independently associated with an increased risk of combined events. Combinations include total mortality, cardiac mortality, heart transplantation, hospitalization, and need for VAD.^260 , 261^

In cases of ischemic heart disease, performing an EPS with programmed ventricular pacing has been shown to identify patients at risk of serious arrhythmic events.^246 , 248^Conversely, EPS results are controversial in cases of NICM, and guidelines do not usually recommend routine EPS for risk stratification in this patient population.

Several studies have evaluated the association between genetic mutations and the pathophysiology and prognosis of NICM, particularly in patients with familial disease.^262^Mutations in the lamin A/C ( *LMNA* ) gene are among the most studied conditions. Such mutations are found in 6% to 8% of NICM cases and may reach up to 30% when combined with conduction system diseases and skeletal muscle involvement.

According to a recent meta-analysis of 34 studies including 4,554 patients, approximately 44% of patients with NICM have myocardial fibrosis (an important arrhythmogenic substrate).^263^These patients show higher rates of mortality, ventricular arrhythmias, and HF hospitalizations.^264^For each percent increase in late gadolinium enhancement (LGE) volume, the risk of mortality or arrhythmic events is estimated to increase by 3% to 20% (8% LGE; HR in univariate analysis 8.23, 95% CI 2.84-23.8). In the largest cohort of patients with NICM undergoing CABG surgery,^265^Gulati et al. reported that the combined endpoint of SCD and aborted SCD occurred in 29.6% of patients with myocardial fibrosis and in 7.0% of patients without fibrosis. The HR for presence of fibrosis was 4.61 (95% CI 2.75-7.74; p < 0.001), and the HR for extent of fibrosis was 1.10 (95% CI 1.05-1.16; p < 0.001). These results suggest that MRI may be useful for the risk stratification of patients with NICM.

Several studies have evaluated the impact of ICDs on patients with NICM. The SCD-HeFT^245^is the largest study and included patients with ischemic and nonischemic heart disease and NYHA functional class II-III HF. Among patients implanted with an ICD, 33.2% received at least one shock, with 22.4% receiving only appropriate shocks and 10.7% receiving only inappropriate shocks.^266^In the DANISH study, a randomized trial of patients with NICM whose primary endpoint was all-cause mortality, 556 patients received ICD therapy and 560 received only ODT.^267^In a median follow-up duration of 67.6 months, the primary outcome occurred in 21.6% of the ICD group and in 23.4% of the control group, with no significant differences (p = 0.28).

The current recommendations for ICD implantation in patients with NICM are listed in Table 21 .

**Table 21:** – Recommendations for placement of implantable cardioverter-defibrillators for primary prevention of nonischemic heart disease

	Class of recommendation	Level of evidence
Patients on optimal drug therapy with LVEF ≤ 35%, NYHA functional class II-III HF, and a life expectancy > 1 year	I	B
An ICD should be considered in the presence of high-risk genetic abnormalities (especially lamin A/C) combined with two or more of the following factors: LVEF ≤ 45%, NSVT, high-risk mutations, and male sex	IIa	B
Refractory NYHA functional class IV heart failure with no chance for heart transplantation or CAD	III	C

*CAD: coronary artery disease; ICD: implantable cardioverter-defibrillator; LVEF: left ventricular ejection fraction; NSVT: nonsustained ventricular tachycardia; NYHA: New York Heart Association.*

#### 4.1.3. Hypertrophic Cardiomyopathy

Hypertrophic cardiomyopathy (HCM) is a genetic disease caused by an autosomal dominant mutation in genes encoding sarcomere proteins, with a prevalence of approximately 1:500.^268 , 269^HCM is characterized by the presence of varying degrees of asymmetric LV hypertrophy, provided that there are no other conditions that may explain this abnormality. It may result in diastolic HF, LVOT obstruction, atrial and ventricular arrhythmias, and, in some cases, SCD.^270 , 271^Most patients have no symptoms, with SCD often being the first manifestation of the disease.^272 - 276^

In 1958, Teare reported on a series of eight patients with asymmetric myocardial hypertrophy (the nomenclature was not consolidated at the time) or hamartoma and correlated the anatomical findings with a higher occurrence of SCD in young adults. Pathology findings consisted of a coarse disarray of the muscle bundles with hypertrophy of the individual muscle fibers and their nuclei.^277^

The annual risk of SCD in patients with an HCM diagnosis is approximately 1%; however, some patients may be at higher risk because of certain characteristics.^278^Before the ICD was introduced, the mortality rate was approximately 1.5% per year; after its introduction, the mortality rate has decreased to 0.5% per year.^279 , 280^

Maron et al. conducted a longitudinal, single-center study including a large cohort of 2,094 patients with HCM followed up for 17 years. Of 527 patients with a primary prevention ICD implanted based on at least one conventional risk factor, 15.6% experienced appropriate ICD interventions (ventricular tachycardia [VT]/ventricular fibrillation [VF]), corresponding to almost 50 times the number of events in the non-ICD group.^281^

HCM is the leading cause of SCD in patients aged < 40 years, mostly resulting from VF. Thus, ICD implantation is the most effective strategy to reduce mortality in high-risk patients despite higher costs and the possibility of complications, discomfort, and psychological stress.^282 , 283^Individual characteristics, clinical manifestations, family history, and definitions of risk factors may hinder patient selection, in addition to the fact that SCD is uncommon in clinical practice.^284 , 285^Patients who are more likely to benefit from ICDs are identified through noninvasive tests such as clinical history, ECG, stress test, Holter monitoring, echocardiography, and cardiac magnetic resonance (CMR). Conventional risk factors for SCD include a family history of HCM-related SCD, unexplained syncope occurring within 6 months of evaluation, NSVT, septal thickness ≥ 30mm, and risk modifiers such as a hypotensive response during stress test, LV fibrosis, and HF with LVEF < 50%.^286^Risk stratification for an ICD indication in patients with HCM should be performed periodically, every 1 or 2 years.^287^

An ICD indication for HCM is not based on randomized clinical trials but rather on observational studies. Studies of patients with HCM and implanted ICDs reported that life-threatening events with appropriate ICD therapy occur at rates of 12% per year in secondary prevention and 4% per year in primary prevention.^288^In this setting, the likelihood of experiencing appropriate ICD therapies seems to be similar among patients with 1, 2, 3, or more conventional risk factors (primary prevention), which suggests that the presence of a single marker may justify ICD implantation. Among conventional risk factors, a family history of SCD likely or definitely due to HCM in first-degree relatives aged ≤ 50 years, especially during childhood or adolescence, is highly significant.^289^Another risk marker for SCD is the extent and magnitude of hypertrophy, especially when wall thickness ≥ 30mm; borderline thickening (28 to 29 mm) may be considered at the cardiologist’s discretion. Spirito et al. evaluated 480 patients and reported that SCD incidence was almost twice as high for each 5-mm increase in ventricular myocardial thickness, reaching 1.8% per year in those with wall thickness ≥ 30mm.^290^

The presence of unexplained syncope (when syncopal episodes are unrelated to LVOT obstruction or when vasovagal syncope is unlikely or has been ruled out) has been strongly associated with SCD risk in patients with HCM, especially if occurring within 6 months of initial evaluation. These patients had a 5-fold higher risk compared with those without syncope. Remote episodes of syncope (> 5 years before initial evaluation) did not correlate with an increased risk of SCD.^291^

NSVT is defined by the presence of 3 or more episodes with 3 or more repetitive ventricular beats and/or 1 or more prolonged episode with 10 or more beats at a rate of ≥ 130bpm on 24-hour or 48-hour Holter monitoring. Reported NSVT incidence in patients with HCM has ranged from 20% to 46%. In patients with HCM, VT episodes are undoubtedly associated with SCD; however, data are less robust for demonstrating that the presence of NSVT alone is an independent risk factor. Conversely, the risk increases in the presence of risk modifiers, especially LV fibrosis.^292 , 293^

Genetic counseling is important in patients with HCM. Identifying carriers of specific genetic mutations may help investigating the disease in close relatives. Because patients with positive gene tests are likely to develop HCM, they should be closely monitored for symptoms and risk factors over time.^294^

There is growing evidence of the relationship between myocardial fibrosis on CMR and SCD risk, which is considered a risk modifier.^295 - 297^A Brazilian study of high-risk patients with HCM and implanted ICDs found myocardial fibrosis in 96.4%, with a mean fibrosis rate of 15.96%. This suggests that fibrosis may be more sensitive than other conventional risk markers.^298^Chan et al. reported that LGE ≥ 15% of LV mass was associated with a 2-fold higher risk of SCD in patients otherwise considered low risk.^299^

Klopotowski et al. prospectively analyzed 328 patients with HCM undergoing CMR to evaluate whether LGE location could be used as an auxiliary tool in the risk stratification of SCD. LGE suggesting the presence of fibrosis outside the interventricular septal region in patients with HCM was associated with an increased risk of SCD or its equivalent, such as unstable VT or appropriate ICD therapy. Considering the risk calculator developed by the European Society of Cardiology (ESC), the presence of fibrosis outside the interventricular septal region in intermediate-risk patients may help identify those who are more likely to benefit from ICD and thus favor an ICD indication.^300 - 302^

Although the ESC has encouraged the use of the risk calculator in patients with HCM, the tool has a low sensitivity to determine whether an ICD should be implanted in high-risk patients. The American society strategy is to analyze risk factors alone or combined with risk modifiers in each patient with HCM. This strategy has a sensitivity of 95% for predicting potentially fatal VT events, being superior to the mathematical model of the ESC risk score, whose sensitivity is approximately 34%. Conversely, the ESC risk calculator is more sensitive for identifying patients who are truly low-risk (those with a lower likelihood of events), approximately 92% vs 78% of the American society strategy, and this avoids unnecessary ICD implantations.

Abnormal responses or exercise-induced hypotension affect 1 in 3 patients with HCM. The mechanism reflects an exacerbated fall in systemic vascular resistance due to an autonomic dysfunction and/or dynamic obstruction of the LVOT. In young patients, an abnormal blood pressure response is associated with an increased risk of SCD.^303^

The identification of a LV apical aneurysm on echocardiography or CMR regardless of size may be associated with an increased risk of sustained monomorphic VT.^304^Rowin et al. retrospectively evaluated 1,940 patients with HCM and found LV aneurysms in 93 of them (4.8%). The adverse event rate was 6.4% per year, which is 3 times higher than that observed in patients without aneurysms, and included SCD, appropriate ICD therapy, thromboembolic events, and end-stage HF with LVEF < 50%. Fifty-four patients were implanted with an ICD for primary prevention, including 19 in whom the aneurysm alone was considered a risk factor. Appropriate ICD interventions for VT/VF were experienced by 20% of patients. Patients with apical aneurysms had an arrhythmic event rate of almost 5% per year, over 5-fold higher than that in patients without aneurysms, suggesting an equivalence to other conventional risk markers in high-risk HCM populations.^305^

Subcutaneous ICDs are potentially advantageous, especially in young people, considering the device capability to preserve the venous system and avoid chronic lead complications (as long as ventricular pacing is not required). Conversely, the effectiveness of subcutaneous ICDs in aborting VF in patients with HCM remains uncertain.^306^

Finally, studies evaluating the role of EPS in the risk stratification of SCD in patients with HCM found no benefits. Therefore, EPS should not be indicated for this purpose^307^( Figure 2 and Table 22 ).

**Figure 2:** – Algorithm for primary prevention of SCD in patients with HCM. +FH: positive family history; HCM: hypertrophic cardiomyopathy; LV: left ventricular; LVEF: left ventricular ejection fraction; LVOT: left ventricular outflow tract; NSVT: nonsustained ventricular tachycardia; SCD: sudden cardiac death.

**Table 22:** – Recommendations for placement of implantable cardioverter-defibrillators for primary prevention of hypertrophic cardiomyopathy

	Class of Recommendation	Level of evidence
Patients with at least one major risk factor	IIa	B
Patients with NSVT or abnormal arterial hypotension during exercise in the presence of additional risk factors, if life expectancy > 1 year	IIa	B
Invasive risk stratification with EPS	III	B

*Risk factors: wall thickness > 30 mm, family history of sudden death, NSVT, late gadolinium enhancement on MRI, syncope < 5 years, LV aneurysm, and LVEF <50%. EPS: electrophysiology study; NSVT: nonsustained ventricular tachycardia.*

#### 4.1.4. Chagas Cardiomyopathy

Chagas disease is caused by the protozoan *Trypanosoma cruzi* , being mostly transmitted to humans through the feces of bloodsucking insects of the Triatominae family.^308^The infection generally occurs during childhood, and the acute phase has an incubation period of 1 to 2 weeks that can last up to 3 months. In the chronic phase, patients have no clinically apparent signs or symptoms of the disease, while their serological tests remain positive for 2 to 4 decades.^309 , 310^These patients have the indeterminate form of Chagas disease, whose prognosis is mostly favorable.^311 , 312^Although many patients, through mechanisms that are still not fully understood,^313^will carry the indeterminate form throughout life, approximately 30% to 50% of infected patients will develop one of the determinate forms (cardiac, digestive, or mixed).

Chronic Chagas cardiomyopathy (CCC) has very marked pathophysiological characteristics and is the most common and severe clinical form of the disease, with increased morbidity and mortality rates in Latin America and in countries with significant immigration.^314^

Eight to 10 million people are estimated to be infected with *Trypanosoma cruzi* in Latin American and other countries.^315 - 317^Based on previous estimates and considering the worst-case scenario, 3 to 5 million infected individuals are expected to manifest clinical forms of the disease in the chronic phase.

The estimated average annual mortality rate of CCC is 4% but may range from 1% to 10% according to risk stratifications based on clinical characteristics and simple cardiac tests.^318^

In addition to risk stratification criteria, several markers of poor prognosis have been identified by different studies, especially regarding SCD in different clinical settings.^319 - 338^Variables such as presyncope and syncope, LV dysfunction and HF, sustained ventricular tachycardia (SVT) or NSVT, severe bradyarrhythmia (SND and AV block), and recovered cardiac arrest were identified as risk markers for SCD. Conversely, ventricular extrasystoles alone on Holter monitoring and RBBB do not significantly affect the prognosis of CCC.

SCD accounts for approximately 55% to 65% of all death causes and is often associated with HF manifestations, although it may also occur in patients with asymptomatic LV dysfunction.^339 - 341^Refractory HF accounts for approximately 25% to 30% of deaths. The correlation between CCC stages and causes of death was recently described: SCD is more prevalent in stage III, whereas the prevalence of death from HF progressively increases from stage I to IV.

The main mechanism of sudden death in CCC is arrhythmogenic, and SVT with subsequent VF accounts for the vast majority of fatal events.^342^Thus, the structural abnormalities of CCC (with inflammation, cell death, and reactive and reparative fibrosis) represent the ideal anatomical substrate because they promote unidirectional blocks and slow conduction areas favorable to electrical reentry triggering. The triggers that affect this anatomical substrate, named ventricular extrasystoles, are invariably present and complete the key elements for the onset of reentry ventricular tachyarrhythmia.^314 , 329 , 331 , 333 , 336 , 338 , 345^Thus, NSVT may affect approximately 40% of patients with CCC and abnormal segmental mobility and practically all patients with global LV systolic dysfunction and HF.^343^SVT, which has a more ominous prognosis, occurs spontaneously and can be reproduced on EPS in approximately 80% to 85% of patients.^319 , 328 , 329^

Complete AV block, although less common, is another cause of SCD in CCC, resulting from necrotic degeneration and diffuse fibrosis predominantly in the AV region.^330^

As previously mentioned, SCD may also result from massive pulmonary thromboembolism or systemic thromboembolism in vital organs. Exceptionally, SCD may be due to a ruptured LV apical aneurysm.

Rassi et al. developed a risk score to predict death in patients with CCC based on clinical variables and routine cardiac tests.^322^The score was also used elsewhere^344^in a retrospective cohort of 149 patients. The hypothesis that the presence of VT on cardiac stress test or Holter monitoring, LVEF < 0.50, and QRS > 50ms on SAECG could identify patients with CCC at risk of death in 5 years was raised. Low-risk groups are characterized by the absence or presence of one risk factor, intermediate-risk groups by the presence of two risk factors, and high-risk groups by the presence of three risk factors.

Primary prevention of SCD in patients with CCC theoretically includes the use of amiodarone or ICD therapy.^345^However, there is no scientific evidence to support an ICD indication for primary prevention of SCD in CCC. There are many pathogenetic and pathophysiological characteristics that hinder any direct comparison with the results of studies evaluating other heart diseases.^317^The most significant characteristic is that many patients with CCC, even those with preserved LV function, already have substrates for potentially fatal arrhythmias^331 , 345 - 348^( Table 23 ).

**Table 23:** – Recommendations for placement of implantable cardioverter-defibrillators for primary prevention of chronic Chagas cardiomyopathy

	Class of recommendation	Level of evidence
Patients with stable SVT, LVEF < 35%, on optimal medical treatment	I	C
Patients with stable SVT, LVEF > 35%, on optimal medical treatment	IIa	C
Patients with NSVT, LVEF < 35%, on optimal medical treatment	IIb	C
Patients with refractory NYHA IV HF, not candidates for heart transplantation	III	C

*LVEF: left ventricular ejection fraction; NYHA: New York Heart Association; NSVT: nonsustained ventricular tachycardia; SVT: sustained ventricular tachycardia; HF: heart failure.*

The Chronic Use of Amiodarone against ICD Therapy in Chagas Cardiomyopathy for Primary Prevention of Death (CHAGASICS) study is an ongoing open-label, randomized, multicenter clinical trial whose aim is to compare the efficacy of ICD therapy vs amiodarone for the primary prevention of all-cause mortality in patients with CCC and NSVT stratified by the Rassi risk score.^322^The CHAGASICS trial is expected to provide the scientific evidence needed to define the criteria for amiodarone and/or ICD indication in patients with CCC without life-threatening clinical outcomes.

#### 4.1.5. Arrhythmogenic Right Ventricular Cardiomyopathy

Arrhythmogenic RV cardiomyopathy (ARVC) has an autosomal dominant mode of inheritance and variable penetrance, which causes mutations in genes encoding cell adhesion proteins, named desmosomes. ARVC predominantly affects the RV but may also affect the LV in approximately 0.5% of cases, resulting in myocardial tissue replacement by fibrosis and adipose tissue. Such structural changes often cause ventricular arrhythmias and SCD.^346 , 347^The estimated prevalence of ARVC ranges from 1:1,000 to 1:5,000 in the general population, representing a leading cause of SCD in athletes and young adults.^348^

Ventricular arrhythmias, syncope, and SCD occur particularly in the second and third decades of life, usually during a physical activity. Syncope is reported in 16% to 39% of patients with ARVC at the time of diagnosis, is often related to physical activity, and has been associated with an increased risk of arrhythmias.^349^

Frequent ventricular extrasystoles, NSVT, and SVT are important predictors of cardiac events, and SVT is an important predictor of SCD and appropriate ICD therapies. SCD may be the first manifestation of ARVC.^350 , 351^

Ventricular arrhythmias usually originate in the RV (LBBB morphology), but the QRS axis during SVT often differs from the RV outflow tract (RVOT). Many patients may have QRS complexes with different morphologies.^352^

Regions of fibrofatty tissue create areas of delayed ventricular activation, resulting in fractional deflections at the end of the QRS complex (epsilon waves) and late potentials on SAECG. In patients with suspected ARVC, performing an SAECG may be useful for diagnosis and risk stratification (class of recommendation: IIa; level of evidence: B). On CMR, SAECG abnormalities are related to ARVC severity and the occurrence of adverse events.^353^

CMR uses LGE to provide information regarding ventricular function, cardiac chamber size, segmental abnormalities, and extent of fibrosis. LGE has demonstrated biventricular and LV involvement alone in 34% to 56% and 4% to 9% of patients, respectively. LGE areas on CMR are related to the location of the ventricular arrythmia substrate, identified by endocardial and epicardial electrophysiological mapping.^354^

The value of EPS as a risk predictor of SCD in asymptomatic ARVC is uncertain (class of recommendation: IIb, level of evidence: B). In patients implanted with an ICD for primary prevention, inducible SVT is not a predictor of appropriate shocks.^355^The diagnostic criteria for ARVC are listed in Table 24 .^355^

**Table 24:** – Revised criteria for the diagnosis of arrhythmogenic right ventricular cardiomyopathy

I. Regional or global dysfunction and structural abnormalities	
Major criteria	Echocardiography
	RV segmental akinesia, dyskinesia, or aneurysm and one of the following (end-diastole):
	RVOT on PLAX view ≥ 32mm (≥ 19mm/m^2^corrected for BSA)
	RVOT on PSAX view ≥ 36mm (≥ 21mm/m^2^corrected for BSA)
	Change in fractional area ≤ 33%
	Cardiac magnetic resonance
	RV segmental akinesia, dyskinesia, or dyssynchronous RV contraction and one of the following:
	Ratio of RV end-diastolic volume to BSA ≥ 110 mL/m^2^(men) or ≥ 100 mL/m^2^(women)
	RV ejection fraction ≤ 40%
	RV angiography
	RV segmental akinesia, dyskinesia, or aneurysm
Minor criteria	Echocardiography
	RV segmental akinesia or dyskinesia and one of the following (end-diastole):
	RVOT on PLAX view ≥ 29 to < 32mm (≥ 16mm to < 19mm/m^2^corrected for BSA)
	RVOT on PSAX view ≥ 32 to < 36mm (≥ 18 to < 21mm/m^2^corrected for BSA)
	Change in fractional area > 33% to ≤ 40%
	CMR
	RV segmental akinesia, dyskinesia, or dyssynchronous RV contraction and one of the following:
	Ratio of RV end-diastolic volume to BSA ≥100 to <110 mL/m^2^(men) or ≥ 90 to < 100mL/m^2^(women)
	RV ejection fraction > 40% to ≤ 45%
**II. Wall tissue characterization**	
Major criteria	Less than 60% of residual myocytes by morphometric analysis (or < 50% if estimated), with RV free wall fibrous replacement in more than one sample, with or without fat replacement on endomyocardial biopsy
Minor criteria	60% to 75% of residual myocytes by morphometric analysis (or 50% to 65% if estimated), with RV free wall fibrous replacement in more than one sample, with or without fat replacement on endomyocardial biopsy
**III. Changes in repolarization**	
Major criteria	Inverted T waves in the precordial leads (V_1_, V_2_, and V_3_) or beyond in patients aged > 14 years (in the absence of complete RBBB QRS ≥ 20ms)
Minor criteria	Inverted T waves in leads V_1_and V_2_in patients aged > 14 years (in the absence of complete RBBB) or leads V_4_, V_5_e V_6_
	Inverted T waves in leads V_1_, V_2_, V_3_, and V_4_in patients aged > 14 years in the presence of complete RBBB
**IV. Repolarization/conduction abnormalities**	
Major criteria	Epsilon wave (low-amplitude signals between the end of the QRS complex and the onset of the T wave) in leads V_1_to V_3_
Minor criteria	Late potentials by ≥ 1 of the following 3 SAECG parameters in the absence of QRS duration ≥ 110ms on ECG
	Filtered QRS duration ≥ 114ms
	Terminal QRS duration < 40µV (low-amplitude signal duration) ≥38 ms
	Root-mean-square voltage of terminal 40 ms ≤ 20µV
	Terminal activation duration of QRS ≥ 55ms measured from the nadir of the S wave to the end of the QRS, including R’, in V_1_, V_2_, or V_3_in the absence of complete RBBB
**V. Arrhythmias**	
Major criteria	NSVT or SVT with LBBB morphology and superior axis (negative or indeterminate QRS in leads II, III, and aVF and positive QRS in lead aVL)
Minor criteria	NSVT or SVT of the RVOT, LBBB morphology, and inferior (positive QRS in leads II, III, and aVF and negative QRS in lead aVL) or unknown axis
	> 500 ventricular extrasystoles in 24 hours (Holter monitoring)
**VI. Family history**	
Major criteria	ARVC confirmed in a first-degree relative diagnosed according to these criteria
	ARVC confirmed during autopsy or surgery in a first-degree relative
	Identification of a pathogenic mutation categorized as associated or probably associated with ARVC
Minor criteria	History of ARVC in a first-degree relative in whom it is not possible to determine whether the diagnostic criteria were met
	Sudden death in a first-degree relative aged < 35 years with suspected ARVC
	ARVC pathologically confirmed by these criteria in a second-degree relative

*ARVC: arrhythmogenic right ventricular cardiomyopathy; BSA: body surface area; CMR: cardiac magnetic resonance; ECG: electrocardiography; LBBB: left bundle branch block; NSVT: nonsustained ventricular tachycardia; PLAX: parasternal long axis; PSAX: parasternal short axis; RBBB: right bundle branch block; RV: right ventricle; RVOT: right ventricular outflow tract; SAECG: signal-averaged electrocardiogram; SVT: sustained ventricular tachycardia.*

Genetic tests performed on probands with suspected ARVC are positive in 30% to 54% of cases. Importantly, a negative test does not rule out the disease, and a positive test does not define the course of treatment. ARVC is detected in approximately 35% to 40% of first-degree relatives, and clinical screening with ECG, Holter monitoring, stress test, and cardiac imaging may identify family members at risk for ARVC.^356^

Asymptomatic patients without ventricular arrhythmias should receive only beta-blockers and undergo periodical evaluations of ventricular function and arrhythmia.^357^Randomized trials evaluating the best option among antiarrhythmic drugs for the treatment of SVT are lacking. An observational study reported suppression of inducible VT with sotalol in 58% of patients; only 10% of patients had recurrent arrhythmias.^358^In another observational registry, beta-blockers and sotalol were not associated with a reduction in ventricular arrhythmias, whereas amiodarone was superior in preventing them in a small cohort.^359^Finally, SVT ablation reduces recurrent arrhythmias but does not eliminate the need for ICD implantation.^360^

Patients with a history of aborted SCD, poorly tolerated SVT, and syncope are at increased risk of SCD, with an annual rate > 10%. ICD implantation is indicated in these cases. Different cohorts have shown SVT, unexplained syncope, frequent NSVT, family history of early SCD, extensive RV involvement, very prolonged QRS complexes, LGE on CMR, LV dysfunction, and inducible SVT on EPS as risk factors for SCD or appropriate shock.^361^A recent systematic review including 610 patients followed up for a mean period of 3.8 years revealed annual rates of appropriate and inappropriate shocks of 9.5% and 3.7%, respectively.^362 , 363^

An ICD indication for primary prevention of ARVC is difficult to assess and should rely on detailed clinical evaluation, including family history, RV and LV dysfunction severity, long-term ICD complications, and psychological and economic impacts. ICD indications for primary prevention of ARVC are listed in Table 25 and Figure 3 .

**Table 25:** – Recommendations for placement of implantable cardioverter-defibrillators for primary prevention of arrhythmogenic right ventricular cardiomyopathy

	Class of recommendation	Level of evidence
ARVC and aborted SCD, or SVT if expected survival > 1 year	I	B
ARVC and major RV dysfunction, or LVEF ≤ 35% if expected survival > 1 year	I	B
In ARVC and syncope likely due to ventricular arrhythmia, an ICD may be useful if expected survival > 1 year	IIa	B
ARVC and well-tolerated SVT, provided that ICD benefits and long-term complications are evaluated	IIa	C

*ARVC: arrhythmogenic RV cardiomyopathy; ICD: implantable cardioverter-defibrillator; LVEF: left ventricular ejection fraction; RV: right ventricular; SCD: sudden cardiac death; SVT: sustained ventricular tachycardia.*

**Figure 3:** – Flow diagram for patient stratification and implantable cardioverter-defibrillator indication in arrhythmogenic right ventricular cardiomyopathy. ICD: implantable cardioverter-defibrillator; LV: left ventricle; NSVT: nonsustained ventricular tachycardia; RV: right ventricle; SCD: sudden cardiac death; SVT: sustained ventricular tachycardia.

A new model for predicting ventricular arrhythmias in ARVC was recently published.^364^Predictive variables included male sex, age, syncope in the last 6 months, previous NSVT, number of ventricular extrasystoles on 24-hour Holter monitoring, number of leads with inverted T wave in the inferior and anterior leads, and RV ejection fraction. This new model allowed greater refinement in patient selection for ICD implantation when compared with the 2015 International Task Force flowchart, reducing the rate of implant indication by 20.6%.^365^The authors of the new model have launched an online risk calculator (www.arvcrisk.com) that calculates the risk of ventricular arrhythmia in 5 years. Although it does not determine an acceptable risk threshold for ICD implantation, the model is believed to help in the decision-making process for primary prevention.

#### 4.1.6. Noncompaction Cardiomyopathy

Noncompaction cardiomyopathy (NCCM) is a rare congenital abnormality characterized by the formation of prominent trabeculations and deep intertrabecular recesses in the LV and RV. It occurs during the endomyocardial morphogenesis phase (between weeks 5 and 8 of fetal life), most commonly reaching the LV apex.^363^Both ventricles are involved in 22% to 38% of patients. Left ventricular noncompaction (LVNC) occurs alone or in combination with other congenital heart diseases.^366^

An autosomal dominant mode of inheritance is present in at least 30% to 50% of patients. Several genes that cause LVNC have already been identified, and they generally encode sarcomere (contractile apparatus) or cytoskeletal proteins.^367^

The clinical manifestations of NCCM are heterogeneous, ranging from completely asymptomatic cases to cases of severe and fatal manifestations such as HF, thromboembolism, AV and IV blocks, ventricular arrhythmia, and SCD. Predictors of higher mortality include age, LV end-diastolic diameter, symptomatic HF, permanent or persistent AF, bundle branch block, and associated neuromuscular diseases.^368^

Echocardiography is routinely performed during initial investigations, and contrast use may improve diagnostic sensitivity.^369^CMR allows visualization of noncompacted and compacted myocardial segments and is able to identify thrombi and myocardial fibrosis.^370^

SCD is the leading cause of death in NCCM and may occur at any age. There are no diagnostic tools for accurate risk stratification in these patients. Ventricular arrhythmias are reported in 38% to 47% of cases, and SCD occurs in 13% to 18% of patients.^371^Histological examination shows continuity between the ventricular endocardium and the deep intertrabecular recesses; the latter may facilitate arrhythmogenesis by forming reentry circuits underlying the scar tissue, predominantly at the LV apex and mid-apical segments.^372^

Steffel et al. showed that SVT inducibility on EPS has limited value in the risk stratification of NCCM; in contrast, noninducibility may identify low-risk patients.^373^Endocardial and/or epicardial catheter ablation seems to be useful in patients implanted with an ICD who have frequent ventricular arrhythmias.^374 , 375^The rate of appropriate shocks in these patients, in secondary prevention, ranged from 33% to 37% during a mean follow-up period of 34 to 40 months.^376^

There are no convincing data demonstrating that LVNC alone is sufficient for an ICD indication. The indication should be guided by the severity of LV systolic dysfunction and the presence of sustained ventricular arrhythmia (similar to idiopathic dilated cardiomyopathy).^377 , 378^However, a study of 77 patients with NCCM showed that LV dysfunction and dilation were not prominent in patients receiving an ICD for secondary prevention, which means that these criteria are fragile for primary prevention indication. Conversely, the presence of NSVT was more common in patients receiving secondary prevention compared with those receiving an ICD for primary prevention or those who did not undergo ICD implantation. Other risk factors that should be considered are family history and syncope.^378^

Although there are no prospective studies addressing SCD prevention in NCCM, there are sufficient data from observational studies to support an ICD indication as a reasonable strategy for SCD prevention in these patients^379^( Table 26 ).

**Table 26:** – Recommendations for placement of implantable cardioverter-defibrillators for primary prevention of noncompaction cardiomyopathy

	Class of recommendation	Level of evidence
Patients with NCCM, LVEF ≤ 35%, NYHA functional class II and III HF, and a life expectancy of at least 1 year should receive a primary prevention ICD for SCD based on DCM recommendations	I	B
Patients with an NCCM diagnosis, normal ventricular function, and risk factors such as NSVT, family history of SCD, and syncope may receive a primary prevention ICD for SCD based on DCM recommendations	IIb	C
Patients with NCCM, normal ventricular function, and no risk factors should not be considered candidates for an ICD	III	C
Performing an EPS for risk stratification	III	C

*DCM: dilated cardiomyopathy; EPS: electrophysiology study; LVEF: left ventricular ejection fraction; MNC: noncompacted cardiomyopathy; NSVT: nonsustained ventricular tachycardia; NYHA: New York Heart Association.*

#### 4.1.7. Congenital Long and Short QT Syndromes

Congenital long QT syndrome (LQTS) is characterized by QT interval prolongation and polymorphic ventricular arrhythmias often triggered by adrenergic stimulation.^380^

Hundreds of mutations have been described in more than 13 different genes encoding ion channels that restore cardiomyocyte resting potential. LQTS may be inherited via an autosomal dominant pattern of transmission (Romano-Ward), such as in LQTS types 1 to 6; an autosomal recessive pattern associated with congenital deafness and very prolonged QT intervals (Lange-Nielsen); or an autosomal dominant pattern associated with extracardiac disorders, dysmorphism, and hypokalemic or hyperkalemic periodic paralysis (Andersen-Tawil, LQTS type 7).^379 , 381^

The risk of sudden death depends on several factors, such as the type of mutation causing the phenotype, QT interval duration, and the presence of symptoms. Patients with a very prolonged QT interval (QTc > 500ms) or recurrent syncope may be at an annual risk of sudden death of up to 5%.

Short QT syndrome (SQTS) is defined by a QTc interval < 340ms or < 360ms in survivors of cardiac arrest due to VF/VT, a family history of sudden death in those aged < 40 years, and the presence of a confirmed mutation or a family history of short QT. SQTS is a rare condition in which mutations in genes encoding potassium channels can be found in up to 20% of cases.^382^

Some patients with short QT may benefit from quinidine. Survivors of cardiac arrest should undergo ICD implantation, whereas asymptomatic patients should be closely monitored. The recommendations for ICD implantation for primary prevention of long and short QT syndromes are listed in Table 27 .

**Table 27:** – Recommendations for placement of implantable cardioverter-defibrillators for primary prevention of long and short QT syndromes

	Class of recommendation	Level of evidence
Patients with long QT refractory to beta-blockers and/or sympathectomy, with syncope or recurrent polymorphic VT	I	B
Asymptomatic patients with beta-blocker-related QTc > 500ms may benefit from an ICD alone or combined with sympathectomy	IIb	C
Beta-blocker-related long QT with genetic testing confirming LQTS types 2 or 3	IIb	B
Asymptomatic patients with QTc < 330ms and a family history of sudden death	IIb	C

*ICD: implantable cardioverter-defibrillator; LQTS: long QT syndrome; VT: ventricular tachycardia.*

#### 4.1.8. Brugada Syndrome

Brugada syndrome is characterized by ST-segment elevation > 2mm in leads V_1_and V_2_(coved type) in the second, third, or fourth intercostal space in combination with the occurrence of polymorphic ventricular arrhythmia, syncope, or cardiac arrest. ST-segment elevation may occur spontaneously or be induced by sodium channel blockers such as ajmaline and procainamide.

Patients with a spontaneous ECG pattern (type 1) associated with unexplained syncope or recovered cardiac arrest are at greatest risk of sudden death. ICD implantation is associated with risk reduction in symptomatic patients^383^( Table 28 ).

**Table 28:** – Recommendations for placement of implantable cardioverter-defibrillators for primary prevention of Brugada syndrome

	Class of recommendation	Level of evidence
SVT or syncope likely due to an arrhythmia and a spontaneous type 1 Brugada ECG pattern	I	B
Syncope likely due to an arrhythmia and a drug-induced type 1 Brugada ECG pattern	IIa	B
SVT induced by PES with one or two extrastimuli in two sites in asymptomatic patients with a spontaneous type 1 Brugada ECG pattern	IIb	C


*ECG: electrocardiogram; PES: programmed electrical stimulation; SVT: sustained ventricular tachycardia.*

The phenotype of Brugada syndrome is associated with detectable genetic defects in up to 30% of cases. The *SCN5A* gene is involved in most mutations, but a negative genetic test does not rule out the diagnosis.^384^

Several factors may trigger ECG manifestations or precipitate arrhythmic episodes, such as fever, anesthetic agents, and various psychotropic drugs (www.brugadadrugs.org).

Asymptomatic patients are at lower risk of sudden death. The role of programmed electrical stimulation (PES) in risk stratification is controversial. Brugada et al. found an association between polymorphic VT induction with up to 2 extrastimuli in the RV and the risk of death in asymptomatic patients. Arrhythmia induction with 3 extrastimuli reduces specificity and should be avoided. Other studies reported reductions in the positive predictive value of PES over time.^385 - 387^

Patients in electrical storm triggering ICD shock therapy may benefit from clinical management with quinidine and from epicardial ablation of abnormal RV activation regions identified by electroanatomical mapping.^388 , 389^

#### 4.1.9. Catecholaminergic polymorphic ventricular tachycardia

Catecholaminergic polymorphic ventricular tachycardia (CPVT) is a rare arrhythmogenic genetic disorder characterized by adrenergic-induced bidirectional and polymorphic VT. The estimated prevalence of the disease is 1:10,000. Two types of genes have been identified: a dominant variant secondary to a mutation in the cardiac ryanodine receptor gene ( *RyR2* ) and a rare recessive variant caused by a mutation in the cardiac calsequestrin gene ( *CASQ2* ).^390^

Clinical manifestations usually develop in the first or second decades of life and are triggered by exercise or emotional stress. ECG and echocardiogram are generally normal, but stress tests trigger atrial and ventricular arrhythmias (bidirectional and polymorphic).

A maximally tolerated beta-blocker dose is the treatment of choice. Flecainide and left thoracoscopic cardiac sympathectomy may be used as adjunctive therapies.

ICD implantation should be considered in patients with cardiac arrest, recurrent syncope, or VT despite ODT^391^( Table 29 ).

**Table 29:** – Recommendations for placement of implantable cardioverter-defibrillators for primary prevention of catecholaminergic polymorphic ventricular tachycardia

	Class of recommendation	Level of evidence
Patients with CPVT and syncope or SVT despite maximally tolerated beta-blocker doses or patients with a beta-blocker contraindication and a life expectancy > 1 year	IIa	C
Patients with asymptomatic CPVT and a good response to beta-blocker treatment	III	C

*CPVT: catecholaminergic polymorphic ventricular tachycardia; SVT: sustained ventricular tachycardia.*

In most cases, the ICD should be programmed with a long detection interval given that shock-related pain and stress may trigger more arrhythmias and, consequently, an electrical storm. The decision to implant an ICD should consider the high probability of shock delivery (appropriate and inappropriate) and the chance of complications associated with young patient age.

A recent systematic review reported incidence rates of 40% for shocks, 19.6% for electrical storms, 1.4% for post-implant mortality, and 32.4% for additional complications (lead fracture, endocarditis, and surgical revisions) in patients whose mean age was 15 years (11 to 21 years).^392^

#### 4.1.10. Idiopathic Ventricular Tachycardia

Ventricular arrhythmias in patients with normally structured hearts are mostly benign. However, a small number of patients may have malignant forms of monomorphic or polymorphic VTs and even ventricular fibrillation.

Many of these tachycardias are triggered by ventricular ectopics originating in very similar locations compared to those of benign aspect (outflow tract, aortic cusp, His-Purkinje system, mitral annulus, and papillary muscles). The exact mechanism of malignant ventricular arrhythmias is not completely understood yet. Anisotropy associated with slow conduction and functional block caused by rapid arrhythmogenic foci likely results in rhythm degeneration to VF and polymorphic VT.

High-risk characteristics are related to syncope or cardiac arrest and ECG findings of a short coupling interval in the first or second extrasystole, NSVT with short cycles, wide QRS complex (in VT or sinus rhythm), and polymorphic VT.^393 , 394^The recommendations for ICD implantation for primary prevention of idiopathic ventricular arrhythmias are listed in Table 30 .

**Table 30:** – Recommendations for placement of implantable cardioverter-defibrillators for primary prevention of idiopathic ventricular tachycardia

	Class of recommendation	Level of evidence
Patients with syncope, malignant NSVT, and a life expectancy > 1 year	IIb	C
Patients with benign asymptomatic idiopathic VT and a good response to pharmacological or ablative treatment	III	C

*NSVT: nonsustained ventricular tachycardia; VT: ventricular tachycardia.*

## 4.2. Secondary prevention of sudden death

### 4.2.1. Recovered cardiac arrest or sustained ventricular tachycardia

### 4.2.1.1. Recovered cardiac arrest or sustained ventricular tachycardia in the presence of structural heart disease

Cardiac arrest due to VT/VF and subsequent SCD constitute a serious public health issue, accounting for approximately 50% of all cardiovascular deaths. Additionally, the survival rate of out-of-hospital cardiac arrest is significantly low, ranging from 6% to 10%.^395^The few patients who survive cardiac arrest due to VT/VF are at high risk of recurrence of potentially fatal tachyarrhythmias. Thus, preventive measures are essential and include treatment of underlying heart disease and comorbidities, use of antiarrhythmic drugs, and adequate patient selection for ICD implantation.^396 , 397^

In patients with ischemic or dilated heart disease, the protective role of drugs such as beta-blockers, angiotensin-converting enzyme inhibitors (ACEIs), angiotensin receptor blockers (ARBs), and statins, which reduce total, cardiovascular, and sudden deaths, is well known.^398^

A meta-analysis^399^including more than 35,000 patients with LV dysfunction (LVEF <40%) revealed that beta-blockers, ACEIs, ARBs, and mineralocorticoid receptor antagonists reduce the risk of SCD compared with placebo (HR: 0.89, 95% CI 0.82-0.98; p = 0.02). When combined with ODT, ICD implantation provides additional benefits by reducing SCD rates (HR: 0.39, 95% CI 0.30-0.51; p < 0.0001). More recently, the combination of a neprilysin inhibitor (LCZ696) with an ARB (sacubitril/valsartan) was shown to be even more efficient than enalapril in reducing both HF and arrhythmic deaths.^258^Thus, whether LCZ696 has a primary antiarrhythmic function or whether cardiac arrhythmia reduction results from clinical HF improvement remains unknown.^400^

For several years, antiarrhythmic drugs were the main strategy for secondary SCD prevention, although their use was based on only a few studies that reported a high rate of recurrent events. Until the early 1990s, class I agents (eg, quinidine, flecainide, encainide) were believed to reduce ventricular extrasystoles and mortality. A subsequent demonstration of the deleterious effects of these drugs after AMI and in HF led amiodarone to become the treatment of choice in these patients. The Cardiac Arrest in Seattle: Conventional versus Amiodarone Drug Evaluation (CASCADE) study enrolled 228 survivors of cardiac arrest who were randomly assigned to empiric treatment with amiodarone or class I drugs guided by EPS or 24-hour Holter monitoring.^401^At 6-year follow-up, event-free survival (cardiac death or VT) was 41% in the amiodarone group vs 20% in the standard therapy group. However, the absence of a placebo group prevents a conclusion on whether the outcomes could be explained by amiodarone use or by the risks associated with other antiarrhythmic drugs.

The ICD is considered a major advance in secondary SCD prevention, and its benefits have been evaluated in several randomized clinical trials. The Antiarrhythmics Versus Implantable Defibrillators (AVID)^402^study compared antiarrhythmic therapy (amiodarone or sotalol) vs ICD in 1,016 patients with either recovered cardiac arrest due to VT/VF, VT causing syncope, or hemodynamic compromise and LVEF < 40%. Survival was significantly improved in the ICD group at 1 year (89.3% vs 82.3%), 2 years (81.6% vs 74.7%), and 3 years (75.4% vs 64.1%) (p < 0.02). The study was mostly criticized for the greater number of patients on beta-blockers in the ICD group compared with the antiarrhythmic therapy group. A subsequent analysis reported that ICD implantation mostly benefited patients with lower LVEF.^403^Survival did not differ significantly among patients with LVEF > 35%. In patients with LVEF between 20% and 34%, 1-year survival was 89.6% vs 79.8% and 2-year survival was 82.5% vs 71.8% (p < 0.05) in the ICD and antiarrhythmic groups, respectively. In patients with LVEF < 20%, 1-year survival was 82.4% vs 73% and 2-year survival was 71.6% vs 63.8%, with no significant differences.

The Canadian Implantable Defibrillator Study (CIDS)^404^evaluated amiodarone vs ICD in 659 patients with documented VF, recovered cardiac arrest, VT causing syncope, VT > 150bpm/min causing presyncope or angina in patients with LVEF <35%, or syncope associated with inducible VT or documented spontaneous VT. Total mortality after a mean follow-up period of 4 years was 27% in the ICD group vs 33% in the amiodarone group, with no significant differences. A subsequent analysis reported that ICD implantation was superior in patients with two of the following criteria: LVEF < 35%, NYHA functional class III or IV HF, and age > 70 years.^405^After a mean follow-up period of 5.6 ± 2.6 years, mortality was 47% in the amiodarone group vs 27% in the ICD group (p = 0.002).

The Cardiac Arrest Study Hamburg (CASH)^406^included 349 survivors of cardiac arrest who were randomly assigned to treatment with propafenone, amiodarone, metoprolol, or an ICD. Propafenone treatment was discontinued after an interim analysis found increased mortality compared with ICD implantation. After a mean follow-up period of 2 years, total mortality was 12.1% in the ICD group vs 19.6% in the amiodarone and metoprolol groups combined, with no significant differences.

A meta-analysis evaluating the results of those 3 studies demonstrated relative reductions of 50% in arrhythmic mortality (p < 0.0001) and 28% in total mortality among patients with an ICD compared with those who received antiarrhythmic treatment (NNT = 29, p < 0.00006).^407^Patients with LVEF <35% benefited the most at 6-year follow-up (patients with an ICD had an increased survival of 4.4 months).

The Midlands Trial of Empirical Amiodarone versus Electrophysiology-guided Interventions and Implantable Cardioverter-defibrillators (MAVERIC) compared EPS-guided therapies (antiarrhythmic drugs, ICD) vs empirical amiodarone in patients with SVT or recovered cardiac arrest.^408^The results showed reduced mortality in the ICD group and no benefits from performing an EPS.

The recommendations for ICD implantation in secondary prevention are based on those studies ( Table 31 ).

**Table 31:** – Recommendations for placement of implantable cardioverter-defibrillators in patients with recovered cardiac arrest or sustained ventricular tachycardia in the presence of structural heart disease

	Class of recommendation	Level of evidence
Cardiac arrest due to VT/VF without a reversible cause, LVEF ≤ 35%, and a life expectancy ≥ 1 year	I	A
Spontaneous SVT with hemodynamic compromise or syncope without a reversible cause, LVEF ≤ 35%, and a life expectancy ≥ 1 year	I	A
Survivors of cardiac arrest due to VT/VF without a reversible cause, LVEF ≤ 35%, and a life expectancy ≥ 1 year	IIa	B
Patients with spontaneous SVT without a reversible cause, LVEF ≤ 35% refractory to other treatments, and a life expectancy ≥ 1 year	IIa	B
Patients with syncope of unknown origin, inducible hemodynamically unstable SVT, and a life expectancy ≥ 1 year	IIa	B
Incessant VT	III	C

*LVEF: left ventricular ejection fraction; SVT: sustained ventricular tachycardia; VF: ventricular fibrillation; VT: ventricular tachycardia.*

### 4.2.1.2. Survivors of Cardiac Arrest or Sustained Ventricular Tachycardia in the Absence of Structural Heart Disease

Channelopathies are genetically determined diseases involving different types of cardiac ion channel dysfunctions, such as increased or reduced ion channel function and ionic imbalance These conditions increase the risk of potentially fatal tachyarrhythmias and SCD.^409^

The range of genetic mutations is extremely wide, with a large overlap of phenotypic expressions. Congenital LQTS, Brugada syndrome, catecholaminergic polymorphic ventricular tachycardia (CPVT), SQTS, J-wave syndrome, and early repolarization are classified as channelopathies. Although other mechanisms may be involved in the development of idiopathic VF, sudden arrhythmic death syndrome, and sudden childhood death syndrome, these are included in this section because of the predominant arrhythmic manifestation of VT/VF in the absence of structural heart disease. A detailed discussion of the characteristics of each channelopathy phenotypic expression is outside the scope of this guideline, but an extensive literature is available.^410 , 411^

Survivors of cardiac arrest with channelopathies are at a higher risk for a new SCD episode. ICD implantation in this setting reduces the risk of SCD, with a reported rate of appropriate therapy between 8% and 33%. Overall, patients with channelopathies and syncope or VT despite appropriate drug therapy have an ICD indication unless specific conditions, such as very young age or low weight, are considered high-risk for device implantation.^412^In special situations, the clinical decision to implant an ICD should also consider other therapeutic options or adjuvant therapies, such as left cardiac sympathetic denervation in LQTS ( Table 32 ).

**Table 32:** – Recommendations for placement of implantable cardioverter-defibrillators in patients with recovered cardiac arrest or sustained ventricular tachycardia in the absence of structural heart disease

	Class of recommendation	Level of evidence
Patients with a channelopathy, cardiac arrest due to VT/VF, and a life expectancy ≥ 1 year	I	B
Patients with a channelopathy who develop SVT or syncope despite drug therapy, with a life expectancy ≥ 1 year	IIa	B
Patients with congenital LQTS who develop syncope or SVT despite maximally tolerated beta-blocker dose	IIa	B
Patients with Brugada syndrome and spontaneous ECG abnormalities, syncope likely due to an arrhythmia, and a life expectancy ≥ 1 year	IIa	C
Patients with Brugada syndrome and documented SVT, with or without syncope, and a life expectancy ≥ 1 year	IIa	C
Patients with CPVT who progress with syncope or SVT despite maximally tolerated beta-blocker dose, with a life expectancy ≥ 1 year	IIa	C
Patients with Brugada syndrome and drug-induced ECG abnormalities, syncope of unknown origin, and a life expectancy ≥ 1 year	IIb	C

*CPVT: catecholaminergic polymorphic ventricular tachycardia; ECG: electrocardiography; LQTS: long QT syndrome; SVT: sustained ventricular tachycardia; VF: ventricular fibrillation; VT: ventricular tachycardia.*

### 4.2.2. Syncope and Ventricular Tachycardia/Fibrillation on Electrophysiology Study

Syncope is a rare, exuberant symptom with a range of etiologies, pathophysiological mechanisms, and prognoses, and may be the only symptom to precede sudden death. Syncope is defined as the sudden and transient loss of consciousness due to cerebral hypoperfusion, with rapid, spontaneous, and complete restoration of consciousness.

In the presence of heart disease, syncope (even of unknown origin) may indicate the need for an ICD for SCD prevention given the strong association between syncope and potentially fatal ventricular arrhythmias.^413^

In some cases, performing an EPS may be useful to confirm an arrhythmic cause. Inducible SVT is a predictor of SCD risk in ischemic heart disease with reduced LVEF,^414^whereas inducible VF is considered a nonspecific finding.^415^Conversely, patients without inducible SVT are considered low-risk^416^( Table 33 ).

**Table 33:** – Recommendations for placement of implantable cardioverter-defibrillators in patients with syncope and ventricular tachycardia/fibrillation induced on electrophysiology study

	Class of recommendation	Level of evidence
Patients with ischemic cardiomyopathy, LVEF > 35%, syncope of unknown origin, and inducible SVT on EPS^29-30^	I	B
Patients with nonischemic cardiomyopathy, syncope, inducible SVT on EPS, and no indication for primary prevention of sudden death^31-32^	IIa	B

*EPS: electrophysiology study; LVEF: left ventricular ejection fraction; SVT: sustained ventricular tachycardia.*

## 4.3. Children, Adolescents, and Congenital Heart Disease

ICD indications in children have not been properly addressed in recent international guidelines because of the limited number of published studies. Consequently, current indications are mostly based on some small case series.^417^ICD indications in pediatric patients often follow similar criteria to ICD indications in adults for both primary and secondary prevention strategies, although common sense is required when evaluating these patients.^418^

The Brazilian Society of Cardiac Arrhythmias (SOBRAC) and the Department of Congenital Heart Diseases and Pediatric Cardiology (DCC-CP) have recently published the Cardiac Arrhythmias in Children and Congenital Heart Diseases Guideline with the aim of standardizing the diagnosis and treatment of children with cardiac arrhythmias.^419^

It is extremely important that physicians seek alternatives to avoid an ICD indication in children, as long as they do not result in increased risk. Adequate options of clinical treatment should be exhausted and, when indicated, ablation of arrhythmogenic foci should be considered.

Unlike in the adult population, there is no evidence to support routine use of ICDs in pediatric patients based on LV dysfunction alone. ICD implantation in children should be an exception because pulse generator size and lead caliber are associated with technical difficulties due to small body size, limited sites for generator pocket creation (usually in the abdomen), limited access routes, risk of venous thrombosis/obstructions, and increased risk of extrusion.^420^

Clinical and electronic follow-up should include ECG and routine assessment by telemetry as well as periodic radiological assessment, which is essential to monitor lead behavior as the child grows. Lead implantation should be careful to leave a redundant curve to allow for patient growth without the need for multiple interventions.^421^

Electronic programming of the device in children also differs from that in adults. The PM function should consider a baseline pacing rate adjusted for age and type of heart disease, usually ranging from 90 to 160 ppm (programming used in adolescents usually follows adult protocols). Importantly, a rate-adaptive AV interval and a short but adequate post-ventricular atrial refractory period should be programmed to avoid tachycardia due to electronic reentry, given that these patients usually exhibit good ventriculoatrial conduction.

Electronic ICD programming and clinical follow-up should be even more careful.^422^Children may develop sinus tachycardia with increased heart rates very easily, which could result in inappropriate shock delivery with a major psychological impact. Inappropriate shocks, especially if immediately after implantation, may lead the patient to distrust the device and medical staff and develop difficult-to-treat panic syndrome.^423 - 428^

The criteria for arrhythmic detection and classification must be rigorously defined. Therapies should be restricted to shocks, and antitachycardia pacing (ATP) should be avoided because potentially fatal arrhythmias rarely manifest as monomorphic VTs.^429 - 431^In case of monomorphic VT development, ablation should be conducted whenever possible.^432 , 433^Polymorphic VTs respond better to early shock delivery and commonly deteriorate to VF during ATP attempts.

In adolescents, subcutaneous ICD placement may be an interesting option given that subcutaneous systems do not require intravascular leads.^434 - 437^However, use of larger generators and parasternal leads may cause local discomfort and image issues in thinner patients. Another limitation of subcutaneous ICDs is the inability to provide long-term antibradycardia pacing. These devices have been associated with inappropriate shocks due to a greater susceptibility to extracardiac noise detection.^438 , 439^

The main indications for ICD implantation in children and adolescents are summarized in Table 34 .

**Table 34:** – Recommendations for placement of implantable cardioverter-defibrillators in children and adolescents in the presence of congenital heart disease

	Class of recommendation	Level of evidence
Survivors of cardiac arrest, provided that reversible causes have been ruled out	I	B
Patients with stable VT and ventricular dysfunction, provided that reversible causes have been ruled out		A
Patients with symptomatic VT who have undergone hemodynamic and anatomical evaluation, provided that surgical correction or ablation of arrhythmogenic substrate is not an option		C
Patients with recurrent syncope and ventricular dysfunction or induced VT	IIa	B
Patients with recurrent syncope associated with LQTS or catecholaminergic polymorphic VT despite maximally tolerated beta-blocker dose		B
Patients with asymptomatic congenital long QT unresponsive to treatment or a family history of sudden death		C
Patients with asymptomatic hypertrophic obstructive cardiomyopathy, with high-risk features		C
Patients with arrhythmogenic RV cardiomyopathy and extensive ventricular involvement (RV and/or LF), VT, a family history of sudden death, or syncope of unknown origin		C
Incessant VT or VT due to a reversible cause	III	C

*LQTS: long QT syndrome; LV: left ventricle; RV: right ventricle; VT: ventricular tachycardia.*

The evolution of surgical treatment for congenital heart diseases has led to increased survival rates among young adults. The presence of myocardial scarring secondary to the underlying congenital heart disease or to surgical treatment may result in complex ventricular arrhythmias or even SCD in these patients.

There is a significant correlation between residual hemodynamic abnormalities and VT in patients undergoing surgical correction of tetralogy of Fallot (TOF). RV hypertrophy and dilation together with residual RVOT obstruction and regurgitation are considered risk factors for VT and SCD.^440 - 446^A hybrid approach combining surgical repair of structural abnormalities and arrhythmia ablation guided by pre- or intraoperative mapping has been successfully used for reducing the incidence of arrhythmias.^447 - 449^Pulmonary valve replacement alone in patients with TOF results in hemodynamic and functional improvement but does not eliminate VT risk. SCD risk assessment should be conducted postoperatively to evaluate whether an ICD should be indicated.^450 , 451^

Approximately 50% of ICD implants in adults with congenital heart disease are indicated for secondary prevention in patients between 36 and 41 years of age.^452 , 453^The rate of appropriate shocks in these patients ranged from 3% to 6% per year, and the rates of inappropriate shocks (15% to 25%) and complications (26% to 45%) were higher than those of other populations.^450 , 451 , 454 - 457^Thus, an ICD indication in this population should consider cost-effectiveness and psychological impact.

ICD implantation in adults with congenital heart disease may be challenging because of anatomical complexity, intracardiac shunts, and limited vascular access. A subcutaneous ICD may be a good option for these patients.^456^

An ICD indication for primary prevention in patients with congenital heart disease is controversial. Kairy et al. proposed a risk score for patients undergoing surgical correction of TOF in which a score > 5 is considered sufficient to indicate an ICD. The score included the following criteria: previous palliative shunt (2), inducible SVT on EPS (2), QRS ≥ 180ms (1), ventriculotomy (2), NSVT (2), and LV end-diastolic pressure ≥ 12mmHg (3).^457^

Patients with surgically repaired TOF account for approximately 50% of ICD implantations in adults with congenital heart disease. Annual rates of appropriate shocks in this population have been reported at up to 7.7% in primary prevention and 9.8% in secondary prevention.^458^Inducible SVT on EPS in patients with congenital heart disease does not seem to correlate with the occurrence of appropriate shocks.^458^In some cases, catheter ablation of recurrent monomorphic SVT may be an effective alternative to prevent ICD implantation.^459 - 463^

Among patients with operated congenital heart disease, those with transposition of the great arteries (TGA) via atrial switch procedure, Ebstein anomaly, aortic valve stenosis, and single ventricle physiology are at higher risk for SCD.^464 - 467^

Patients with a previous Senning or Mustard procedure are at higher risk for SCD, especially during exercise. In these patients, atrial switch procedures may result in increased volume and consequent stenosis of the pulmonary veins and increased end-diastolic pressures.^468^Additionally, myocardial perfusion studies have identified RV ischemia and infarction in more than 40% of patients.^469 , 470^Risk factors for cardiac arrest in patients who had atrial switch procedures include previous ventricular septal defect closure, HF symptoms, atrial arrhythmia, right ventricular ejection fraction between < 30% and 35%, and QRS > 140ms.^471 , 472^A multicenter study evaluating patients who had atrial switch procedures and ICD implants reported that the lack of beta-blockers was associated with a high risk of appropriate ICD therapy.^464^Since atrial arrhythmias often precede SVT in patients with TGA, atrial tachycardia treatment should be intensified.^473 , 474^

SCD risk is higher among patients with adult congenital heart disease ( Table 35 ) compared with the general population; mean age at death ranges from 30 to 49 years.^473 - 476^Patients with moderate to severe congenital heart disease are at even greater risk of SCD, accounting for approximately 25% of all causes of cardiac death.^477 , 478^

**Table 35:** – Congenital heart disease and risk of sudden cardiac death

Congenital heart disease	Sudden death incidence	High-risk features
**Simple**		
Atrial septal defect	< 1.5%	Ventricular pacing; RV dilation; pulmonary hypertension; *NKX2-5* gene
Ventricular septal defect	< 3%	Ventricular pacing; RV dilation; pulmonary hypertension; *NKX2-5* gene
**Moderate**		
Tetralogy of Fallot	1.4% to 8.3%	Unexplained syncope; complex ventricular arrhythmia or sustained ventricular tachycardia; QRS >180 ms; inducible sustained ventricular tachycardia; atrial tachycardia; LV dysfunction; significant RV dilation; severe pulmonary insufficiency or stenosis
Aortic stenosis	3% to 20%	Unexplained syncope; significant LV hypertrophy; aortic stenosis with mean gradient >40 mm Hg; ventricular dysfunction
Coarctation of the aorta	2%	Aneurysm at the repair site; aortic stenosis; hypertension; early coronary artery disease
Ebstein anomaly	3% to 6%	Cardiomegaly; atrial fibrillation; tachycardia with wide QRS complex; mitral regurgitation; RV outflow tract dilation
**Severe**		
Transposition of the great arteries	Atrial switch 3% to 9.5%; Arterial switch 1%; Congenitally corrected 17% to 25%	Mustard atrial switch; previous ventricular septal defect closure; unexplained syncope; atrial tachycardia; coronary ostial stenosis; systemic ventricular dysfunction; tricuspid valve regurgitation
Truncus arteriosus	4%	Multiple repair operations; coronary artery anomalies; ventricular dysfunction and/or hypertrophy
Fontan procedure for single ventricle physiology	2.8% to 5.4 %	Atrial tachycardia; long-term survival; protein-losing enteropathy; ascites

*LV: left ventricular; RV: right ventricular.*

Family history and presence of septal defects, cardiomyopathy, or conduction system blocks may be related to a mutation in the *NKX2-5* gene, which is associated with early SCD risk. A positive genetic test warrants ICD implantation.^479 - 481^

Patients with complex forms of congenital heart disease and multiple surgical interventions in the first decades of life and patients with hypertrophy with subsequent subendocardial ischemia are at increased risk of potentially fatal ventricular arrhythmias. Other risk factors for SCD in patients with congenital heart disease include greater disease complexity, ventricular and supraventricular arrhythmias, progressive increase in QRS duration, systemic ventricular dysfunction, and subpulmonary ventricular dysfunction. A history of unexplained syncope in adults with moderate to severe congenital heart disease may be suggestive of SCD risk; in such cases, an EPS may be performed to assess the need for an ICD.^457^

Adults aged 40 to 50 years account for 40% to 67% of patients with congenital heart disease who receive an ICD for primary prevention. In these patients, the rates of appropriate shocks ranged from 14% to 22% in the first 3 to 5 years of follow-up.^482^In patients without vascular access or with a previous Fontan procedure, the risks of epicardial ICD implantation may outweigh the potential benefits; therefore, subcutaneous ICD implantation or heart transplantation should be considered.^478^

The safety of antiarrhythmic therapy in patients with congenital heart disease may be affected by the presence of ventricular hypertrophy and dysfunction. Flecainide use was associated with proarrhythmia in 5.8% of patients and SCD in 3.9%.^482^Conversely, amiodarone is usually reserved for patients with symptomatic arrhythmias or for prevention of worsening ventricular function.^483 , 484^

The main recommendations for ICD placement in adult patients with congenital heart disease are listed in Table 36 .

**Table 36:** – Recommendations for placement of implantable cardioverter-defibrillators in adult patients with congenital heart disease

	Class of recommendation	Level of evidence
In adult patients with congenital heart disease and complex or sustained ventricular arrhythmia in the presence of significant residual hemodynamic abnormalities, ICD implantation is indicated only after treatment of abnormalities (if possible)	I	B
In adult patients with congenital heart disease and unstable VT, ICD implantation is recommended only after evaluation and adequate treatment of residual abnormalities/ventricular dysfunction, with expected survival > 1 year	I	B
In adult patients with repaired tetralogy of Fallot and inducible VT/VF or spontaneous SVT, ICD implantation is reasonable if expected survival > 1 year	IIa	B
In adult patients with highly complex congenital heart disease who underwent surgical repair and frequent and/or complex ventricular arrhythmias, beta-blockers may help reduce the risk of sudden death	IIa	B
In adult patients with previously repaired moderate to severe congenital heart disease, unexplained syncope and moderate ventricular dysfunction or marked hypertrophy, an ICD indication is reasonable if expected survival > 1 year in cases of inducible SVT on EPS	IIa	B
In adult patients with congenital heart disease, LVEF < 35%, and HF symptoms, ICD implantation may be considered if there is expected survival > 1 year (even in the presence of additional risk factors)	IIb	B
In patients with incessant VT or VT due to a reversible cause	III	C

*EPS: electrophysiology study; HF: heart failure; ICD: implantable cardioverter-defibrillator; LVEF: left ventricular ejection fraction; SVT: sustained ventricular tachycardia; VF: ventricular fibrillation; VT: ventricular tachycardia.*

Choosing Implantable Cardioverter-defibrillator Type and Pacing Mode

## 4.4. Choosing Implantable Cardioverter-defibrillator Type and Pacing Mode

Once an ICD is indicated for SCD prevention, the physician must choose the surgical technique for device implantation (transvenous, epicardial, or subcutaneous) and pacing mode (ventricular, atrioventricular, biventricular, or atrio-biventricular).

### 4.4.1. Implantation Technique

In the absence of an atrial septal defect, patients weighing > 15kg normally undergo transvenous implantation.^485^If antibradycardia pacing (AV block, SND) is not required, a subcutaneous ICD may be a good option.

### 4.4.2. Pacing Mode

In patients requiring a PM function, the choice of pacing mode is crucial. Pacing modes that prioritize the preservation of spontaneous AV and IV conductions are associated with a lower incidence of AF and ventricular remodeling related to RV pacing-induced LBBB.^486^Furthermore, in patients with LV remodeling at the time of implantation (LVEF ≤ 40% and LV end-diastolic diameter ≥ 60mm) requiring ventricular pacing, biventricular pacing is superior to RV pacing alone.^487 , 488^

Therefore, after an ICD indication, the choice of pacing mode should consider whether chronotropism and AV conduction are normal, whether IV conduction follows a spontaneous or PM-induced LBBB pattern, and whether there is LV remodeling.

Patients with SND and normal AV and IV conductions may receive either single- or dual-chamber ICDs provided that intrinsic conduction search algorithms are programmed to avoid PM-induced dyssynchrony.

## 4.5. Cost-effectiveness of Implantable Cardioverter-defibrillators in Primary and Secondary Prevention of Sudden Death

Although ICDs increase survival in patients with LV dysfunction at risk of SCD, their use is limited by high therapy costs. These costs refer to the device itself, hospital expenses, medical fees, complications, readmissions, and pulse generator and lead replacement.

Cost-effectiveness analyses consider the cost in local currency per quality-adjusted life years (QALYs)^489^or life years gained (LYG),^490^varying according to socioeconomic and cultural factors specific to each study population.

Cost-effectiveness should be analyzed in terms of mortality, based on multicenter studies. For example, if the ICD does not improve survival, it is not cost-effective. Therefore, expected survival is a key factor in cost-effectiveness analyses.

The risk of death from nonarrhythmic causes should also be considered given that ICDs are not recommended for this purpose. The cost-effectiveness ratio becomes unfavorable when the survival rate of ICD candidates is < 1 year; therefore, ICD implantation in patients with high morbidity and mortality may not be cost-effective. In older patients, some studies suggest that the expected survival should be > 5 years to achieve cost-effectiveness.^491^Cost-effectiveness studies evaluating ICD implantation for less common diseases, such as hypertrophic cardiomyopathy and channelopathies, are lacking.

### 4.5.1. Primary Prevention

Sanders et al.^492^analyzed the results of the MADIT, MADIT II,^247^COMPANION,^161^MUSTT,^493^SCD-HeFT,^245^and DEFINITE^494^studies and projected that ICD use would provide a gain of 1.01 to 2.99 QALYs at a cost of US$68,300,00 to US$101,500.00. Assuming that the generator would be replaced every 5 years, cost-effectiveness was estimated at US$30,000.00 to US$70,200.00 for each QALY gained compared with the results for controls. The authors estimated that cost-effectiveness would be less than US$100,000 per QALY if the ICD reduced mortality for ≥7 years.

In another analysis conducted by Sanders et al., patients treated with an ICD in the DINAMIT and CABG Patch trials^250^did not experience mortality reductions compared with controls; therefore, ICD implantation was not cost-effective. In the DINAMIT trial, an ICD was implanted 6 to 40 days after myocardial infarction in patients with LVEF ≤ 35% and depressed heart rate variability. The primary outcome (death from any cause) did not differ between the ICD group and the control group. The CABG Patch trial enrolled patients with coronary heart disease, EF ≤ 35%, and abnormalities on SAECG undergoing CABG surgery. Prophylactic ICD implantation at the time of CABG surgery did not reduce the primary endpoint of death from any cause. Thus, prophylactic ICD implantation in patients at high risk for sudden death (EF ≤ 35%, abnormal SAECG, and depressed heart rate variability) was not cost-effective in the first 40 days post-infarction or immediately after CABG surgery.

A 2010 Brazilian study evaluated the cost-effectiveness of ICDs in patients with HF from both public and private health perspectives (effectiveness was measured in QALYs).^495^Ribeiro et al.^495^reported that the cost-effectiveness ratio was R$68,318 per QALY in the public setting and R$ 90,942 per QALY in the private setting. The variables with the highest impact in the analysis were the costs of ICD implantation, the frequency of generator replacement, and ICD effectiveness. In more complex study populations, such as the MADIT population, cost-effectiveness was much more favorable in the public setting (R$23,739.00 per QALY) than in the private setting (R$33,592.00 per QALY).^496^

In a 2007 Brazilian study, Matos et al. evaluated the cost-effectiveness ratio of ICDs vs drug treatment per LYG.^497^The calculated cost per LYG was R$20,530.00 (US$9,550.00) at the time. This calculation was based on the parameters of an incremental R$54,200.00 cost and a life expectancy of 2.64 years gained from ICD implantation compared with clinical treatment. The cost-effectiveness ratio was considered favorable for Brazilian standards.

In the UK, Buxton et al. (2006) reported costs of £57,000 per LYG and £76,000 per QALY over a long-term follow-up period.^498^The authors concluded that cost-effectiveness would be more favorable in patients with low LVEF, including higher-risk subgroups, but not for routine use.

Cowie et al. conducted in 2009 a meta-analysis of primary prevention studies in the European setting including patients with reduced LVEF and ICD indications according to European guidelines. Prophylactic ICD implantation was found to have a good cost-effectiveness ratio.^499^Estimated mean LYG and QALY were 1.88 and 1.57, respectively, and mean estimated cost per QALY was €31,717. These findings were reproduced in another European registry evaluating a primary prevention setting.^500^

In a systematic review of economic evaluations, Gialama et al. (2014) reported that ICDs may have a good cost-effectiveness ratio in selected patient groups,^496^comparable to other established therapies for cardiovascular and noncardiovascular diseases. Variables such as ICD efficacy and safety, device costs (implantation and replacement), patient characteristics, and SDC risk had the highest impact in the analysis.

### 4.5.2. Secondary Prevention

In the Antiarrhythmics Versus Implantable Defibrillators (AVID) study, Larsen et al. evaluated the cost-effectiveness of ICDs compared with antiarrhythmic drugs (mostly amiodarone) in survivors of SVT and VF.^501^At 3 years, ICD cost-effectiveness per LYG was estimated at $66,677.00 (95% CI $30,761.00 to $154,768.00) compared with antiarrhythmic drug therapy. The 6- and 20-year projections estimated costs of approximately $68,000 and $80,000 per LYG. In the subgroup analysis, ICD implantation was more cost-effective in patients with VF and less cost-effective in patients with EF > 35%.

Thijssen et al.^502^evaluated ICD cost-effectiveness and found acceptable results compared with those of other treatments available in the public health system, such as erythropoietin in patients on dialysis, some chemotherapies for leukemia in older patients, lung transplantation, and neurosurgery for malignant intracranial tumors. The cost per QALY was similar to those of heart transplantation, hemodialysis, and peritoneal dialysis. Importantly, some factors may considerably reduce cost-effectiveness, such as complications, infections, and comorbidities that negatively impact patient survival and ICD longevity.

Appropriate and inappropriate shocks may reduce survival and quality of life and, consequently, cost-effectiveness. Several studies have analyzed the importance of programming ICDs with longer SVT detection times and increased heart rate detection. These features were able to avoid inappropriate and “unnecessary” shocks, with improved survival rates and/or reduced hospitalization rates.^503 , 504^Thus, less aggressive programming may improve the cost-effectiveness of ICDs.

Mealings et al. analyzed 13 cost-effectiveness studies of ICDs and CRTs using an analytical method to adapt treatment costs to the UK setting.^505^Cost-effectiveness was evaluated in several subgroups of patients based on clinical criteria such as functional class, QRS duration, age, presence of LBBB, and ischemic etiology. At a maximum acceptable cost of £30,000 per QALY, ICDs were cost-effective in patients with HF, LV systolic dysfunction, NYHA functional class < IV, and QRS <120ms. In patients with QRS between 120 and 149 ms, the ICD was cost-effective only in those with NYHA functional class I and II HF. In patients with NYHA functional class IV HF, ICD was cost-effective only when combined with CRT in patients with LBBB and QRS >120ms.

In older patients, especially those aged >80 years, the clinical efficacy and cost-effectiveness of ICDs are uncertain. Mean patient age at the time of enrollment in primary and secondary prevention studies was 58 to 66 years and 58 to 65 years, respectively. However, approximately 28% of eligible patients for ICD implantation are estimated to be over 80 years of age.^506^Real-world data revealed that approximately 8% to 12% of implants in the USA and Canada are placed in patients aged > 80 years. The sudden death/death from any cause ratio decreases with age, being 0.51 in patients aged < 50 years and 0.26 in patients aged > 80 years.^507^Given that the number of appropriate ICD therapy is similar in all age groups in both primary and secondary prevention, the sudden death/death from any cause ratio decreases in older patients because of an increase in deaths from other comorbidities.

Pellegrini et al. evaluated the impact of age at the time of ICD implantation on survival.^491^Patients were categorized as < 65, 65 to 75, and > 75 years of age.^501^Mean survival after ICD implantation in patients aged > 75 years was 5.3 years (half than in the other groups). For survival rates < 5 years, the cost per QALY would increase from $34,000-$70,200 (Sanders^4^) to $90,000-$250,000. In this scenario, the ICD would not be cost-effective if the patient died less than 5 years after implantation.

The cost-effectiveness ratio of ICDs in Brazil and other developing countries needs to be assessed within the socioeconomic context of each country considering local aspects, gross domestic product, effectiveness, and complications. Thus, patients with compromised LV function, fewer comorbidities, and at higher risk of death from arrhythmia should be prioritized.

Reducing the cost of long-lasting devices and batteries can significantly increase cost-effectiveness. Additionally, every effort should be made to avoid inappropriate or unnecessary shocks, which would improve quality of life (a positive impact on the QALY index assessment) and increase battery longevity.

## 5. Recommendations for Implantable Loop Recorders

Implantable loop recorders allow continuous heart rhythm monitoring regardless of active patient participation. With the ability to record different events (bradycardia, tachyarrhythmia, pauses) and a battery life of approximately 3 to 4 years, the implantable loop recorder is an extremely attractive diagnostic tool in the investigation of unusual symptoms (eg, less than once a month) that may be attributed to bradyarrhythmias or tachyarrhythmias.^508^

In patients with unexplained syncope in whom initial noninvasive investigation with ECG, 24-hour Holter, or prolonged monitoring could not explain the nature of the symptoms, implantable loop recorders have been superior to conventional investigation strategies, such as tilt table test and invasive EPS. Several studies have identified bradyarrhythmia as the main cause of syncope, particularly in older patients with an IVCD. In these cases, bradyarrhythmia was identified in up to 41% of cases, 70% of which were intermittent complete AV blocks.^509^

In patients with cryptogenic ischemic stroke without documented AF, active investigation with serial ECG and prolonged monitoring can detect silent AF episodes in up to 23% of cases.^510^AF detection in these patients may change the course of treatment, and full anticoagulation therapy may be required indefinitely. However, randomized trials are still needed to support the efficacy of anticoagulant therapy in patients with silent AF detected by prolonged monitoring in cryptogenic stroke.

The Cryptogenic Stroke and Underlying AF (Crystal AF) study randomly assigned 441 patients with cryptogenic stroke after initial investigation to monitoring by an implantable loop recorder or conventional follow-up.^511^At 6-month follow-up, AF > 30s was detected in 8.9% of patients with an implantable loop recorder. At 12 months, AF was detected in 12% of these patients vs 2% of patients with conventional follow-up (p < 0.001).

The Stroke of Known Cause and Underlying Atrial Fibrillation (STROKE-AF) trial enrolled 496 patients aged > 50 years and was presented at the 2021 International Stroke Conference (late-breaking abstract 6). AF episodes > 2 minutes occurred in 12% of patients with an implantable loop recorder vs 1.8% in the control group (p < 0.001). Patients with an implantable loop recorder received more anticoagulation therapy and had reduced stroke recurrence. Although not derived from controlled studies comparing different therapeutic interventions, the data suggest that prolonged monitoring may be beneficial.

The current recommendations for implantable loop recorders are listed in Table 37 .

**Table 37:** – Recommendations for implantable loop recorders

	Class of recommendation	Level of evidence
Patients with recurrent syncope of unknown origin and no formal indication for a pacemaker or an ICD, after inconclusive clinical and laboratory investigation	I	A
Patients with recurrent palpitations of probable arrhythmic cause in whom other diagnostic methods found no correlation with symptoms	IIa	B
Cryptogenic stroke for AF detection in patients with a negative or inconclusive noninvasive investigation	IIa	B
Patients with suspected recurrent reflex syncope presenting with frequent and severe episodes	IIa	B
Patients with epilepsy in whom treatment was ineffective	IIb	B
Patients with unexplained falls	IIb	B

*AF: atrial fibrillation; ICD: implantable cardioverter-defibrillator.*

## 6. Recommendations for CIED Evaluation and Programming

### 6.1. Conventional Pacemakers

PM programming should adhere to the following basic principles:^502 , 512^

To preserve or restore baseline resting heart rate and adapt it to stress demands, as well as to restrict pacing to the condition for which it was indicated, avoiding pacing when there are no proven benefits.
To preserve intrinsic atrioventricular conduction whenever possible.
To increase the longevity of the pulse generator battery while providing clinical benefits to the patient without compromising safety.
To detect arrhythmias and system malfunctions.

The care of patients with a PM should include electronic and clinical evaluation. In addition to personal (including current medications) and family history, investigation of symptoms, and physical examination, a 12-lead ECG should be performed, which is essential to assess sensing, capture, and arrhythmia functions. Echocardiography, although usually performed before implantation, may be required in the follow-up period to monitor LV remodeling due to the possible deleterious effects of chronic RV pacing and PM syndrome.

The electronic evaluation is performed by telemetry and should include the pulse generator, the leads, and the retrieval of data stored in the device’s memory, especially arrhythmic events and malfunctions.

System interrogation allows the assessment of generator battery longevity, lead integrity, and pacing and sensing threshold measurements. Temporarily inhibiting the PM function allows the identification of intrinsic rhythm, which is essential for optimal system programming. Statistical data related to each cardiac chamber and to arrhythmic events should be accessed, as well as intracavitary electrogram recordings.

The choice of pacing mode should consider the patient’s intrinsic rhythm: normal sinus, AF, SND, and/or AV block.

#### 6.1.1. Sinus Node Disease

Single-chamber ventricular pacing (VVI) was initially widely used regardless of bradycardia type because of its simplicity and safety. However, more than a quarter of patients with VVI pacing develop PM syndrome (ventricular pacing that causes retrograde atrial conduction and results in symptoms such as dyspnea, palpitations, dizziness, and signs of low cardiac output), with significantly impaired quality of life. Thus, atrial pacing allows spontaneous AV and IV conduction in SND, preventing loss of AV synchronism, PM syndrome, and IV dyssynchrony secondary to RV pacing.^513 , 514^

Atrial pacing may be programmed to AAI or DDD mode. The DDD mode allows the preservation of intrinsic conduction through specific algorithms but has more complications, such as lead dislodgement, than the VVI mode. Conversely, AAI devices require twice as many reoperations compared with DDD devices, often due to AV block development (injury progression). AV blocks in patients with SND occur between 0.6% and 1.9% per year, requiring system upgrade to DDD mode.^515 , 516^

AAI mode is associated with a lower occurrence of AF and thromboembolic events compared with VVI mode in patients with SND. Similar results were observed with DDD mode, which is also associated with lower rates of AF and better quality of life compared with VVI mode. However, these benefits have no impact on the outcomes of mortality, HF, and cardiovascular death.^33 - 35^

The deleterious effects of RV pacing may result in HF and poor survival as a consequence of induced dyssynchrony. Thus, algorithms that preserve intrinsic AV conduction, automatically prolong the AV interval, or promote device upgrade to AAI mode (with ventricular backup) should be programmed when using a dual-chamber PM to avoid unnecessary RV pacing in patients with preserved AV conduction. Initial data suggest that these algorithms promote significant reductions in ventricular pacing percentage (99% to 9%, p < 0.001) and AF (40%). Patients with SND-related first-degree AV block may lose these benefits if the PR interval is too long.^517^

In the DANPACE study, which enrolled 1,415 patients with SND, DDDR mode with a maximum AV interval of 220ms was associated with a lower occurrence of paroxysmal AF; a very prolonged AV interval was associated with mitral regurgitation, increased preload, and AF, suggesting that there is a limit to AV interval prolongation. Thus, programming AV intervals > 220ms is not usually recommended.

Sensors for rate-responsive pacing are another important PM feature in SND. These sensors aim to increase heart rate in cases of increased metabolic demand, such as during physical exercise. Three small studies reported improved quality of life and increased exercise tolerance during sensor activation; however, these findings were not reproduced in the ADEPT study.^507 , 518^

The automatic mode switching (AMS) function reverses DDD(R) to VVI(R) mode in case of AF development. Although there is no strong evidence supporting the benefits of AMS, it should be programmed particularly in patients with paroxysmal AF for symptom relief.^519^

#### 6.1.2. Atrioventricular Block

RV pacing in AV block may be programmed to DDD or VVI mode. DDD mode maintains AV synchrony but is associated with more complications (6.2% vs 3.2%), especially lead dislodgement, threshold increases, and infection.^520^

In studies comparing DDD vs VVI mode in patients with complete AV block and SND (PASE, CTOPP), DDD mode was not associated with reduced cardiovascular mortality or hospitalizations. The CTOPP study reported AF reduction with DDD mode (greater benefit in patients with SND); however, 26% of patients in VVI mode had PM syndrome and had to be crossed over to DDD mode. Patients’ conditions improved significantly after upgrade to DDD mode.^31 , 33^In patients aged > 70 years, DDD mode did not seem to be superior to VVI mode in those with complete AV block after a 3-year follow-up period (including PM syndrome). Therefore, DDD mode is a suitable alternative in older patients with low life expectancy and physical activity restrictions.

#### 6.1.3. Atrial Fibrillation

In patients with permanent AF and no possibility of reversion to sinus rhythm, only ventricular pacing is required. In these cases, VVI(R) mode is recommended. Sensors for rate-responsive pacing have been associated with improved functional capacity and quality of life in small studies.^521 , 522^

#### 6.1.4. Neurally Mediated Syncope and Carotid Sinus Syndrome

Neurally mediated syncope with a cardioinhibitory response is characterized by periods of intermittent bradycardia, requiring limited pacing periods with increased baseline rate to compensate for the sudden instability occurring during the event. In these cases, pacing should last for only a short period of time, only during symptomatic episodes (hysteresis function). DDI, DVI, or DDD mode may be programmed in combination with an algorithm that preserves intrinsic conduction. VVI mode has been more closely associated with syncope and presyncope occurrence than dual-chamber pacing (DDD and DVI) in some studies.^72 , 73^

Algorithms such as the Rate Drop Response (RDR)^®^and Sudden Brady Response (SBR)^®^can identify abrupt drops in heart rate and respond by applying an accelerated intervention rate at programmable intervals. These algorithms are effective for symptom improvement in patients with neurally mediated syncope (cardioinhibitory) compared with conventional treatment without a PM. Although these algorithms have not been compared with other pacing modes, they are effective and allow PM inhibition for most of the time. In the International Study on Syncope of Uncertain Etiology III (ISSUE III), DDD mode plus RDR reduced the chance of syncope recurrence by 57%. RDR was programmed to intervene if heart rate reached 40 bpm or dropped by 20 beats from baseline heart rate (90bpm for 1 minute).^523^

The Closed Loop Selection (CLS)^®^algorithm uses intracardiac impedance measurements to assess myocardial contractility changes in order to predict syncope onset and initiate intervention (myocardial contractility increase occurs in the early phase of syncope episodes).^520 , 524^

The automatic capture threshold testing feature has proven to be safe and may prolong the pulse generator longevity by 60%, reducing costs by 42% in 10 years. With few exceptions, these algorithms should be programmed routinely^525^( Table 38 ).

**Table 38:** – Electronic programming recommendations for conventional pacemakers

	Class of recommendation	Level of evidence
DDD mode with a maximum AV interval of 220ms and algorithms for intrinsic conduction search in SND	I	A
DDD mode in complete AV block to avoid pacemaker syndrome	I	B
VVIR mode in permanent atrial fibrillation	I	C
The automatic mode switching function should be programmed routinely	I	C
Sensors for rate-responsive pacing in patients with chronotropic incompetence	IIa	B
The automatic capture threshold testing feature should be programmed routinely to prolong generator longevity	IIa	B
Specific algorithms, hysteresis function, and a prolonged AV interval should be programmed to avoid unnecessary pacing in neurally mediated syncope and carotid sinus hypersensitivity	IIa	B

*AV: atrioventricular; SND: sinus node disease.*

## 6.2. Cardiac Resynchronization Therapy

The evaluation of patients with CRT should follow the same principles for conventional PMs and include parameters specific to dyssynchrony correction. Thus, periodic ECG allows evaluating whether biventricular pacing is active, whereas conducting an echocardiogram 90 days after implantation and repeatedly throughout follow-up should document reverse remodeling in patients who respond to CRT.

The pattern of ventricular activation depends on lead placement and how early each chamber is activated. LV activation results in right axis deviation, with a qR or Qr pattern in lead I and r or R waves in V_1_(see Figure 4 ). Ventricular fusion occurs during biventricular pacing. A qR or Qr pattern in lead I was observed in 90% of patients with biventricular pacing. Loss of the q or Q wave in lead I is highly suggestive of LV capture loss. R or r waves in V_1_were observed in 65% to 93% of patients with biventricular pacing.^526 , 527^

**Figure 4:** – QS pattern in D1 and initial r wave in V1 proving early activation of the LV lateral wall in biventricular pacing.

CRT loss may occur during exercise in the presence of AF with high ventricular rate or due to AV interval shortening; 24-hour Holter monitoring and exercise stress test may be useful to identify such cases. Increased LV capture thresholds account for 10% of cases of CRT pacing loss.^517^This change should be suspected when there is loss of Qr or qR pattern in lead I on 12-lead ECG and confirmed by a pacing threshold test. Current systems have an automatic capture threshold testing feature that helps to identify variations in pacing thresholds that have not been detected during conventional assessment.

Protocols from large CRT studies should be followed when programming basic parameters: DDD 50bpm, sensed AV interval of 100 to 120ms, and VV interval of 0ms (simultaneous biventricular pacing), with deactivation of sensors for rate-responsive pacing. Patients with AF should be programmed to DDI mode if there is an atrial lead, VVI if there is no atrial lead, and DDD if there is paroxysmal AF. In these cases, a rate of 60bpm is recommended in the absence of chronotropic incompetence.

Most large studies use DDD or VDD mode with a rate between 35 and 60bpm to reduce atrial pacing, which could compromise AV synchrony in patients with delayed interatrial conduction and impair ventricular filling. HF guidelines recommend the use of heart rate-lowering drugs when heart rate is > 70bpm despite the use of beta-blockers, which supports the programming of low baseline rates and routine deactivation of rate-responsive pacing sensors.^528^

A short AV interval (100 to 120ms) aims to achieve a biventricular pacing percentage of approximately 100%, avoiding biventricular capture loss associated with PR interval shortening. AV and VV interval optimization by echocardiography or other methods is usually reserved for patients who do not respond to CRT, since there is no consensus on the real usefulness of these methods in routine practice.^529^

The maximum rate of atrioventricular synchrony should be programmed to the maximum, considering the maximum predicted heart rate for age and any limitations related to the underlying heart disease.

Patients with pacing > 93% have a 44% reduction in mortality and HF hospitalization rates (combined outcomes), with optimal results being achieved with pacing > 98%. Fusion and pseudofusion beats may overestimate the biventricular pacing percentage. When heart rate control in patients with AF cannot be achieved by ODT, AV node ablation should be performed as it is associated with reduced mortality.^206^Paroxysmal and persistent AF ablation (pulmonary vein isolation) should be considered in patients with HF.^520^A detailed analysis of clinical situations with an indication for ablation is outside the scope of this Guideline.

Ventricular extrasystoles are related to reduced biventricular pacing percentages and reduced reverse remodeling, even with a relatively low incidence. Antiarrhythmics and, eventually, ablation should be considered in patients who do not respond to CRT.^530^

Short AV intervals result in early ventricular systole, preventing the completion of the atrial contraction phase (A-wave truncation). In these cases, the AV interval should be prolonged until the A wave is evident. Conversely, prolonged AV intervals result in E and A wave fusion; in these cases, the AV interval should be shortened.

Two methods are commonly recommended for AV delay optimization: the interactive method and the Ritter method. In the interactive method, a long AV delay (200ms) is programmed and gradually reduced (20ms at a time) to 60ms while the mitral flow is observed. The shortest AV delay capable of maintaining the E and A waves separated (without fusion), without deformation of the A wave, and maintaining a 40ms distance from the end of the A wave to the beginning of the QRS complex is considered the optimal AV delay. Ritter’s method consists of measuring the QA interval (beginning of the QRS complex to the end of the A wave) with two different AV delays, one short (60ms) and one long (200ms). The optimal AV delay is calculated using the following formula: AV delay = long AV delay − (QA[shortAVdelay] − AQ[longAVdelay]). Thus, an echocardiogram with mitral flow assessment should be conducted after implantation to evaluate AV synchrony: if the E and A waves are separated and the interval at the end of the A wave is above 40 ms, there is no need for AV delay optimization.

The interventricular delay may be programmed empirically or optimized by echocardiography, ECG, or specific algorithms. Optimization by echocardiography is performed by testing different delays and assessing dyssynchrony. The delay resulting in the least dyssynchrony should be programmed. Simultaneous pacing, early LV pacing, and different interventricular delays (ie, 60, 40, and 20ms) should be tested. Subsequently, the same delays should be tested with early RV pacing. M mode, with or without tissue Doppler, and LV longitudinal shortening velocity, measured by tissue Doppler, are the most commonly used methods.

As in AV delay optimization, echocardiography-guided interventricular delay programming should be performed under specific conditions in patients who do not respond to CRT.

Some devices have automatic AV- and interventricular-delay optimization algorithms, whose effectiveness remains controversial but does not seem to be inferior to that of empirical or echocardiography-guided optimization.

A correlation between decreased QRS duration with biventricular pacing and the rate of CRT responders has been observed in retrospective studies, supporting the hypothesis that AV- and interventricular-delay optimization aiming at a shorter QRS may increase the response rate to CRT.^531^

Multipoint pacing consists of stimulating regions (especially basal or apical) with delayed LV activation with a quadripolar lead, allowing capture of greater ventricular mass in a faster and more homogeneous way. The MultiPoint Pacing (MPP) trial compared multipoint pacing with a quadripolar lead vs conventional pacing. The results were similar in both groups, with a statistically significant value for noninferiority. However, there was a lower rate of nonresponders among patients with multipoint pacing programmed with an LV lead distance ≥ 30mm and the shortest delay (5ms). These results were reproduced in the first phase of the More Response on Cardiac Resynchronization Therapy With MultiPoint Pacing (MORE-CRT MPP) study. Patients programmed with an LV lead distance ≥ 30mm combined with the shortest intraventricular and interventricular delays also had better results.

## 6.3. Implantable Cardioverter-defibrillator

ICD programming should follow four basic principles: 1) reduce mortality by effectively reversing potentially fatal ventricular arrhythmias; 2) prioritize reversing ventricular arrhythmias with ATP whenever possible; 3) avoid inappropriate shocks; and 4) reduce RV pacing percentage as much as possible (antibradycardia pacing).

Appropriate therapies for sustained VF and VT termination are the cornerstone for mortality reduction in ICD intervention. To this end, tiered therapies should be programmed in different zones, which are classified into VT (one or two zones) and VF zones. Programmable therapies include shocks (up to 35 or 40 J), as well as pacing therapies with 3 to 20 pulses and a pacing rate faster than that of tachycardia (ATP) that can terminate monomorphic VTs without painful shocks, reducing shock-related myocardial damage.

The effectiveness of shock delivery in ventricular arrhythmia termination used to be evaluated intraoperatively by defibrillation threshold testing (VF induction followed by shock delivery for effective circulation reversal, with an output of at least 10J below the maximum programmable output). Subsequent studies have demonstrated that this strategy is unnecessary, since standard intraoperative measurements (pacing threshold, impedance, and R wave) are sufficient for effective termination of spontaneous arrhythmias.^532^Thus, certain complications due to intraoperative VF induction and shock-related myocardial damage can be avoided. VF zones should therefore be programmed with reversed polarity shocks with the highest possible energy. Conversely, VT zones may be programmed with lower-energy shocks, which are usually preceded by an ATP attempt.^533^

The effectiveness of ATP as first-line treatment for VTs is well known. Monomorphic, organized VTs with stable cycles and no hemodynamic repercussions can be easily terminated by both burst ATP (with a fixed interpulse interval) and ramp ATP (with an automatically decreasing interpulse interval).

Some unstable arrhythmias, even in high-rate zones (in the VF range), may be terminated by ATP before the delivery of programmed shocks. In these cases, an ATP attempt should be programmed during or before shock energy load; if the arrhythmia is terminated, the shock should be aborted. The Pacing Fast VT Reduces Shock Therapies II (PainFREE II) study used ATP as first-line treatment in a fast VT zone (188 to 250bpm) and reported a significant relative reduction in the risk of shock of 71%, without compromising patient safety.^534^

Adequate programming of detection and tiered therapy reduces inappropriate shocks, promotes higher rates of appropriate ATP, and reduces mortality.^535^To this end, basic programming principles should include:

1. A VF zone programmed with a rate > 233bpm (> 188 for Medtronic devices), with at least 30 beats out of 40 (x in y) for detection. This strategy avoids shocks in nonsustained arrhythmias and inappropriate shocks due to intermittent noise, double counting, or extrasystoles.
2. In primary prevention patients, a single VF detection zone may be sufficient. VT monitoring zones without therapies (monitoring) may be programmed at the physician’s discretion. In secondary prevention patients, VT-targeted therapies should be programmed with a detection cutoff of 10-20bpm lower than the documented tachycardia rate. Based on clinical criteria, low-rate therapy zones may be programmed according to the risk of slower VT, but ATP should always be prioritized.^536^
3. Noise and lead integrity monitoring algorithms should be programmed, as well as automatic adjustment and oversensing prevention features, such as T-wave detection.
4. Adequate programming of supraventricular arrhythmia discrimination algorithms, particularly in the morphology discrimination criterion (single-chamber devices) and in the evaluation of algorithms based on the atrioventricular relationship (dual-chamber devices). Timers, such as the Sustained Rate Duration (Boston Scientific) and Timeout, should be deactivated, as they ignore the discrimination of events classified as SVT after the preestablished period and deliver inappropriate therapies.

Finally, the need for concomitant antibradycardia pacing must be carefully evaluated. Most patients with an ICD do not require antibradycardia therapy, especially for primary prevention. However, conventional RV pacing is known to increase the risk of ventricular dysfunction and mortality. ICD programming should, whenever possible, prioritize RV pacing percentage reduction. To this end, single-chamber ICDs should be programmed to VVI mode with 40 ppm, whereas dual-chamber ICDs should prioritize atrial pacing alone by using ventricular pacing minimization algorithms (eg, RYTHMIQ, MVP, IRS plus) or by programming an AV interval long enough to avoid unnecessary ventricular pacing. In patients requiring ventricular pacing due to AV conduction block, the possibility of pacing alternative sites, such as biventricular pacing (CRT) or conduction system pacing (His bundle/LBB), should be considered according to ventricular function.^537^

## 6.4. Implantable Loop Recorder

Implantable loop recorders should be adequately programmed to detect ventricular electrical activity without undersensing and without noise oversensing, which could impair rhythm identification.

The automatic detection function, regardless of manual activation by the patient, a memory with electrogram storage capacity, and a battery life of 3 to 4 years help to identify arrhythmic events that have not been documented by conventional tests.

The implantation technique is similar for all available models. The adequacy of electrical signal at the position chosen for device placement should be confirmed intraoperatively. After confirmation of adequate signal capture, the implantable loop recorder should be programmed to detect arrhythmias on an individual basis.^538^Some manufacturers suggest an initial empirical programming as follows:

- Pauses: 3 s
- Bradycardia: heart rate ≤ 30bpm for more than 4 consecutive beats
- Tachycardia: heart rate > maximum predicted heart rate for age (220 - age) for 16 beats or more
- Atrial fibrillation: episodes > 2 minutes are characterized as AF rhythm

Careful adjudication of the episodes recorded by the implantable loop recorder is required, as false detections may have been stored. Episodes classified as AF, for example, may be misclassified due to RR interval variations resulting from ventricular extrasystoles or intermittent QRS undersensing. An analysis of 695 spontaneous or scheduled transmissions revealed a false-positive transmission rate of up to 81%.^539^Adequate programming of the detection criterion and subsequent analysis of tracings is essential for optimal monitoring.

## 6.5. Remote Monitoring (Online)

Online remote monitoring is a reality for patients with CIEDs. Data from the device can be transmitted via a broadband Internet connection. Newer devices allow data transmission via a Bluetooth connection in a smartphone. Remote monitoring allows access to several programming features, such as pacing rate and mode, pacing output, sensing and detection algorithms, as well as to diagnostic records and battery status.

Data transmission needs to be adjusted because information may be automatically transmitted by the device after a trigger or manually transmitted by the patient. A scheduled transmission may also be programmed. Remote access to device information is given to the service responsible for monitoring the patient through private access to the system’s server, provided that patients’ data privacy is protected.

## 7. Recommendations for Prevention and Treatment of CIED infecTions and for System Removal

### 7.1. Prevention and Treatment of Infections

Recent publications have shown an increased incidence of CIED-related infectious processes. Demographic and clinical factors, such as population aging and comorbidities, may influence both hematogenous seeding and direct contamination from device implantation and replacement. A recent EHRA survey reported that CIED infections are more frequent after reoperations, including those for isolated pulse generator replacement.^540^

The most recent consensus statements have emphasized the need for standardizing management strategies and forming expert teams to address this particular and uncommon type of infection with the aim of mitigating the still frequent controversies over the topic.^541 - 546^

CIED-related infectious processes manifest as either involvement of the pulse generator pocket or exclusive intravascular involvement. Exclusive pocket involvement is more frequent and accounts for approximately 60% of cases (generally resulting from contamination during surgery or subsequent manipulation). Late skin erosion may be due to or result in pocket infection; in both cases, this may progress to a systemic infection. Pocket involvement associated with intravascular infection accounts for approximately 20% of infections and is usually secondary to delayed or inadequate management. Exclusive intravascular involvement also represents approximately 20% of cases and results from bloodstream contamination in most cases.^547^Such contamination may occur during bacteremia caused by a distant infectious focus, such as septic thrombophlebitis, osteomyelitis, pneumonia, surgical site infection, contaminated vascular catheters, or bacterial infection originating from the skin, mouth, or gastrointestinal or urinary tract.

An expert consensus statement issued by the EHRA and endorsed by other international societies aimed to define the terminology that should be used in clinical studies and registries for the therapeutic approach to CIED infections and for system removal.^548^ Table 39 shows the terminology recommended for different clinical presentations.

**Table 39:** – CIED-related infection types

Infection types		
	Clinical scenarios	Definition
Local	Superficial incisional infection	Involves only skin and subcutaneous tissue, not the CIED
	Isolated pocket infection	Presence of clinical signs of inflammation limited to the pulse generator pocket (erythema, warmth, fluctuance, wound dehiscence, tenderness, or purulent drainage) with negative blood cultures
	Isolated pocket erosion	Complete or partial extrusion of pulse generator or lead through the skin
Systemic	Bacteremia	Positive blood cultures with or without systemic infection symptoms and signs
	Pocket infection or erosion with associated bacteremia	Generator pocket infection or erosion with positive blood cultures, without lead or valvular vegetation(s)
	CIED-related endocarditis without pocket infection	Bacteremia and lead or valvular vegetation(s) without pocket infection
	Pocket infection with lead/valvular endocarditis	Bacteremia and lead or valvular vegetation(s) with pocket infection
	Occult bacteremia with probable CIED infection	Bacteremia without an obvious source other than the CIED

*CIED: cardiac implantable electronic device.*

**Table 40:** – Modified criteria for diagnosis of CIED infections

Definite generator pocket infection	Presence of one of the following findings: 1) edema, erythema, warmth, pain, and purulent discharge; 2) fistula formation; 3) deformation, adherence, or threatened erosion of the skin; 4) generator or lead exposure
Definite CIED-related endocarditis	Presence of 1) 2 major criteria; 2) 1 major and 3 minor criteria
Possible CIED-related endocarditis	Presence of 1) 1 major and 1 minor criteria; 2) 3 minor criteria
Rejected ICED-related endocarditis	Absence of the aforementioned criteria

*CIED: cardiac implantable electronic device.*

**Table 41:** – Major and minor criteria for diagnosis of CIED infections

MAJOR CRITERIA	
Microbiology	A. Blood cultures positive for typical microorganisms found in CIED infection and/or IE (coagulase-negative staphylococci, *S. aureus* )
	B. Microorganisms consistent with IE from 2 separate blood cultures:
	a. *Streptococcus viridans, Streptococcus gallolyticus* ( *S. bovis* ), HACEK group, *S. aureus*
	b. Community-acquired enterococci, in the absence of a primary focus
	C. Microorganisms consistent with IE from persistently positive blood cultures:
	a. ≥ 2 positive blood cultures of blood samples drawn > 12h apart
	b. All of 3 or a majority of ≥ 4 separate blood cultures (first and last samples drawn ≥ 1 h apart)
	c. Single positive blood culture for *Coxiella burnetii* or phase I IgG antibody titer > 1: 800
Imaging positive for CIED infections and/or IE	A. Echocardiogram (including intracardiac) positive for:
	a. CIED infection:
	I. Clinical pocket/generator infection
	II. Lead vegetation
	b. Valve IE
	I. Vegetations
	II. Abscess, pseudoaneurysm, intracardiac fistula
	III. Valvular perforation or aneurysm
	IV. New partial dehiscence of prosthetic valve
	B. [^18^F]FDG PET/CT (caution should be taken in case of recent implants) or radiolabeled WBC SPECT/CT detection of abnormal activity at pocket/generator site, along leads, or at valve site
	C. Definite paravalvular leakage by cardiac CT
**MINOR CRITERIA**	
	a. Predisposition such as predisposing heart condition (eg, preexisting structural heart defects, new-onset tricuspid valve regurgitation) or injection drug use
	b. Fever (temperature > 38°C)
	c. Vascular phenomena (including those detected only by imaging): major arterial emboli, septic pulmonary embolisms, infectious (mycotic) aneurysm, intracranial hemorrhage, conjunctival hemorrhages, and Janeway lesions
	d. Microbiological evidence: positive blood culture which does not meet a major criterion as noted above or serological evidence of active infection with organism consistent with IE or pocket culture or lead culture (extracted by noninfected pocket).

*CIED: cardiac implantable electronic device; IE: infective endocarditis; PET/CT: positron emission tomography/computed tomography; WBC SPECT/CT: white blood cell single-photon emission tomography/computed tomography; Janeway lesions: painless hemorrhagic lesions due to septic emboli.*

A definite diagnosis of CIED infection is based on three major findings: 1) presence of purulent drainage or CIED exposure on clinical examination; 2) growth of microorganisms in blood cultures, and 3) presence of tricuspid valve or lead vegetations seen on transesophageal echocardiogram (TEE). When the diagnosis of CIED infection cannot be defined using these criteria, additional tests (eg, PET-CT) may be needed. The modified Duke University criteria for diagnosing CIED infections are listed in Tables 40 and 41.

Proof that the CIED is definitely contaminated is essential for proper treatment because, once contamination is confirmed, complete device removal will be necessary for a successful outcome. Conversely, if the CIED is free from contamination and the infectious process is related to a different focus, unnecessary device removal will imply avoidable cost and surgical risk related to lead extraction. A flowchart for diagnosing and treating CIED infections is shown in Figure 5 .

**Figure 5:** – Flowchart for diagnosis and treatment of CIED infections. CIED: cardiac implantable electronic device; FDG: fluorodeoxyglucose; PET/CT: positron emission tomography/computed tomography; TEE: transesophageal echocardiogram; TTE: transthoracic echocardiogram; WBC SPECT/CT: white blood cell single-photon emission tomography/computed tomography.

Imaging tests are useful for both diagnosis and treatment. Thus, some imaging information may be relevant, including 1) identification of CIED type; 2) identification of abandoned leads; 3) findings of intracardiac vegetations and their size; and 4) signs suggestive of septic pulmonary embolism.

In cases of fever in which a CIED pocket evaluation is unable to determine the infection, blood cultures or TEE and radiopharmaceutical-based imaging tests may be relevant.

Although complete removal of the pulse generator and all leads is essential, the infection must be treated with antimicrobial therapy. Antibiotic choice should be based on blood cultures and removed pocket/lead fragment cultures. When the microorganism cannot be identified, empirical antibiotic use should be defined by clinical criteria. Likewise, treatment duration should also be defined according to the clinical status, and the starting point should always be complete CIED removal ( Figure 6 ).

**Figure 6:** – Treatment duration and need for system removal in different presentations of CIED infection. CIED: cardiac implantable electronic device.

Complete CIED removal is critical to preventing recurrent infections. Lead extraction, however, should rarely be considered an emergency, even in cases of septic shock. With the exception of recent implants, which tend to be technically easier, extraction should only be performed when the hemodynamic status and the infection have stabilized, given the risks associated with the procedure (vein and heart adhesions).

Transvenous lead extraction should be the preferred technique, except when leads are epicardial or when intracavitary vegetations are greater than 2.5cm (largest diameter). Recommendations for removing the pulse generator and leads are listed in Table 42 .

**Table 42:** – Recommendations for CIED removal

	Class of recommendation	Level of evidence
Complete system removal in patients with definite CIED infection (systemic or local)	I	B
Complete CIED removal in patients with bacteremia with S. aureus, CoNS, Cutibacterium spp., or fungemia with Candida spp., after other infection sites are ruled out	I	C
Complete CIED removal in patients with bacteremia with Pneumococcus spp. or nonpseudomonal/Serratia gram-negative bacteria, in the presence of recurrent/persistent bacteremia despite appropriate antibiotic therapy when there is no other identifiable source for recurrence or persistent infection	I	C
Complete CIED removal in patients with infective endocarditis with or without definite involvement of the CIED system	IIa	C
Complete CIED removal in patients with bacteremia with alpha- or beta-hemolytic Streptococcus spp. or Enterococcus spp. performed as first-line treatment or, in case of recurrent/persistent bacteremia despite appropriate antibiotic therapy, as second-line treatment	IIb	C

*CIED: cardiac implantable electronic device.*

Implantation of a new CIED should only be performed after complete remission of the infectious process, based on the clinical status. Until the infection has completely resolved, patients dependent on artificial pacing should maintain treatment with a temporary PM. Nondependent patients should remain under cardiac rhythm monitoring until the implant is performed. In some cases, implantation of a new CIED may not be necessary because of a change in the disease pattern or a revision in the management strategy. Therefore, reassessing the need for a CIED is always essential. Recommendations for implantation of a new CIED are listed in Table 43 .

**Table 43:** – Recommendations for implantation of a new CIED

	Class of recommendation	Level of evidence
Reassessment of the indication for reimplantation after device extraction	I	C
Preferred access sites for implantation of a new device are the contralateral side, the femoral vein, or an epicardial approach	I	C
Whenever possible, reimplantation should be avoided or delayed until symptoms and signs of systemic and local infection have resolved	IIa	C
A temporary pacemaker with ipsilateral active fixation strategy in pacemaker-dependent patients as they wait for reimplantation	IIa	C

*CIED: cardiac implantable electronic device.*

Several risk factors for the development of CIED infection have been reported. These factors may be related to the patient, the medical procedures performed, or the CIED itself. Major factors for CIED infection are listed in Table 44 .

**Table 44:** – Risk factors predisposing patients to CIED infection

Patient-related factors
End-stage renal disease^a^
History of device infection
Fever prior to implantation
Corticosteroid use
Renal insufficiency^b^
Chronic obstructive pulmonary disease
NYHA class ≥II
Skin disorders
Malignancy
Diabetes mellitus
Heparin bridging
Heart failure
Oral anticoagulants
**Procedure-related factors**
Procedure duration
Hematoma
Lead repositioning
Inexperienced operator^c^
Temporary pacing
Device replacement/revision/upgrade
Generator change
Antibiotic prophylaxis^d^
**Device-related factors**
Epicardial leads
Abdominal pocket
≥ 2 leads
Dual-chamber device

*NYHA: New York Heart Association. Risk parameters which were statistically significant for retrospective and prospective data are shown. Analyses restricted to prospective data only for the same parameters (if available) are also shown. Adapted from Polyzos et al.549 aGlomerular filtration rate (GFR) < 60mL/min or creatinine clearance (CrCL) < 60mL/min. bGFR ≤15 mL/min or hemodialysis or peritoneal dialysis. c <100 previous procedures. dThe pooled effect estimate from randomized studies was 0.26 [0.13, 0.52].*

Preventive care is crucial to reducing the occurrence of procedure-related infections. Table 45 and Figure 7 show the most commonly recommended preventive measures.

**Table 45:** – List of recommended preventive measures for CIED infections

	Class of recommendation	Level of evidence
**Preprocedural measures**		
Delay CIED implantation in patients with infection	I	C
Avoid temporary transvenous pacing and central venous access whenever possible. If used, they should ideally be removed prior to introducing a new device	I	A
Measures to avoid pocket hematoma: discontinue antiplatelet agents whenever possible and, in the case of oral anticoagulants, avoid heparin bridging, discontinuing the use during implantation if possible	I	B
Antibiotic prophylaxis is recommended within 30min to 1h of incision for cefazolin and within 90-120min for vancomycin	I	A
**Periprocedural measures**		
Use of antibiotic envelope in high-risk situations for infection*	IIa	B
Instillation of antiseptic and/or antibiotics in the generator pocket	IIb	C
Postprocedural measures		
Use of postoperative antibiotic therapy	IIb	B
Hematoma drainage or evacuation (except in the presence of tense tissue, wound dehiscence, or severe pain)	III	B

* As defined in the WRAP-IT study:550 patients undergoing pocket or lead revision, generator replacement, system upgrade, or initial CRT-D implantation, and those with risk factors as listed in Table 44. CIED: cardiac implantable electronic device.

**Table 46:** – Definitions for extraction approaches and techniques

Technique	Definition
**Transvenous**	Intravascular (percutaneous) lead extraction performed through a central vein (subclavian, jugular, or femoral)
- Venous entry site approach	Lead extraction by traction or countertraction using the same vein and site through which the lead was introduced
- Transjugular or transfemoral approach	Extraction with an intravascular tool introduced through right or left jugular or femoral veins
**Transthoracic**	Lead extraction by opening the thoracic cavity using median sternotomy, lateral thoracotomy, or subxiphoid incision, including a hybrid approach, with or without cardiopulmonary bypass

**Figure 7:** – Preventive measures for reducing CIED infections considering modifiable and nonmodifiable risk factors as determined in the literature. CIED: cardiac implantable electronic device; COPD: chronic obstructive pulmonary disease; CRT: cardiac resynchronization therapy; ICD: implantable cardioverter-defibrillator; NYHA: New York Heart Association; OAC: oral anticoagulation.

Older people, children, and adults with congenital heart disease constitute subgroups that deserve special attention regarding CIED infections. Submuscular positioning of pocket in patients with limited subcutaneous tissue is essential to preventing skin erosion. In pediatric patients, especially those with congenital heart disease, the operator should be experienced in multiple and alternative surgical approaches. Extravascular or subcutaneous ICD implantation should be considered in younger children, patients with congenital heart disease, and those with limited or no venous access.^551 , 552^

Interestingly, retrospective registries^553 , 554^have reported higher CIED infection rates than those of prospective studies^537 , 555 - 558^(3.4% vs 1.2%). This phenomenon may reflect greater adherence to preventive procedures in clinical studies compared to daily clinical practice. The most important procedure-related factors for risk of infection include pocket hematoma, long procedure duration, and reinterventions for lead repositioning. Regarding reoperations for pulse generator replacement, lead dysfunction correction, or pacing mode change, appropriate management of the pulse generator pocket, either by complete removal of the fibrotic capsule or by use of antibacterial envelopes, reduces the number of infections.

Preoperative antibiotic prophylaxis with a single dose of first-generation cephalosporins (cefazolin) is strongly recommended, which is not the case with systematic postoperative antibiotic use.^11^

The time interval between diagnosis and appropriate treatment of CIED infections is critical. Literature data show that if the device is removed within 3 days of hospitalization, both length of stay and in-hospital mortality are significantly reduced. Thus, when there are no sufficient data to establish the diagnosis of infection based on the triad of infectious signs in the pulse generator pocket, bacterial growth in blood cultures, and vegetations on TEE, additional resources should be employed.

[^18^F]FDG PET/CT scanning or radiolabeled WBC scintigraphy or contrast-enhanced CT are recommended in cases of suspected CIED-related infective endocarditis, positive blood cultures, and negative echocardiogram, or in patients with *S. aureus* bacteremia in the presence of CIED. Needle aspiration and surgical debridement in cases of generator pocket infection, in an attempt to avoid lead extraction, should be strongly discouraged.

Large, nonvoluntary, easy-to-fill, high-quality registries are essential to monitoring the number of cases of CIED infection and the outcomes of preventive and therapeutic measures. Each center should establish routines for accurate diagnosis and timely treatment. Constant reassessment of the performance of each center is highly recommended.

### 7.2. Lead Removal from Cardiac Implantable Electronic Devices

There has been a growing demand for CIED lead removal in recent years because of two major factors: 1) increase in CIED infection rates and 2) development of multisite PM and ICD systems with a higher number of leads.

Indications for lead removal may be (a) mandatory for treating infections; (b) necessary for obtaining access for new leads in patients with venous occlusions; or (c) optional for performing lead replacement in patients with appropriate venous access.

As most devices require venous access for lead implantation, transvenous extraction techniques are most commonly used. Currently, open-chest lead removal is rarely performed as few scenarios require this method, such as removal of epicardial leads or correction of complications occurring in transvenous extraction procedures.

The current panorama of lead extraction shows well-established indications and operative techniques. The tools used are well developed, and the outcomes of different procedures are well known, with high success rates. Catastrophic complications, however, may occur during extraction procedures. Such complications, although rare, are potentially lethal and often require emergency open surgery.

This item presents the recommendations for extraction in noninfected patients, as the management of infections has been previously addressed.

Epicardial lead removal is necessarily performed by reopening the thoracic cavity preferably using the same access through which the lead was implanted. Transvenous lead removal should preferably be done by intravascular access. Exceptionally, a transthoracic approach may be used with or without cardiopulmonary bypass (eg, failure of transvenous extraction or presence of large lead vegetations). The choice of transvenous lead extraction approach depends essentially on the possibility of obtaining access to the lead targeted for removal. Unfortunately, many patients have entirely intravascular leads because of spontaneous lead fractures or iatrogenic events during removal procedures.

When the lead to be removed is intact or has an extravascular segment, however small this segment may be, the venous entry site approach must be used. This approach consists of introducing a sheath into the vein which is guided by the lead to be removed. This sheath is used to cut through adhesions that form between the lead and the venous endothelium or the endocardium. When the patient has more than one transvenous lead implanted, adhesions are often seen between the leads. After all adhesions have been removed and the site where the lead is attached to the heart has been reached, the same sheath is used to apply pressure against the heart muscle while pulling the lead (countertraction maneuver). There are several tools specifically developed for this type of approach, namely:

Locking stylets, which are stylets coated with a fine steel mesh that expands in the light of the lead, providing the lead with the necessary support for its traction.
Sheaths for adhesion dissection and countertraction:
*Nonpowered sheaths* are sets of rigid metal and flexible Teflon or polypropylene tubes that cut through adhesions by blunt dissection, with an intensity level determined by the strength of the operator’s hand.
*Rotational mechanical sheaths* are activated by a trigger in the operator’s hand or by an electric motor and cut through adhesions.
*Laser sheaths* use photoablation to cut through adhesions.

When the lead that needs to be removed does not have an extravascular segment, intravascular extraction is mandatory. There are tools in the shape of a loop or basket made with very malleable metal wires and designed to grasp these fragments. Such tools are usually introduced by puncturing the femoral or jugular veins. Once grasped, the lead can be pulled directly. Specific cases may require a combined countertraction maneuver after grasping the lead.^559^Tables 46 and 47 show recommended definitions for tools and approaches used in lead extraction.

The term *removal* has been generically used to refer to CIED lead removal regardless of the type of approach. It can be performed by simple traction of the transvenous lead without using any tools; by thoracotomy for removing epicardial leads; or by thoracotomy with cardiopulmonary bypass for removing transvenous leads. The term *extraction* should be reserved for cases requiring the use of techniques and tools to 1) dilate the venous path through which the lead passes; 2) cut through adhesions; 3) perform a countertraction maneuver; or 4) grasp lead fragments inside the heart vessels or chambers.

Completion of a lead removal or lead extraction procedure may result in 1) complete removal of the targeted lead; 2) partial removal; or 3) unsuccessful removal. Depending on the type of indication for lead removal, the procedure may be considered clinically successful even if the lead was not entirely removed. The procedure is considered unsuccessful when 1) clinical success is not achieved; 2) any permanently disabling complication occurs; or 3) the patient dies. Table 48 lists the definitions established by the 2018 EHRA consensus statement and endorsed by other representative societies.

**Table 47:** – Definitions for extraction tools

Tools and techniques	Definition
**Sheath for dissection and countertraction**	
- Mechanical nonpowered	Sets of rigid metal and flexible Teflon or polypropylene tubes
- Rotational mechanical ^548^	Metal-cutting blades driven by manual or electrical activation
- Laser ^560^	Glass microtubes conducive of the light produced by an (external) emission source of the Excimer laser
**Locking stylet**	Stylets coated with a steel mesh that provide the leads with rigidity and support
**Intravascular extraction tools**	Snares, ^561^ baskets, and other tools used to grasp intravascular fragments
**Vascular occlusion balloons** ^**562**^	Sets of guidewires and balloons for venous dilation or occlusion
**Accessory tools**	Lead extenders, compression coils ^563^

**Table 48:** – Definitions for lead removal procedures and outcomes

Concept	Definition
**Procedure**	
Lead removal	Lead removal using simple traction techniques and no specialized tools
Lead extraction	Removal of at least one lead using specialized tools (listed in Table 47)
**Outcome**	
**Lead-related**	
- Complete lead removal	Lead explant or extraction with removal of all lead material
- Incomplete lead removal	Lead explant or extraction in which part of the lead remains in the patient’s body
**Patient-related**	
- Complete procedural success	Removal of all targeted leads, with no permanently disabling complication or procedure-related death
- Clinical procedural success	Retention of a small portion of a lead that does not negatively impact the outcome of the procedure. This may be the tip or a small part (< 4cm) of the lead (conductor coil, insulation, or both) provided that the fragment does not increase the risk of perforation, embolic events, or perpetuation of infection, in the absence of any permanently disabling complication or procedure-related death
- Procedural failure	Inability to achieve clinical success, or the development of any permanently disabling complication or procedure-related death

Leads may be deactivated for several reasons, such as 1) loss of ability to adequately stimulate the heart, 2) need to change the device type, and 3) manufacturing-related problems.

Noninfected leads may be abandoned in situ at the discretion of the surgical team. There are, however, disadvantages to abandoning a lead, including 1) risk of thrombotic phenomena, 2) limitations to MRI scans, and 3) increased risk in a future extraction procedure, since the longer the lead remains, the higher the risk of unsuccessful extraction. The main argument for abandoning a noninfected lead in situ is the risk of severe complications associated with the extraction procedure. A recent publication shows that 1-year expected survival following a lead replacement procedure is similar for cases of lead extraction vs in situ abandonment.^564^Therefore, the decision to extract or not a lead that will be deactivated essentially depends on the expertise of each center. Table 49 shows the EHRA 2018 consensus definitions for noninfected leads.

**Table 49:** – Definitions of terms for noninfected leads

Noninfected leads	Definitions
Lead function	Any lead function, including pacing, sensing, and/or defibrillation
Lead failure	Loss of any lead function
Nonfunctional lead	Lead not usable for pacing and/or defibrillation due to loss of functional integrity
Abandoned lead	Lead left in place in the heart and not connected to a CIED. It may be functional or nonfunctional
Recall^558,565-568^	Removal or correction of a lead due to manufacturing-related problems, regulatory agency requirements, or voluntary manufacturer guidance
- Class I	Dangerous or defective products with reasonable probability of causing serious health problems or death (eg, short circuit without warning)
- Class II	Products that might cause a temporary health problem or pose a slight threat of a serious nature (eg, premature battery depletion)
- Class III	Products that are unlikely to cause any adverse health reaction but that violate regulatory standards

*CIED: cardiac implantable electronic device.*

CIED durability depends on both manufacturing (material and/or design) and usage aspects. Leads are directly influenced by the operative technique used, which may negatively impact their durability. Nonetheless, specific surveillance strategies must be adopted by manufacturers and regulatory agencies to assess the durability of CIED components.

In cases of in situ lead abandonment due to a dysfunction or need to change the pacing mode, or, inappropriately, in cases of treatment of a CIED infection, caution is required because how leads are abandoned may hinder future extraction procedures. Recommendations for lead abandonment are listed in Table 50 .

**Table 50:** – Recommendations for managing abandoned, unnecessary, or dysfunctional leads

	Class of recommendation	Level of evidence
When a lead is abandoned, it should be left in a condition that prevents retraction inside the vein and that allows extraction in the future	I	C
In cases of clinically unnecessary or dysfunctional leads, both options (abandonment and extraction) can be considered within the surgical strategy^569-571^	IIa	B

Noninfected lead removal may be mandatory in certain clinical situations, such as for 1) treatment of superior vena cava syndrome caused by the presence of leads; 2) treatment of severe cardiac arrhythmia mechanically caused by a lead fragment; 3) prevention of cardiac injury from a fractured lead; or 4) radiation therapy in the region where the device is implanted.

Also, lead extraction may be necessary in cases of severe venous occlusion or obstruction preventing the passage of a new lead.

In many cases, however, lead extraction is optional and can be defined by evaluation of less objective factors, such as 1) patient’s age or estimated life expectancy; 2) future need for MRI scans; 3) risk of developing severe venous obstructions; or 4) risk of infection via a hematogenous route (eg, patients with renal failure on dialysis). Such situations require the expertise of the professional who performs the procedure for defining an appropriate management strategy. Table 51 lists the recommendations for removal of noninfected leads.

**Table 51:** – Recommendations for lead removal in noninfectious situations

	Class of recommendation	Level of evidence
**Thrombosis/Vascular involvement**		
Clinically significant embolic events attributable to the presence of a lead or lead fragment^572-574^	I	C
SVC stenosis or occlusion^575^preventing the passage of a new lead	I	C
Need for stenting to avoid lead entrapment^576,577^	I	C
**Others**		
Lead- or fragment-induced arrhythmias^578^	I	C
Device implanted in a site that interferes with the treatment of a malignant disease^57^9	IIa	C
Lead overpopulation: > 4 leads on one side or > 5 leads across the SVC^569,580,581^	IIa	C
Abandoned leads interfering with the operation of an implanted device^582,583^	IIa	C
Leads that due to their design or failure pose a threat or harm to the patient if left in place^584-587^	IIb	C
Contraindications to magnetic resonance imaging: abandoned leads or fragments; nonconditional leads for magnetic resonance imaging^588-593^	IIb	C
Permanent device removal by shared decision	IIb	C

*SVC: superior vena cava.*

During transvenous lead extraction procedures, veins or cardiac structures may be injured. Injuries to the axillary or subclavian veins, brachiocephalic veins, or superior vena cava may cause severe hemorrhage requiring blood transfusion or even surgical correction. Muscle avulsion of the right atrium or RV and perforation of coronary sinus tributaries may lead to cardiac tamponade. Extrapericardial laceration of the superior vena cava, however, is the most frequent and most lethal catastrophic complication. Other complications, such as self-limiting cardiac arrhythmias, pneumothorax, or lead fragment retention, may also occur and require specific care.

Complications are generally grouped into major and minor according to severity and type of correction required. Table 52 lists complications according to their classification and incidence.^577^

**Table 52:** – Classification and incidence of the most frequent perioperative complications*

Complications	Incidence, %
**Major**	**0.19–1.80**
Death	0.19–1.20
Cardiac avulsion	0.19–0.96
Vascular laceration	0.16–0.41
Respiratory arrest	0.20
Cerebrovascular accident	0.07–0.08
Pericardial effusion requiring intervention	0.23–0.59
Hemothorax requiring intervention	0.07–0.20
Cardiac arrest	0.07
Thromboembolism requiring intervention	0.07
Flail tricuspid valve leaflet requiring intervention	0.03
Massive pulmonary embolism	0.08
**Minor**	**0.06–6.20**
Pericardial effusion without intervention	0.07–0.16
Hematoma requiring evacuation	0.90–1.60
Venous thrombosis requiring medical intervention	0.10–0.21
Vascular repair at venous entry site	0.07–0.13
Migrated lead fragment without sequelae	0.20
Bleeding requiring blood transfusion	0.08–1.00
Arteriovenous fistula requiring intervention	0.16
Pneumothorax requiring chest tube	1.10
Worsening tricuspid valve function	0.02–0.59
Pulmonary embolism	0.24–0.59

* Source: adapted from Kusumoto et al., 2017.^594^

Several studies have been designed to identify risk factors that determine morbidity and mortality in transvenous lead extraction procedures. These studies have reported low rates of catastrophic complications and perioperative death, not allowing proper identification of those risk factors. Conversely, several demographic, clinical, and surgical factors are associated with 30-day mortality following an extraction procedure. Factors associated with late complications and death have also been described.^30 , 595 - 603^ Table 53 lists the identified risk factors and their impact on morbidity and mortality.

**Table 53:** – Risk factors for death and complications associated with lead removal

Factor	Risk associated with factor
Age	Increases mortality by 1.05 times
Female sex	Increases risk of major complications by 4.5 times
Low body mass index (<25 kg/m^2^)	Increases 30-day mortality by 1.8 times Increases number of extraction-related complications
Stroke history	Increases risk of major complications by 2.0 times
Severe LV dysfunction	Increases risk of major complications by 2.0 times
Advanced HF	Increases 30-day mortality risk by 1.3 to 8.5 times Increases 1-year mortality by 3.0 times
Renal dysfunction	End-stage renal disease increases 30-day risk of death by 4.8 times Creatinine > 2.0 increases in-hospital mortality and 1-year mortality by 2.0 times
Diabetes mellitus	Increases in-hospital mortality and overall mortality by 1.7 times
Coagulopathy	High INR increases risk of major complications by 2.7 times and 30-day mortality by 1.3 times Anticoagulants increase 1-year mortality by 1.8 times
Platelet count	Thrombocytopenia increases risk of major complications by 1.7 times
Anemia	Increases 30-day risk of death by 3.3 times
Number of extracted leads	Increases risk of any complications by 3.5 times Increases long-term mortality by 1.6 times
Presence of dual-coil ICD	Increases 30-day mortality by 2.7 times
Extraction for infection	Increases 30-day mortality by 2.7 to 30 times Increases 1-year mortality by 5.0 to 9.7 times CRP > 7.2mg/L increases 30-day mortality Increases overall mortality by 3.5 times
Operator’s experience	Increases number of procedure-related complications by 2.6 times
Previous heart surgery	Reduces incidence of major complications

*CRP: C-reactive protein; ICD: implantable cardioverter-defibrillator; INR: international normalized ratio; LV: left ventricle; HF: heart failure.*

Given the difficulty of predicting perioperative catastrophic complications, prevention of associated deaths becomes crucial, which implies training the staff and providing centers with the technical skills required for lead extraction. A recent systematic review^604^shows the close relationship between the volume of procedures performed at the center and the rate of complications associated with lead extraction. Naive operators should be supervised by more experienced operators during the first 40 transvenous extraction procedures. A minimum volume of 20 transvenous extraction procedures per year is recommended for all operators to maintain their technical skills.

## 8. Recommendations for the prevention of electromagnetic interference

### 8.1. Surgery Using Electrocautery

Electrosurgery uses high-frequency alternating current (200kHz to 2.2MHz), which is converted into heat when passing through tissue with sufficient resistance, allowing the desired effects to be achieved: coagulation and cutting. An electric scalpel is used in most surgical specialties.

Monopolar electrosurgery is the most effective and therefore most widely used technique in surgical practice. In this modality, the active electrode is located at the surgical site (on the surgical instrument), whereas the indifferent (return) electrode is a plate placed on the patient’s skin at a distance. The current flows between the electrodes, passing through the body.

An increasing number of patients with CIEDs are treated surgically, which exposes these patients to electromagnetic interference. Monopolar electrosurgery can cause a number of CIED abnormalities, such as pulse generator reprogramming, temporary pacing inhibition, high-frequency pacing triggering, battery depletion and pacing failure, circuit damage, threshold elevation, and triggering of inappropriate therapy (shocks) in the case of ICDs.^605^

To minimize the risks of using electrocautery, some precautions should be taken perioperatively: (1) the monopolar probe should be used intermittently, with short bursts of current and low energy levels; (2) the indifferent plate should be positioned so that the current does not flow through the generator or electrodes.

In general, when the surgical site is located above the umbilicus or at a distance of less than 15 cm from the generator, the use of a monopolar probe should be avoided. In this scenario, a bipolar probe should be preferred as it is safer, but it should not be applied directly to the generator.^606 , 607^In head and neck surgery, the indifferent plate of the monopolar probe should be placed on the posterior shoulder contralateral to the device pocket. For example, if the generator is placed on the left infraclavicular region, the probe plate should be placed on the right shoulder.

In order to protect both patients and CIEDs from the undesirable effects of electrocautery, two approaches have been used: placing a magnet over the pulse generator and reprogramming the device before the procedure. In the case of PMs, magnet use during surgery is an option when circuit sensing has been deactivated by the generator under magnetic effect (asynchronous mode) and the battery is in good condition.^608 , 609^Placing a magnet over the generator pocket causes the PM to revert to asynchronous mode, that is, it disables the sensors and changes to magnet pacing rate, which is often higher than the programmed pacing rate.

In the case of ICDs, a randomized trial compared magnet application versus reprogramming in patients with ICDs undergoing surgery using monopolar electrocautery, at a distance greater than 15 cm from the generator; the authors concluded that both strategies are safe.^610^Magnet application to the ICD pocket only disables tachyarrhythmia therapy, it does not change PM function. In PM-dependent patients with ICDs, the device should be reprogrammed to asynchronous mode before the procedure. CIED reprogramming should be performed immediately before the surgical intervention and reverted to original programming immediately after the end of the procedure ( Table 54 ).

**Table 54:** – Recommendations for the prevention of electromagnetic interference in surgery using electrocautery

	Class of recommendation	Level of evidence
Monopolar electrosurgery can cause several CIED abnormalities, and bipolar electrocautery should be used preferably In patients with ICDs who are not pacemaker-dependent, a magnet can be safely placed over the ICD pocket to disable tachyarrhythmia therapy and avoid inappropriate shocks	I	A
In the case of pacemakers, magnet use during surgery is an option when circuit sensing has been deactivated by the generator under magnetic effect (asynchronous mode) and the battery is in good condition	I	B
In head and neck surgery using a monopolar probe, the indifferent plate should be placed on the posterior shoulder contralateral to the device pocket In pacemaker-dependent patients with ICDs, therapies are deactivated and the device should be reprogrammed to asynchronous mode before the procedure CIED reprogramming should be performed immediately before the surgical intervention and reverted to original programming immediately after the end of the procedure	I	C
When the surgical site is located above the umbilicus, a bipolar probe should be used. If the use of a monopolar probe cannot be avoided, the generator should be reprogrammed	IIa	C

*CIED: cardiac implantable electronic device; ICD: implantable cardioverter-defibrillator.*

### 8.2. Magnetic Resonance Imaging

MRI has emerged as an increasingly useful and accessible diagnostic tool, with growing relevance for diagnostic and prognostic evaluation.

The number of MRI scans has grown substantially over the past 20 years, with over 60 million scans performed worldwide every year. It is estimated that, after CIED implantation, a patient has a 50% to 75% probability of being indicated for an MRI over the lifetime of their device.^594 , 611^

The MRI environment can be divided into zones, as described by Kanal *et al* .^612^and adopted by the 2017 HRS expert consensus.^593^Zone 4 refers to the scanner room, which is the space with the greatest risk for patients and health care staff, including the potential risk of dislodgement of metal objects. Zone 3 is the space outside the scanner room, including the scan control room. Because of the potential risks in this area, access to it must be limited to only trained personnel. Zone 2 includes the reception area, and zone 1 corresponds to areas freely accessible to the general public.

During MRI scanning, CIEDs can be defined as:^613^

*MRI safe* : devices that pose no hazards for MRI scanning.
*MRI conditional* : devices that pose no hazards for MRI scanning, as long as specified conditions of use are met. These conditions include parameters such as: region of the body to be scanned, magnetic field strength, spatial gradient, magnetic field exposure time, radiofrequency field, and specific absorption rate. Additional conditions might be required, including the use of specific generator and electrode combinations as well as device programming mode. Specific conditions may vary among manufacturers and among devices from the same manufacturer.
*MRI nonconditional* : devices that pose hazards for MRI scanning. They include all CIED systems that are nonconditional for MRI scanning, such as MRI-conditional generators combined with nonconditional leads or MRI-conditional systems implanted in patients who do not meet all specified conditions of use, including patients with abandoned leads.

No CIED system is classified as *MRI safe* , and new CIEDs that have been developed with appropriate technology are considered *MRI conditional* for MRI scanning.

In clinical practice, devices need to be programmed before MRI scanning, with a highly acceptable level of safety.

Interactions of the static and gradient magnetic fields with radiofrequency on CIEDs can impair the functioning of electronic components, cause migration or dislodgement of system components, generate energy currents that might damage the device and/or the myocardium, and cause oversensing, undersensing, or arrhythmias. The influence of these parameters on CIEDs can be divided into two groups: transient and permanent impairment of the CIED operation.^614 , 615^

Responses to these sources of interference may vary:

*Static magnetic field* : device dislodgement, sensor activation, sudden loss of device function, ECG changes.
*Gradient magnetic field* : induction of arrhythmias (rare), oversensing or undersensing.
*Radiofrequency field* : heating of tissue adjacent to lead electrodes, induction of arrhythmias (rare), device reprogramming (reset), oversensing or undersensing interactions.
*Combined field effects* : sudden loss of device function, alteration of device function (parameters), mechanical forces (vibration), device reset, damage to generator and/or leads.
*Imaging-related* : artifacts that prevent adequate device image visualization.

Potential interactions between CIEDs and MRI electromagnetic interference include:

*Magnetic field-induced force and torque due to ferromagnetic materials* : generator movement is extremely unlikely due to confinement and adjacent subcutaneous tissues. Leads do not contain sufficient ferromagnetic material to cause movement.
*Gradient magnetic field-induced electrical current* : gradient magnetic fields can induce current, which can lead to myocardial capture and potentially cause atrial or ventricular arrhythmias.
*Heating and tissue damage* : radiofrequency fields can lead to nonconditional CIED component heating, causing heating of and thermal damage to the adjacent tissue (functional ablation). Changes in sensing or capture thresholds can occur as a result of tissue damage near lead electrodes.
*Effects on device activity* : the CIED can be programmed by placement of a magnet, thus allowing device interactions. Magnetic fields might therefore affect the activity of a nonconditional device, possibly changing the programming of the device.
*Electrical reset* : High-energy electromagnetic interference can lead to electrical power-on reset, and a backup demand mode may be activated. Power-on reset parameters vary among vendors and device types and can include a set of variations. Inhibition of pacing function by MRI-generated signals or pacing at an output below threshold (bipolar or unipolar) in a PM-dependent patient may occur. Additionally, battery status can be affected, particularly for devices that are near an elective replacement interval (ERI), which may result in unreliable function.
*Inappropriate function and therapies* : electromagnetic interference from radiofrequency energy pulses or rapidly changing magnetic field gradients might cause oversensing that can lead to inappropriate pacing inhibition and possibility asystole in PM pacing-dependent patients, or induction of therapies leading to inappropriate shocks in patients with ICDs.

These effects are influenced by many factors, including magnetic field strength, radiofrequency power, position of the patient and device inside the MRI bore, device characteristics, and the size of the patient.

Traditionally, MRI scanning has been contraindicated in patients with CIEDs. The first MRI-conditional system was introduced in Europe in 2010 and approved by the Food and Drug Administration (FDA) in 2011 for use in the United States.^613 , 616^

To render CIEDs MRI conditional, structural changes (eg, use of non-ferromagnetic materials) and software changes have been made to reduce or eliminate potential adverse effects. Once the special programming mode (MRI mode) has been activated, the device reverts to an asynchronous pacing mode and increases the pacing outputs to avoid inhibition of pacing during MRI scanning. In ICDs, the antitachycardia function is temporarily disabled. Therefore, patients with ICDs will be unprotected from ventricular arrhythmias during MRI scanning.

Because the decision to perform MRI scanning in a patient with a CIED system involves risks and benefits, potential risk factors should be identified. Patients with MRI-conditional CIEDs may undergo MRI scanning without additional risks if established recommendations and protocols are followed.^617 , 618^

Before MRI scanning, it is important to identify the patient’s baseline rhythm and whether the patient is PM dependent, activate the specific MRI programming mode, confirm that the entire system is MRI conditional, and check for the presence of abandoned or epicardial leads.

In general, most CIED systems have been approved for MRI scanning with 1.5T, a gradient slew rate of 200 T/m/s, a maximum specific absorption rate of 2 W/kg, and a limited number and length of imaging sequences. New devices allow safe MRI scanning under broader conditions. Most new systems allow full-body MRI scanning.

An MRI-conditional system consists of a combination of leads and generator that has been specifically tested to ensure safe conditions of use during MRI scanning. The presence of any device component that does not meet the criteria for MRI conditionality renders the CIED MRI nonconditional. This includes an MRI-conditional generator combined with nonconditional components and device systems that combine individual MRI lead components and MRI-conditional generators from various manufacturers, as these are not combinations specifically tested together for MRI scanning safety.

Conditional labeling also specifies the location of the generator (eg, pectoral location for a transvenous system). Other examples of nonconditional components include epicardial leads, abandoned leads, fractured leads, and active noncardiac devices.

Programming of the device outside the MRI-conditional programming mode also renders the device MRI nonconditional. Battery status must be adequate to consider the device MRI conditional ( Table 55 ).

**Table 55:** – Recommendations for the prevention of electromagnetic interference in MRI scanning

	Class of recommendation	Level of evidence
MRI-conditional CIEDs should be considered MRI conditional only when the product labeling is adhered to, which includes programming the appropriate “MRI mode” and scanning with the prerequisites specified for the device MRI in a patient with an MRI-conditional system should always be performed in the context of a rigorously applied standardized institutional workflow, following appropriate technical conditions It is recommended for patients with an MRI-conditional system that ECG and pulse oximetry monitoring be continued until patient observation is completed, or until other clinically appropriate device settings are restored	I	A
It is recommended for patients with an MRI-nonconditional CIED that device evaluation be performed immediately before and after the scan, with documentation of pacing threshold, P- and R-wave amplitude, and lead impedance using a standardized protocol A defibrillator/monitor (with external pacing function) and a manufacturer-specific CIED programming system should be immediately available in the area adjacent to the scanner room while the MRI-nonconditional device is programmed for imaging It is recommended that continuous ECG and pulse oximetry monitoring be used while the MRI-nonconditional device is programmed for imaging It is recommended that personnel skilled to perform advanced cardiac life support accompany the patient with an MRI-nonconditional CIED until assessed and declared stable to return to unmonitored status For patients with an MRI-nonconditional CIED who are pacing-dependent (PM or ICD), it is recommended that: a) a physician skilled to implant a temporary PM be immediately available on the premises of the facility; b) a physician skilled to program the CIED be immediately available on the premises of the facility It is recommended for patients with an MRI-unconditional CIED who are pacing-dependent that the device be programmed to an asynchronous pacing mode with deactivation of adaptive features (rate-response sensor) during MRI scanning. The appropriate pacing rate should be selected to avoid competitive pacing All tachycardia detections for patients with an ICD should be disabled prior to MRI	I	B
It is recommended for patients with an MRI-conditional system that personnel with advanced cardiac life support skills be in attendance. If advanced cardiac life support is required, the patient should be monitored for the duration of time the CIED is reprogrammed, or until the patient is assessed and declared stable to return to unmonitored status The MRI-responsible physician should be informed of the presence of a patient with an MRI-nonconditional CIED It is recommended that ECG and pulse oximetry monitoring be continued until the end of MRI scanning or until the device settings are reprogrammed All resuscitative efforts and emergency treatments involving the use of a defibrillator/monitor, CIED programming system, or any other MRI-unsafe equipment should be performed after moving the patient outside zone 4	I	C
It is reasonable for patients with an MRI-nonconditional system to undergo MRI if there are no fractured, epicardial, or abandoned leads, and MRI is the best test for the condition. There should be an institutional protocol and a responsible MRI physician and CIED physician It is reasonable to perform MRI immediately after implantation of a lead or generator of an MRI-nonconditional device if clinically warranted For a patient with an MRI-nonconditional CIED who is not pacing-dependent, it is possible to program the device to either a nonpacing mode (OVO/ODO) or to an inhibited mode (DDI/VVI), with deactivation of advanced or adaptive features during MRI scanning	IIa	B
It is reasonable to perform MRI on a patient with an MRI-conditional system implanted more recently than the exempt period for conditionality of the system, based on the risk-benefit assessment for that patient For patients with an MRI-nonconditional CIED, it is possible to perform repeat MRI when required, without restriction as to the minimum interval between imaging studies or the maximum number of studies performed It is reasonable to program patients with an MRI-nonconditional CIED who are not pacing-dependent to an asynchronous pacing mode (VOO/DOO) with deactivation of advanced or adaptive features during scanning, and with a pacing rate that avoids competitive pacing For patients with an MRI-nonconditional CIED, it is reasonable to schedule a complete device evaluation within 1 week for a pacing lead threshold increase > 1.0 V, P-wave or R-wave amplitude decrease > 50%, pacing lead impedance change > 50 Ω, and shock lead impedance change > 5 Ω	IIa	C

*CIED: cardiac implantable electronic device; ECG: electrocardiogram; ICD: implantable cardioverter-defibrillator; MRI: magnetic resonance imaging; PM: pacemaker.*

### 8.3. Radiotherapy

An increasing number of patients undergoing radiotherapy have a CIED. Although radiotherapy-induced malfunction is rare, safety recommendations are important.

Ionizing radiation can interfere with the complementary metal oxide semiconductor (CMOS) components of the generator. The production of secondary neutrons is the strongest predictor of CIED malfunction in the setting of radiotherapy. Modern pulse generators have lower power consumption and smaller circuits, made of semiconductor metal. This renders modern devices more susceptible to possible damage caused by ionizing radiation.^596 , 619^

High radiation doses, especially with energy > 6MV, can cause software and hardware errors. These disturbances are usually transient, such as pacing inhibition, sensing abnormalities, and inappropriate pacing at the maximum sensor rate. Reset to backup mode, which can be corrected with reprogramming, is one of the most reported malfunctions. Permanent damage to the device may also occur, such as loss of telemetry and premature battery depletion. CIED failure, with complete interruption of device functioning, has been described in vitro.^593 , 619 - 621^

It is also important to consider that damage to the CIED may appear weeks or months after the end of radiotherapy (latent damage).^622^Device malfunction has been reported in up to 3% of radiotherapy courses. Clinically relevant events are rare and dependent on the type of device and patient’s tolerance to changes. For example, a PM-dependent patient may have bradycardia and associated symptoms.^593 , 619^

Radiotherapy planning should consider the conditions specified for the CIED and patient characteristics, such as whether the patient is pacing-dependent or not and has a history of ventricular tachycardia or ventricular fibrillation (VT/VF) ( Table 56 ).

**Table 56:** – Recommendations for the prevention of electromagnetic interference in radiotherapy

	Class of recommendation	Level of evidence
Prior to the initiation of RT, a complete CIED evaluation should be performed and the treatment team should be informed of: Whether the device is a PM or ICD Whether the patient is pacing-dependent The minimum programmed pacing rate The maximum programmed tracking and sensor rates Non-neutron-producing RT is preferred over neutron-producing treatment to minimize the risk of causing CIED malfunction, such as generator reset Complete CIED evaluation should be performed weekly for patients undergoing neutron-producing RT Complete CIED evaluation should be performed at the end of the course of RTs	I	B
It is recommended that CIED interrogation and evaluation be performed at 1, 3, and 6 months after the end of RT due to the risk of latent damage	I	C
CIED relocation is recommended if the generator is situated in the path of the radiation beam	IIa	C
CIED relocation is not recommended for devices receiving a cumulative dose <5 Gy	III	B

*CIED: cardiac implantable electronic device; ICD: implantable cardioverter-defibrillator; PM: pacemaker; RT: radiotherapy.*

## 9. Conclusion

Much scientific evidence has emerged since the latest Brazilian guidelines for CIEDs were published by SOBRAC/SBC. Advances in technology and knowledge must be in line with clinical practice and public health care. In this respect, the present document highlights the evolution of the treatment of cardiac arrhythmias, but it does not shy away from highlighting the pressing need for the rational use of financial resources in favor of the greater good, that is, collective health.
